# α-Amylase immobilization on amidoximated acrylic microfibres activated by cyanuric chloride

**DOI:** 10.1098/rsos.172164

**Published:** 2018-11-28

**Authors:** Yaaser Q. Almulaiky, Faisal M. Aqlan, Musab Aldhahri, Mohammed Baeshen, Tariq Jamal Khan, Khalid A. Khan, Mohamed Afifi, Ammar AL-Farga, Mohiuddin Khan Warsi, Mohammed Alkhaled, Aisha A. M. Alayafi

**Affiliations:** 1 Department of Biochemistry, Faculty of Science, University of Jeddah, Jeddah, Saudi Arabia; 2 Chemistry Department, Faculty of Science, University of Jeddah, Jeddah, Saudi Arabia; 3 Department of Biology, Faculty of Science, University of Jeddah, Jeddah, Saudi Arabia; 4 Chemistry Department, Faculty of Applied Science, Taiz University, Taiz, Yemen; 5 Department of Biochemistry, Faculty of Science, Faculty of Science, King Abdulaziz University, Jeddah, Saudi Arabia; 6 Center of Nanotechnology, Faculty of Science, King Abdulaziz University, Jeddah, Saudi Arabia; 7 Chemistry Department, Faculty of Science, King Abdulaziz University, Jeddah, Saudi Arabia; 8 Stem Cell P2 Laboratory, The Center for Reproductive Medicine, Shantou University Medical College, Shantou 515041, People's Republic of China; 9 Biochemistry Department, Faculty of Veterinary Medicine, Zagazig University, Egypt

**Keywords:** α-amylase, acrylic fabric, scanning electron microscopy, immobilization, amidoximated

## Abstract

Enzyme immobilization is one of the most important techniques for industrial applications. It makes the immobilized enzyme more stable and advantageous than the free form in different aspects. α-Amylase was immobilized on 4% cyanuric chloride-activated amidoximated acrylic fabric at pH 7.0 with (79%) maximum efficiency. A field emission scanning electron microscope and Fourier transform infrared were used to confirm the immobilization process. Even after being recycled 10 times, the immobilized enzyme lost just 28% of its initial activity. Owing to immobilization, the pH of the soluble α-amylase was shifted from 6.0 to 6.5. The immobilized α-amylases showed thermal stability at 60°C, and became more resistant to heavy metal ions. The *k*_m_ values of the immobilized and soluble α-amylases were 9.6 and 3.8 mg starch ml^−1^, respectively. In conclusion, this method shows that the immobilized α-amylase proved to be more efficient than its soluble form, and hence could be used during saccharification of starch.

## 1. Introduction

The immobilization of enzymes is important for several reasons, and it can be beneficial for enzymatic reactions. During enzyme-substrate reactions, the product can become contaminated with undesirable proteins. However, the immobilization of enzymes enables the complete separation of the enzyme from the product and thus removes the undesirable proteins from the product. Enzymatic immobilization is important for reducing the production cost of industrial processes by facilitating the recovery and reuse of enzymes. Immobilization maintains the integrity of the structures of the enzymes during interactions with detergent or organic solvents at elevated temperatures [1]. α-Amylases are employed in several industrial applications ranging from food processing to drug and pharmaceutical applications [2]. Interestingly, in starch processing, newly developed microbial amylases have replaced the chemical reagents that were commonly used during starch hydrolysis, and this has led to a global increase in the sale of the amylolytic enzymes [3–5]. Similarly, immobilized amylase offers improved stability and reusability through multi-point covalent bonding to the monomeric enzymes [6–9]. Eupergit C, an acrylic resin, is an important support material for the immobilization of enzymes. It binds enzymes using covalent bonds, and is widely used in industrial applications [1,10]. Polymers other than Eupergit C that are widely used as substrates for the immobilization of α-amylase include magnetic poly(2-hydroxyethylmethacrylate) and treated wool [11,12]. During the biochemical cross-linking of enzymes with resins, hydroxylamine hydrochlorides are used as a strong reducing agent. To prepare the amidoxime polyacrylonitrile (PAN) nanofibrous membranes where PAN nanofibrous membrane acts as a base material, aqueous hydroxylamine hydrochloride aqueous is used [13]. The hydrophilic nature of amidoxime groups helps improve the biological and chemical properties of the PAN nanofibrous membranes. Various applications of PAN nanofibrous membranes have been reported in environmental and biological processes, including metal ion adsorption, cell adhesion and enzyme immobilization [14–17]. In the cross-linking of enzymes with supports, cyanuric chloride (2,4,6-trichloro-1,3,5-triazine) acts as a coupling reagent [12,18,19]. In the molecule, the chlorine atoms react with the nucleophilic groups to form stable linkages.

The hydrolysis of starch using α- and β-amylases leads to the formation of glucose, maltose and dextrin [20]. α-Amylases are considered as a commercially important enzymes for the catalytic hydrolysis of α-1,4-glycosidic linkages in starch in industrial application [5]. α-Amylase is used in various industries, including beer and other drink manufacturing, the designing of fabrics in the textiles, pulp and paper industry and in the analysis of experiments in the medicinal and clinical chemistry fields [3]. However, following the enzymatic reaction, the α-amylase cannot be completely recovered from the reaction systems. Hence, it is highly important to employ immobilized *α*-amylase to minimize the loss of *α* -amylase over the course of the reaction [21].

In this study, we synthesized amidoximated microfibres by treating acrylic microfibers with hydroxylamine hydrochloride. We used various concentrations of cyanuric chloride to activate the amidoximated microfibres. To provide stability and retain the structure of the α-amylase under different chemical and physical conditions, we have immobilized it on the activated acrylic microfibres.

## 2. Material and methods

### 2.1. α-Amylase

α-Amylase from *Bacillus subtilis* was purchased from Sigma-Aldrich.

### 2.2. Acrylic fabrics

The acrylic fabrics used in this study were 1/1 woven acrylic (40.6 × 40.6 threads inch^−1^ for both weft and warp) with densities of 0.36 g cm^−3^, and they were supplied by Misr El-Mehalla Co., Egypt. The fabric was washed with ethanol three times and dried at room temperature.

### 2.3. α-Amylase assay

To determine the α-amylase activity, we adopted the method described by Miller [22], for both the immobilized and soluble enzymes. In the standard assay procedure, a 1 cm^2^ section of acrylic fabric was used to determine the activity of immobilized enzyme on activated acrylic microfibres. The immobilized α-amylase was incubated at 37°C for 30 min with 1 ml of starch (1%) and for colour development; 1 ml of dinitrosalicylic acid (DNS) reagent was used. The immobilized enzyme was removed from the reaction mixture and washed with distilled water before adding DNS reagent. This reaction mixture was incubated, and the absorbance was finally recorded at 560 nm. The amount of enzyme required to produce 1 µmol of maltose min^−1^ is defined as one unit of activity.

### 2.4. Preparation of support

The pretreatment of a particular weight of acrylic fibre using 1% hydroxylamine hydrochloride and 2% aqueous ammonium acetate at a liquor-to-goods ratio of 50 : 1 was performed at 85°C for 1 h. The pretreated sample was carefully rinsed in water and air-dried. Different weight percents of cyanuric chloride (2–6% w/w of substrate) in a water/acetone mixture (50% v/v) were used to activate the amidoxime acrylic microfibres at 0°C for 2 h. After that, the substrate was rinsed with acetone and cold water and allowed to dry in a ventilated refrigerator prior to immobilization of the enzyme.

### 2.5. Immobilization procedure

The treated microfibres were used in the immobilization of *α*-amylase dissolved in Tris–HCl buffer (pH 8.5, 7.0) or 50 mM sodium acetate buffer (pH 4.0). The reaction was carried out at room temperature overnight. The microfibres were dried at room temperature until the aliquots of the enzymatic supernatant were dry; the evaporation was used to monitor the progression of the immobilization.

The following formula was used to calculate the immobilization efficiency (%):

$$\text{Immobilization efficiency}\;(\text{\%})=\left(\frac{\text{Activity of immobilized enzyme}}{\text{Initial activity of enzyme}}\right)\times 100.$$

### 2.6. Morphological characterization

A field emission scanning electron microscope (FESEM, Jeol 7600) was used to examine the morphological features of the immobilized enzyme using accelerating voltages of 10 and 20 kV. A PerkinElmer spectrum 100 Fourier transform infrared (FTIR) spectrometer was used to obtain the FTIR spectrum of the immobilized enzyme.

### 2.7. Reusability of immobilized enzyme

The immobilized enzyme could be recycled up to 10 times. The initial activity was taken as the control (100%) to calculate the percentage activity during each repeated use.

### 2.8. Physico-chemical characterization of the enzyme

#### 2.8.1. Effect of temperature

The temperature ranges from 20 to 80°C, under standard assay conditions, were taken as the optimal ranges for the synthesis of soluble α-amylase and immobilized α-amylase. For measuring the thermal stability, the activities of immobilized and soluble α-amylase were estimated after 15 min of incubation at different temperatures before the addition of the substrate. The percent relative activity was plotted against different temperatures.

#### 2.8.2. Effect of pH

The pH ranges from 4.0 to 9 were taken as the optimal ranges for the synthesis of soluble α-amylase and immobilized α-amylase under standard assay conditions.

#### 2.8.3. Kinetic properties

The Line weaver–Burk plots were prepared using various concentrations of starch as the substrate to calculate the *k*_m_ values.

#### 2.8.4. Substrate specificity

The investigation of the substrate specificity was performed by incubating the soluble and immobilized α-amylases with glycogen, α-cyclodextrine, starch, amylopectin, amylose and β-cyclodextrin. The enzyme activity with starch was taken as 100%

### 2.9. Effect of metal ions

The effect of metal ions on the enzymatic activity of the immobilized and soluble α-amylases was determined by incubating the enzyme with 2 mM of metal ions for 15 min before adding the starch. The activity without metal ion incubation was taken as the control (100%).

### 2.10. Effect of metal chelators and inhibitors

The activities of immobilized and soluble α-amylase were determined in the presence of 2 mM EDTA, sodium citrate, sodium oxalate and metal chelators, 1,10 phenanthroline monohydrate and inhibitors dithiobis (2-nitrobenzoic acid) (DTNB). The enzyme activity in the absence of chelator or inhibitor was taken as 100% and percentage of the relative activity was determined in the presence of each compound.

## 3. Results and discussion

**Scheme 1.**
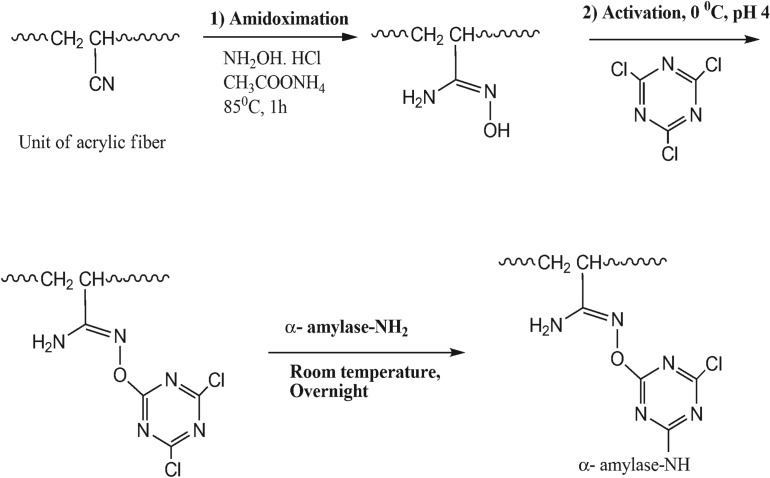
Amidoximation, activation and immobilization of α-amylase onto acrylic fibre.

**Figure 1.**
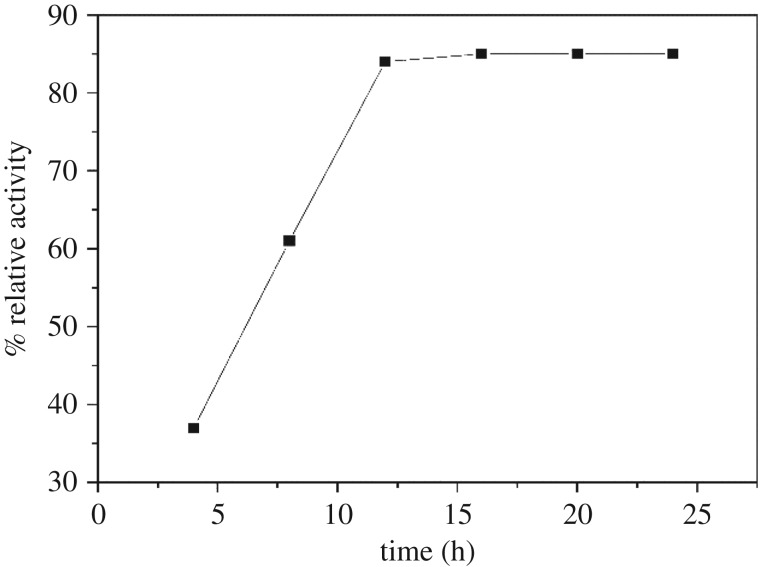
Effect of immobilization time on the relative activity of the immobilized α-amylase.

**Figure 2.**
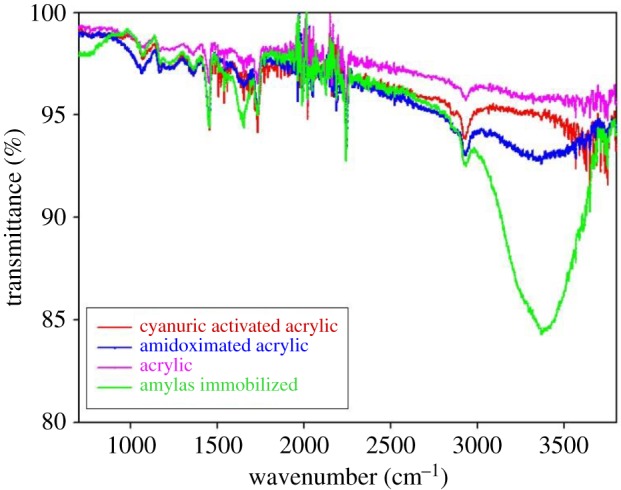
FTIR spectra of acrylic, amidoximated acrylic, activated acrylic and immobilized α-amylase fabric samples.

The facile nature of amidoximation allowed the study of the immobilization of α-amylase using a triazinyl reactive centre as an activating agent (scheme 1) [23]. Bickerstaff [24] reported the importance of treated polymer in the immobilization of enzyme. Treated polymers are important due to their high mechanical and chemical stabilities and enhanced capacity for enzyme immobilization, and they allow the simple access to the active sites of the enzyme. We used hydroxylamine hydrochloride and acrylic microfibres to produce amidoxime acrylic microfibres, which served as the base for the immobilization of the enzyme. The immobilization of the enzyme onto acrylic microfibres using cyanuric chloride as coupling reagent enhances the efficiency of the enzyme. This is the first study reporting the immobilization of α-amylase using acrylic microfibres, hydroxylamine hydrochloride and cyanuric chloride. Here, we have studied the immobilization of α-amylase on amidoxime acrylic microfibres using various concentrations of cyanuric chloride at different pH levels. The maximum efficiency of the immobilized α-amylase (79%) was observed with 4% cyanuric chloride and pH 7.0 (table 1). Whereas, the lowest efficiency of the immobilized α-amylase was observed with 6% cyanuric chloride and pH 4.0 or pH 8.5. Previous studies reported increasing concentrations of cyanuric chloride cause decreased retention of enzymatic activity [12,25,26]. We demonstrated the effect of immobilization time on the relative activity of the immobilized α-amylase. Figure 1 shows the initial rapid increase in the activity of the immobilized enzyme, but the activity plateaued at 16 h of immobilization. The plateau was due to saturation of the carrier with the enzyme after 16 h. However, the carrier became saturated with horseradish peroxide after 11 h [27] or 6 h [25]. Figure 2 shows the (ATR-FTIR) spectra of the acrylic fabric, activated acrylic fabric, amidoximated acrylic fabric and immobilized enzyme. The spectra of the activated amidoximated acrylic fabric and the fabric with the immobilized enzyme were remarkably different from the spectrum of the untreated acrylic fabric. A characteristic nitrile band was observed in all the samples at approximately 2244 cm^−1^ with noticeable differences. In the spectrum of the amidoxime-treated acrylic fabric, the intensity of the nitrile absorption peak was lower and the peak was red-shirted to 2241 cm^−1^ relative to the untreated sample. Owing to the amidoxime group, the newly formed bands from the N–O and C=N stretching vibrations were observed at 1071 cm^−1^ and 1659 cm^−1^, respectively. The absorption bands of the treated samples at 3350 cm^−1^ showed more pronounced overlap with the vibrations of the OH and NH_2_ moieties of the amidoxime group than they did in the spectrum of the acrylic sample. The changes in the intensities of the bands at 1673 and 3350 cm^−1^ of the treated samples are correlated with the order of the chemical treatment. The reaction of amidoximated acrylic fabric with cyanuric chloride decreases the intensities of the bands due to the formation of covalent bonds between the OH and/or NH_2_ groups.

**Table 1.** Effect of cyanuric chloride percentage and pH on the immobilization efficiency of α-amylase. Each point represents the mean of three experiments ± s.d.

cyanuric chloride (%)	immobilization efficiency (%)		
	pH 8.5	pH 7	pH 4
2	16 ± 0.015	38 ± 0.043	21 ± 0.011
4	23 ± 0.024	79 ± 0.061	18 ± 0.015
6	27 ± 0.011	42 ± 0.021	15 ± 0.035

However, the immobilization of α-amylase on to the activated acrylic fabric increases the absorption intensity of the bands due to overlap with the vibrations of the OH and/or NH_2_ groups in α-amylase. The triazinyl ring present in the immobilized and activated enzymes is characterized by the vibrations observed at 1455 cm^−1^ and above 1550 cm^−1^ [12,25,28]. The above results confirm the successful synthesis of the final acrylic-α-amylase product along with the success of each of the chemical treatments involved.

**Figure 3.**
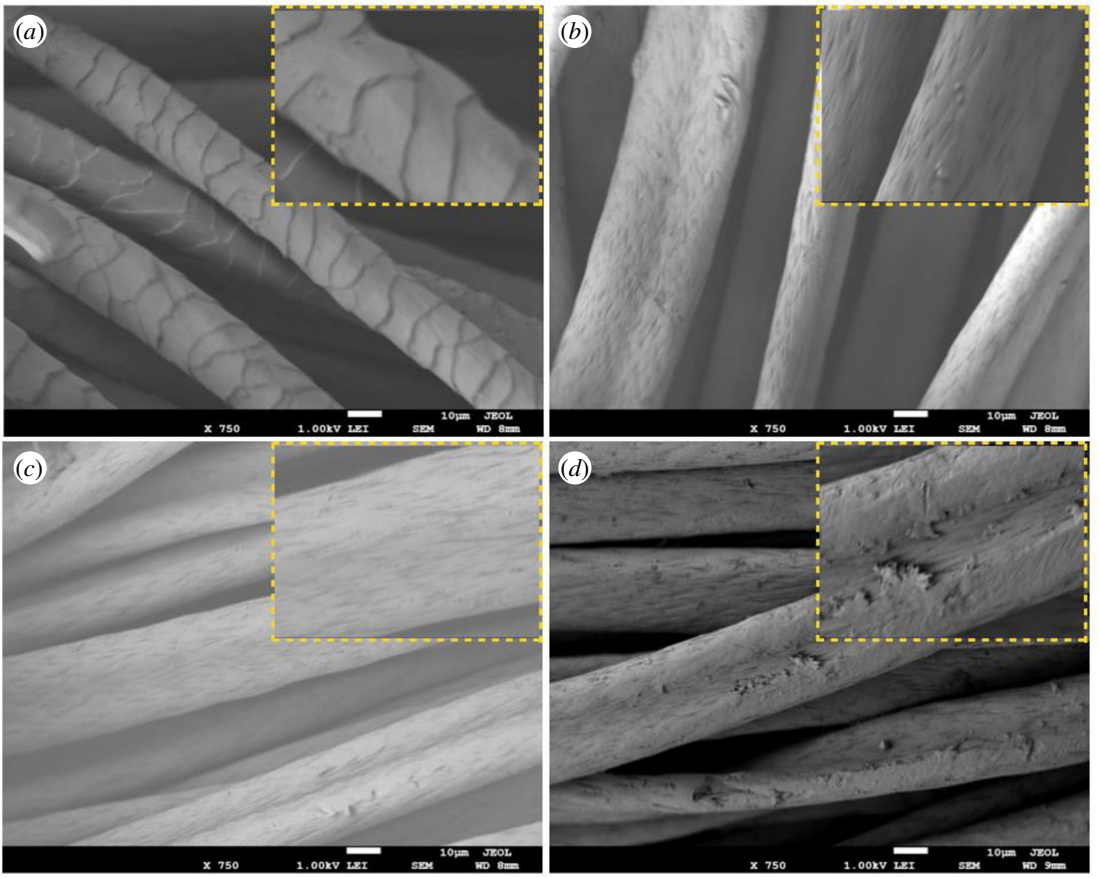
Low and high magnification (inset) FESEM images of *(a*) pure acrylic fibre, (*b*,*c*) acrylic fibres treated with NH_2_OH · HCl and cyanuric chloride, respectively, (*d*) immobilization of α-amylase after chemical treatment.

Figure 3 shows the structural surface of the amidoximated acrylic, cyanuric-activated fabric, α-amylase-acrylic fabric and pristine acrylic fabric samples. Two different images taken at different magnifications show the morphological changes at different times. Figure 3*a* shows the cuticle layer and edges on the surface of the pristine acrylic fibres. Relative to those shown in figure 3*a*, the structural surface of amidoxime-treated acrylic fibres is better defined but less compact (figure 3*b*). Furthermore, we did not observe a substantial difference between the structural surfaces of the cyanuric chloride-activated acrylic fibres; however, a clearer surface was observed in figure 3*c*. The immobilization of α-amylase generated clear coating on the surface of the acrylic-treated fibres, which indicates the presence of α-amylase enzyme and this can be seen in figure 3*d*.

**Figure 4.**
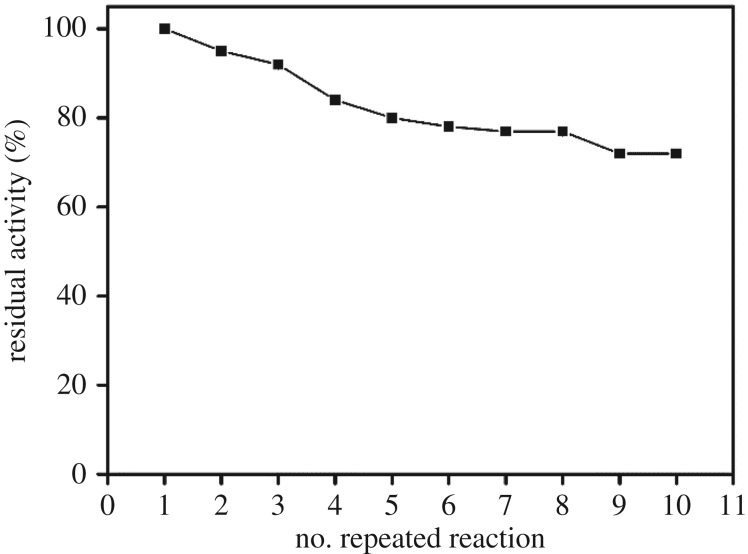
Reuse of immobilized α-amylase.

**Figure 5.**
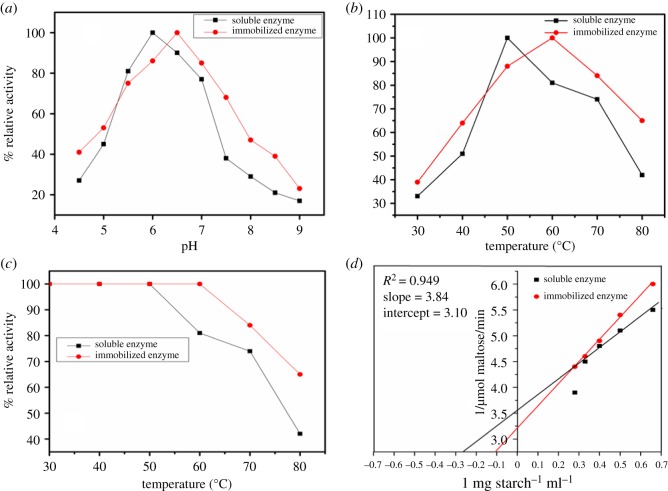
Optimum pH (*a*), optimum temperature (*b*), thermal stability (*c*) and *k*_m_ (*d*) of soluble and immobilized α-amylase. Each point represents the average of two experiments.

The immobilization was important for the recycling of the enzymes. In figure 4, we show the measured activities of the immobilized α-amylase and its reusability as determined by using the same experimental conditions through 10 reaction cycles. Even after being re-used 10 times, the immobilized enzyme was found to have retained 72% of its initial activity, which indicated the stability and reusability of the immobilized α-amylase [12,29,30]. Akhtar *et al.* [31] and Qiu *et al.* [32] suggested that the high concentrations of substrates and the damage to the support material have a role in the decreased enzymatic efficiency observed during recycling. Incubating buffers with pH levels between 4.0 and 9.0 were used to evaluate the effect of pH on the activity of immobilized α-amylase and soluble α-amylase (figure 5*a*).

The shift in pH was observed from 6.0 for soluble α-amylase to 6.5 for immobilized enzyme. In the immobilized enzymes, the enzymes were fixed to the support material through multiple cross-linking interactions, which stabilized the structure of the overall enzyme and protected it from pH and temperature changes [1].The fundamental reason behind the altered properties of the amylase after immobilization was because of the binding between the support material and enzyme, which led to conformational changes in the amylase [29]. Post-immobilization, the optimum pH had increased from 6 to 8 [11,33,34] or 11 [35] depending on the study.

The changes in the activities of soluble and immobilized α-amylase with temperature are shown in figure 5*b*. The optimum reaction temperatures for the immobilized and soluble α-amylases were 50°C and 60°C, respectively. The soluble α-amylase exhibited approximately 42% of its initial activity, whereas immobilized α-amylase retained 65% of its activity at 80°C. Previous studies reported similar results for optimum temperatures [12]. Protein denaturation began higher temperatures in the soluble enzymes relative to immobilized enzymes [36]. The increased number of alterations to the structure of the enzymes at the optimum temperature was caused by changes in the physico-chemical properties of the enzyme. A higher activation energy and increased substrate binding were reported for immobilized α-amylase due to covalent bond formation. Following immobilization, the anticipated increase in the stability of the enzyme against deactivating forces was noticed due to reduced conformational mobility of the enzymes [37,38]. Several studies have reported increased optimum temperatures following enzyme immobilization [20,29]. Figure 5*c* shows the thermal stabilities of the immobilized and soluble α-amylase. Our results indicated immobilized α-amylase was more stable than the soluble form in the temperature range of 30–80°C. Several studies reported similar improvements in thermal stability for various immobilized enzymes [29,34]. The multi-point complexation of the support and the enzyme may lead to an enhancement of the thermal stabilities of immobilized enzymes [39]. For industrial applications, immobilized enzymes prefer the soluble form of the enzymes, because the immobilized form can withstand the high temperatures that are important for enzyme catalysis [40]. Relative to the soluble form, the immobilized α-amylase was more efficient in the hydrolysis of amylopectin, starch, α-cyclodextrine, β-cyclodextrin, glycogen and amylose (table 2). This indicates that the immobilization of the enzyme did not alter the binding site of the substrate in the enzyme. Akkaya *et al*. [35] reported similar findings. Mohamed *et al.* [12] reported that the highest activities of the free and immobilized α-amylases were observed when starch was used as the substrate. The immobilized enzyme showed less affinity towards substrates than the free enzyme due to ionic strength, steric effects and diffusional restrictions [38]. The reduced affinity of the immobilized enzyme for the substrate relative to that of the soluble form could be due to the high concentration of immobilized proteins necessary to produce the diffusion effects or altered active sites from contacting the solid surface of the support material [41]. The apparent *k*_m_ values of the immobilized and the soluble α-amylase were 9.6 and 3.8 mg starch ml^−1^, respectively, and the *V*_max_ values were 0.281 and 0.311 µmol maltose ml^−1^, respectively (figure 5*d*). For starch, the *k*_m_/*V*_max_ of the immobilized and soluble α-amylase were 30.7 and 13.57, respectively. The *k*_m_/*V*_max_ shows that the affinity of soluble α-amylase towards starch is higher than that of the immobilized α-amylase. Previous studies have reported higher *k*_m_ values for immobilized enzymes compared to the corresponding soluble enzymes [25,42,43]. Immobilization can enhance the stability of the enzymes against inactivation caused by interactions with metal ions [44,45]. The immobilized α-amylase is more resistant to heavy metal ions than the soluble form of the enzyme (table 3). The interaction of heavy metals, including Ca^2+^, Ni^2+^ and Co^2+^, activated the immobilized α-amylase more than it did the soluble α-amylase. The other metal ions inhibited the immobilized α-amylase to a lower extent than they did the soluble enzyme. Several studies have shown that immobilization protects α-amylase from strong inhibition due to heavy metal ions [12,46]. Metal chelators can strongly inhibit the amylases because they are metallo enzymes. The Ca^2+^ in the cereal amylases is loosely bound to the enzyme, and it can thus be removed by EDTA (a metal chelator) [47]. The immobilized α-amylase was much more resistant to interference from EDTA than soluble α-amylase. Conversely, the sodium citrate, 1,10 phenanthroline, DTNB and sodium oxalate had a mild inhibitory effect on the immobilized α-amylase relative to the soluble form of enzyme (table 4).

**Table 2.** The substrate specificity of soluble and immobilized α-amylase. The activity with starch as the substrate is regarded as 100% activity. Each point represents the mean of three experiments ± s.d.

substrate	soluble α-amylase	immobilized α-amylase
starch	100 ± 0.75	100 ± 0.69
glycogen	81 ± 0.25	90 ± 0.82
amylopectin	61 ± 0.53	86 ± 0.57
α-cyclodextrine	40 ± 0.24	48 ± 0.26
β-cyclodextrin	26 ± 0.19	65 ± 0.37
amylose	17 ± 0.10	28 ± 0.44

**Table 3.** The effect of 2 mM metal ions on the activities of the soluble and immobilized α-amylase. Each point represents the mean of three experiments ± s.d.

metals 2 mM	soluble α-amylase	immobilized α-amylase
control	100 ± 1.05	100 ± 0.98
Ni^2+^	86 ± 0.76	120 ± 0.82
Ca^2+^	102 ± 0.8	123 ± 1.12
Cu^2+^	54 ± 0.71	81 ± 0.29
Co^2+^	98 ± 0.46	108 ± 0.70
Zn^2+^	63 ± 0.28	76 ± 0.59
Hg^2+^	21 ± 0.12	49 ± 0.48
Pb^2+^	57 ± 0.25	70 ± 0.50

**Table 4.** Effect of 2 mM metal chelating agents and inhibitors on the activities of the soluble and immobilized α-amylase. Each point represents the mean of three experiments ± s.d.

inhibitor 2 mM	soluble α-amylase	immobilized α-amylase
EDTA	18 ± 0.08	60 ± 0.11
sodium citrate	45 ± 0.12	70 ± 0.29
sodium oxalate	59 ± 0.52	76 ± 0.47
1,10 phenatroline	60 ± 0.34	88 ± 0.53
DTNB	61 ± 0.41	84 ± 0.76

## 4. Conclusion

In this study, immobilization on ancyanuric chloride-activated amidoxime acrylic microfibres protected α-amylase from denaturation and loss of activity induced by metal ions, pH, heat, inhibitors and metal chelating agents. Immobilized and soluble α-amylase showed similarly high efficiencies against numerous substrates, which indicates a minimal effect on the substrate-binding site of the enzyme. Enzyme immobilization could be applicable in several industries, particularly during the saccharification of starch.

## Supplementary Material

Supplementary Figure 1

## Supplementary Material

Supplementary Figure 2

## Supplementary Material

Supplementary Figure 4

## Supplementary Material

Supplementary Figure 5

## Supplementary Material

Supplementary Table 1

## Supplementary Material

Supplementary Table 2

## Supplementary Material

Supplementary Table 2

## Supplementary Material

Supplementary Table 4

## Data accessibility

The FTIR spectra and other data for work replication are included in the electronic supplementary material.
