# Supplementary material for: α-Amylase immobilization on amidoximated acrylic microfibres activated by cyanuric chloride
Source: R Soc Open Sci. 2018 Nov 28;5(11):172164. doi: 10.1098/rsos.172164 (PMC6281920; doi:10.1098/rsos.172164)
Supplement: Supplementary Figure 1 [file rsos172164supp1.pdf]

A-Amylase immobilization on amidoximated acrylic microfibers activated by cyanuric chloride

Yaaser Q. Almulaiky[1,2], Faisal M. Aqlan[3], Musab Aldhahri[4,5], Mohammed Baeshen[6] Tariq Jamal Khan[7], Khalid A. Khan[8], Mohamed Afifi[6,9], Ammar AL-Farga[1], Mohiuddin Khan Warsi[1], Mohammed Alkaled[6], Aisha A.M. Alayafi[6]

[1]Department of Biochemistry, Faculty of Science, University of Jeddah, Jeddah, Saudi Arabia
[2]Chemistry Department, Faculty of Applied Science, Taiz University, Taiz, Yemen
[3]Chemistry Department, Faculty of Science, University of Jeddah, Jeddah, Saudi Arabia
[4]Department of Biochemistry, Faculty of Science, King Abdulaziz University, Jeddah, Saudi Arabia
[5]Center of Nanotechnology, King Abdulaziz University, Jeddah, Saudi Arabia
[6]Department of biology, Faculty of Science, University of Jeddah, Jeddah, Saudi Arabia
[7]Stem Cell P2 Laboratory, The Center for Reproductive Medicine, Shantou University Medical College, Shantou, 515041, People‘s Republic of China
[8]Chemistry Department, Faculty of Science, King Abdulaziz University, Jeddah, Saudi Arabia
[9]Biochemistry Department, Faculty of Veterinary Medicine, Zagazig University, Egypt

Supplementary Figure 1

Time hour	Residual activity %	OD at 560nm (n/3)
4	37	0.277
8	61	0.458
12	84	0.630
16	85	0.634
20	85	0.629
24	85	0.631

Supplementary Figure 1 Effect of immobilization time on the relative activity of the immobilized α-amylase.The effect of immobilization time was carried out by determining the activity of enzyme after immobilization in different time. a 1cm2 section of acrylic fabric was used to determine the activity of immobilized enzyme on activated acrylic microfibers. The immobilized α-amylase was incubated at 37 °C for 30 min with 1 ml of starch (1%), 1ml of 50 mM sodium acetate buffer 5.5; 1 ml of dinitrosalicylic acid (DNS) reagent was used. The immobilized enzyme was removed from the reaction mixture and washed with distilled water before added DNS reagent.
