# Supplementary material for: α-Amylase immobilization on amidoximated acrylic microfibres activated by cyanuric chloride
Source: R Soc Open Sci. 2018 Nov 28;5(11):172164. doi: 10.1098/rsos.172164 (PMC6281920; doi:10.1098/rsos.172164)
Supplement: Supplementary Figure 2 [file rsos172164supp2.pdf]

Supplementary Figure 2,
FTIR

399.6813	0.00E+00	0.00E+00	0.00E+00
400.1635	9.86E+01	9.94E+01	9.95E+01
400.6456	9.84E+01	9.95E+01	9.95E+01
401.1277	9.84E+01	9.95E+01	9.97E+01
401.6098	9.85E+01	9.96E+01	9.97E+01
402.0919	9.86E+01	9.96E+01	9.98E+01
402.5741	9.88E+01	9.96E+01	9.98E+01
403.0562	9.90E+01	9.96E+01	9.98E+01
403.5383	9.91E+01	9.95E+01	9.97E+01
404.0204	9.92E+01	9.94E+01	9.97E+01
404.5026	9.92E+01	9.92E+01	9.97E+01
404.9847	9.93E+01	9.90E+01	9.96E+01
405.4668	9.93E+01	9.89E+01	9.96E+01
405.9489	9.92E+01	9.88E+01	9.95E+01
406.4311	9.92E+01	9.87E+01	9.95E+01
406.9132	9.92E+01	9.87E+01	9.94E+01
407.3953	9.92E+01	9.87E+01	9.94E+01
407.8774	9.92E+01	9.88E+01	9.94E+01
408.3596	9.92E+01	9.88E+01	9.93E+01
408.8417	9.91E+01	9.89E+01	9.93E+01
409.3238	9.92E+01	9.89E+01	9.93E+01
409.8059	9.92E+01	9.88E+01	9.94E+01
410.2881	9.93E+01	9.87E+01	9.94E+01
410.7702	9.93E+01	9.87E+01	9.94E+01
411.2523	9.94E+01	9.87E+01	9.95E+01
411.7344	9.95E+01	9.87E+01	9.95E+01
412.2166	9.95E+01	9.88E+01	9.96E+01
412.6987	9.96E+01	9.89E+01	9.96E+01
413.1808	9.96E+01	9.91E+01	9.97E+01
413.663	9.96E+01	9.92E+01	9.97E+01
414.1451	9.95E+01	9.93E+01	9.97E+01
414.6272	9.94E+01	9.93E+01	9.97E+01
415.1093	9.93E+01	9.92E+01	9.96E+01
415.5915	9.91E+01	9.91E+01	9.96E+01
416.0736	9.89E+01	9.90E+01	9.95E+01
416.5557	9.86E+01	9.89E+01	9.94E+01
417.0378	9.83E+01	9.89E+01	9.92E+01
417.52	9.80E+01	9.89E+01	9.91E+01
418.0021	9.78E+01	9.90E+01	9.89E+01
418.4842	9.77E+01	9.92E+01	9.88E+01
418.9663	9.78E+01	9.94E+01	9.88E+01
419.4485	9.82E+01	9.96E+01	9.89E+01
419.9306	9.87E+01	9.97E+01	9.90E+01
420.4127	9.91E+01	9.98E+01	9.92E+01
420.8948	9.94E+01	9.98E+01	9.93E+01
421.377	9.96E+01	9.99E+01	9.94E+01

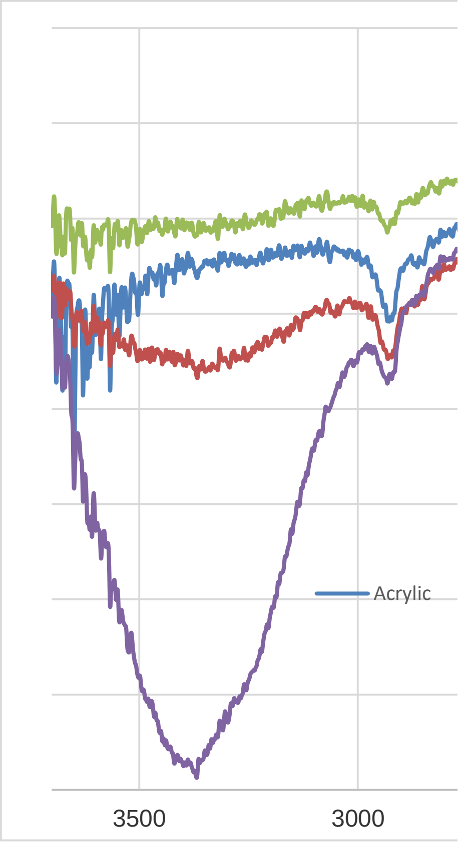

421.8591	9.98E+01	9.99E+01	9.95E+01
422.3412	9.98E+01	9.99E+01	9.95E+01
422.8233	9.98E+01	9.99E+01	9.96E+01
423.3055	9.97E+01	9.98E+01	9.95E+01
423.7876	9.96E+01	9.98E+01	9.95E+01
424.2697	9.95E+01	9.96E+01	9.95E+01
424.7518	9.94E+01	9.95E+01	9.95E+01
425.2339	9.94E+01	9.94E+01	9.95E+01
425.7161	9.94E+01	9.93E+01	9.96E+01
426.1982	9.94E+01	9.93E+01	9.96E+01
426.6803	9.94E+01	9.94E+01	9.97E+01
427.1624	9.94E+01	9.94E+01	9.97E+01
427.6446	9.94E+01	9.95E+01	9.97E+01
428.1267	9.94E+01	9.95E+01	9.97E+01
428.6088	9.94E+01	9.94E+01	9.96E+01
429.0909	9.94E+01	9.93E+01	9.95E+01
429.5731	9.93E+01	9.92E+01	9.93E+01
430.0552	9.93E+01	9.90E+01	9.92E+01
430.5373	9.92E+01	9.89E+01	9.92E+01
431.0194	9.91E+01	9.87E+01	9.91E+01
431.5016	9.90E+01	9.87E+01	9.91E+01
431.9837	9.90E+01	9.86E+01	9.91E+01
432.4658	9.89E+01	9.86E+01	9.92E+01
432.9479	9.89E+01	9.87E+01	9.92E+01
433.4301	9.89E+01	9.87E+01	9.92E+01
433.9122	9.90E+01	9.87E+01	9.92E+01
434.3943	9.90E+01	9.88E+01	9.92E+01
434.8764	9.89E+01	9.88E+01	9.92E+01
435.3586	9.89E+01	9.88E+01	9.91E+01
435.8407	9.88E+01	9.88E+01	9.91E+01
436.3228	9.86E+01	9.88E+01	9.91E+01
436.8049	9.86E+01	9.88E+01	9.91E+01
437.287	9.85E+01	9.88E+01	9.91E+01
437.7692	9.85E+01	9.89E+01	9.91E+01
438.2513	9.86E+01	9.89E+01	9.92E+01
438.7334	9.87E+01	9.90E+01	9.92E+01
439.2155	9.88E+01	9.91E+01	9.93E+01
439.6977	9.89E+01	9.91E+01	9.93E+01
440.1798	9.90E+01	9.92E+01	9.93E+01
440.6619	9.91E+01	9.92E+01	9.92E+01
441.144	9.91E+01	9.93E+01	9.92E+01
441.6262	9.92E+01	9.93E+01	9.92E+01
442.1083	9.92E+01	9.93E+01	9.92E+01
442.5904	9.93E+01	9.92E+01	9.92E+01
443.0725	9.93E+01	9.92E+01	9.93E+01
443.5547	9.93E+01	9.91E+01	9.93E+01
444.0368	9.93E+01	9.90E+01	9.93E+01

444.5189	9.93E+01	9.89E+01	9.94E+01
445.001	9.93E+01	9.88E+01	9.94E+01
445.4832	9.93E+01	9.88E+01	9.94E+01
445.9653	9.93E+01	9.88E+01	9.95E+01
446.4474	9.93E+01	9.88E+01	9.95E+01
446.9295	9.94E+01	9.89E+01	9.96E+01
447.4117	9.94E+01	9.89E+01	9.96E+01
447.8938	9.94E+01	9.90E+01	9.96E+01
448.3759	9.94E+01	9.91E+01	9.96E+01
448.858	9.94E+01	9.92E+01	9.96E+01
449.3402	9.93E+01	9.92E+01	9.95E+01
449.8223	9.92E+01	9.92E+01	9.94E+01
450.3044	9.92E+01	9.92E+01	9.93E+01
450.7865	9.92E+01	9.92E+01	9.93E+01
451.2687	9.92E+01	9.93E+01	9.93E+01
451.7508	9.92E+01	9.93E+01	9.93E+01
452.2329	9.92E+01	9.93E+01	9.93E+01
452.715	9.92E+01	9.94E+01	9.94E+01
453.1972	9.91E+01	9.94E+01	9.94E+01
453.6793	9.90E+01	9.93E+01	9.93E+01
454.1614	9.89E+01	9.93E+01	9.93E+01
454.6436	9.88E+01	9.92E+01	9.92E+01
455.1257	9.88E+01	9.92E+01	9.92E+01
455.6078	9.87E+01	9.91E+01	9.91E+01
456.0899	9.87E+01	9.91E+01	9.91E+01
456.5721	9.87E+01	9.91E+01	9.91E+01
457.0542	9.88E+01	9.91E+01	9.91E+01
457.5363	9.89E+01	9.91E+01	9.91E+01
458.0184	9.90E+01	9.91E+01	9.91E+01
458.5005	9.91E+01	9.91E+01	9.92E+01
458.9827	9.92E+01	9.91E+01	9.93E+01
459.4648	9.93E+01	9.91E+01	9.93E+01
459.9469	9.93E+01	9.90E+01	9.93E+01
460.429	9.93E+01	9.90E+01	9.94E+01
460.9112	9.93E+01	9.90E+01	9.94E+01
461.3933	9.94E+01	9.90E+01	9.94E+01
461.8754	9.94E+01	9.90E+01	9.94E+01
462.3575	9.94E+01	9.90E+01	9.95E+01
462.8397	9.94E+01	9.91E+01	9.95E+01
463.3218	9.94E+01	9.91E+01	9.96E+01
463.8039	9.94E+01	9.92E+01	9.96E+01
464.286	9.94E+01	9.92E+01	9.96E+01
464.7682	9.93E+01	9.92E+01	9.96E+01
465.2503	9.92E+01	9.92E+01	9.96E+01
465.7324	9.91E+01	9.92E+01	9.95E+01
466.2145	9.89E+01	9.91E+01	9.94E+01
466.6967	9.87E+01	9.91E+01	9.94E+01

467.1788	9.86E+01	9.90E+01	9.93E+01
467.6609	9.85E+01	9.90E+01	9.93E+01
468.143	9.85E+01	9.90E+01	9.93E+01
468.6252	9.85E+01	9.90E+01	9.93E+01
469.1073	9.86E+01	9.90E+01	9.92E+01
469.5894	9.86E+01	9.89E+01	9.92E+01
470.0715	9.87E+01	9.89E+01	9.91E+01
470.5536	9.87E+01	9.88E+01	9.91E+01
471.0358	9.87E+01	9.88E+01	9.90E+01
471.5179	9.87E+01	9.87E+01	9.89E+01
472	9.87E+01	9.86E+01	9.89E+01
472.4821	9.88E+01	9.85E+01	9.89E+01
472.9643	9.88E+01	9.85E+01	9.89E+01
473.4464	9.88E+01	9.85E+01	9.89E+01
473.9285	9.89E+01	9.85E+01	9.89E+01
474.4106	9.89E+01	9.85E+01	9.89E+01
474.8928	9.90E+01	9.86E+01	9.89E+01
475.3749	9.91E+01	9.87E+01	9.90E+01
475.857	9.91E+01	9.88E+01	9.91E+01
476.3391	9.92E+01	9.89E+01	9.92E+01
476.8213	9.93E+01	9.90E+01	9.93E+01
477.3034	9.93E+01	9.91E+01	9.94E+01
477.7855	9.94E+01	9.92E+01	9.95E+01
478.2676	9.94E+01	9.93E+01	9.95E+01
478.7498	9.94E+01	9.93E+01	9.95E+01
479.2319	9.94E+01	9.93E+01	9.95E+01
479.714	9.93E+01	9.93E+01	9.95E+01
480.1961	9.93E+01	9.93E+01	9.95E+01
480.6783	9.93E+01	9.93E+01	9.94E+01
481.1604	9.94E+01	9.92E+01	9.94E+01
481.6425	9.94E+01	9.92E+01	9.94E+01
482.1246	9.94E+01	9.92E+01	9.94E+01
482.6068	9.95E+01	9.92E+01	9.94E+01
483.0889	9.95E+01	9.91E+01	9.94E+01
483.571	9.94E+01	9.91E+01	9.93E+01
484.0531	9.93E+01	9.91E+01	9.93E+01
484.5353	9.92E+01	9.90E+01	9.92E+01
485.0174	9.92E+01	9.90E+01	9.92E+01
485.4995	9.91E+01	9.90E+01	9.92E+01
485.9816	9.91E+01	9.90E+01	9.91E+01
486.4638	9.91E+01	9.90E+01	9.91E+01
486.9459	9.91E+01	9.91E+01	9.92E+01
487.428	9.91E+01	9.91E+01	9.92E+01
487.9101	9.91E+01	9.91E+01	9.92E+01
488.3923	9.91E+01	9.91E+01	9.93E+01
488.8744	9.91E+01	9.91E+01	9.93E+01
489.3565	9.90E+01	9.90E+01	9.93E+01

489.8386	9.90E+01	9.90E+01	9.94E+01
490.3208	9.89E+01	9.89E+01	9.94E+01
490.8029	9.89E+01	9.89E+01	9.94E+01
491.285	9.88E+01	9.89E+01	9.93E+01
491.7671	9.88E+01	9.89E+01	9.93E+01
492.2493	9.88E+01	9.89E+01	9.93E+01
492.7314	9.88E+01	9.89E+01	9.93E+01
493.2135	9.88E+01	9.89E+01	9.93E+01
493.6956	9.89E+01	9.89E+01	9.93E+01
494.1778	9.89E+01	9.89E+01	9.93E+01
494.6599	9.90E+01	9.89E+01	9.94E+01
495.142	9.91E+01	9.89E+01	9.94E+01
495.6241	9.93E+01	9.90E+01	9.95E+01
496.1063	9.94E+01	9.90E+01	9.95E+01
496.5884	9.94E+01	9.91E+01	9.96E+01
497.0705	9.95E+01	9.91E+01	9.96E+01
497.5526	9.95E+01	9.92E+01	9.97E+01
498.0348	9.96E+01	9.92E+01	9.97E+01
498.5169	9.95E+01	9.92E+01	9.97E+01
498.999	9.95E+01	9.92E+01	9.97E+01
499.4811	9.95E+01	9.92E+01	9.97E+01
499.9633	9.94E+01	9.91E+01	9.96E+01
500.4454	9.94E+01	9.91E+01	9.95E+01
500.9275	9.94E+01	9.91E+01	9.94E+01
501.4096	9.93E+01	9.90E+01	9.93E+01
501.8918	9.93E+01	9.90E+01	9.92E+01
502.3739	9.92E+01	9.89E+01	9.92E+01
502.856	9.92E+01	9.89E+01	9.91E+01
503.3381	9.92E+01	9.88E+01	9.91E+01
503.8203	9.91E+01	9.88E+01	9.91E+01
504.3024	9.91E+01	9.88E+01	9.91E+01
504.7845	9.91E+01	9.88E+01	9.92E+01
505.2666	9.91E+01	9.87E+01	9.92E+01
505.7487	9.90E+01	9.87E+01	9.93E+01
506.2309	9.90E+01	9.87E+01	9.93E+01
506.713	9.89E+01	9.87E+01	9.93E+01
507.1951	9.89E+01	9.87E+01	9.93E+01
507.6772	9.89E+01	9.86E+01	9.93E+01
508.1594	9.89E+01	9.86E+01	9.94E+01
508.6415	9.89E+01	9.87E+01	9.94E+01
509.1236	9.90E+01	9.87E+01	9.94E+01
509.6057	9.90E+01	9.87E+01	9.94E+01
510.0879	9.90E+01	9.86E+01	9.94E+01
510.57	9.90E+01	9.86E+01	9.93E+01
511.0521	9.90E+01	9.86E+01	9.93E+01
511.5342	9.89E+01	9.86E+01	9.92E+01
512.0164	9.89E+01	9.85E+01	9.92E+01

512.4985	9.89E+01	9.85E+01	9.91E+01
512.9806	9.89E+01	9.86E+01	9.91E+01
513.4628	9.90E+01	9.86E+01	9.90E+01
513.9449	9.90E+01	9.87E+01	9.91E+01
514.427	9.91E+01	9.87E+01	9.91E+01
514.9091	9.92E+01	9.88E+01	9.91E+01
515.3912	9.92E+01	9.89E+01	9.92E+01
515.8734	9.92E+01	9.89E+01	9.92E+01
516.3555	9.92E+01	9.88E+01	9.93E+01
516.8376	9.91E+01	9.88E+01	9.93E+01
517.3198	9.91E+01	9.88E+01	9.92E+01
517.8019	9.90E+01	9.87E+01	9.92E+01
518.284	9.89E+01	9.86E+01	9.92E+01
518.7661	9.89E+01	9.86E+01	9.92E+01
519.2482	9.89E+01	9.86E+01	9.92E+01
519.7303	9.89E+01	9.86E+01	9.91E+01
520.2125	9.89E+01	9.85E+01	9.91E+01
520.6946	9.89E+01	9.85E+01	9.91E+01
521.1768	9.89E+01	9.85E+01	9.90E+01
521.6589	9.89E+01	9.85E+01	9.90E+01
522.141	9.89E+01	9.85E+01	9.90E+01
522.6231	9.89E+01	9.85E+01	9.89E+01
523.1052	9.89E+01	9.85E+01	9.89E+01
523.5873	9.89E+01	9.85E+01	9.89E+01
524.0695	9.89E+01	9.85E+01	9.89E+01
524.5516	9.89E+01	9.84E+01	9.89E+01
525.0338	9.89E+01	9.84E+01	9.89E+01
525.5159	9.89E+01	9.84E+01	9.89E+01
525.998	9.89E+01	9.84E+01	9.89E+01
526.4801	9.89E+01	9.84E+01	9.89E+01
526.9622	9.89E+01	9.84E+01	9.89E+01
527.4443	9.89E+01	9.84E+01	9.89E+01
527.9265	9.89E+01	9.84E+01	9.89E+01
528.4086	9.89E+01	9.85E+01	9.89E+01
528.8907	9.89E+01	9.85E+01	9.90E+01
529.3729	9.89E+01	9.85E+01	9.90E+01
529.855	9.89E+01	9.85E+01	9.90E+01
530.3371	9.89E+01	9.85E+01	9.91E+01
530.8192	9.89E+01	9.85E+01	9.91E+01
531.3013	9.90E+01	9.85E+01	9.92E+01
531.7834	9.90E+01	9.86E+01	9.92E+01
532.2656	9.90E+01	9.86E+01	9.93E+01
532.7477	9.90E+01	9.86E+01	9.93E+01
533.2299	9.89E+01	9.86E+01	9.93E+01
533.712	9.89E+01	9.87E+01	9.93E+01
534.1941	9.89E+01	9.87E+01	9.93E+01
534.6762	9.89E+01	9.87E+01	9.93E+01

535.1583	9.88E+01	9.86E+01	9.92E+01
535.6404	9.88E+01	9.86E+01	9.92E+01
536.1226	9.88E+01	9.86E+01	9.92E+01
536.6047	9.88E+01	9.86E+01	9.91E+01
537.0869	9.89E+01	9.85E+01	9.91E+01
537.569	9.89E+01	9.85E+01	9.91E+01
538.0511	9.89E+01	9.86E+01	9.91E+01
538.5332	9.90E+01	9.86E+01	9.91E+01
539.0153	9.90E+01	9.86E+01	9.91E+01
539.4975	9.90E+01	9.86E+01	9.90E+01
539.9796	9.90E+01	9.86E+01	9.90E+01
540.4617	9.89E+01	9.85E+01	9.90E+01
540.9438	9.89E+01	9.85E+01	9.90E+01
541.426	9.89E+01	9.85E+01	9.90E+01
541.9081	9.88E+01	9.85E+01	9.90E+01
542.3902	9.88E+01	9.85E+01	9.90E+01
542.8723	9.88E+01	9.85E+01	9.90E+01
543.3545	9.88E+01	9.86E+01	9.91E+01
543.8366	9.88E+01	9.86E+01	9.91E+01
544.3187	9.88E+01	9.86E+01	9.91E+01
544.8008	9.89E+01	9.86E+01	9.92E+01
545.283	9.89E+01	9.86E+01	9.92E+01
545.7651	9.89E+01	9.86E+01	9.92E+01
546.2472	9.89E+01	9.86E+01	9.92E+01
546.7293	9.90E+01	9.86E+01	9.92E+01
547.2115	9.90E+01	9.86E+01	9.92E+01
547.6936	9.90E+01	9.87E+01	9.92E+01
548.1757	9.90E+01	9.87E+01	9.93E+01
548.6578	9.90E+01	9.88E+01	9.93E+01
549.14	9.90E+01	9.88E+01	9.94E+01
549.6221	9.90E+01	9.89E+01	9.94E+01
550.1042	9.90E+01	9.89E+01	9.94E+01
550.5864	9.89E+01	9.89E+01	9.94E+01
551.0685	9.89E+01	9.88E+01	9.94E+01
551.5506	9.89E+01	9.88E+01	9.94E+01
552.0327	9.89E+01	9.87E+01	9.93E+01
552.5148	9.89E+01	9.86E+01	9.93E+01
552.9969	9.90E+01	9.86E+01	9.93E+01
553.4791	9.90E+01	9.86E+01	9.94E+01
553.9612	9.90E+01	9.86E+01	9.94E+01
554.4434	9.90E+01	9.86E+01	9.94E+01
554.9255	9.90E+01	9.86E+01	9.94E+01
555.4076	9.91E+01	9.87E+01	9.94E+01
555.8897	9.91E+01	9.87E+01	9.94E+01
556.3718	9.91E+01	9.87E+01	9.94E+01
556.8539	9.91E+01	9.87E+01	9.94E+01
557.3361	9.91E+01	9.87E+01	9.94E+01

557.8182	9.91E+01	9.87E+01	9.94E+01
558.3004	9.91E+01	9.87E+01	9.93E+01
558.7825	9.90E+01	9.86E+01	9.93E+01
559.2646	9.91E+01	9.86E+01	9.93E+01
559.7467	9.91E+01	9.86E+01	9.92E+01
560.2288	9.91E+01	9.85E+01	9.92E+01
560.7109	9.91E+01	9.85E+01	9.92E+01
561.1931	9.91E+01	9.85E+01	9.92E+01
561.6752	9.92E+01	9.85E+01	9.91E+01
562.1573	9.92E+01	9.86E+01	9.91E+01
562.6395	9.91E+01	9.86E+01	9.91E+01
563.1216	9.91E+01	9.86E+01	9.91E+01
563.6037	9.91E+01	9.86E+01	9.91E+01
564.0858	9.90E+01	9.86E+01	9.90E+01
564.5679	9.90E+01	9.86E+01	9.90E+01
565.05	9.90E+01	9.85E+01	9.89E+01
565.5322	9.90E+01	9.85E+01	9.89E+01
566.0143	9.90E+01	9.85E+01	9.89E+01
566.4965	9.90E+01	9.85E+01	9.89E+01
566.9786	9.90E+01	9.85E+01	9.89E+01
567.4607	9.90E+01	9.85E+01	9.89E+01
567.9428	9.91E+01	9.85E+01	9.89E+01
568.4249	9.91E+01	9.86E+01	9.90E+01
568.907	9.91E+01	9.86E+01	9.90E+01
569.3892	9.91E+01	9.87E+01	9.91E+01
569.8713	9.92E+01	9.87E+01	9.91E+01
570.3535	9.92E+01	9.87E+01	9.92E+01
570.8356	9.92E+01	9.88E+01	9.92E+01
571.3177	9.92E+01	9.88E+01	9.93E+01
571.7998	9.93E+01	9.89E+01	9.93E+01
572.2819	9.93E+01	9.89E+01	9.93E+01
572.764	9.93E+01	9.89E+01	9.93E+01
573.2462	9.93E+01	9.89E+01	9.93E+01
573.7283	9.92E+01	9.89E+01	9.93E+01
574.2104	9.92E+01	9.89E+01	9.93E+01
574.6926	9.92E+01	9.89E+01	9.93E+01
575.1747	9.91E+01	9.88E+01	9.93E+01
575.6568	9.90E+01	9.88E+01	9.93E+01
576.1389	9.90E+01	9.88E+01	9.92E+01
576.621	9.90E+01	9.88E+01	9.92E+01
577.1032	9.90E+01	9.88E+01	9.92E+01
577.5853	9.90E+01	9.88E+01	9.92E+01
578.0674	9.90E+01	9.88E+01	9.92E+01
578.5496	9.90E+01	9.88E+01	9.92E+01
579.0317	9.90E+01	9.88E+01	9.92E+01
579.5138	9.91E+01	9.88E+01	9.92E+01
579.9959	9.91E+01	9.89E+01	9.92E+01

580.4781	9.92E+01	9.89E+01	9.93E+01
580.9602	9.92E+01	9.90E+01	9.93E+01
581.4423	9.92E+01	9.90E+01	9.93E+01
581.9244	9.92E+01	9.91E+01	9.93E+01
582.4066	9.92E+01	9.91E+01	9.93E+01
582.8887	9.92E+01	9.91E+01	9.93E+01
583.3708	9.92E+01	9.90E+01	9.93E+01
583.8529	9.92E+01	9.90E+01	9.93E+01
584.3351	9.92E+01	9.90E+01	9.93E+01
584.8172	9.92E+01	9.90E+01	9.93E+01
585.2993	9.93E+01	9.89E+01	9.94E+01
585.7814	9.93E+01	9.89E+01	9.94E+01
586.2635	9.94E+01	9.89E+01	9.94E+01
586.7457	9.94E+01	9.89E+01	9.94E+01
587.2278	9.94E+01	9.89E+01	9.94E+01
587.7099	9.95E+01	9.90E+01	9.94E+01
588.1921	9.95E+01	9.90E+01	9.94E+01
588.6742	9.95E+01	9.90E+01	9.94E+01
589.1563	9.94E+01	9.90E+01	9.94E+01
589.6384	9.94E+01	9.90E+01	9.94E+01
590.1205	9.94E+01	9.90E+01	9.93E+01
590.6027	9.93E+01	9.89E+01	9.93E+01
591.0848	9.93E+01	9.89E+01	9.93E+01
591.5669	9.93E+01	9.89E+01	9.93E+01
592.0491	9.92E+01	9.90E+01	9.93E+01
592.5312	9.92E+01	9.90E+01	9.93E+01
593.0133	9.92E+01	9.90E+01	9.93E+01
593.4954	9.92E+01	9.90E+01	9.94E+01
593.9775	9.92E+01	9.90E+01	9.94E+01
594.4597	9.92E+01	9.90E+01	9.94E+01
594.9418	9.93E+01	9.90E+01	9.94E+01
595.424	9.93E+01	9.90E+01	9.94E+01
595.9061	9.93E+01	9.90E+01	9.94E+01
596.3882	9.92E+01	9.89E+01	9.94E+01
596.8703	9.92E+01	9.89E+01	9.94E+01
597.3524	9.92E+01	9.89E+01	9.94E+01
597.8345	9.91E+01	9.89E+01	9.94E+01
598.3167	9.91E+01	9.88E+01	9.94E+01
598.7988	9.91E+01	9.88E+01	9.93E+01
599.2809	9.91E+01	9.88E+01	9.93E+01
599.7631	9.91E+01	9.88E+01	9.93E+01
600.2452	9.91E+01	9.88E+01	9.92E+01
600.7273	9.91E+01	9.88E+01	9.92E+01
601.2094	9.92E+01	9.89E+01	9.92E+01
601.6915	9.92E+01	9.89E+01	9.92E+01
602.1736	9.92E+01	9.89E+01	9.92E+01
602.6558	9.92E+01	9.89E+01	9.92E+01

603.1379	9.92E+01	9.89E+01	9.93E+01
603.6201	9.92E+01	9.89E+01	9.93E+01
604.1022	9.93E+01	9.89E+01	9.93E+01
604.5843	9.93E+01	9.89E+01	9.93E+01
605.0664	9.93E+01	9.89E+01	9.93E+01
605.5485	9.94E+01	9.89E+01	9.94E+01
606.0306	9.94E+01	9.89E+01	9.94E+01
606.5128	9.94E+01	9.89E+01	9.94E+01
606.9949	9.94E+01	9.89E+01	9.94E+01
607.4771	9.94E+01	9.89E+01	9.94E+01
607.9592	9.94E+01	9.89E+01	9.94E+01
608.4413	9.93E+01	9.89E+01	9.94E+01
608.9234	9.93E+01	9.89E+01	9.94E+01
609.4055	9.93E+01	9.89E+01	9.94E+01
609.8876	9.93E+01	9.89E+01	9.94E+01
610.3698	9.93E+01	9.89E+01	9.94E+01
610.8519	9.93E+01	9.89E+01	9.94E+01
611.334	9.93E+01	9.90E+01	9.94E+01
611.8162	9.93E+01	9.90E+01	9.94E+01
612.2983	9.93E+01	9.90E+01	9.94E+01
612.7804	9.92E+01	9.89E+01	9.94E+01
613.2625	9.92E+01	9.89E+01	9.94E+01
613.7446	9.92E+01	9.89E+01	9.94E+01
614.2268	9.92E+01	9.88E+01	9.94E+01
614.7089	9.92E+01	9.88E+01	9.94E+01
615.191	9.92E+01	9.88E+01	9.94E+01
615.6732	9.92E+01	9.88E+01	9.94E+01
616.1553	9.92E+01	9.88E+01	9.94E+01
616.6374	9.92E+01	9.88E+01	9.94E+01
617.1195	9.92E+01	9.88E+01	9.94E+01
617.6016	9.92E+01	9.88E+01	9.94E+01
618.0838	9.91E+01	9.89E+01	9.94E+01
618.5659	9.91E+01	9.89E+01	9.94E+01
619.048	9.91E+01	9.90E+01	9.95E+01
619.5302	9.91E+01	9.90E+01	9.95E+01
620.0123	9.91E+01	9.90E+01	9.94E+01
620.4944	9.92E+01	9.90E+01	9.94E+01
620.9765	9.92E+01	9.90E+01	9.94E+01
621.4587	9.92E+01	9.90E+01	9.94E+01
621.9408	9.92E+01	9.90E+01	9.94E+01
622.4229	9.92E+01	9.90E+01	9.94E+01
622.905	9.92E+01	9.90E+01	9.94E+01
623.3871	9.92E+01	9.90E+01	9.94E+01
623.8693	9.92E+01	9.90E+01	9.94E+01
624.3514	9.92E+01	9.90E+01	9.94E+01
624.8335	9.92E+01	9.89E+01	9.94E+01
625.3157	9.92E+01	9.89E+01	9.94E+01

625.7978	9.92E+01	9.89E+01	9.94E+01
626.2799	9.92E+01	9.89E+01	9.94E+01
626.762	9.92E+01	9.89E+01	9.94E+01
627.2441	9.92E+01	9.89E+01	9.94E+01
627.7263	9.92E+01	9.89E+01	9.94E+01
628.2084	9.92E+01	9.89E+01	9.94E+01
628.6905	9.92E+01	9.89E+01	9.94E+01
629.1727	9.92E+01	9.89E+01	9.94E+01
629.6548	9.92E+01	9.89E+01	9.94E+01
630.1369	9.92E+01	9.89E+01	9.94E+01
630.619	9.92E+01	9.89E+01	9.94E+01
631.1011	9.92E+01	9.89E+01	9.94E+01
631.5833	9.93E+01	9.89E+01	9.94E+01
632.0654	9.93E+01	9.89E+01	9.94E+01
632.5475	9.93E+01	9.89E+01	9.93E+01
633.0297	9.93E+01	9.89E+01	9.93E+01
633.5118	9.93E+01	9.89E+01	9.93E+01
633.9939	9.93E+01	9.90E+01	9.94E+01
634.476	9.93E+01	9.90E+01	9.94E+01
634.9581	9.93E+01	9.90E+01	9.94E+01
635.4402	9.93E+01	9.90E+01	9.94E+01
635.9224	9.93E+01	9.90E+01	9.94E+01
636.4045	9.92E+01	9.90E+01	9.93E+01
636.8867	9.92E+01	9.90E+01	9.93E+01
637.3688	9.92E+01	9.90E+01	9.93E+01
637.8509	9.92E+01	9.90E+01	9.93E+01
638.333	9.92E+01	9.90E+01	9.92E+01
638.8151	9.92E+01	9.90E+01	9.92E+01
639.2972	9.92E+01	9.90E+01	9.92E+01
639.7794	9.92E+01	9.90E+01	9.93E+01
640.2615	9.92E+01	9.89E+01	9.93E+01
640.7437	9.92E+01	9.89E+01	9.93E+01
641.2258	9.92E+01	9.89E+01	9.93E+01
641.7079	9.92E+01	9.89E+01	9.93E+01
642.19	9.92E+01	9.89E+01	9.93E+01
642.6721	9.92E+01	9.89E+01	9.93E+01
643.1542	9.92E+01	9.90E+01	9.93E+01
643.6364	9.92E+01	9.90E+01	9.93E+01
644.1185	9.92E+01	9.90E+01	9.93E+01
644.6006	9.92E+01	9.90E+01	9.93E+01
645.0828	9.92E+01	9.91E+01	9.93E+01
645.5649	9.92E+01	9.91E+01	9.93E+01
646.047	9.92E+01	9.91E+01	9.93E+01
646.5291	9.92E+01	9.90E+01	9.93E+01
647.0112	9.92E+01	9.90E+01	9.93E+01
647.4934	9.92E+01	9.90E+01	9.93E+01
647.9755	9.92E+01	9.90E+01	9.93E+01

648.4576	9.92E+01	9.90E+01	9.94E+01
648.9398	9.92E+01	9.89E+01	9.94E+01
649.4219	9.92E+01	9.89E+01	9.94E+01
649.904	9.92E+01	9.89E+01	9.94E+01
650.3861	9.93E+01	9.90E+01	9.94E+01
650.8682	9.93E+01	9.90E+01	9.94E+01
651.3504	9.93E+01	9.90E+01	9.94E+01
651.8325	9.93E+01	9.90E+01	9.94E+01
652.3146	9.93E+01	9.90E+01	9.94E+01
652.7968	9.93E+01	9.90E+01	9.94E+01
653.2789	9.93E+01	9.90E+01	9.94E+01
653.761	9.93E+01	9.90E+01	9.94E+01
654.2431	9.93E+01	9.90E+01	9.94E+01
654.7252	9.92E+01	9.90E+01	9.94E+01
655.2074	9.92E+01	9.90E+01	9.94E+01
655.6895	9.92E+01	9.90E+01	9.94E+01
656.1716	9.92E+01	9.90E+01	9.94E+01
656.6537	9.92E+01	9.89E+01	9.94E+01
657.1359	9.92E+01	9.89E+01	9.94E+01
657.618	9.92E+01	9.89E+01	9.94E+01
658.1001	9.91E+01	9.89E+01	9.94E+01
658.5822	9.91E+01	9.89E+01	9.94E+01
659.0644	9.91E+01	9.89E+01	9.93E+01
659.5465	9.91E+01	9.89E+01	9.93E+01
660.0286	9.91E+01	9.89E+01	9.93E+01
660.5107	9.91E+01	9.89E+01	9.93E+01
660.9929	9.91E+01	9.89E+01	9.93E+01
661.475	9.91E+01	9.89E+01	9.93E+01
661.9571	9.91E+01	9.89E+01	9.93E+01
662.4393	9.91E+01	9.89E+01	9.93E+01
662.9214	9.91E+01	9.89E+01	9.93E+01
663.4035	9.91E+01	9.89E+01	9.94E+01
663.8856	9.91E+01	9.88E+01	9.94E+01
664.3677	9.92E+01	9.88E+01	9.94E+01
664.8499	9.92E+01	9.88E+01	9.94E+01
665.332	9.92E+01	9.88E+01	9.94E+01
665.8141	9.92E+01	9.88E+01	9.94E+01
666.2963	9.92E+01	9.88E+01	9.94E+01
666.7784	9.92E+01	9.88E+01	9.94E+01
667.2605	9.92E+01	9.88E+01	9.94E+01
667.7426	9.92E+01	9.88E+01	9.93E+01
668.2247	9.92E+01	9.89E+01	9.93E+01
668.7068	9.92E+01	9.89E+01	9.93E+01
669.189	9.92E+01	9.89E+01	9.94E+01
669.6711	9.92E+01	9.89E+01	9.94E+01
670.1533	9.93E+01	9.88E+01	9.94E+01
670.6354	9.93E+01	9.88E+01	9.94E+01

671.1175	9.93E+01	9.88E+01	9.94E+01
671.5996	9.93E+01	9.88E+01	9.94E+01
672.0817	9.93E+01	9.88E+01	9.94E+01
672.5638	9.93E+01	9.88E+01	9.94E+01
673.046	9.92E+01	9.88E+01	9.94E+01
673.5281	9.92E+01	9.88E+01	9.93E+01
674.0103	9.92E+01	9.89E+01	9.93E+01
674.4924	9.92E+01	9.89E+01	9.93E+01
674.9745	9.92E+01	9.89E+01	9.93E+01
675.4566	9.91E+01	9.89E+01	9.94E+01
675.9387	9.91E+01	9.89E+01	9.94E+01
676.4208	9.91E+01	9.90E+01	9.94E+01
676.903	9.92E+01	9.90E+01	9.94E+01
677.3851	9.92E+01	9.90E+01	9.94E+01
677.8672	9.92E+01	9.90E+01	9.95E+01
678.3494	9.92E+01	9.90E+01	9.95E+01
678.8315	9.92E+01	9.90E+01	9.94E+01
679.3136	9.92E+01	9.90E+01	9.94E+01
679.7957	9.92E+01	9.90E+01	9.94E+01
680.2778	9.92E+01	9.90E+01	9.94E+01
680.7599	9.92E+01	9.90E+01	9.94E+01
681.2421	9.92E+01	9.90E+01	9.93E+01
681.7242	9.92E+01	9.90E+01	9.93E+01
682.2064	9.92E+01	9.90E+01	9.93E+01
682.6885	9.92E+01	9.90E+01	9.93E+01
683.1706	9.91E+01	9.89E+01	9.93E+01
683.6527	9.91E+01	9.89E+01	9.93E+01
684.1348	9.91E+01	9.89E+01	9.93E+01
684.6169	9.91E+01	9.89E+01	9.93E+01
685.0991	9.91E+01	9.89E+01	9.93E+01
685.5812	9.91E+01	9.89E+01	9.93E+01
686.0634	9.91E+01	9.90E+01	9.93E+01
686.5455	9.92E+01	9.90E+01	9.93E+01
687.0276	9.92E+01	9.91E+01	9.93E+01
687.5097	9.93E+01	9.91E+01	9.94E+01
687.9918	9.93E+01	9.91E+01	9.94E+01
688.474	9.93E+01	9.91E+01	9.94E+01
688.9561	9.93E+01	9.91E+01	9.94E+01
689.4382	9.92E+01	9.91E+01	9.94E+01
689.9203	9.92E+01	9.90E+01	9.94E+01
690.4025	9.92E+01	9.90E+01	9.93E+01
690.8846	9.91E+01	9.90E+01	9.93E+01
691.3667	9.91E+01	9.89E+01	9.93E+01
691.8488	9.91E+01	9.89E+01	9.93E+01
692.331	9.91E+01	9.89E+01	9.93E+01
692.8131	9.91E+01	9.90E+01	9.93E+01
693.2952	9.91E+01	9.90E+01	9.93E+01

693.7773	9.91E+01	9.90E+01	9.93E+01
694.2595	9.91E+01	9.90E+01	9.93E+01
694.7416	9.91E+01	9.90E+01	9.93E+01
695.2237	9.91E+01	9.90E+01	9.93E+01
695.7058	9.91E+01	9.89E+01	9.94E+01
696.188	9.91E+01	9.89E+01	9.94E+01
696.6701	9.91E+01	9.89E+01	9.94E+01
697.1522	9.91E+01	9.89E+01	9.94E+01
697.6343	9.91E+01	9.89E+01	9.94E+01
698.1165	9.91E+01	9.89E+01	9.94E+01
698.5986	9.91E+01	9.89E+01	9.94E+01
699.0807	9.91E+01	9.90E+01	9.94E+01
699.5628	9.91E+01	9.90E+01	9.94E+01
700.045	9.91E+01	9.90E+01	9.94E+01
700.5271	9.91E+01	9.90E+01	9.94E+01
701.0092	9.91E+01	9.90E+01	9.93E+01
701.4913	9.91E+01	9.90E+01	9.93E+01
701.9734	9.91E+01	9.89E+01	9.93E+01
702.4556	9.91E+01	9.89E+01	9.93E+01
702.9377	9.91E+01	9.89E+01	9.92E+01
703.4199	9.91E+01	9.89E+01	9.92E+01
703.902	9.91E+01	9.89E+01	9.92E+01
704.3841	9.91E+01	9.89E+01	9.92E+01
704.8662	9.91E+01	9.89E+01	9.93E+01
705.3483	9.91E+01	9.89E+01	9.93E+01
705.8304	9.91E+01	9.90E+01	9.93E+01
706.3126	9.92E+01	9.90E+01	9.93E+01
706.7947	9.92E+01	9.90E+01	9.93E+01
707.2769	9.92E+01	9.90E+01	9.94E+01
707.759	9.92E+01	9.90E+01	9.93E+01
708.2411	9.93E+01	9.90E+01	9.93E+01
708.7232	9.93E+01	9.90E+01	9.93E+01
709.2053	9.93E+01	9.90E+01	9.93E+01
709.6874	9.93E+01	9.90E+01	9.93E+01
710.1696	9.93E+01	9.90E+01	9.93E+01
710.6517	9.93E+01	9.90E+01	9.93E+01
711.1339	9.93E+01	9.90E+01	9.94E+01
711.616	9.92E+01	9.90E+01	9.94E+01
712.0981	9.92E+01	9.90E+01	9.94E+01
712.5802	9.92E+01	9.90E+01	9.94E+01
713.0623	9.92E+01	9.90E+01	9.94E+01
713.5444	9.92E+01	9.90E+01	9.94E+01
714.0266	9.92E+01	9.90E+01	9.94E+01
714.5087	9.92E+01	9.90E+01	9.94E+01
714.9908	9.92E+01	9.90E+01	9.94E+01
715.473	9.92E+01	9.90E+01	9.94E+01
715.9551	9.92E+01	9.90E+01	9.94E+01

716.4372	9.92E+01	9.90E+01	9.93E+01
716.9193	9.92E+01	9.91E+01	9.93E+01
717.4014	9.92E+01	9.91E+01	9.93E+01
717.8835	9.92E+01	9.90E+01	9.93E+01
718.3657	9.92E+01	9.90E+01	9.93E+01
718.8478	9.92E+01	9.90E+01	9.93E+01
719.33	9.92E+01	9.90E+01	9.93E+01
719.8121	9.92E+01	9.89E+01	9.93E+01
720.2942	9.92E+01	9.89E+01	9.93E+01
720.7763	9.92E+01	9.89E+01	9.93E+01
721.2584	9.91E+01	9.89E+01	9.93E+01
721.7405	9.91E+01	9.89E+01	9.93E+01
722.2227	9.91E+01	9.88E+01	9.93E+01
722.7048	9.91E+01	9.88E+01	9.93E+01
723.187	9.91E+01	9.88E+01	9.93E+01
723.6691	9.91E+01	9.89E+01	9.93E+01
724.1512	9.91E+01	9.89E+01	9.93E+01
724.6333	9.91E+01	9.89E+01	9.93E+01
725.1154	9.91E+01	9.89E+01	9.93E+01
725.5975	9.91E+01	9.90E+01	9.94E+01
726.0797	9.91E+01	9.90E+01	9.94E+01
726.5618	9.91E+01	9.90E+01	9.94E+01
727.0439	9.91E+01	9.90E+01	9.94E+01
727.5261	9.91E+01	9.90E+01	9.94E+01
728.0082	9.90E+01	9.90E+01	9.94E+01
728.4903	9.90E+01	9.90E+01	9.94E+01
728.9724	9.90E+01	9.90E+01	9.93E+01
729.4546	9.90E+01	9.89E+01	9.93E+01
729.9367	9.90E+01	9.89E+01	9.93E+01
730.4188	9.90E+01	9.90E+01	9.93E+01
730.9009	9.90E+01	9.90E+01	9.93E+01
731.3831	9.91E+01	9.90E+01	9.93E+01
731.8652	9.91E+01	9.90E+01	9.93E+01
732.3473	9.91E+01	9.90E+01	9.93E+01
732.8294	9.91E+01	9.90E+01	9.93E+01
733.3116	9.91E+01	9.90E+01	9.93E+01
733.7937	9.91E+01	9.90E+01	9.93E+01
734.2758	9.90E+01	9.90E+01	9.93E+01
734.7579	9.90E+01	9.90E+01	9.92E+01
735.2401	9.90E+01	9.90E+01	9.92E+01
735.7222	9.90E+01	9.90E+01	9.92E+01
736.2043	9.90E+01	9.90E+01	9.92E+01
736.6864	9.90E+01	9.90E+01	9.92E+01
737.1686	9.90E+01	9.90E+01	9.92E+01
737.6507	9.90E+01	9.90E+01	9.92E+01
738.1328	9.90E+01	9.90E+01	9.92E+01
738.6149	9.91E+01	9.90E+01	9.92E+01

739.097	9.91E+01	9.90E+01	9.92E+01
739.5792	9.91E+01	9.90E+01	9.92E+01
740.0613	9.90E+01	9.90E+01	9.92E+01
740.5434	9.90E+01	9.90E+01	9.92E+01
741.0256	9.90E+01	9.90E+01	9.92E+01
741.5077	9.90E+01	9.90E+01	9.92E+01
741.9898	9.90E+01	9.90E+01	9.92E+01
742.4719	9.90E+01	9.90E+01	9.92E+01
742.954	9.90E+01	9.90E+01	9.92E+01
743.4362	9.90E+01	9.90E+01	9.92E+01
743.9183	9.90E+01	9.90E+01	9.92E+01
744.4005	9.90E+01	9.89E+01	9.92E+01
744.8826	9.90E+01	9.89E+01	9.93E+01
745.3647	9.90E+01	9.89E+01	9.93E+01
745.8468	9.90E+01	9.89E+01	9.93E+01
746.3289	9.89E+01	9.89E+01	9.93E+01
746.811	9.89E+01	9.90E+01	9.93E+01
747.2932	9.89E+01	9.90E+01	9.93E+01
747.7753	9.89E+01	9.90E+01	9.93E+01
748.2574	9.89E+01	9.90E+01	9.93E+01
748.7396	9.89E+01	9.89E+01	9.93E+01
749.2217	9.89E+01	9.89E+01	9.93E+01
749.7038	9.89E+01	9.89E+01	9.93E+01
750.1859	9.89E+01	9.89E+01	9.93E+01
750.668	9.89E+01	9.89E+01	9.93E+01
751.1501	9.89E+01	9.89E+01	9.93E+01
751.6323	9.89E+01	9.89E+01	9.93E+01
752.1144	9.89E+01	9.89E+01	9.93E+01
752.5966	9.89E+01	9.89E+01	9.93E+01
753.0787	9.89E+01	9.89E+01	9.93E+01
753.5608	9.89E+01	9.89E+01	9.93E+01
754.0429	9.89E+01	9.89E+01	9.93E+01
754.525	9.89E+01	9.89E+01	9.93E+01
755.0071	9.88E+01	9.89E+01	9.92E+01
755.4893	9.88E+01	9.89E+01	9.93E+01
755.9714	9.88E+01	9.89E+01	9.93E+01
756.4536	9.88E+01	9.89E+01	9.93E+01
756.9357	9.88E+01	9.89E+01	9.93E+01
757.4178	9.88E+01	9.89E+01	9.93E+01
757.8999	9.89E+01	9.89E+01	9.93E+01
758.382	9.89E+01	9.89E+01	9.93E+01
758.8641	9.89E+01	9.89E+01	9.93E+01
759.3463	9.90E+01	9.89E+01	9.93E+01
759.8284	9.90E+01	9.89E+01	9.93E+01
760.3105	9.91E+01	9.89E+01	9.94E+01
760.7927	9.91E+01	9.89E+01	9.94E+01
761.2748	9.91E+01	9.89E+01	9.94E+01

761.7569	9.90E+01	9.89E+01	9.94E+01
762.239	9.90E+01	9.89E+01	9.93E+01
762.7211	9.90E+01	9.89E+01	9.93E+01
763.2033	9.90E+01	9.89E+01	9.93E+01
763.6854	9.89E+01	9.89E+01	9.92E+01
764.1675	9.89E+01	9.89E+01	9.92E+01
764.6497	9.89E+01	9.89E+01	9.91E+01
765.1318	9.89E+01	9.89E+01	9.91E+01
765.6139	9.89E+01	9.89E+01	9.91E+01
766.096	9.88E+01	9.89E+01	9.91E+01
766.5781	9.88E+01	9.89E+01	9.91E+01
767.0603	9.89E+01	9.89E+01	9.91E+01
767.5424	9.89E+01	9.89E+01	9.91E+01
768.0245	9.89E+01	9.89E+01	9.91E+01
768.5067	9.89E+01	9.89E+01	9.92E+01
768.9888	9.89E+01	9.88E+01	9.92E+01
769.4709	9.89E+01	9.88E+01	9.92E+01
769.953	9.89E+01	9.88E+01	9.92E+01
770.4352	9.89E+01	9.88E+01	9.92E+01
770.9173	9.89E+01	9.88E+01	9.92E+01
771.3994	9.89E+01	9.88E+01	9.92E+01
771.8815	9.89E+01	9.88E+01	9.91E+01
772.3636	9.89E+01	9.88E+01	9.91E+01
772.8458	9.89E+01	9.88E+01	9.91E+01
773.3279	9.89E+01	9.88E+01	9.91E+01
773.81	9.89E+01	9.88E+01	9.91E+01
774.2922	9.89E+01	9.89E+01	9.91E+01
774.7743	9.89E+01	9.89E+01	9.92E+01
775.2564	9.89E+01	9.89E+01	9.92E+01
775.7385	9.90E+01	9.89E+01	9.92E+01
776.2206	9.90E+01	9.90E+01	9.92E+01
776.7028	9.90E+01	9.90E+01	9.92E+01
777.1849	9.90E+01	9.90E+01	9.92E+01
777.667	9.90E+01	9.90E+01	9.92E+01
778.1492	9.90E+01	9.90E+01	9.92E+01
778.6313	9.90E+01	9.89E+01	9.92E+01
779.1134	9.90E+01	9.89E+01	9.92E+01
779.5955	9.90E+01	9.89E+01	9.92E+01
780.0776	9.90E+01	9.89E+01	9.92E+01
780.5598	9.90E+01	9.89E+01	9.92E+01
781.0419	9.90E+01	9.89E+01	9.92E+01
781.524	9.90E+01	9.89E+01	9.92E+01
782.0062	9.89E+01	9.89E+01	9.92E+01
782.4883	9.89E+01	9.89E+01	9.92E+01
782.9704	9.89E+01	9.89E+01	9.92E+01
783.4525	9.89E+01	9.89E+01	9.92E+01
783.9346	9.88E+01	9.89E+01	9.92E+01

784.4167	9.88E+01	9.89E+01	9.92E+01
784.8989	9.88E+01	9.89E+01	9.92E+01
785.381	9.88E+01	9.88E+01	9.91E+01
785.8632	9.88E+01	9.88E+01	9.91E+01
786.3453	9.88E+01	9.88E+01	9.91E+01
786.8274	9.88E+01	9.88E+01	9.92E+01
787.3095	9.89E+01	9.88E+01	9.92E+01
787.7916	9.89E+01	9.89E+01	9.92E+01
788.2737	9.89E+01	9.89E+01	9.92E+01
788.7559	9.89E+01	9.89E+01	9.93E+01
789.238	9.89E+01	9.89E+01	9.93E+01
789.7202	9.89E+01	9.89E+01	9.93E+01
790.2023	9.89E+01	9.90E+01	9.93E+01
790.6844	9.89E+01	9.90E+01	9.92E+01
791.1665	9.89E+01	9.90E+01	9.92E+01
791.6486	9.89E+01	9.90E+01	9.92E+01
792.1307	9.90E+01	9.90E+01	9.92E+01
792.6129	9.90E+01	9.90E+01	9.92E+01
793.095	9.90E+01	9.90E+01	9.92E+01
793.5771	9.90E+01	9.90E+01	9.92E+01
794.0593	9.90E+01	9.90E+01	9.92E+01
794.5414	9.90E+01	9.89E+01	9.92E+01
795.0235	9.90E+01	9.89E+01	9.92E+01
795.5056	9.89E+01	9.89E+01	9.91E+01
795.9877	9.89E+01	9.89E+01	9.91E+01
796.4698	9.89E+01	9.89E+01	9.91E+01
796.952	9.89E+01	9.89E+01	9.92E+01
797.4341	9.89E+01	9.89E+01	9.92E+01
797.9163	9.89E+01	9.89E+01	9.92E+01
798.3984	9.89E+01	9.89E+01	9.92E+01
798.8805	9.89E+01	9.89E+01	9.92E+01
799.3626	9.89E+01	9.90E+01	9.92E+01
799.8447	9.90E+01	9.90E+01	9.93E+01
800.3269	9.90E+01	9.90E+01	9.93E+01
800.809	9.90E+01	9.90E+01	9.93E+01
801.2911	9.90E+01	9.90E+01	9.93E+01
801.7733	9.90E+01	9.90E+01	9.93E+01
802.2554	9.90E+01	9.89E+01	9.93E+01
802.7375	9.90E+01	9.89E+01	9.93E+01
803.2196	9.90E+01	9.89E+01	9.93E+01
803.7017	9.89E+01	9.89E+01	9.93E+01
804.1839	9.89E+01	9.89E+01	9.93E+01
804.666	9.89E+01	9.89E+01	9.93E+01
805.1481	9.89E+01	9.89E+01	9.93E+01
805.6302	9.89E+01	9.89E+01	9.93E+01
806.1124	9.89E+01	9.89E+01	9.92E+01
806.5945	9.88E+01	9.89E+01	9.92E+01

807.0766	9.89E+01	9.89E+01	9.92E+01
807.5587	9.89E+01	9.89E+01	9.92E+01
808.0409	9.89E+01	9.89E+01	9.92E+01
808.523	9.89E+01	9.89E+01	9.93E+01
809.0051	9.89E+01	9.89E+01	9.93E+01
809.4872	9.89E+01	9.89E+01	9.93E+01
809.9694	9.90E+01	9.90E+01	9.93E+01
810.4515	9.90E+01	9.90E+01	9.93E+01
810.9336	9.90E+01	9.90E+01	9.93E+01
811.4158	9.90E+01	9.90E+01	9.93E+01
811.8979	9.90E+01	9.90E+01	9.93E+01
812.38	9.90E+01	9.91E+01	9.93E+01
812.8621	9.91E+01	9.91E+01	9.93E+01
813.3442	9.91E+01	9.91E+01	9.93E+01
813.8264	9.91E+01	9.91E+01	9.93E+01
814.3085	9.91E+01	9.91E+01	9.93E+01
814.7906	9.91E+01	9.90E+01	9.93E+01
815.2728	9.90E+01	9.90E+01	9.93E+01
815.7549	9.90E+01	9.90E+01	9.93E+01
816.237	9.90E+01	9.90E+01	9.93E+01
816.7191	9.90E+01	9.90E+01	9.93E+01
817.2012	9.90E+01	9.90E+01	9.93E+01
817.6833	9.90E+01	9.90E+01	9.93E+01
818.1655	9.90E+01	9.90E+01	9.93E+01
818.6476	9.90E+01	9.90E+01	9.93E+01
819.1298	9.90E+01	9.90E+01	9.93E+01
819.6119	9.90E+01	9.90E+01	9.93E+01
820.094	9.90E+01	9.90E+01	9.93E+01
820.5761	9.90E+01	9.90E+01	9.93E+01
821.0582	9.90E+01	9.89E+01	9.93E+01
821.5403	9.89E+01	9.89E+01	9.93E+01
822.0225	9.89E+01	9.89E+01	9.92E+01
822.5046	9.89E+01	9.89E+01	9.92E+01
822.9868	9.88E+01	9.89E+01	9.92E+01
823.4689	9.88E+01	9.89E+01	9.92E+01
823.951	9.88E+01	9.90E+01	9.92E+01
824.4331	9.88E+01	9.90E+01	9.92E+01
824.9152	9.88E+01	9.90E+01	9.92E+01
825.3973	9.88E+01	9.90E+01	9.92E+01
825.8795	9.88E+01	9.90E+01	9.92E+01
826.3616	9.88E+01	9.90E+01	9.92E+01
826.8438	9.88E+01	9.89E+01	9.92E+01
827.3259	9.89E+01	9.89E+01	9.92E+01
827.808	9.89E+01	9.89E+01	9.93E+01
828.2901	9.89E+01	9.89E+01	9.93E+01
828.7722	9.89E+01	9.89E+01	9.93E+01
829.2543	9.89E+01	9.89E+01	9.93E+01

829.7365	9.89E+01	9.89E+01	9.93E+01
830.2186	9.89E+01	9.89E+01	9.93E+01
830.7007	9.89E+01	9.89E+01	9.93E+01
831.1829	9.89E+01	9.89E+01	9.93E+01
831.665	9.89E+01	9.89E+01	9.93E+01
832.1471	9.88E+01	9.89E+01	9.92E+01
832.6292	9.88E+01	9.88E+01	9.92E+01
833.1113	9.88E+01	9.88E+01	9.92E+01
833.5934	9.88E+01	9.88E+01	9.92E+01
834.0756	9.88E+01	9.88E+01	9.92E+01
834.5577	9.88E+01	9.88E+01	9.92E+01
835.0399	9.88E+01	9.88E+01	9.92E+01
835.522	9.89E+01	9.88E+01	9.92E+01
836.0041	9.89E+01	9.88E+01	9.92E+01
836.4862	9.89E+01	9.88E+01	9.92E+01
836.9683	9.89E+01	9.88E+01	9.92E+01
837.4504	9.89E+01	9.88E+01	9.92E+01
837.9326	9.89E+01	9.88E+01	9.92E+01
838.4147	9.89E+01	9.88E+01	9.92E+01
838.8969	9.89E+01	9.88E+01	9.92E+01
839.379	9.89E+01	9.89E+01	9.92E+01
839.8611	9.89E+01	9.89E+01	9.92E+01
840.3432	9.89E+01	9.89E+01	9.92E+01
840.8253	9.89E+01	9.90E+01	9.93E+01
841.3075	9.89E+01	9.90E+01	9.93E+01
841.7896	9.90E+01	9.90E+01	9.93E+01
842.2717	9.90E+01	9.90E+01	9.93E+01
842.7538	9.90E+01	9.90E+01	9.93E+01
843.236	9.90E+01	9.90E+01	9.93E+01
843.7181	9.89E+01	9.89E+01	9.92E+01
844.2002	9.89E+01	9.89E+01	9.92E+01
844.6823	9.89E+01	9.89E+01	9.92E+01
845.1645	9.89E+01	9.89E+01	9.92E+01
845.6466	9.89E+01	9.89E+01	9.92E+01
846.1287	9.89E+01	9.89E+01	9.93E+01
846.6108	9.90E+01	9.89E+01	9.93E+01
847.093	9.90E+01	9.89E+01	9.93E+01
847.5751	9.90E+01	9.89E+01	9.93E+01
848.0572	9.90E+01	9.89E+01	9.93E+01
848.5393	9.90E+01	9.89E+01	9.93E+01
849.0215	9.90E+01	9.89E+01	9.93E+01
849.5036	9.90E+01	9.89E+01	9.93E+01
849.9857	9.90E+01	9.89E+01	9.93E+01
850.4678	9.90E+01	9.89E+01	9.93E+01
850.95	9.90E+01	9.89E+01	9.92E+01
851.4321	9.90E+01	9.89E+01	9.92E+01
851.9142	9.90E+01	9.89E+01	9.92E+01

852.3964	9.90E+01	9.89E+01	9.92E+01
852.8785	9.90E+01	9.89E+01	9.92E+01
853.3606	9.89E+01	9.89E+01	9.92E+01
853.8427	9.89E+01	9.89E+01	9.92E+01
854.3248	9.89E+01	9.89E+01	9.92E+01
854.8069	9.89E+01	9.89E+01	9.92E+01
855.2891	9.89E+01	9.89E+01	9.92E+01
855.7712	9.89E+01	9.89E+01	9.92E+01
856.2534	9.89E+01	9.89E+01	9.92E+01
856.7355	9.89E+01	9.89E+01	9.92E+01
857.2176	9.89E+01	9.89E+01	9.92E+01
857.6997	9.89E+01	9.89E+01	9.92E+01
858.1818	9.89E+01	9.89E+01	9.92E+01
858.6639	9.89E+01	9.89E+01	9.92E+01
859.1461	9.89E+01	9.89E+01	9.92E+01
859.6282	9.89E+01	9.89E+01	9.92E+01
860.1104	9.89E+01	9.89E+01	9.92E+01
860.5925	9.89E+01	9.89E+01	9.92E+01
861.0746	9.89E+01	9.89E+01	9.92E+01
861.5567	9.89E+01	9.89E+01	9.92E+01
862.0388	9.89E+01	9.89E+01	9.92E+01
862.5209	9.89E+01	9.89E+01	9.91E+01
863.0031	9.89E+01	9.89E+01	9.91E+01
863.4852	9.89E+01	9.89E+01	9.91E+01
863.9673	9.89E+01	9.89E+01	9.91E+01
864.4495	9.89E+01	9.90E+01	9.91E+01
864.9316	9.90E+01	9.90E+01	9.91E+01
865.4137	9.90E+01	9.90E+01	9.92E+01
865.8958	9.90E+01	9.90E+01	9.92E+01
866.3779	9.90E+01	9.90E+01	9.92E+01
866.86	9.90E+01	9.90E+01	9.92E+01
867.3422	9.90E+01	9.90E+01	9.92E+01
867.8243	9.90E+01	9.90E+01	9.92E+01
868.3065	9.90E+01	9.89E+01	9.92E+01
868.7886	9.90E+01	9.89E+01	9.92E+01
869.2707	9.90E+01	9.89E+01	9.92E+01
869.7528	9.89E+01	9.89E+01	9.92E+01
870.2349	9.89E+01	9.89E+01	9.92E+01
870.717	9.89E+01	9.89E+01	9.92E+01
871.1992	9.89E+01	9.89E+01	9.92E+01
871.6813	9.90E+01	9.89E+01	9.93E+01
872.1635	9.90E+01	9.90E+01	9.93E+01
872.6456	9.90E+01	9.90E+01	9.93E+01
873.1277	9.90E+01	9.90E+01	9.93E+01
873.6098	9.90E+01	9.90E+01	9.93E+01
874.0919	9.90E+01	9.90E+01	9.93E+01
874.574	9.90E+01	9.90E+01	9.92E+01

875.0562	9.90E+01	9.89E+01	9.92E+01
875.5383	9.89E+01	9.89E+01	9.92E+01
876.0204	9.89E+01	9.89E+01	9.92E+01
876.5026	9.89E+01	9.89E+01	9.92E+01
876.9847	9.89E+01	9.89E+01	9.92E+01
877.4668	9.89E+01	9.89E+01	9.92E+01
877.9489	9.89E+01	9.88E+01	9.92E+01
878.431	9.89E+01	9.88E+01	9.92E+01
878.9132	9.89E+01	9.88E+01	9.92E+01
879.3953	9.89E+01	9.88E+01	9.92E+01
879.8774	9.89E+01	9.88E+01	9.92E+01
880.3596	9.89E+01	9.88E+01	9.92E+01
880.8417	9.89E+01	9.88E+01	9.92E+01
881.3238	9.89E+01	9.88E+01	9.92E+01
881.8059	9.89E+01	9.88E+01	9.92E+01
882.2881	9.89E+01	9.88E+01	9.92E+01
882.7702	9.89E+01	9.89E+01	9.92E+01
883.2523	9.89E+01	9.89E+01	9.92E+01
883.7344	9.89E+01	9.90E+01	9.92E+01
884.2166	9.89E+01	9.90E+01	9.92E+01
884.6987	9.89E+01	9.90E+01	9.93E+01
885.1808	9.89E+01	9.90E+01	9.93E+01
885.6629	9.89E+01	9.90E+01	9.93E+01
886.1451	9.89E+01	9.90E+01	9.92E+01
886.6272	9.89E+01	9.89E+01	9.92E+01
887.1093	9.89E+01	9.89E+01	9.92E+01
887.5914	9.89E+01	9.89E+01	9.92E+01
888.0735	9.89E+01	9.89E+01	9.92E+01
888.5557	9.89E+01	9.88E+01	9.92E+01
889.0378	9.89E+01	9.88E+01	9.92E+01
889.5199	9.89E+01	9.88E+01	9.93E+01
890.0021	9.89E+01	9.88E+01	9.93E+01
890.4842	9.90E+01	9.88E+01	9.93E+01
890.9663	9.90E+01	9.88E+01	9.93E+01
891.4484	9.90E+01	9.88E+01	9.93E+01
891.9305	9.90E+01	9.88E+01	9.93E+01
892.4127	9.90E+01	9.88E+01	9.93E+01
892.8948	9.90E+01	9.88E+01	9.93E+01
893.377	9.90E+01	9.88E+01	9.92E+01
893.8591	9.90E+01	9.88E+01	9.92E+01
894.3412	9.90E+01	9.88E+01	9.92E+01
894.8233	9.89E+01	9.88E+01	9.91E+01
895.3054	9.89E+01	9.88E+01	9.91E+01
895.7875	9.89E+01	9.87E+01	9.91E+01
896.2697	9.89E+01	9.87E+01	9.91E+01
896.7518	9.89E+01	9.87E+01	9.91E+01
897.2339	9.88E+01	9.87E+01	9.91E+01

897.7161	9.88E+01	9.87E+01	9.91E+01
898.1982	9.88E+01	9.87E+01	9.91E+01
898.6803	9.88E+01	9.87E+01	9.91E+01
899.1624	9.89E+01	9.87E+01	9.91E+01
899.6445	9.89E+01	9.87E+01	9.91E+01
900.1266	9.89E+01	9.87E+01	9.91E+01
900.6088	9.89E+01	9.87E+01	9.91E+01
901.0909	9.90E+01	9.87E+01	9.91E+01
901.5731	9.90E+01	9.87E+01	9.91E+01
902.0552	9.90E+01	9.87E+01	9.91E+01
902.5373	9.90E+01	9.87E+01	9.92E+01
903.0194	9.90E+01	9.87E+01	9.92E+01
903.5015	9.90E+01	9.87E+01	9.92E+01
903.9836	9.90E+01	9.87E+01	9.92E+01
904.4658	9.89E+01	9.87E+01	9.92E+01
904.9479	9.89E+01	9.87E+01	9.92E+01
905.4301	9.89E+01	9.87E+01	9.92E+01
905.9122	9.89E+01	9.87E+01	9.92E+01
906.3943	9.90E+01	9.87E+01	9.91E+01
906.8764	9.90E+01	9.87E+01	9.91E+01
907.3585	9.89E+01	9.87E+01	9.91E+01
907.8406	9.89E+01	9.87E+01	9.91E+01
908.3228	9.89E+01	9.87E+01	9.91E+01
908.8049	9.89E+01	9.87E+01	9.91E+01
909.287	9.89E+01	9.87E+01	9.91E+01
909.7692	9.89E+01	9.87E+01	9.91E+01
910.2513	9.88E+01	9.87E+01	9.91E+01
910.7334	9.88E+01	9.87E+01	9.91E+01
911.2155	9.88E+01	9.87E+01	9.91E+01
911.6976	9.88E+01	9.87E+01	9.91E+01
912.1798	9.88E+01	9.87E+01	9.91E+01
912.6619	9.89E+01	9.87E+01	9.92E+01
913.144	9.89E+01	9.87E+01	9.92E+01
913.6262	9.89E+01	9.87E+01	9.92E+01
914.1083	9.89E+01	9.87E+01	9.92E+01
914.5904	9.89E+01	9.87E+01	9.91E+01
915.0725	9.89E+01	9.87E+01	9.91E+01
915.5546	9.89E+01	9.87E+01	9.91E+01
916.0368	9.89E+01	9.87E+01	9.91E+01
916.5189	9.89E+01	9.87E+01	9.91E+01
917.001	9.89E+01	9.87E+01	9.91E+01
917.4832	9.89E+01	9.87E+01	9.91E+01
917.9653	9.89E+01	9.87E+01	9.91E+01
918.4474	9.89E+01	9.87E+01	9.91E+01
918.9295	9.89E+01	9.87E+01	9.91E+01
919.4116	9.89E+01	9.87E+01	9.91E+01
919.8938	9.90E+01	9.87E+01	9.91E+01

920.3759	9.90E+01	9.87E+01	9.92E+01
920.858	9.90E+01	9.87E+01	9.92E+01
921.3401	9.90E+01	9.87E+01	9.92E+01
921.8223	9.89E+01	9.87E+01	9.92E+01
922.3044	9.89E+01	9.87E+01	9.92E+01
922.7865	9.89E+01	9.87E+01	9.92E+01
923.2687	9.89E+01	9.87E+01	9.92E+01
923.7508	9.89E+01	9.86E+01	9.92E+01
924.2329	9.88E+01	9.86E+01	9.91E+01
924.715	9.88E+01	9.86E+01	9.91E+01
925.1971	9.88E+01	9.86E+01	9.91E+01
925.6793	9.88E+01	9.86E+01	9.91E+01
926.1614	9.88E+01	9.86E+01	9.91E+01
926.6435	9.88E+01	9.86E+01	9.91E+01
927.1257	9.88E+01	9.86E+01	9.91E+01
927.6078	9.88E+01	9.86E+01	9.91E+01
928.0899	9.88E+01	9.86E+01	9.91E+01
928.572	9.88E+01	9.86E+01	9.91E+01
929.0541	9.88E+01	9.86E+01	9.90E+01
929.5363	9.88E+01	9.86E+01	9.90E+01
930.0184	9.88E+01	9.86E+01	9.90E+01
930.5005	9.88E+01	9.86E+01	9.90E+01
930.9827	9.88E+01	9.86E+01	9.90E+01
931.4648	9.88E+01	9.86E+01	9.90E+01
931.9469	9.88E+01	9.86E+01	9.90E+01
932.429	9.88E+01	9.86E+01	9.90E+01
932.9111	9.88E+01	9.86E+01	9.90E+01
933.3932	9.88E+01	9.86E+01	9.90E+01
933.8754	9.88E+01	9.86E+01	9.90E+01
934.3575	9.88E+01	9.86E+01	9.90E+01
934.8397	9.88E+01	9.86E+01	9.90E+01
935.3218	9.88E+01	9.86E+01	9.90E+01
935.8039	9.88E+01	9.86E+01	9.90E+01
936.286	9.88E+01	9.86E+01	9.90E+01
936.7681	9.88E+01	9.86E+01	9.90E+01
937.2502	9.88E+01	9.86E+01	9.90E+01
937.7324	9.87E+01	9.86E+01	9.90E+01
938.2145	9.87E+01	9.86E+01	9.90E+01
938.6967	9.88E+01	9.86E+01	9.90E+01
939.1788	9.88E+01	9.86E+01	9.90E+01
939.6609	9.88E+01	9.86E+01	9.90E+01
940.143	9.88E+01	9.86E+01	9.90E+01
940.6251	9.88E+01	9.86E+01	9.90E+01
941.1072	9.88E+01	9.86E+01	9.90E+01
941.5894	9.88E+01	9.86E+01	9.90E+01
942.0715	9.88E+01	9.86E+01	9.90E+01
942.5536	9.88E+01	9.86E+01	9.90E+01

943.0358	9.88E+01	9.86E+01	9.90E+01
943.5179	9.88E+01	9.86E+01	9.90E+01
944	9.88E+01	9.86E+01	9.90E+01
944.4821	9.88E+01	9.85E+01	9.90E+01
944.9642	9.88E+01	9.85E+01	9.90E+01
945.4464	9.88E+01	9.85E+01	9.90E+01
945.9285	9.88E+01	9.85E+01	9.90E+01
946.4106	9.88E+01	9.85E+01	9.90E+01
946.8928	9.88E+01	9.85E+01	9.90E+01
947.3749	9.88E+01	9.84E+01	9.90E+01
947.857	9.88E+01	9.84E+01	9.90E+01
948.3391	9.88E+01	9.84E+01	9.90E+01
948.8212	9.88E+01	9.84E+01	9.90E+01
949.3034	9.88E+01	9.84E+01	9.90E+01
949.7855	9.88E+01	9.84E+01	9.90E+01
950.2676	9.88E+01	9.84E+01	9.90E+01
950.7498	9.88E+01	9.84E+01	9.90E+01
951.2319	9.88E+01	9.84E+01	9.90E+01
951.714	9.88E+01	9.84E+01	9.90E+01
952.1961	9.88E+01	9.85E+01	9.90E+01
952.6782	9.88E+01	9.85E+01	9.90E+01
953.1604	9.88E+01	9.85E+01	9.90E+01
953.6425	9.88E+01	9.84E+01	9.90E+01
954.1246	9.88E+01	9.84E+01	9.90E+01
954.6068	9.88E+01	9.84E+01	9.90E+01
955.0889	9.87E+01	9.84E+01	9.90E+01
955.571	9.87E+01	9.84E+01	9.90E+01
956.0531	9.87E+01	9.84E+01	9.90E+01
956.5352	9.87E+01	9.84E+01	9.90E+01
957.0174	9.88E+01	9.85E+01	9.90E+01
957.4995	9.88E+01	9.85E+01	9.90E+01
957.9816	9.88E+01	9.85E+01	9.90E+01
958.4637	9.88E+01	9.85E+01	9.90E+01
958.9459	9.88E+01	9.85E+01	9.90E+01
959.428	9.88E+01	9.84E+01	9.90E+01
959.9101	9.88E+01	9.84E+01	9.90E+01
960.3922	9.88E+01	9.84E+01	9.90E+01
960.8744	9.88E+01	9.84E+01	9.90E+01
961.3565	9.88E+01	9.84E+01	9.90E+01
961.8386	9.88E+01	9.84E+01	9.90E+01
962.3207	9.88E+01	9.84E+01	9.90E+01
962.8029	9.88E+01	9.84E+01	9.90E+01
963.285	9.88E+01	9.84E+01	9.90E+01
963.7671	9.88E+01	9.84E+01	9.90E+01
964.2493	9.88E+01	9.84E+01	9.90E+01
964.7314	9.88E+01	9.84E+01	9.90E+01
965.2135	9.88E+01	9.84E+01	9.90E+01

965.6956	9.88E+01	9.84E+01	9.90E+01
966.1777	9.88E+01	9.84E+01	9.90E+01
966.6599	9.88E+01	9.84E+01	9.90E+01
967.142	9.88E+01	9.84E+01	9.89E+01
967.6241	9.88E+01	9.83E+01	9.89E+01
968.1063	9.88E+01	9.83E+01	9.89E+01
968.5884	9.88E+01	9.83E+01	9.89E+01
969.0705	9.88E+01	9.83E+01	9.89E+01
969.5526	9.88E+01	9.83E+01	9.89E+01
970.0347	9.89E+01	9.82E+01	9.89E+01
970.5168	9.89E+01	9.83E+01	9.90E+01
970.999	9.89E+01	9.83E+01	9.90E+01
971.4811	9.89E+01	9.83E+01	9.90E+01
971.9633	9.89E+01	9.83E+01	9.90E+01
972.4454	9.89E+01	9.83E+01	9.90E+01
972.9275	9.89E+01	9.83E+01	9.90E+01
973.4096	9.89E+01	9.83E+01	9.90E+01
973.8917	9.89E+01	9.83E+01	9.90E+01
974.3738	9.89E+01	9.83E+01	9.90E+01
974.856	9.89E+01	9.83E+01	9.91E+01
975.3381	9.89E+01	9.83E+01	9.91E+01
975.8203	9.89E+01	9.83E+01	9.91E+01
976.3024	9.89E+01	9.83E+01	9.91E+01
976.7845	9.89E+01	9.83E+01	9.91E+01
977.2666	9.89E+01	9.83E+01	9.91E+01
977.7487	9.89E+01	9.83E+01	9.91E+01
978.2308	9.89E+01	9.83E+01	9.90E+01
978.713	9.89E+01	9.83E+01	9.90E+01
979.1951	9.89E+01	9.83E+01	9.90E+01
979.6772	9.89E+01	9.82E+01	9.90E+01
980.1594	9.89E+01	9.82E+01	9.90E+01
980.6415	9.89E+01	9.82E+01	9.90E+01
981.1236	9.88E+01	9.82E+01	9.90E+01
981.6057	9.88E+01	9.82E+01	9.90E+01
982.0878	9.88E+01	9.82E+01	9.90E+01
982.5699	9.88E+01	9.82E+01	9.90E+01
983.0521	9.89E+01	9.82E+01	9.90E+01
983.5342	9.89E+01	9.82E+01	9.90E+01
984.0164	9.89E+01	9.82E+01	9.90E+01
984.4985	9.89E+01	9.82E+01	9.90E+01
984.9806	9.89E+01	9.82E+01	9.90E+01
985.4627	9.89E+01	9.82E+01	9.90E+01
985.9448	9.89E+01	9.82E+01	9.90E+01
986.4269	9.89E+01	9.82E+01	9.90E+01
986.9091	9.89E+01	9.82E+01	9.90E+01
987.3912	9.89E+01	9.82E+01	9.90E+01
987.8734	9.89E+01	9.82E+01	9.90E+01

988.3555	9.89E+01	9.82E+01	9.90E+01
988.8376	9.89E+01	9.82E+01	9.90E+01
989.3197	9.89E+01	9.82E+01	9.90E+01
989.8018	9.88E+01	9.81E+01	9.90E+01
990.284	9.88E+01	9.81E+01	9.90E+01
990.7661	9.88E+01	9.81E+01	9.90E+01
991.2482	9.89E+01	9.81E+01	9.90E+01
991.7303	9.89E+01	9.81E+01	9.90E+01
992.2125	9.89E+01	9.81E+01	9.90E+01
992.6946	9.89E+01	9.81E+01	9.90E+01
993.1767	9.89E+01	9.81E+01	9.90E+01
993.6588	9.89E+01	9.81E+01	9.90E+01
994.141	9.89E+01	9.81E+01	9.90E+01
994.6231	9.89E+01	9.81E+01	9.90E+01
995.1052	9.88E+01	9.81E+01	9.90E+01
995.5873	9.88E+01	9.81E+01	9.89E+01
996.0695	9.88E+01	9.81E+01	9.89E+01
996.5516	9.88E+01	9.81E+01	9.89E+01
997.0337	9.88E+01	9.81E+01	9.89E+01
997.5158	9.88E+01	9.81E+01	9.89E+01
997.998	9.88E+01	9.81E+01	9.89E+01
998.4801	9.88E+01	9.81E+01	9.89E+01
998.9622	9.89E+01	9.81E+01	9.89E+01
999.4443	9.89E+01	9.81E+01	9.89E+01
999.9265	9.89E+01	9.81E+01	9.89E+01
1000.409	9.89E+01	9.81E+01	9.89E+01
1000.891	9.89E+01	9.80E+01	9.89E+01
1001.373	9.89E+01	9.80E+01	9.89E+01
1001.855	9.88E+01	9.80E+01	9.89E+01
1002.337	9.88E+01	9.80E+01	9.89E+01
1002.819	9.88E+01	9.80E+01	9.89E+01
1003.301	9.88E+01	9.80E+01	9.89E+01
1003.783	9.88E+01	9.80E+01	9.89E+01
1004.266	9.88E+01	9.80E+01	9.88E+01
1004.748	9.88E+01	9.80E+01	9.88E+01
1005.23	9.88E+01	9.80E+01	9.88E+01
1005.712	9.87E+01	9.80E+01	9.88E+01
1006.194	9.87E+01	9.80E+01	9.88E+01
1006.676	9.87E+01	9.79E+01	9.88E+01
1007.158	9.87E+01	9.79E+01	9.88E+01
1007.64	9.87E+01	9.79E+01	9.88E+01
1008.123	9.87E+01	9.79E+01	9.88E+01
1008.605	9.87E+01	9.79E+01	9.88E+01
1009.087	9.87E+01	9.79E+01	9.88E+01
1009.569	9.87E+01	9.79E+01	9.88E+01
1010.051	9.87E+01	9.79E+01	9.88E+01
1010.533	9.87E+01	9.79E+01	9.88E+01

1011.015	9.87E+01	9.79E+01	9.88E+01
1011.497	9.87E+01	9.79E+01	9.88E+01
1011.98	9.87E+01	9.79E+01	9.88E+01
1012.462	9.87E+01	9.79E+01	9.88E+01
1012.944	9.87E+01	9.78E+01	9.88E+01
1013.426	9.87E+01	9.78E+01	9.88E+01
1013.908	9.87E+01	9.78E+01	9.88E+01
1014.39	9.87E+01	9.78E+01	9.88E+01
1014.872	9.87E+01	9.78E+01	9.88E+01
1015.354	9.87E+01	9.78E+01	9.88E+01
1015.837	9.87E+01	9.78E+01	9.88E+01
1016.319	9.87E+01	9.78E+01	9.89E+01
1016.801	9.87E+01	9.78E+01	9.89E+01
1017.283	9.87E+01	9.78E+01	9.89E+01
1017.765	9.87E+01	9.79E+01	9.89E+01
1018.247	9.87E+01	9.78E+01	9.89E+01
1018.729	9.87E+01	9.78E+01	9.89E+01
1019.211	9.87E+01	9.78E+01	9.88E+01
1019.694	9.87E+01	9.78E+01	9.88E+01
1020.176	9.87E+01	9.78E+01	9.88E+01
1020.658	9.87E+01	9.78E+01	9.88E+01
1021.14	9.87E+01	9.78E+01	9.88E+01
1021.622	9.88E+01	9.78E+01	9.88E+01
1022.104	9.88E+01	9.77E+01	9.88E+01
1022.586	9.88E+01	9.77E+01	9.88E+01
1023.068	9.87E+01	9.77E+01	9.88E+01
1023.551	9.87E+01	9.77E+01	9.88E+01
1024.033	9.87E+01	9.77E+01	9.87E+01
1024.515	9.87E+01	9.77E+01	9.87E+01
1024.997	9.87E+01	9.77E+01	9.87E+01
1025.479	9.86E+01	9.77E+01	9.87E+01
1025.961	9.86E+01	9.77E+01	9.87E+01
1026.443	9.86E+01	9.77E+01	9.87E+01
1026.925	9.87E+01	9.77E+01	9.87E+01
1027.408	9.87E+01	9.77E+01	9.87E+01
1027.89	9.87E+01	9.76E+01	9.87E+01
1028.372	9.87E+01	9.76E+01	9.87E+01
1028.854	9.87E+01	9.76E+01	9.87E+01
1029.336	9.87E+01	9.76E+01	9.87E+01
1029.818	9.87E+01	9.76E+01	9.87E+01
1030.3	9.86E+01	9.76E+01	9.87E+01
1030.782	9.86E+01	9.76E+01	9.87E+01
1031.265	9.86E+01	9.76E+01	9.87E+01
1031.747	9.86E+01	9.76E+01	9.87E+01
1032.229	9.86E+01	9.76E+01	9.87E+01
1032.711	9.86E+01	9.76E+01	9.87E+01
1033.193	9.86E+01	9.75E+01	9.87E+01

1033.675	9.86E+01	9.75E+01	9.87E+01
1034.157	9.86E+01	9.75E+01	9.87E+01
1034.639	9.86E+01	9.75E+01	9.87E+01
1035.122	9.85E+01	9.75E+01	9.87E+01
1035.604	9.85E+01	9.75E+01	9.87E+01
1036.086	9.85E+01	9.75E+01	9.87E+01
1036.568	9.85E+01	9.75E+01	9.87E+01
1037.05	9.85E+01	9.75E+01	9.87E+01
1037.532	9.85E+01	9.75E+01	9.87E+01
1038.014	9.85E+01	9.74E+01	9.87E+01
1038.496	9.84E+01	9.74E+01	9.86E+01
1038.979	9.84E+01	9.74E+01	9.86E+01
1039.461	9.84E+01	9.74E+01	9.86E+01
1039.943	9.84E+01	9.74E+01	9.86E+01
1040.425	9.83E+01	9.74E+01	9.85E+01
1040.907	9.83E+01	9.74E+01	9.85E+01
1041.389	9.83E+01	9.74E+01	9.85E+01
1041.871	9.83E+01	9.74E+01	9.85E+01
1042.353	9.83E+01	9.74E+01	9.85E+01
1042.836	9.83E+01	9.74E+01	9.85E+01
1043.318	9.83E+01	9.74E+01	9.85E+01
1043.8	9.83E+01	9.74E+01	9.85E+01
1044.282	9.83E+01	9.74E+01	9.85E+01
1044.764	9.83E+01	9.74E+01	9.85E+01
1045.246	9.83E+01	9.74E+01	9.85E+01
1045.728	9.83E+01	9.74E+01	9.85E+01
1046.21	9.83E+01	9.74E+01	9.86E+01
1046.693	9.83E+01	9.74E+01	9.86E+01
1047.175	9.83E+01	9.74E+01	9.86E+01
1047.657	9.83E+01	9.74E+01	9.86E+01
1048.139	9.83E+01	9.74E+01	9.86E+01
1048.621	9.83E+01	9.74E+01	9.86E+01
1049.103	9.83E+01	9.74E+01	9.86E+01
1049.585	9.83E+01	9.74E+01	9.86E+01
1050.067	9.83E+01	9.74E+01	9.86E+01
1050.55	9.83E+01	9.74E+01	9.86E+01
1051.032	9.83E+01	9.74E+01	9.86E+01
1051.514	9.83E+01	9.74E+01	9.86E+01
1051.996	9.83E+01	9.74E+01	9.86E+01
1052.478	9.83E+01	9.74E+01	9.86E+01
1052.96	9.83E+01	9.74E+01	9.86E+01
1053.442	9.82E+01	9.74E+01	9.86E+01
1053.924	9.82E+01	9.74E+01	9.86E+01
1054.406	9.82E+01	9.74E+01	9.85E+01
1054.889	9.82E+01	9.74E+01	9.85E+01
1055.371	9.81E+01	9.73E+01	9.85E+01
1055.853	9.81E+01	9.73E+01	9.85E+01

1056.335	9.81E+01	9.73E+01	9.84E+01
1056.817	9.81E+01	9.73E+01	9.84E+01
1057.299	9.80E+01	9.73E+01	9.84E+01
1057.781	9.80E+01	9.72E+01	9.84E+01
1058.264	9.80E+01	9.72E+01	9.83E+01
1058.746	9.80E+01	9.71E+01	9.83E+01
1059.228	9.80E+01	9.71E+01	9.83E+01
1059.71	9.80E+01	9.71E+01	9.83E+01
1060.192	9.80E+01	9.71E+01	9.83E+01
1060.674	9.80E+01	9.71E+01	9.83E+01
1061.156	9.80E+01	9.71E+01	9.83E+01
1061.638	9.79E+01	9.71E+01	9.84E+01
1062.12	9.79E+01	9.71E+01	9.84E+01
1062.603	9.79E+01	9.71E+01	9.84E+01
1063.085	9.79E+01	9.71E+01	9.83E+01
1063.567	9.79E+01	9.71E+01	9.83E+01
1064.049	9.79E+01	9.71E+01	9.83E+01
1064.531	9.78E+01	9.71E+01	9.83E+01
1065.013	9.78E+01	9.71E+01	9.83E+01
1065.495	9.78E+01	9.71E+01	9.83E+01
1065.978	9.78E+01	9.70E+01	9.83E+01
1066.46	9.77E+01	9.71E+01	9.83E+01
1066.942	9.77E+01	9.71E+01	9.83E+01
1067.424	9.77E+01	9.71E+01	9.83E+01
1067.906	9.77E+01	9.71E+01	9.83E+01
1068.388	9.77E+01	9.72E+01	9.83E+01
1068.87	9.77E+01	9.72E+01	9.83E+01
1069.352	9.78E+01	9.72E+01	9.83E+01
1069.834	9.78E+01	9.73E+01	9.84E+01
1070.317	9.78E+01	9.73E+01	9.84E+01
1070.799	9.78E+01	9.73E+01	9.84E+01
1071.281	9.78E+01	9.73E+01	9.84E+01
1071.763	9.79E+01	9.73E+01	9.84E+01
1072.245	9.79E+01	9.73E+01	9.84E+01
1072.727	9.79E+01	9.73E+01	9.84E+01
1073.209	9.79E+01	9.73E+01	9.84E+01
1073.692	9.78E+01	9.73E+01	9.84E+01
1074.174	9.78E+01	9.73E+01	9.84E+01
1074.656	9.78E+01	9.73E+01	9.84E+01
1075.138	9.78E+01	9.72E+01	9.84E+01
1075.62	9.78E+01	9.72E+01	9.83E+01
1076.102	9.78E+01	9.72E+01	9.83E+01
1076.584	9.78E+01	9.72E+01	9.83E+01
1077.066	9.78E+01	9.72E+01	9.83E+01
1077.548	9.78E+01	9.72E+01	9.83E+01
1078.031	9.78E+01	9.72E+01	9.83E+01
1078.513	9.78E+01	9.72E+01	9.83E+01

1078.995	9.79E+01	9.72E+01	9.83E+01
1079.477	9.79E+01	9.72E+01	9.83E+01
1079.959	9.79E+01	9.73E+01	9.83E+01
1080.441	9.79E+01	9.73E+01	9.83E+01
1080.923	9.79E+01	9.73E+01	9.83E+01
1081.406	9.79E+01	9.73E+01	9.83E+01
1081.888	9.79E+01	9.73E+01	9.83E+01
1082.37	9.79E+01	9.73E+01	9.84E+01
1082.852	9.79E+01	9.74E+01	9.84E+01
1083.334	9.79E+01	9.74E+01	9.84E+01
1083.816	9.79E+01	9.74E+01	9.84E+01
1084.298	9.79E+01	9.74E+01	9.84E+01
1084.78	9.79E+01	9.74E+01	9.84E+01
1085.262	9.80E+01	9.74E+01	9.84E+01
1085.745	9.80E+01	9.74E+01	9.84E+01
1086.227	9.80E+01	9.75E+01	9.84E+01
1086.709	9.80E+01	9.75E+01	9.84E+01
1087.191	9.80E+01	9.75E+01	9.85E+01
1087.673	9.80E+01	9.75E+01	9.85E+01
1088.155	9.81E+01	9.75E+01	9.85E+01
1088.637	9.81E+01	9.75E+01	9.85E+01
1089.12	9.81E+01	9.75E+01	9.85E+01
1089.602	9.81E+01	9.75E+01	9.85E+01
1090.084	9.81E+01	9.75E+01	9.85E+01
1090.566	9.81E+01	9.76E+01	9.85E+01
1091.048	9.81E+01	9.76E+01	9.85E+01
1091.53	9.81E+01	9.76E+01	9.85E+01
1092.012	9.81E+01	9.76E+01	9.85E+01
1092.494	9.81E+01	9.76E+01	9.85E+01
1092.977	9.80E+01	9.76E+01	9.85E+01
1093.459	9.80E+01	9.76E+01	9.85E+01
1093.941	9.80E+01	9.76E+01	9.85E+01
1094.423	9.80E+01	9.76E+01	9.85E+01
1094.905	9.80E+01	9.76E+01	9.85E+01
1095.387	9.80E+01	9.76E+01	9.85E+01
1095.869	9.81E+01	9.76E+01	9.85E+01
1096.351	9.81E+01	9.76E+01	9.85E+01
1096.833	9.81E+01	9.76E+01	9.85E+01
1097.316	9.81E+01	9.76E+01	9.85E+01
1097.798	9.81E+01	9.76E+01	9.85E+01
1098.28	9.82E+01	9.76E+01	9.85E+01
1098.762	9.82E+01	9.76E+01	9.85E+01
1099.244	9.82E+01	9.77E+01	9.85E+01
1099.726	9.82E+01	9.77E+01	9.85E+01
1100.208	9.82E+01	9.77E+01	9.86E+01
1100.691	9.82E+01	9.77E+01	9.86E+01
1101.173	9.82E+01	9.77E+01	9.86E+01

1101.655	9.82E+01	9.77E+01	9.86E+01
1102.137	9.82E+01	9.77E+01	9.86E+01
1102.619	9.82E+01	9.77E+01	9.86E+01
1103.101	9.82E+01	9.77E+01	9.86E+01
1103.583	9.82E+01	9.77E+01	9.86E+01
1104.065	9.82E+01	9.77E+01	9.86E+01
1104.547	9.82E+01	9.77E+01	9.86E+01
1105.03	9.82E+01	9.77E+01	9.86E+01
1105.512	9.82E+01	9.77E+01	9.86E+01
1105.994	9.82E+01	9.77E+01	9.86E+01
1106.476	9.82E+01	9.77E+01	9.87E+01
1106.958	9.82E+01	9.78E+01	9.87E+01
1107.44	9.83E+01	9.77E+01	9.87E+01
1107.922	9.82E+01	9.77E+01	9.87E+01
1108.405	9.82E+01	9.77E+01	9.87E+01
1108.887	9.82E+01	9.77E+01	9.86E+01
1109.369	9.82E+01	9.77E+01	9.86E+01
1109.851	9.82E+01	9.77E+01	9.86E+01
1110.333	9.82E+01	9.77E+01	9.86E+01
1110.815	9.81E+01	9.76E+01	9.86E+01
1111.297	9.81E+01	9.76E+01	9.86E+01
1111.779	9.81E+01	9.76E+01	9.86E+01
1112.261	9.81E+01	9.76E+01	9.86E+01
1112.744	9.81E+01	9.76E+01	9.86E+01
1113.226	9.82E+01	9.77E+01	9.86E+01
1113.708	9.82E+01	9.77E+01	9.86E+01
1114.19	9.82E+01	9.77E+01	9.86E+01
1114.672	9.82E+01	9.77E+01	9.86E+01
1115.154	9.82E+01	9.78E+01	9.86E+01
1115.636	9.82E+01	9.78E+01	9.86E+01
1116.119	9.82E+01	9.78E+01	9.87E+01
1116.601	9.82E+01	9.78E+01	9.87E+01
1117.083	9.83E+01	9.78E+01	9.87E+01
1117.565	9.83E+01	9.78E+01	9.87E+01
1118.047	9.83E+01	9.78E+01	9.87E+01
1118.529	9.83E+01	9.78E+01	9.86E+01
1119.011	9.83E+01	9.79E+01	9.86E+01
1119.493	9.83E+01	9.79E+01	9.86E+01
1119.975	9.83E+01	9.79E+01	9.86E+01
1120.458	9.83E+01	9.79E+01	9.86E+01
1120.94	9.83E+01	9.79E+01	9.86E+01
1121.422	9.83E+01	9.80E+01	9.86E+01
1121.904	9.83E+01	9.80E+01	9.86E+01
1122.386	9.83E+01	9.80E+01	9.86E+01
1122.868	9.83E+01	9.80E+01	9.86E+01
1123.35	9.83E+01	9.80E+01	9.86E+01
1123.833	9.83E+01	9.80E+01	9.86E+01

1124.315	9.83E+01	9.80E+01	9.86E+01
1124.797	9.83E+01	9.80E+01	9.86E+01
1125.279	9.83E+01	9.80E+01	9.87E+01
1125.761	9.83E+01	9.80E+01	9.87E+01
1126.243	9.83E+01	9.80E+01	9.87E+01
1126.725	9.84E+01	9.80E+01	9.87E+01
1127.207	9.84E+01	9.80E+01	9.87E+01
1127.689	9.84E+01	9.81E+01	9.88E+01
1128.172	9.84E+01	9.81E+01	9.88E+01
1128.654	9.84E+01	9.81E+01	9.88E+01
1129.136	9.85E+01	9.81E+01	9.88E+01
1129.618	9.85E+01	9.81E+01	9.88E+01
1130.1	9.85E+01	9.81E+01	9.88E+01
1130.582	9.85E+01	9.81E+01	9.88E+01
1131.064	9.85E+01	9.81E+01	9.88E+01
1131.547	9.85E+01	9.81E+01	9.88E+01
1132.029	9.85E+01	9.81E+01	9.88E+01
1132.511	9.85E+01	9.81E+01	9.88E+01
1132.993	9.84E+01	9.81E+01	9.88E+01
1133.475	9.84E+01	9.81E+01	9.88E+01
1133.957	9.84E+01	9.81E+01	9.88E+01
1134.439	9.84E+01	9.81E+01	9.88E+01
1134.921	9.84E+01	9.81E+01	9.88E+01
1135.403	9.84E+01	9.81E+01	9.88E+01
1135.886	9.84E+01	9.81E+01	9.88E+01
1136.368	9.85E+01	9.81E+01	9.88E+01
1136.85	9.85E+01	9.82E+01	9.88E+01
1137.332	9.85E+01	9.82E+01	9.88E+01
1137.814	9.85E+01	9.82E+01	9.88E+01
1138.296	9.85E+01	9.82E+01	9.88E+01
1138.778	9.85E+01	9.82E+01	9.88E+01
1139.26	9.85E+01	9.82E+01	9.88E+01
1139.743	9.85E+01	9.81E+01	9.88E+01
1140.225	9.84E+01	9.81E+01	9.88E+01
1140.707	9.84E+01	9.81E+01	9.88E+01
1141.189	9.84E+01	9.81E+01	9.88E+01
1141.671	9.84E+01	9.81E+01	9.88E+01
1142.153	9.84E+01	9.81E+01	9.87E+01
1142.635	9.84E+01	9.81E+01	9.87E+01
1143.117	9.84E+01	9.81E+01	9.87E+01
1143.6	9.84E+01	9.81E+01	9.87E+01
1144.082	9.83E+01	9.81E+01	9.87E+01
1144.564	9.83E+01	9.81E+01	9.87E+01
1145.046	9.83E+01	9.81E+01	9.87E+01
1145.528	9.83E+01	9.80E+01	9.87E+01
1146.01	9.83E+01	9.80E+01	9.87E+01
1146.492	9.83E+01	9.80E+01	9.87E+01

1146.974	9.82E+01	9.80E+01	9.87E+01
1147.457	9.82E+01	9.80E+01	9.86E+01
1147.939	9.82E+01	9.80E+01	9.86E+01
1148.421	9.82E+01	9.80E+01	9.86E+01
1148.903	9.82E+01	9.79E+01	9.86E+01
1149.385	9.82E+01	9.79E+01	9.86E+01
1149.867	9.81E+01	9.79E+01	9.86E+01
1150.349	9.81E+01	9.79E+01	9.86E+01
1150.831	9.81E+01	9.79E+01	9.86E+01
1151.314	9.81E+01	9.78E+01	9.86E+01
1151.796	9.81E+01	9.78E+01	9.86E+01
1152.278	9.80E+01	9.78E+01	9.85E+01
1152.76	9.80E+01	9.77E+01	9.85E+01
1153.242	9.80E+01	9.77E+01	9.85E+01
1153.724	9.80E+01	9.77E+01	9.85E+01
1154.206	9.80E+01	9.77E+01	9.85E+01
1154.688	9.80E+01	9.77E+01	9.85E+01
1155.171	9.80E+01	9.77E+01	9.85E+01
1155.653	9.80E+01	9.76E+01	9.85E+01
1156.135	9.80E+01	9.76E+01	9.85E+01
1156.617	9.79E+01	9.76E+01	9.85E+01
1157.099	9.79E+01	9.75E+01	9.85E+01
1157.581	9.78E+01	9.75E+01	9.84E+01
1158.063	9.78E+01	9.75E+01	9.84E+01
1158.545	9.77E+01	9.74E+01	9.84E+01
1159.028	9.77E+01	9.74E+01	9.84E+01
1159.51	9.77E+01	9.74E+01	9.84E+01
1159.992	9.77E+01	9.74E+01	9.84E+01
1160.474	9.77E+01	9.73E+01	9.84E+01
1160.956	9.76E+01	9.73E+01	9.83E+01
1161.438	9.76E+01	9.72E+01	9.83E+01
1161.92	9.76E+01	9.72E+01	9.83E+01
1162.402	9.76E+01	9.72E+01	9.82E+01
1162.885	9.75E+01	9.72E+01	9.82E+01
1163.367	9.75E+01	9.71E+01	9.82E+01
1163.849	9.75E+01	9.71E+01	9.82E+01
1164.331	9.75E+01	9.71E+01	9.82E+01
1164.813	9.74E+01	9.71E+01	9.82E+01
1165.295	9.74E+01	9.71E+01	9.82E+01
1165.777	9.74E+01	9.71E+01	9.82E+01
1166.259	9.74E+01	9.71E+01	9.81E+01
1166.742	9.74E+01	9.71E+01	9.81E+01
1167.224	9.74E+01	9.71E+01	9.81E+01
1167.706	9.74E+01	9.71E+01	9.81E+01
1168.188	9.74E+01	9.71E+01	9.81E+01
1168.67	9.74E+01	9.71E+01	9.81E+01
1169.152	9.74E+01	9.72E+01	9.81E+01

1169.634	9.74E+01	9.72E+01	9.81E+01
1170.116	9.74E+01	9.72E+01	9.81E+01
1170.599	9.74E+01	9.72E+01	9.81E+01
1171.081	9.73E+01	9.71E+01	9.81E+01
1171.563	9.73E+01	9.71E+01	9.81E+01
1172.045	9.73E+01	9.71E+01	9.81E+01
1172.527	9.73E+01	9.71E+01	9.82E+01
1173.009	9.73E+01	9.71E+01	9.82E+01
1173.491	9.73E+01	9.71E+01	9.82E+01
1173.973	9.73E+01	9.72E+01	9.82E+01
1174.456	9.73E+01	9.72E+01	9.82E+01
1174.938	9.73E+01	9.72E+01	9.82E+01
1175.42	9.73E+01	9.72E+01	9.82E+01
1175.902	9.74E+01	9.72E+01	9.82E+01
1176.384	9.74E+01	9.73E+01	9.82E+01
1176.866	9.74E+01	9.73E+01	9.83E+01
1177.348	9.74E+01	9.73E+01	9.83E+01
1177.83	9.74E+01	9.73E+01	9.83E+01
1178.313	9.74E+01	9.74E+01	9.83E+01
1178.795	9.75E+01	9.74E+01	9.83E+01
1179.277	9.75E+01	9.74E+01	9.83E+01
1179.759	9.75E+01	9.74E+01	9.83E+01
1180.241	9.75E+01	9.74E+01	9.83E+01
1180.723	9.75E+01	9.74E+01	9.83E+01
1181.205	9.75E+01	9.74E+01	9.83E+01
1181.687	9.75E+01	9.74E+01	9.83E+01
1182.17	9.75E+01	9.75E+01	9.83E+01
1182.652	9.75E+01	9.75E+01	9.83E+01
1183.134	9.75E+01	9.75E+01	9.83E+01
1183.616	9.75E+01	9.75E+01	9.83E+01
1184.098	9.75E+01	9.75E+01	9.83E+01
1184.58	9.75E+01	9.75E+01	9.83E+01
1185.062	9.75E+01	9.75E+01	9.83E+01
1185.544	9.75E+01	9.75E+01	9.83E+01
1186.027	9.75E+01	9.75E+01	9.83E+01
1186.509	9.75E+01	9.75E+01	9.83E+01
1186.991	9.74E+01	9.75E+01	9.83E+01
1187.473	9.74E+01	9.75E+01	9.83E+01
1187.955	9.74E+01	9.75E+01	9.83E+01
1188.437	9.74E+01	9.75E+01	9.83E+01
1188.919	9.74E+01	9.75E+01	9.83E+01
1189.401	9.74E+01	9.75E+01	9.83E+01
1189.884	9.74E+01	9.75E+01	9.83E+01
1190.366	9.74E+01	9.75E+01	9.82E+01
1190.848	9.74E+01	9.75E+01	9.82E+01
1191.33	9.75E+01	9.75E+01	9.82E+01
1191.812	9.75E+01	9.75E+01	9.82E+01

1192.294	9.74E+01	9.74E+01	9.82E+01
1192.776	9.74E+01	9.74E+01	9.82E+01
1193.258	9.74E+01	9.74E+01	9.82E+01
1193.741	9.74E+01	9.74E+01	9.82E+01
1194.223	9.74E+01	9.74E+01	9.82E+01
1194.705	9.74E+01	9.74E+01	9.82E+01
1195.187	9.74E+01	9.73E+01	9.82E+01
1195.669	9.74E+01	9.73E+01	9.82E+01
1196.151	9.74E+01	9.73E+01	9.82E+01
1196.633	9.73E+01	9.73E+01	9.82E+01
1197.115	9.73E+01	9.73E+01	9.82E+01
1197.598	9.73E+01	9.73E+01	9.82E+01
1198.08	9.73E+01	9.73E+01	9.82E+01
1198.562	9.73E+01	9.73E+01	9.82E+01
1199.044	9.73E+01	9.73E+01	9.83E+01
1199.526	9.73E+01	9.73E+01	9.83E+01
1200.008	9.73E+01	9.73E+01	9.83E+01
1200.49	9.73E+01	9.73E+01	9.83E+01
1200.972	9.73E+01	9.73E+01	9.83E+01
1201.455	9.73E+01	9.73E+01	9.83E+01
1201.937	9.73E+01	9.73E+01	9.82E+01
1202.419	9.73E+01	9.73E+01	9.82E+01
1202.901	9.73E+01	9.73E+01	9.82E+01
1203.383	9.73E+01	9.73E+01	9.82E+01
1203.865	9.73E+01	9.73E+01	9.82E+01
1204.347	9.73E+01	9.73E+01	9.83E+01
1204.829	9.73E+01	9.73E+01	9.83E+01
1205.312	9.73E+01	9.73E+01	9.83E+01
1205.794	9.73E+01	9.73E+01	9.83E+01
1206.276	9.73E+01	9.73E+01	9.83E+01
1206.758	9.73E+01	9.73E+01	9.83E+01
1207.24	9.74E+01	9.73E+01	9.83E+01
1207.722	9.74E+01	9.73E+01	9.83E+01
1208.204	9.74E+01	9.73E+01	9.82E+01
1208.686	9.74E+01	9.73E+01	9.82E+01
1209.169	9.74E+01	9.73E+01	9.82E+01
1209.651	9.74E+01	9.73E+01	9.82E+01
1210.133	9.74E+01	9.73E+01	9.82E+01
1210.615	9.74E+01	9.73E+01	9.82E+01
1211.097	9.74E+01	9.73E+01	9.82E+01
1211.579	9.74E+01	9.73E+01	9.82E+01
1212.061	9.74E+01	9.73E+01	9.82E+01
1212.543	9.74E+01	9.73E+01	9.82E+01
1213.026	9.74E+01	9.73E+01	9.82E+01
1213.508	9.74E+01	9.73E+01	9.82E+01
1213.99	9.74E+01	9.73E+01	9.82E+01
1214.472	9.74E+01	9.74E+01	9.82E+01

1214.954	9.74E+01	9.74E+01	9.82E+01
1215.436	9.74E+01	9.74E+01	9.82E+01
1215.918	9.74E+01	9.74E+01	9.82E+01
1216.4	9.74E+01	9.74E+01	9.82E+01
1216.883	9.74E+01	9.74E+01	9.82E+01
1217.365	9.74E+01	9.74E+01	9.82E+01
1217.847	9.74E+01	9.74E+01	9.82E+01
1218.329	9.74E+01	9.74E+01	9.82E+01
1218.811	9.74E+01	9.74E+01	9.82E+01
1219.293	9.74E+01	9.73E+01	9.82E+01
1219.775	9.74E+01	9.73E+01	9.82E+01
1220.257	9.73E+01	9.73E+01	9.82E+01
1220.74	9.73E+01	9.73E+01	9.82E+01
1221.222	9.73E+01	9.73E+01	9.82E+01
1221.704	9.73E+01	9.73E+01	9.82E+01
1222.186	9.73E+01	9.73E+01	9.82E+01
1222.668	9.73E+01	9.73E+01	9.82E+01
1223.15	9.73E+01	9.73E+01	9.82E+01
1223.632	9.73E+01	9.73E+01	9.82E+01
1224.114	9.73E+01	9.73E+01	9.82E+01
1224.597	9.73E+01	9.73E+01	9.82E+01
1225.079	9.73E+01	9.72E+01	9.82E+01
1225.561	9.73E+01	9.72E+01	9.82E+01
1226.043	9.73E+01	9.72E+01	9.82E+01
1226.525	9.73E+01	9.72E+01	9.82E+01
1227.007	9.73E+01	9.73E+01	9.82E+01
1227.489	9.73E+01	9.73E+01	9.82E+01
1227.971	9.73E+01	9.73E+01	9.82E+01
1228.453	9.73E+01	9.73E+01	9.82E+01
1228.936	9.73E+01	9.73E+01	9.82E+01
1229.418	9.73E+01	9.73E+01	9.82E+01
1229.9	9.73E+01	9.73E+01	9.82E+01
1230.382	9.73E+01	9.73E+01	9.82E+01
1230.864	9.73E+01	9.73E+01	9.82E+01
1231.346	9.73E+01	9.73E+01	9.82E+01
1231.828	9.73E+01	9.73E+01	9.82E+01
1232.311	9.74E+01	9.73E+01	9.82E+01
1232.793	9.74E+01	9.73E+01	9.83E+01
1233.275	9.74E+01	9.73E+01	9.83E+01
1233.757	9.74E+01	9.73E+01	9.83E+01
1234.239	9.74E+01	9.74E+01	9.83E+01
1234.721	9.74E+01	9.74E+01	9.83E+01
1235.203	9.74E+01	9.74E+01	9.83E+01
1235.685	9.74E+01	9.74E+01	9.83E+01
1236.167	9.74E+01	9.74E+01	9.83E+01
1236.65	9.74E+01	9.74E+01	9.83E+01
1237.132	9.74E+01	9.74E+01	9.83E+01

1237.614	9.74E+01	9.74E+01	9.83E+01
1238.096	9.74E+01	9.74E+01	9.83E+01
1238.578	9.74E+01	9.73E+01	9.83E+01
1239.06	9.74E+01	9.73E+01	9.83E+01
1239.542	9.74E+01	9.73E+01	9.83E+01
1240.025	9.74E+01	9.73E+01	9.83E+01
1240.507	9.74E+01	9.73E+01	9.83E+01
1240.989	9.74E+01	9.73E+01	9.83E+01
1241.471	9.74E+01	9.73E+01	9.83E+01
1241.953	9.74E+01	9.73E+01	9.83E+01
1242.435	9.74E+01	9.73E+01	9.83E+01
1242.917	9.74E+01	9.73E+01	9.83E+01
1243.399	9.74E+01	9.73E+01	9.83E+01
1243.881	9.74E+01	9.73E+01	9.83E+01
1244.364	9.74E+01	9.73E+01	9.83E+01
1244.846	9.74E+01	9.73E+01	9.83E+01
1245.328	9.74E+01	9.73E+01	9.82E+01
1245.81	9.74E+01	9.73E+01	9.82E+01
1246.292	9.74E+01	9.73E+01	9.82E+01
1246.774	9.74E+01	9.73E+01	9.82E+01
1247.256	9.74E+01	9.73E+01	9.82E+01
1247.739	9.74E+01	9.73E+01	9.82E+01
1248.221	9.74E+01	9.73E+01	9.82E+01
1248.703	9.74E+01	9.73E+01	9.82E+01
1249.185	9.74E+01	9.73E+01	9.82E+01
1249.667	9.74E+01	9.73E+01	9.82E+01
1250.149	9.74E+01	9.73E+01	9.82E+01
1250.631	9.74E+01	9.73E+01	9.82E+01
1251.113	9.74E+01	9.73E+01	9.82E+01
1251.595	9.74E+01	9.73E+01	9.82E+01
1252.078	9.74E+01	9.73E+01	9.82E+01
1252.56	9.74E+01	9.73E+01	9.82E+01
1253.042	9.74E+01	9.73E+01	9.82E+01
1253.524	9.74E+01	9.73E+01	9.82E+01
1254.006	9.74E+01	9.74E+01	9.82E+01
1254.488	9.74E+01	9.74E+01	9.82E+01
1254.97	9.74E+01	9.74E+01	9.82E+01
1255.453	9.74E+01	9.74E+01	9.82E+01
1255.935	9.74E+01	9.74E+01	9.82E+01
1256.417	9.74E+01	9.74E+01	9.82E+01
1256.899	9.75E+01	9.75E+01	9.83E+01
1257.381	9.75E+01	9.75E+01	9.83E+01
1257.863	9.75E+01	9.75E+01	9.83E+01
1258.345	9.75E+01	9.75E+01	9.83E+01
1258.827	9.75E+01	9.75E+01	9.83E+01
1259.309	9.75E+01	9.75E+01	9.83E+01
1259.792	9.75E+01	9.75E+01	9.83E+01

1260.274	9.75E+01	9.75E+01	9.83E+01
1260.756	9.75E+01	9.74E+01	9.83E+01
1261.238	9.75E+01	9.74E+01	9.83E+01
1261.72	9.75E+01	9.74E+01	9.83E+01
1262.202	9.75E+01	9.74E+01	9.83E+01
1262.684	9.76E+01	9.74E+01	9.83E+01
1263.167	9.76E+01	9.74E+01	9.84E+01
1263.649	9.76E+01	9.75E+01	9.84E+01
1264.131	9.76E+01	9.75E+01	9.84E+01
1264.613	9.76E+01	9.75E+01	9.84E+01
1265.095	9.76E+01	9.75E+01	9.83E+01
1265.577	9.76E+01	9.75E+01	9.83E+01
1266.059	9.76E+01	9.75E+01	9.83E+01
1266.541	9.76E+01	9.75E+01	9.83E+01
1267.023	9.76E+01	9.75E+01	9.83E+01
1267.506	9.76E+01	9.75E+01	9.83E+01
1267.988	9.75E+01	9.75E+01	9.83E+01
1268.47	9.75E+01	9.75E+01	9.83E+01
1268.952	9.75E+01	9.75E+01	9.83E+01
1269.434	9.76E+01	9.75E+01	9.83E+01
1269.916	9.76E+01	9.75E+01	9.83E+01
1270.398	9.76E+01	9.75E+01	9.83E+01
1270.88	9.76E+01	9.75E+01	9.84E+01
1271.363	9.76E+01	9.75E+01	9.84E+01
1271.845	9.76E+01	9.76E+01	9.84E+01
1272.327	9.76E+01	9.76E+01	9.84E+01
1272.809	9.76E+01	9.76E+01	9.84E+01
1273.291	9.76E+01	9.76E+01	9.84E+01
1273.773	9.76E+01	9.76E+01	9.84E+01
1274.255	9.77E+01	9.76E+01	9.84E+01
1274.737	9.77E+01	9.76E+01	9.84E+01
1275.22	9.77E+01	9.76E+01	9.84E+01
1275.702	9.77E+01	9.76E+01	9.84E+01
1276.184	9.77E+01	9.76E+01	9.83E+01
1276.666	9.77E+01	9.76E+01	9.83E+01
1277.148	9.77E+01	9.76E+01	9.83E+01
1277.63	9.77E+01	9.76E+01	9.83E+01
1278.112	9.77E+01	9.76E+01	9.83E+01
1278.594	9.77E+01	9.76E+01	9.83E+01
1279.077	9.77E+01	9.76E+01	9.84E+01
1279.559	9.77E+01	9.76E+01	9.84E+01
1280.041	9.77E+01	9.76E+01	9.84E+01
1280.523	9.78E+01	9.76E+01	9.84E+01
1281.005	9.78E+01	9.77E+01	9.84E+01
1281.487	9.78E+01	9.77E+01	9.84E+01
1281.969	9.78E+01	9.77E+01	9.84E+01
1282.451	9.78E+01	9.77E+01	9.84E+01

1282.934	9.78E+01	9.77E+01	9.84E+01
1283.416	9.78E+01	9.77E+01	9.84E+01
1283.898	9.78E+01	9.77E+01	9.84E+01
1284.38	9.78E+01	9.77E+01	9.84E+01
1284.862	9.78E+01	9.77E+01	9.84E+01
1285.344	9.78E+01	9.77E+01	9.84E+01
1285.826	9.78E+01	9.77E+01	9.84E+01
1286.308	9.78E+01	9.77E+01	9.84E+01
1286.791	9.78E+01	9.77E+01	9.84E+01
1287.273	9.78E+01	9.77E+01	9.84E+01
1287.755	9.78E+01	9.77E+01	9.84E+01
1288.237	9.78E+01	9.77E+01	9.84E+01
1288.719	9.78E+01	9.77E+01	9.84E+01
1289.201	9.78E+01	9.77E+01	9.84E+01
1289.683	9.79E+01	9.77E+01	9.85E+01
1290.165	9.79E+01	9.77E+01	9.85E+01
1290.648	9.79E+01	9.77E+01	9.85E+01
1291.13	9.79E+01	9.77E+01	9.85E+01
1291.612	9.79E+01	9.77E+01	9.85E+01
1292.094	9.79E+01	9.77E+01	9.85E+01
1292.576	9.79E+01	9.77E+01	9.85E+01
1293.058	9.79E+01	9.77E+01	9.85E+01
1293.54	9.79E+01	9.77E+01	9.85E+01
1294.022	9.79E+01	9.77E+01	9.84E+01
1294.505	9.79E+01	9.77E+01	9.84E+01
1294.987	9.79E+01	9.77E+01	9.85E+01
1295.469	9.79E+01	9.77E+01	9.85E+01
1295.951	9.79E+01	9.77E+01	9.85E+01
1296.433	9.79E+01	9.77E+01	9.85E+01
1296.915	9.79E+01	9.77E+01	9.85E+01
1297.397	9.79E+01	9.77E+01	9.85E+01
1297.88	9.79E+01	9.77E+01	9.85E+01
1298.362	9.79E+01	9.77E+01	9.85E+01
1298.844	9.79E+01	9.77E+01	9.85E+01
1299.326	9.79E+01	9.77E+01	9.85E+01
1299.808	9.79E+01	9.77E+01	9.85E+01
1300.29	9.79E+01	9.77E+01	9.85E+01
1300.772	9.79E+01	9.77E+01	9.85E+01
1301.254	9.79E+01	9.77E+01	9.85E+01
1301.736	9.79E+01	9.77E+01	9.85E+01
1302.219	9.79E+01	9.77E+01	9.85E+01
1302.701	9.79E+01	9.77E+01	9.85E+01
1303.183	9.79E+01	9.77E+01	9.85E+01
1303.665	9.79E+01	9.77E+01	9.85E+01
1304.147	9.79E+01	9.77E+01	9.85E+01
1304.629	9.79E+01	9.77E+01	9.85E+01
1305.111	9.79E+01	9.77E+01	9.85E+01

1305.594	9.79E+01	9.77E+01	9.85E+01
1306.076	9.79E+01	9.77E+01	9.85E+01
1306.558	9.79E+01	9.77E+01	9.85E+01
1307.04	9.79E+01	9.77E+01	9.84E+01
1307.522	9.79E+01	9.77E+01	9.84E+01
1308.004	9.79E+01	9.77E+01	9.84E+01
1308.486	9.79E+01	9.77E+01	9.84E+01
1308.968	9.79E+01	9.76E+01	9.84E+01
1309.45	9.79E+01	9.76E+01	9.84E+01
1309.933	9.78E+01	9.76E+01	9.84E+01
1310.415	9.78E+01	9.76E+01	9.84E+01
1310.897	9.78E+01	9.76E+01	9.84E+01
1311.379	9.78E+01	9.76E+01	9.84E+01
1311.861	9.77E+01	9.76E+01	9.84E+01
1312.343	9.77E+01	9.76E+01	9.84E+01
1312.825	9.77E+01	9.76E+01	9.84E+01
1313.307	9.77E+01	9.76E+01	9.84E+01
1313.79	9.77E+01	9.75E+01	9.84E+01
1314.272	9.77E+01	9.75E+01	9.84E+01
1314.754	9.77E+01	9.75E+01	9.84E+01
1315.236	9.77E+01	9.75E+01	9.83E+01
1315.718	9.77E+01	9.75E+01	9.83E+01
1316.2	9.77E+01	9.75E+01	9.83E+01
1316.682	9.76E+01	9.75E+01	9.83E+01
1317.164	9.76E+01	9.75E+01	9.83E+01
1317.647	9.76E+01	9.75E+01	9.83E+01
1318.129	9.76E+01	9.75E+01	9.83E+01
1318.611	9.76E+01	9.75E+01	9.83E+01
1319.093	9.76E+01	9.75E+01	9.83E+01
1319.575	9.76E+01	9.75E+01	9.83E+01
1320.057	9.76E+01	9.75E+01	9.83E+01
1320.539	9.76E+01	9.75E+01	9.83E+01
1321.021	9.76E+01	9.75E+01	9.83E+01
1321.504	9.77E+01	9.75E+01	9.83E+01
1321.986	9.77E+01	9.75E+01	9.83E+01
1322.468	9.77E+01	9.75E+01	9.84E+01
1322.95	9.77E+01	9.75E+01	9.84E+01
1323.432	9.77E+01	9.75E+01	9.84E+01
1323.914	9.78E+01	9.75E+01	9.84E+01
1324.396	9.78E+01	9.76E+01	9.84E+01
1324.878	9.78E+01	9.76E+01	9.84E+01
1325.361	9.78E+01	9.76E+01	9.84E+01
1325.843	9.78E+01	9.76E+01	9.84E+01
1326.325	9.78E+01	9.76E+01	9.84E+01
1326.807	9.78E+01	9.76E+01	9.84E+01
1327.289	9.78E+01	9.76E+01	9.84E+01
1327.771	9.77E+01	9.75E+01	9.84E+01

1328.253	9.77E+01	9.75E+01	9.84E+01
1328.735	9.77E+01	9.75E+01	9.84E+01
1329.218	9.77E+01	9.75E+01	9.83E+01
1329.7	9.77E+01	9.75E+01	9.83E+01
1330.182	9.77E+01	9.75E+01	9.83E+01
1330.664	9.77E+01	9.75E+01	9.83E+01
1331.146	9.77E+01	9.75E+01	9.83E+01
1331.628	9.77E+01	9.75E+01	9.83E+01
1332.11	9.77E+01	9.75E+01	9.83E+01
1332.592	9.77E+01	9.75E+01	9.83E+01
1333.075	9.77E+01	9.75E+01	9.83E+01
1333.557	9.77E+01	9.75E+01	9.83E+01
1334.039	9.77E+01	9.75E+01	9.83E+01
1334.521	9.76E+01	9.75E+01	9.83E+01
1335.003	9.76E+01	9.75E+01	9.82E+01
1335.485	9.75E+01	9.74E+01	9.82E+01
1335.967	9.75E+01	9.74E+01	9.82E+01
1336.449	9.74E+01	9.74E+01	9.82E+01
1336.932	9.74E+01	9.73E+01	9.82E+01
1337.414	9.73E+01	9.73E+01	9.81E+01
1337.896	9.72E+01	9.73E+01	9.81E+01
1338.378	9.72E+01	9.72E+01	9.81E+01
1338.86	9.71E+01	9.72E+01	9.81E+01
1339.342	9.71E+01	9.72E+01	9.81E+01
1339.824	9.71E+01	9.72E+01	9.81E+01
1340.306	9.72E+01	9.72E+01	9.81E+01
1340.789	9.72E+01	9.72E+01	9.81E+01
1341.271	9.73E+01	9.72E+01	9.81E+01
1341.753	9.74E+01	9.72E+01	9.82E+01
1342.235	9.74E+01	9.72E+01	9.82E+01
1342.717	9.75E+01	9.72E+01	9.82E+01
1343.199	9.75E+01	9.72E+01	9.82E+01
1343.681	9.75E+01	9.72E+01	9.82E+01
1344.163	9.75E+01	9.72E+01	9.82E+01
1344.646	9.75E+01	9.72E+01	9.82E+01
1345.128	9.75E+01	9.72E+01	9.82E+01
1345.61	9.74E+01	9.72E+01	9.81E+01
1346.092	9.74E+01	9.72E+01	9.81E+01
1346.574	9.74E+01	9.72E+01	9.81E+01
1347.056	9.73E+01	9.71E+01	9.81E+01
1347.538	9.73E+01	9.71E+01	9.81E+01
1348.02	9.73E+01	9.71E+01	9.81E+01
1348.503	9.73E+01	9.71E+01	9.81E+01
1348.985	9.72E+01	9.71E+01	9.81E+01
1349.467	9.72E+01	9.71E+01	9.81E+01
1349.949	9.72E+01	9.70E+01	9.81E+01
1350.431	9.73E+01	9.70E+01	9.81E+01

1350.913	9.73E+01	9.70E+01	9.81E+01
1351.395	9.73E+01	9.70E+01	9.81E+01
1351.877	9.72E+01	9.70E+01	9.81E+01
1352.359	9.72E+01	9.70E+01	9.81E+01
1352.842	9.72E+01	9.70E+01	9.81E+01
1353.324	9.72E+01	9.70E+01	9.81E+01
1353.806	9.72E+01	9.70E+01	9.80E+01
1354.288	9.72E+01	9.70E+01	9.80E+01
1354.77	9.72E+01	9.70E+01	9.80E+01
1355.252	9.72E+01	9.71E+01	9.80E+01
1355.734	9.72E+01	9.71E+01	9.80E+01
1356.217	9.72E+01	9.71E+01	9.80E+01
1356.699	9.72E+01	9.70E+01	9.80E+01
1357.181	9.72E+01	9.70E+01	9.80E+01
1357.663	9.72E+01	9.70E+01	9.80E+01
1358.145	9.72E+01	9.70E+01	9.80E+01
1358.627	9.72E+01	9.70E+01	9.80E+01
1359.109	9.72E+01	9.70E+01	9.80E+01
1359.591	9.72E+01	9.70E+01	9.80E+01
1360.073	9.71E+01	9.70E+01	9.80E+01
1360.556	9.71E+01	9.70E+01	9.80E+01
1361.038	9.71E+01	9.70E+01	9.80E+01
1361.52	9.70E+01	9.69E+01	9.80E+01
1362.002	9.70E+01	9.69E+01	9.79E+01
1362.484	9.70E+01	9.69E+01	9.79E+01
1362.966	9.70E+01	9.69E+01	9.79E+01
1363.448	9.70E+01	9.69E+01	9.79E+01
1363.931	9.71E+01	9.70E+01	9.80E+01
1364.413	9.71E+01	9.70E+01	9.80E+01
1364.895	9.72E+01	9.70E+01	9.80E+01
1365.377	9.72E+01	9.71E+01	9.80E+01
1365.859	9.73E+01	9.71E+01	9.81E+01
1366.341	9.73E+01	9.71E+01	9.81E+01
1366.823	9.73E+01	9.71E+01	9.81E+01
1367.305	9.73E+01	9.71E+01	9.81E+01
1367.787	9.73E+01	9.71E+01	9.81E+01
1368.27	9.73E+01	9.71E+01	9.81E+01
1368.752	9.73E+01	9.71E+01	9.81E+01
1369.234	9.74E+01	9.71E+01	9.81E+01
1369.716	9.74E+01	9.71E+01	9.81E+01
1370.198	9.74E+01	9.71E+01	9.81E+01
1370.68	9.74E+01	9.71E+01	9.81E+01
1371.162	9.74E+01	9.71E+01	9.81E+01
1371.645	9.74E+01	9.71E+01	9.81E+01
1372.127	9.73E+01	9.71E+01	9.81E+01
1372.609	9.73E+01	9.71E+01	9.81E+01
1373.091	9.73E+01	9.71E+01	9.81E+01

1373.573	9.72E+01	9.71E+01	9.81E+01
1374.055	9.72E+01	9.72E+01	9.81E+01
1374.537	9.72E+01	9.72E+01	9.81E+01
1375.019	9.73E+01	9.72E+01	9.81E+01
1375.501	9.73E+01	9.72E+01	9.81E+01
1375.984	9.74E+01	9.72E+01	9.81E+01
1376.466	9.74E+01	9.72E+01	9.82E+01
1376.948	9.74E+01	9.73E+01	9.82E+01
1377.43	9.75E+01	9.73E+01	9.82E+01
1377.912	9.75E+01	9.73E+01	9.82E+01
1378.394	9.75E+01	9.73E+01	9.82E+01
1378.876	9.75E+01	9.73E+01	9.82E+01
1379.359	9.75E+01	9.74E+01	9.82E+01
1379.841	9.76E+01	9.74E+01	9.82E+01
1380.323	9.76E+01	9.74E+01	9.82E+01
1380.805	9.76E+01	9.74E+01	9.83E+01
1381.287	9.77E+01	9.74E+01	9.83E+01
1381.769	9.77E+01	9.74E+01	9.83E+01
1382.251	9.77E+01	9.74E+01	9.83E+01
1382.733	9.77E+01	9.74E+01	9.83E+01
1383.215	9.77E+01	9.75E+01	9.83E+01
1383.698	9.77E+01	9.75E+01	9.83E+01
1384.18	9.77E+01	9.75E+01	9.83E+01
1384.662	9.76E+01	9.75E+01	9.83E+01
1385.144	9.76E+01	9.75E+01	9.83E+01
1385.626	9.75E+01	9.75E+01	9.83E+01
1386.108	9.75E+01	9.75E+01	9.83E+01
1386.59	9.75E+01	9.75E+01	9.83E+01
1387.073	9.75E+01	9.75E+01	9.83E+01
1387.555	9.75E+01	9.75E+01	9.83E+01
1388.037	9.75E+01	9.75E+01	9.83E+01
1388.519	9.76E+01	9.75E+01	9.83E+01
1389.001	9.76E+01	9.75E+01	9.83E+01
1389.483	9.77E+01	9.75E+01	9.83E+01
1389.965	9.77E+01	9.75E+01	9.83E+01
1390.447	9.77E+01	9.75E+01	9.84E+01
1390.93	9.78E+01	9.75E+01	9.84E+01
1391.412	9.78E+01	9.75E+01	9.84E+01
1391.894	9.78E+01	9.75E+01	9.84E+01
1392.376	9.77E+01	9.75E+01	9.84E+01
1392.858	9.77E+01	9.75E+01	9.84E+01
1393.34	9.76E+01	9.75E+01	9.83E+01
1393.822	9.76E+01	9.75E+01	9.83E+01
1394.304	9.75E+01	9.75E+01	9.83E+01
1394.786	9.75E+01	9.75E+01	9.83E+01
1395.269	9.74E+01	9.75E+01	9.83E+01
1395.751	9.74E+01	9.75E+01	9.83E+01

1396.233	9.74E+01	9.75E+01	9.83E+01
1396.715	9.74E+01	9.75E+01	9.83E+01
1397.197	9.74E+01	9.75E+01	9.82E+01
1397.679	9.75E+01	9.75E+01	9.82E+01
1398.161	9.75E+01	9.75E+01	9.82E+01
1398.644	9.75E+01	9.75E+01	9.82E+01
1399.126	9.76E+01	9.76E+01	9.82E+01
1399.608	9.76E+01	9.76E+01	9.83E+01
1400.09	9.77E+01	9.76E+01	9.83E+01
1400.572	9.78E+01	9.76E+01	9.84E+01
1401.054	9.79E+01	9.77E+01	9.84E+01
1401.536	9.79E+01	9.77E+01	9.85E+01
1402.018	9.79E+01	9.77E+01	9.85E+01
1402.5	9.79E+01	9.77E+01	9.85E+01
1402.983	9.79E+01	9.76E+01	9.85E+01
1403.465	9.79E+01	9.76E+01	9.85E+01
1403.947	9.79E+01	9.76E+01	9.84E+01
1404.429	9.78E+01	9.76E+01	9.84E+01
1404.911	9.78E+01	9.76E+01	9.84E+01
1405.393	9.78E+01	9.76E+01	9.84E+01
1405.875	9.78E+01	9.76E+01	9.84E+01
1406.358	9.79E+01	9.76E+01	9.84E+01
1406.84	9.79E+01	9.76E+01	9.84E+01
1407.322	9.79E+01	9.76E+01	9.85E+01
1407.804	9.79E+01	9.76E+01	9.85E+01
1408.286	9.79E+01	9.76E+01	9.85E+01
1408.768	9.79E+01	9.76E+01	9.85E+01
1409.25	9.79E+01	9.76E+01	9.84E+01
1409.732	9.78E+01	9.76E+01	9.84E+01
1410.214	9.78E+01	9.76E+01	9.84E+01
1410.697	9.78E+01	9.76E+01	9.84E+01
1411.179	9.78E+01	9.76E+01	9.84E+01
1411.661	9.78E+01	9.76E+01	9.84E+01
1412.143	9.78E+01	9.76E+01	9.85E+01
1412.625	9.79E+01	9.76E+01	9.85E+01
1413.107	9.79E+01	9.76E+01	9.84E+01
1413.589	9.78E+01	9.76E+01	9.84E+01
1414.072	9.78E+01	9.75E+01	9.84E+01
1414.554	9.78E+01	9.75E+01	9.84E+01
1415.036	9.77E+01	9.75E+01	9.84E+01
1415.518	9.76E+01	9.75E+01	9.83E+01
1416	9.75E+01	9.75E+01	9.83E+01
1416.482	9.74E+01	9.74E+01	9.83E+01
1416.964	9.73E+01	9.74E+01	9.82E+01
1417.446	9.72E+01	9.74E+01	9.82E+01
1417.928	9.72E+01	9.73E+01	9.81E+01
1418.411	9.71E+01	9.73E+01	9.81E+01

1418.893	9.71E+01	9.73E+01	9.81E+01
1419.375	9.72E+01	9.73E+01	9.80E+01
1419.857	9.73E+01	9.73E+01	9.81E+01
1420.339	9.73E+01	9.73E+01	9.81E+01
1420.821	9.74E+01	9.73E+01	9.81E+01
1421.303	9.74E+01	9.73E+01	9.81E+01
1421.786	9.74E+01	9.73E+01	9.81E+01
1422.268	9.74E+01	9.73E+01	9.82E+01
1422.75	9.74E+01	9.73E+01	9.82E+01
1423.232	9.74E+01	9.73E+01	9.82E+01
1423.714	9.74E+01	9.73E+01	9.82E+01
1424.196	9.74E+01	9.73E+01	9.82E+01
1424.678	9.74E+01	9.73E+01	9.82E+01
1425.16	9.74E+01	9.72E+01	9.82E+01
1425.642	9.75E+01	9.72E+01	9.82E+01
1426.125	9.75E+01	9.72E+01	9.82E+01
1426.607	9.75E+01	9.72E+01	9.82E+01
1427.089	9.74E+01	9.72E+01	9.82E+01
1427.571	9.74E+01	9.71E+01	9.81E+01
1428.053	9.73E+01	9.71E+01	9.81E+01
1428.535	9.72E+01	9.70E+01	9.81E+01
1429.017	9.71E+01	9.70E+01	9.80E+01
1429.5	9.71E+01	9.69E+01	9.80E+01
1429.982	9.70E+01	9.68E+01	9.80E+01
1430.464	9.69E+01	9.68E+01	9.79E+01
1430.946	9.69E+01	9.67E+01	9.79E+01
1431.428	9.68E+01	9.67E+01	9.79E+01
1431.91	9.68E+01	9.67E+01	9.79E+01
1432.392	9.68E+01	9.66E+01	9.78E+01
1432.874	9.67E+01	9.66E+01	9.78E+01
1433.356	9.67E+01	9.66E+01	9.78E+01
1433.839	9.66E+01	9.66E+01	9.78E+01
1434.321	9.66E+01	9.65E+01	9.77E+01
1434.803	9.65E+01	9.65E+01	9.77E+01
1435.285	9.64E+01	9.64E+01	9.77E+01
1435.767	9.63E+01	9.63E+01	9.76E+01
1436.249	9.63E+01	9.62E+01	9.76E+01
1436.731	9.62E+01	9.61E+01	9.75E+01
1437.214	9.62E+01	9.60E+01	9.75E+01
1437.696	9.62E+01	9.60E+01	9.75E+01
1438.178	9.62E+01	9.60E+01	9.74E+01
1438.66	9.62E+01	9.59E+01	9.74E+01
1439.142	9.62E+01	9.59E+01	9.74E+01
1439.624	9.62E+01	9.59E+01	9.74E+01
1440.106	9.62E+01	9.59E+01	9.74E+01
1440.588	9.62E+01	9.58E+01	9.74E+01
1441.07	9.61E+01	9.58E+01	9.74E+01

1441.553	9.61E+01	9.58E+01	9.74E+01
1442.035	9.60E+01	9.57E+01	9.74E+01
1442.517	9.60E+01	9.57E+01	9.73E+01
1442.999	9.59E+01	9.56E+01	9.73E+01
1443.481	9.58E+01	9.55E+01	9.73E+01
1443.963	9.58E+01	9.55E+01	9.72E+01
1444.445	9.57E+01	9.54E+01	9.72E+01
1444.927	9.56E+01	9.53E+01	9.71E+01
1445.41	9.55E+01	9.53E+01	9.71E+01
1445.892	9.54E+01	9.52E+01	9.70E+01
1446.374	9.53E+01	9.51E+01	9.70E+01
1446.856	9.52E+01	9.51E+01	9.69E+01
1447.338	9.51E+01	9.50E+01	9.69E+01
1447.82	9.51E+01	9.50E+01	9.68E+01
1448.302	9.50E+01	9.49E+01	9.68E+01
1448.784	9.50E+01	9.49E+01	9.68E+01
1449.267	9.50E+01	9.48E+01	9.68E+01
1449.749	9.49E+01	9.48E+01	9.67E+01
1450.231	9.49E+01	9.48E+01	9.67E+01
1450.713	9.49E+01	9.47E+01	9.67E+01
1451.195	9.49E+01	9.47E+01	9.67E+01
1451.677	9.48E+01	9.47E+01	9.66E+01
1452.159	9.48E+01	9.47E+01	9.66E+01
1452.641	9.48E+01	9.47E+01	9.66E+01
1453.124	9.47E+01	9.47E+01	9.66E+01
1453.606	9.47E+01	9.47E+01	9.66E+01
1454.088	9.47E+01	9.47E+01	9.66E+01
1454.57	9.46E+01	9.47E+01	9.66E+01
1455.052	9.46E+01	9.47E+01	9.66E+01
1455.534	9.44E+01	9.47E+01	9.66E+01
1456.016	9.43E+01	9.48E+01	9.66E+01
1456.498	9.43E+01	9.49E+01	9.66E+01
1456.981	9.43E+01	9.50E+01	9.66E+01
1457.463	9.45E+01	9.53E+01	9.68E+01
1457.945	9.48E+01	9.55E+01	9.69E+01
1458.427	9.52E+01	9.58E+01	9.71E+01
1458.909	9.55E+01	9.60E+01	9.73E+01
1459.391	9.58E+01	9.61E+01	9.75E+01
1459.873	9.61E+01	9.63E+01	9.76E+01
1460.355	9.63E+01	9.64E+01	9.77E+01
1460.838	9.65E+01	9.66E+01	9.78E+01
1461.32	9.67E+01	9.67E+01	9.79E+01
1461.802	9.68E+01	9.68E+01	9.80E+01
1462.284	9.69E+01	9.68E+01	9.80E+01
1462.766	9.69E+01	9.69E+01	9.81E+01
1463.248	9.70E+01	9.70E+01	9.81E+01
1463.73	9.70E+01	9.71E+01	9.81E+01

1464.212	9.71E+01	9.72E+01	9.82E+01
1464.695	9.72E+01	9.73E+01	9.83E+01
1465.177	9.72E+01	9.74E+01	9.83E+01
1465.659	9.73E+01	9.74E+01	9.84E+01
1466.141	9.74E+01	9.75E+01	9.84E+01
1466.623	9.75E+01	9.76E+01	9.85E+01
1467.105	9.76E+01	9.76E+01	9.85E+01
1467.587	9.76E+01	9.76E+01	9.85E+01
1468.069	9.77E+01	9.77E+01	9.85E+01
1468.552	9.77E+01	9.77E+01	9.85E+01
1469.034	9.77E+01	9.77E+01	9.85E+01
1469.516	9.77E+01	9.77E+01	9.84E+01
1469.998	9.76E+01	9.77E+01	9.84E+01
1470.48	9.75E+01	9.77E+01	9.84E+01
1470.962	9.74E+01	9.77E+01	9.83E+01
1471.444	9.73E+01	9.76E+01	9.82E+01
1471.926	9.71E+01	9.76E+01	9.82E+01
1472.409	9.71E+01	9.76E+01	9.81E+01
1472.891	9.70E+01	9.76E+01	9.81E+01
1473.373	9.71E+01	9.76E+01	9.82E+01
1473.855	9.71E+01	9.77E+01	9.82E+01
1474.337	9.72E+01	9.77E+01	9.83E+01
1474.819	9.73E+01	9.78E+01	9.83E+01
1475.301	9.74E+01	9.78E+01	9.84E+01
1475.783	9.74E+01	9.78E+01	9.84E+01
1476.266	9.75E+01	9.79E+01	9.84E+01
1476.748	9.76E+01	9.79E+01	9.85E+01
1477.23	9.77E+01	9.79E+01	9.85E+01
1477.712	9.78E+01	9.79E+01	9.85E+01
1478.194	9.78E+01	9.79E+01	9.85E+01
1478.676	9.78E+01	9.79E+01	9.85E+01
1479.158	9.79E+01	9.79E+01	9.85E+01
1479.64	9.78E+01	9.79E+01	9.85E+01
1480.123	9.78E+01	9.79E+01	9.85E+01
1480.605	9.78E+01	9.79E+01	9.85E+01
1481.087	9.78E+01	9.79E+01	9.85E+01
1481.569	9.79E+01	9.80E+01	9.85E+01
1482.051	9.79E+01	9.80E+01	9.85E+01
1482.533	9.79E+01	9.80E+01	9.85E+01
1483.015	9.79E+01	9.80E+01	9.85E+01
1483.497	9.80E+01	9.80E+01	9.86E+01
1483.98	9.80E+01	9.80E+01	9.86E+01
1484.462	9.79E+01	9.80E+01	9.86E+01
1484.944	9.79E+01	9.80E+01	9.85E+01
1485.426	9.78E+01	9.80E+01	9.85E+01
1485.908	9.77E+01	9.79E+01	9.85E+01
1486.39	9.76E+01	9.79E+01	9.85E+01

1486.872	9.75E+01	9.79E+01	9.84E+01
1487.354	9.74E+01	9.78E+01	9.84E+01
1487.837	9.73E+01	9.78E+01	9.83E+01
1488.319	9.72E+01	9.78E+01	9.83E+01
1488.801	9.71E+01	9.78E+01	9.82E+01
1489.283	9.70E+01	9.78E+01	9.82E+01
1489.765	9.70E+01	9.78E+01	9.82E+01
1490.247	9.71E+01	9.78E+01	9.82E+01
1490.729	9.72E+01	9.79E+01	9.82E+01
1491.211	9.73E+01	9.79E+01	9.83E+01
1491.694	9.75E+01	9.79E+01	9.83E+01
1492.176	9.76E+01	9.79E+01	9.83E+01
1492.658	9.76E+01	9.79E+01	9.84E+01
1493.14	9.77E+01	9.79E+01	9.84E+01
1493.622	9.77E+01	9.79E+01	9.84E+01
1494.104	9.77E+01	9.79E+01	9.84E+01
1494.586	9.76E+01	9.79E+01	9.84E+01
1495.068	9.76E+01	9.79E+01	9.84E+01
1495.551	9.75E+01	9.79E+01	9.83E+01
1496.033	9.74E+01	9.79E+01	9.83E+01
1496.515	9.74E+01	9.79E+01	9.83E+01
1496.997	9.74E+01	9.79E+01	9.83E+01
1497.479	9.74E+01	9.79E+01	9.84E+01
1497.961	9.74E+01	9.79E+01	9.84E+01
1498.443	9.74E+01	9.79E+01	9.84E+01
1498.925	9.74E+01	9.79E+01	9.84E+01
1499.408	9.75E+01	9.79E+01	9.84E+01
1499.89	9.75E+01	9.79E+01	9.84E+01
1500.372	9.75E+01	9.79E+01	9.84E+01
1500.854	9.75E+01	9.79E+01	9.84E+01
1501.336	9.76E+01	9.79E+01	9.84E+01
1501.818	9.76E+01	9.79E+01	9.84E+01
1502.3	9.75E+01	9.78E+01	9.84E+01
1502.782	9.75E+01	9.78E+01	9.84E+01
1503.265	9.75E+01	9.78E+01	9.84E+01
1503.747	9.74E+01	9.78E+01	9.83E+01
1504.229	9.73E+01	9.78E+01	9.83E+01
1504.711	9.72E+01	9.77E+01	9.82E+01
1505.193	9.70E+01	9.77E+01	9.81E+01
1505.675	9.67E+01	9.76E+01	9.79E+01
1506.157	9.64E+01	9.75E+01	9.77E+01
1506.639	9.61E+01	9.74E+01	9.76E+01
1507.122	9.59E+01	9.73E+01	9.75E+01
1507.604	9.59E+01	9.74E+01	9.76E+01
1508.086	9.61E+01	9.74E+01	9.77E+01
1508.568	9.63E+01	9.75E+01	9.78E+01
1509.05	9.65E+01	9.76E+01	9.79E+01

1509.532	9.66E+01	9.76E+01	9.80E+01
1510.014	9.68E+01	9.76E+01	9.80E+01
1510.496	9.69E+01	9.77E+01	9.81E+01
1510.979	9.70E+01	9.77E+01	9.81E+01
1511.461	9.70E+01	9.77E+01	9.81E+01
1511.943	9.71E+01	9.77E+01	9.82E+01
1512.425	9.71E+01	9.77E+01	9.82E+01
1512.907	9.72E+01	9.78E+01	9.82E+01
1513.389	9.72E+01	9.78E+01	9.82E+01
1513.871	9.71E+01	9.78E+01	9.82E+01
1514.353	9.71E+01	9.78E+01	9.82E+01
1514.836	9.70E+01	9.78E+01	9.81E+01
1515.318	9.69E+01	9.78E+01	9.81E+01
1515.8	9.69E+01	9.77E+01	9.81E+01
1516.282	9.68E+01	9.77E+01	9.80E+01
1516.764	9.68E+01	9.77E+01	9.80E+01
1517.246	9.68E+01	9.76E+01	9.80E+01
1517.728	9.68E+01	9.76E+01	9.80E+01
1518.21	9.68E+01	9.76E+01	9.80E+01
1518.693	9.68E+01	9.76E+01	9.80E+01
1519.175	9.68E+01	9.76E+01	9.80E+01
1519.657	9.67E+01	9.76E+01	9.80E+01
1520.139	9.66E+01	9.76E+01	9.80E+01
1520.621	9.65E+01	9.76E+01	9.79E+01
1521.103	9.64E+01	9.76E+01	9.79E+01
1521.585	9.64E+01	9.76E+01	9.79E+01
1522.067	9.64E+01	9.77E+01	9.79E+01
1522.55	9.65E+01	9.77E+01	9.80E+01
1523.032	9.66E+01	9.77E+01	9.80E+01
1523.514	9.67E+01	9.77E+01	9.81E+01
1523.996	9.68E+01	9.77E+01	9.81E+01
1524.478	9.68E+01	9.77E+01	9.81E+01
1524.96	9.68E+01	9.77E+01	9.81E+01
1525.442	9.68E+01	9.77E+01	9.81E+01
1525.924	9.68E+01	9.77E+01	9.81E+01
1526.406	9.69E+01	9.77E+01	9.81E+01
1526.889	9.69E+01	9.77E+01	9.81E+01
1527.371	9.70E+01	9.77E+01	9.81E+01
1527.853	9.71E+01	9.77E+01	9.81E+01
1528.335	9.71E+01	9.77E+01	9.82E+01
1528.817	9.72E+01	9.77E+01	9.82E+01
1529.299	9.73E+01	9.77E+01	9.82E+01
1529.781	9.73E+01	9.77E+01	9.82E+01
1530.264	9.73E+01	9.77E+01	9.82E+01
1530.746	9.73E+01	9.77E+01	9.82E+01
1531.228	9.73E+01	9.77E+01	9.82E+01
1531.71	9.72E+01	9.76E+01	9.81E+01

1532.192	9.71E+01	9.76E+01	9.81E+01
1532.674	9.69E+01	9.76E+01	9.81E+01
1533.156	9.69E+01	9.76E+01	9.80E+01
1533.638	9.68E+01	9.76E+01	9.80E+01
1534.12	9.69E+01	9.76E+01	9.80E+01
1534.603	9.69E+01	9.76E+01	9.81E+01
1535.085	9.70E+01	9.76E+01	9.81E+01
1535.567	9.70E+01	9.76E+01	9.81E+01
1536.049	9.71E+01	9.76E+01	9.81E+01
1536.531	9.71E+01	9.76E+01	9.81E+01
1537.013	9.70E+01	9.76E+01	9.80E+01
1537.495	9.69E+01	9.76E+01	9.80E+01
1537.978	9.68E+01	9.75E+01	9.79E+01
1538.46	9.66E+01	9.75E+01	9.78E+01
1538.942	9.63E+01	9.74E+01	9.76E+01
1539.424	9.60E+01	9.73E+01	9.74E+01
1539.906	9.58E+01	9.72E+01	9.73E+01
1540.388	9.56E+01	9.71E+01	9.73E+01
1540.87	9.56E+01	9.71E+01	9.73E+01
1541.352	9.57E+01	9.72E+01	9.74E+01
1541.834	9.58E+01	9.72E+01	9.75E+01
1542.317	9.60E+01	9.73E+01	9.76E+01
1542.799	9.61E+01	9.73E+01	9.77E+01
1543.281	9.63E+01	9.74E+01	9.78E+01
1543.763	9.64E+01	9.74E+01	9.79E+01
1544.245	9.65E+01	9.74E+01	9.79E+01
1544.727	9.67E+01	9.74E+01	9.80E+01
1545.209	9.69E+01	9.75E+01	9.81E+01
1545.692	9.70E+01	9.75E+01	9.81E+01
1546.174	9.72E+01	9.75E+01	9.82E+01
1546.656	9.73E+01	9.76E+01	9.82E+01
1547.138	9.73E+01	9.76E+01	9.82E+01
1547.62	9.73E+01	9.76E+01	9.82E+01
1548.102	9.73E+01	9.76E+01	9.82E+01
1548.584	9.73E+01	9.76E+01	9.82E+01
1549.066	9.72E+01	9.76E+01	9.82E+01
1549.548	9.71E+01	9.76E+01	9.82E+01
1550.031	9.71E+01	9.75E+01	9.82E+01
1550.513	9.71E+01	9.75E+01	9.82E+01
1550.995	9.71E+01	9.75E+01	9.82E+01
1551.477	9.72E+01	9.76E+01	9.82E+01
1551.959	9.72E+01	9.76E+01	9.82E+01
1552.441	9.72E+01	9.76E+01	9.82E+01
1552.923	9.72E+01	9.76E+01	9.82E+01
1553.406	9.71E+01	9.76E+01	9.82E+01
1553.888	9.71E+01	9.76E+01	9.81E+01
1554.37	9.70E+01	9.76E+01	9.81E+01

1554.852	9.70E+01	9.75E+01	9.81E+01
1555.334	9.69E+01	9.75E+01	9.81E+01
1555.816	9.69E+01	9.75E+01	9.80E+01
1556.298	9.68E+01	9.75E+01	9.80E+01
1556.78	9.67E+01	9.75E+01	9.80E+01
1557.262	9.66E+01	9.75E+01	9.80E+01
1557.745	9.64E+01	9.74E+01	9.79E+01
1558.227	9.62E+01	9.74E+01	9.78E+01
1558.709	9.61E+01	9.73E+01	9.78E+01
1559.191	9.63E+01	9.73E+01	9.78E+01
1559.673	9.65E+01	9.73E+01	9.79E+01
1560.155	9.67E+01	9.73E+01	9.80E+01
1560.637	9.69E+01	9.74E+01	9.80E+01
1561.12	9.71E+01	9.74E+01	9.81E+01
1561.602	9.72E+01	9.75E+01	9.81E+01
1562.084	9.72E+01	9.75E+01	9.82E+01
1562.566	9.73E+01	9.75E+01	9.82E+01
1563.048	9.73E+01	9.75E+01	9.82E+01
1563.53	9.73E+01	9.75E+01	9.82E+01
1564.012	9.73E+01	9.75E+01	9.82E+01
1564.494	9.73E+01	9.75E+01	9.82E+01
1564.976	9.73E+01	9.75E+01	9.82E+01
1565.459	9.73E+01	9.75E+01	9.82E+01
1565.941	9.73E+01	9.74E+01	9.82E+01
1566.423	9.73E+01	9.74E+01	9.81E+01
1566.905	9.73E+01	9.74E+01	9.81E+01
1567.387	9.73E+01	9.74E+01	9.81E+01
1567.869	9.72E+01	9.74E+01	9.81E+01
1568.351	9.72E+01	9.74E+01	9.81E+01
1568.833	9.71E+01	9.75E+01	9.81E+01
1569.316	9.71E+01	9.75E+01	9.81E+01
1569.798	9.72E+01	9.75E+01	9.81E+01
1570.28	9.72E+01	9.76E+01	9.81E+01
1570.762	9.73E+01	9.76E+01	9.82E+01
1571.244	9.74E+01	9.76E+01	9.82E+01
1571.726	9.74E+01	9.76E+01	9.83E+01
1572.208	9.75E+01	9.76E+01	9.83E+01
1572.69	9.75E+01	9.76E+01	9.83E+01
1573.173	9.75E+01	9.76E+01	9.83E+01
1573.655	9.74E+01	9.76E+01	9.83E+01
1574.137	9.74E+01	9.75E+01	9.83E+01
1574.619	9.73E+01	9.75E+01	9.82E+01
1575.101	9.72E+01	9.75E+01	9.82E+01
1575.583	9.71E+01	9.74E+01	9.81E+01
1576.065	9.70E+01	9.74E+01	9.81E+01
1576.547	9.70E+01	9.74E+01	9.81E+01
1577.03	9.71E+01	9.73E+01	9.81E+01

1577.512	9.71E+01	9.74E+01	9.81E+01
1577.994	9.72E+01	9.74E+01	9.81E+01
1578.476	9.73E+01	9.74E+01	9.81E+01
1578.958	9.74E+01	9.74E+01	9.82E+01
1579.44	9.75E+01	9.75E+01	9.82E+01
1579.922	9.75E+01	9.75E+01	9.82E+01
1580.404	9.75E+01	9.75E+01	9.82E+01
1580.887	9.76E+01	9.75E+01	9.82E+01
1581.369	9.76E+01	9.75E+01	9.82E+01
1581.851	9.76E+01	9.75E+01	9.83E+01
1582.333	9.76E+01	9.75E+01	9.83E+01
1582.815	9.76E+01	9.75E+01	9.83E+01
1583.297	9.76E+01	9.75E+01	9.83E+01
1583.779	9.76E+01	9.75E+01	9.82E+01
1584.261	9.76E+01	9.75E+01	9.82E+01
1584.744	9.76E+01	9.75E+01	9.82E+01
1585.226	9.76E+01	9.75E+01	9.82E+01
1585.708	9.76E+01	9.75E+01	9.82E+01
1586.19	9.76E+01	9.74E+01	9.82E+01
1586.672	9.76E+01	9.74E+01	9.82E+01
1587.154	9.76E+01	9.74E+01	9.82E+01
1587.636	9.76E+01	9.74E+01	9.82E+01
1588.118	9.76E+01	9.74E+01	9.82E+01
1588.601	9.76E+01	9.74E+01	9.82E+01
1589.083	9.76E+01	9.74E+01	9.82E+01
1589.565	9.76E+01	9.74E+01	9.82E+01
1590.047	9.75E+01	9.74E+01	9.82E+01
1590.529	9.75E+01	9.73E+01	9.81E+01
1591.011	9.75E+01	9.73E+01	9.81E+01
1591.493	9.75E+01	9.73E+01	9.81E+01
1591.975	9.75E+01	9.73E+01	9.81E+01
1592.458	9.75E+01	9.73E+01	9.81E+01
1592.94	9.74E+01	9.73E+01	9.81E+01
1593.422	9.74E+01	9.73E+01	9.81E+01
1593.904	9.74E+01	9.73E+01	9.81E+01
1594.386	9.74E+01	9.73E+01	9.81E+01
1594.868	9.74E+01	9.73E+01	9.81E+01
1595.35	9.74E+01	9.73E+01	9.81E+01
1595.833	9.74E+01	9.73E+01	9.82E+01
1596.315	9.75E+01	9.72E+01	9.82E+01
1596.797	9.75E+01	9.72E+01	9.82E+01
1597.279	9.75E+01	9.72E+01	9.82E+01
1597.761	9.75E+01	9.72E+01	9.82E+01
1598.243	9.75E+01	9.72E+01	9.82E+01
1598.725	9.75E+01	9.72E+01	9.81E+01
1599.207	9.75E+01	9.72E+01	9.81E+01
1599.689	9.75E+01	9.72E+01	9.81E+01

1600.172	9.75E+01	9.72E+01	9.81E+01
1600.654	9.75E+01	9.72E+01	9.81E+01
1601.136	9.74E+01	9.72E+01	9.81E+01
1601.618	9.74E+01	9.72E+01	9.81E+01
1602.1	9.74E+01	9.72E+01	9.81E+01
1602.582	9.74E+01	9.72E+01	9.81E+01
1603.064	9.74E+01	9.72E+01	9.81E+01
1603.547	9.74E+01	9.72E+01	9.81E+01
1604.029	9.74E+01	9.72E+01	9.81E+01
1604.511	9.74E+01	9.72E+01	9.81E+01
1604.993	9.74E+01	9.72E+01	9.81E+01
1605.475	9.74E+01	9.72E+01	9.81E+01
1605.957	9.73E+01	9.72E+01	9.81E+01
1606.439	9.73E+01	9.72E+01	9.81E+01
1606.921	9.73E+01	9.72E+01	9.80E+01
1607.403	9.72E+01	9.72E+01	9.80E+01
1607.886	9.72E+01	9.72E+01	9.80E+01
1608.368	9.72E+01	9.72E+01	9.80E+01
1608.85	9.72E+01	9.71E+01	9.80E+01
1609.332	9.72E+01	9.71E+01	9.80E+01
1609.814	9.72E+01	9.71E+01	9.80E+01
1610.296	9.72E+01	9.71E+01	9.80E+01
1610.778	9.73E+01	9.71E+01	9.80E+01
1611.26	9.73E+01	9.71E+01	9.80E+01
1611.743	9.73E+01	9.71E+01	9.80E+01
1612.225	9.73E+01	9.71E+01	9.80E+01
1612.707	9.73E+01	9.71E+01	9.80E+01
1613.189	9.73E+01	9.71E+01	9.80E+01
1613.671	9.72E+01	9.70E+01	9.80E+01
1614.153	9.72E+01	9.70E+01	9.80E+01
1614.635	9.71E+01	9.70E+01	9.79E+01
1615.117	9.70E+01	9.70E+01	9.79E+01
1615.6	9.69E+01	9.69E+01	9.79E+01
1616.082	9.68E+01	9.69E+01	9.79E+01
1616.564	9.68E+01	9.69E+01	9.78E+01
1617.046	9.68E+01	9.68E+01	9.79E+01
1617.528	9.68E+01	9.68E+01	9.79E+01
1618.01	9.69E+01	9.69E+01	9.79E+01
1618.492	9.69E+01	9.69E+01	9.79E+01
1618.974	9.70E+01	9.69E+01	9.79E+01
1619.457	9.70E+01	9.69E+01	9.79E+01
1619.939	9.70E+01	9.69E+01	9.79E+01
1620.421	9.70E+01	9.69E+01	9.79E+01
1620.903	9.70E+01	9.69E+01	9.79E+01
1621.385	9.69E+01	9.68E+01	9.78E+01
1621.867	9.68E+01	9.68E+01	9.78E+01
1622.349	9.67E+01	9.67E+01	9.77E+01

1622.831	9.66E+01	9.67E+01	9.77E+01
1623.314	9.66E+01	9.66E+01	9.76E+01
1623.796	9.67E+01	9.66E+01	9.77E+01
1624.278	9.67E+01	9.67E+01	9.77E+01
1624.76	9.68E+01	9.67E+01	9.77E+01
1625.242	9.68E+01	9.67E+01	9.78E+01
1625.724	9.69E+01	9.67E+01	9.78E+01
1626.206	9.69E+01	9.67E+01	9.78E+01
1626.688	9.69E+01	9.67E+01	9.78E+01
1627.171	9.69E+01	9.67E+01	9.78E+01
1627.653	9.69E+01	9.67E+01	9.78E+01
1628.135	9.69E+01	9.67E+01	9.78E+01
1628.617	9.69E+01	9.67E+01	9.78E+01
1629.099	9.70E+01	9.67E+01	9.78E+01
1629.581	9.70E+01	9.67E+01	9.78E+01
1630.063	9.71E+01	9.68E+01	9.78E+01
1630.545	9.71E+01	9.68E+01	9.78E+01
1631.028	9.71E+01	9.68E+01	9.78E+01
1631.51	9.71E+01	9.68E+01	9.78E+01
1631.992	9.71E+01	9.68E+01	9.78E+01
1632.474	9.70E+01	9.68E+01	9.77E+01
1632.956	9.70E+01	9.68E+01	9.77E+01
1633.438	9.69E+01	9.68E+01	9.77E+01
1633.92	9.68E+01	9.67E+01	9.76E+01
1634.402	9.67E+01	9.67E+01	9.76E+01
1634.885	9.65E+01	9.67E+01	9.76E+01
1635.367	9.64E+01	9.66E+01	9.76E+01
1635.849	9.63E+01	9.66E+01	9.76E+01
1636.331	9.64E+01	9.66E+01	9.76E+01
1636.813	9.64E+01	9.66E+01	9.77E+01
1637.295	9.66E+01	9.66E+01	9.77E+01
1637.777	9.67E+01	9.66E+01	9.78E+01
1638.259	9.68E+01	9.66E+01	9.78E+01
1638.742	9.69E+01	9.66E+01	9.78E+01
1639.224	9.69E+01	9.66E+01	9.78E+01
1639.706	9.70E+01	9.66E+01	9.78E+01
1640.188	9.70E+01	9.67E+01	9.78E+01
1640.67	9.71E+01	9.67E+01	9.78E+01
1641.152	9.71E+01	9.67E+01	9.78E+01
1641.634	9.71E+01	9.67E+01	9.78E+01
1642.116	9.71E+01	9.67E+01	9.78E+01
1642.599	9.71E+01	9.67E+01	9.78E+01
1643.081	9.71E+01	9.67E+01	9.78E+01
1643.563	9.70E+01	9.67E+01	9.77E+01
1644.045	9.70E+01	9.67E+01	9.77E+01
1644.527	9.69E+01	9.66E+01	9.77E+01
1645.009	9.67E+01	9.66E+01	9.76E+01

1645.491	9.66E+01	9.66E+01	9.75E+01
1645.973	9.64E+01	9.65E+01	9.74E+01
1646.456	9.62E+01	9.65E+01	9.73E+01
1646.938	9.61E+01	9.64E+01	9.73E+01
1647.42	9.61E+01	9.64E+01	9.73E+01
1647.902	9.62E+01	9.65E+01	9.73E+01
1648.384	9.63E+01	9.65E+01	9.73E+01
1648.866	9.64E+01	9.65E+01	9.74E+01
1649.348	9.64E+01	9.65E+01	9.74E+01
1649.83	9.65E+01	9.66E+01	9.74E+01
1650.313	9.65E+01	9.66E+01	9.74E+01
1650.795	9.65E+01	9.66E+01	9.74E+01
1651.277	9.64E+01	9.66E+01	9.74E+01
1651.759	9.62E+01	9.65E+01	9.73E+01
1652.241	9.60E+01	9.65E+01	9.72E+01
1652.723	9.58E+01	9.64E+01	9.71E+01
1653.205	9.57E+01	9.64E+01	9.71E+01
1653.687	9.58E+01	9.64E+01	9.71E+01
1654.17	9.60E+01	9.64E+01	9.72E+01
1654.652	9.62E+01	9.64E+01	9.73E+01
1655.134	9.64E+01	9.64E+01	9.74E+01
1655.616	9.66E+01	9.65E+01	9.75E+01
1656.098	9.68E+01	9.65E+01	9.75E+01
1656.58	9.69E+01	9.65E+01	9.76E+01
1657.062	9.70E+01	9.66E+01	9.76E+01
1657.544	9.71E+01	9.66E+01	9.76E+01
1658.026	9.71E+01	9.66E+01	9.77E+01
1658.509	9.72E+01	9.66E+01	9.77E+01
1658.991	9.72E+01	9.66E+01	9.77E+01
1659.473	9.72E+01	9.66E+01	9.77E+01
1659.955	9.71E+01	9.66E+01	9.77E+01
1660.437	9.71E+01	9.66E+01	9.77E+01
1660.919	9.70E+01	9.66E+01	9.77E+01
1661.401	9.70E+01	9.66E+01	9.77E+01
1661.884	9.69E+01	9.66E+01	9.77E+01
1662.366	9.69E+01	9.66E+01	9.77E+01
1662.848	9.69E+01	9.66E+01	9.77E+01
1663.33	9.69E+01	9.67E+01	9.77E+01
1663.812	9.70E+01	9.67E+01	9.77E+01
1664.294	9.71E+01	9.67E+01	9.78E+01
1664.776	9.71E+01	9.67E+01	9.78E+01
1665.258	9.72E+01	9.67E+01	9.78E+01
1665.74	9.72E+01	9.67E+01	9.78E+01
1666.223	9.71E+01	9.66E+01	9.78E+01
1666.705	9.71E+01	9.66E+01	9.77E+01
1667.187	9.69E+01	9.66E+01	9.77E+01
1667.669	9.68E+01	9.65E+01	9.76E+01

1668.151	9.67E+01	9.65E+01	9.75E+01
1668.633	9.65E+01	9.65E+01	9.74E+01
1669.115	9.64E+01	9.65E+01	9.74E+01
1669.598	9.63E+01	9.65E+01	9.73E+01
1670.08	9.63E+01	9.65E+01	9.73E+01
1670.562	9.63E+01	9.66E+01	9.74E+01
1671.044	9.64E+01	9.66E+01	9.74E+01
1671.526	9.64E+01	9.66E+01	9.75E+01
1672.008	9.65E+01	9.66E+01	9.75E+01
1672.49	9.66E+01	9.67E+01	9.76E+01
1672.972	9.66E+01	9.67E+01	9.76E+01
1673.454	9.66E+01	9.67E+01	9.77E+01
1673.937	9.67E+01	9.67E+01	9.77E+01
1674.419	9.67E+01	9.67E+01	9.77E+01
1674.901	9.67E+01	9.68E+01	9.78E+01
1675.383	9.68E+01	9.68E+01	9.78E+01
1675.865	9.69E+01	9.69E+01	9.79E+01
1676.347	9.69E+01	9.69E+01	9.79E+01
1676.829	9.70E+01	9.70E+01	9.79E+01
1677.312	9.70E+01	9.70E+01	9.79E+01
1677.794	9.70E+01	9.70E+01	9.79E+01
1678.276	9.70E+01	9.70E+01	9.79E+01
1678.758	9.69E+01	9.70E+01	9.78E+01
1679.24	9.69E+01	9.70E+01	9.78E+01
1679.722	9.68E+01	9.70E+01	9.78E+01
1680.204	9.68E+01	9.69E+01	9.77E+01
1680.686	9.67E+01	9.69E+01	9.77E+01
1681.168	9.66E+01	9.69E+01	9.77E+01
1681.651	9.65E+01	9.69E+01	9.76E+01
1682.133	9.64E+01	9.69E+01	9.76E+01
1682.615	9.63E+01	9.68E+01	9.75E+01
1683.097	9.61E+01	9.68E+01	9.75E+01
1683.579	9.60E+01	9.68E+01	9.75E+01
1684.061	9.59E+01	9.68E+01	9.74E+01
1684.543	9.60E+01	9.68E+01	9.75E+01
1685.026	9.61E+01	9.68E+01	9.75E+01
1685.508	9.63E+01	9.69E+01	9.76E+01
1685.99	9.64E+01	9.69E+01	9.76E+01
1686.472	9.65E+01	9.69E+01	9.76E+01
1686.954	9.65E+01	9.70E+01	9.77E+01
1687.436	9.66E+01	9.70E+01	9.77E+01
1687.918	9.66E+01	9.70E+01	9.77E+01
1688.4	9.66E+01	9.70E+01	9.77E+01
1688.883	9.67E+01	9.71E+01	9.77E+01
1689.365	9.67E+01	9.71E+01	9.78E+01
1689.847	9.68E+01	9.71E+01	9.78E+01
1690.329	9.69E+01	9.71E+01	9.79E+01

1690.811	9.70E+01	9.72E+01	9.79E+01
1691.293	9.70E+01	9.72E+01	9.79E+01
1691.775	9.71E+01	9.72E+01	9.80E+01
1692.257	9.71E+01	9.72E+01	9.80E+01
1692.74	9.71E+01	9.72E+01	9.80E+01
1693.222	9.70E+01	9.72E+01	9.80E+01
1693.704	9.70E+01	9.72E+01	9.79E+01
1694.186	9.68E+01	9.71E+01	9.79E+01
1694.668	9.67E+01	9.70E+01	9.79E+01
1695.15	9.65E+01	9.69E+01	9.78E+01
1695.632	9.63E+01	9.68E+01	9.77E+01
1696.114	9.62E+01	9.68E+01	9.77E+01
1696.597	9.61E+01	9.68E+01	9.76E+01
1697.079	9.60E+01	9.68E+01	9.76E+01
1697.561	9.60E+01	9.68E+01	9.76E+01
1698.043	9.61E+01	9.69E+01	9.77E+01
1698.525	9.61E+01	9.70E+01	9.77E+01
1699.007	9.62E+01	9.71E+01	9.77E+01
1699.489	9.63E+01	9.71E+01	9.77E+01
1699.971	9.63E+01	9.72E+01	9.77E+01
1700.453	9.64E+01	9.72E+01	9.77E+01
1700.936	9.65E+01	9.72E+01	9.77E+01
1701.418	9.65E+01	9.72E+01	9.77E+01
1701.9	9.65E+01	9.72E+01	9.77E+01
1702.382	9.65E+01	9.72E+01	9.77E+01
1702.864	9.65E+01	9.71E+01	9.77E+01
1703.346	9.64E+01	9.71E+01	9.77E+01
1703.828	9.64E+01	9.71E+01	9.77E+01
1704.311	9.63E+01	9.70E+01	9.77E+01
1704.793	9.63E+01	9.70E+01	9.77E+01
1705.275	9.62E+01	9.69E+01	9.76E+01
1705.757	9.62E+01	9.69E+01	9.76E+01
1706.239	9.63E+01	9.69E+01	9.76E+01
1706.721	9.63E+01	9.69E+01	9.77E+01
1707.203	9.64E+01	9.69E+01	9.77E+01
1707.685	9.65E+01	9.69E+01	9.77E+01
1708.167	9.66E+01	9.70E+01	9.77E+01
1708.65	9.66E+01	9.70E+01	9.77E+01
1709.132	9.66E+01	9.70E+01	9.78E+01
1709.614	9.67E+01	9.70E+01	9.78E+01
1710.096	9.67E+01	9.70E+01	9.77E+01
1710.578	9.66E+01	9.69E+01	9.77E+01
1711.06	9.66E+01	9.69E+01	9.77E+01
1711.542	9.66E+01	9.69E+01	9.77E+01
1712.025	9.65E+01	9.68E+01	9.77E+01
1712.507	9.64E+01	9.68E+01	9.76E+01
1712.989	9.63E+01	9.67E+01	9.76E+01

1713.471	9.62E+01	9.67E+01	9.75E+01
1713.953	9.61E+01	9.66E+01	9.74E+01
1714.435	9.59E+01	9.66E+01	9.73E+01
1714.917	9.58E+01	9.65E+01	9.73E+01
1715.399	9.56E+01	9.65E+01	9.72E+01
1715.881	9.55E+01	9.65E+01	9.72E+01
1716.364	9.54E+01	9.64E+01	9.71E+01
1716.846	9.53E+01	9.63E+01	9.71E+01
1717.328	9.52E+01	9.62E+01	9.71E+01
1717.81	9.52E+01	9.61E+01	9.71E+01
1718.292	9.53E+01	9.60E+01	9.71E+01
1718.774	9.54E+01	9.60E+01	9.71E+01
1719.256	9.55E+01	9.59E+01	9.71E+01
1719.739	9.55E+01	9.59E+01	9.71E+01
1720.221	9.56E+01	9.59E+01	9.71E+01
1720.703	9.56E+01	9.59E+01	9.71E+01
1721.185	9.56E+01	9.58E+01	9.71E+01
1721.667	9.56E+01	9.58E+01	9.71E+01
1722.149	9.55E+01	9.58E+01	9.71E+01
1722.631	9.55E+01	9.57E+01	9.71E+01
1723.113	9.54E+01	9.57E+01	9.71E+01
1723.595	9.54E+01	9.56E+01	9.71E+01
1724.078	9.54E+01	9.56E+01	9.71E+01
1724.56	9.53E+01	9.55E+01	9.71E+01
1725.042	9.53E+01	9.55E+01	9.71E+01
1725.524	9.53E+01	9.55E+01	9.71E+01
1726.006	9.53E+01	9.54E+01	9.70E+01
1726.488	9.53E+01	9.54E+01	9.70E+01
1726.97	9.52E+01	9.54E+01	9.70E+01
1727.453	9.51E+01	9.53E+01	9.69E+01
1727.935	9.51E+01	9.53E+01	9.69E+01
1728.417	9.50E+01	9.52E+01	9.68E+01
1728.899	9.49E+01	9.52E+01	9.67E+01
1729.381	9.48E+01	9.51E+01	9.67E+01
1729.863	9.47E+01	9.51E+01	9.67E+01
1730.345	9.46E+01	9.51E+01	9.66E+01
1730.827	9.46E+01	9.51E+01	9.66E+01
1731.309	9.45E+01	9.51E+01	9.66E+01
1731.792	9.44E+01	9.51E+01	9.66E+01
1732.274	9.43E+01	9.51E+01	9.65E+01
1732.756	9.42E+01	9.51E+01	9.65E+01
1733.238	9.42E+01	9.51E+01	9.65E+01
1733.72	9.42E+01	9.52E+01	9.66E+01
1734.202	9.43E+01	9.52E+01	9.66E+01
1734.684	9.44E+01	9.53E+01	9.67E+01
1735.167	9.46E+01	9.53E+01	9.68E+01
1735.649	9.48E+01	9.54E+01	9.69E+01

1736.131	9.49E+01	9.55E+01	9.69E+01
1736.613	9.50E+01	9.55E+01	9.70E+01
1737.095	9.51E+01	9.56E+01	9.70E+01
1737.577	9.51E+01	9.56E+01	9.70E+01
1738.059	9.51E+01	9.57E+01	9.70E+01
1738.541	9.52E+01	9.57E+01	9.70E+01
1739.023	9.52E+01	9.58E+01	9.70E+01
1739.506	9.53E+01	9.59E+01	9.71E+01
1739.988	9.54E+01	9.60E+01	9.71E+01
1740.47	9.55E+01	9.61E+01	9.72E+01
1740.952	9.56E+01	9.62E+01	9.72E+01
1741.434	9.56E+01	9.62E+01	9.73E+01
1741.916	9.57E+01	9.63E+01	9.73E+01
1742.398	9.57E+01	9.64E+01	9.73E+01
1742.88	9.58E+01	9.64E+01	9.73E+01
1743.363	9.58E+01	9.65E+01	9.73E+01
1743.845	9.58E+01	9.65E+01	9.74E+01
1744.327	9.59E+01	9.66E+01	9.74E+01
1744.809	9.60E+01	9.66E+01	9.74E+01
1745.291	9.60E+01	9.67E+01	9.75E+01
1745.773	9.61E+01	9.67E+01	9.75E+01
1746.255	9.61E+01	9.68E+01	9.76E+01
1746.737	9.61E+01	9.68E+01	9.76E+01
1747.22	9.61E+01	9.69E+01	9.77E+01
1747.702	9.61E+01	9.69E+01	9.77E+01
1748.184	9.61E+01	9.70E+01	9.77E+01
1748.666	9.62E+01	9.70E+01	9.78E+01
1749.148	9.62E+01	9.71E+01	9.78E+01
1749.63	9.63E+01	9.72E+01	9.78E+01
1750.112	9.65E+01	9.72E+01	9.79E+01
1750.594	9.66E+01	9.73E+01	9.79E+01
1751.077	9.67E+01	9.74E+01	9.79E+01
1751.559	9.68E+01	9.74E+01	9.80E+01
1752.041	9.69E+01	9.75E+01	9.80E+01
1752.523	9.70E+01	9.75E+01	9.81E+01
1753.005	9.71E+01	9.75E+01	9.81E+01
1753.487	9.72E+01	9.75E+01	9.81E+01
1753.969	9.72E+01	9.75E+01	9.81E+01
1754.451	9.72E+01	9.75E+01	9.82E+01
1754.934	9.72E+01	9.75E+01	9.82E+01
1755.416	9.72E+01	9.75E+01	9.82E+01
1755.898	9.71E+01	9.75E+01	9.82E+01
1756.38	9.71E+01	9.75E+01	9.82E+01
1756.862	9.71E+01	9.75E+01	9.82E+01
1757.344	9.71E+01	9.75E+01	9.82E+01
1757.826	9.72E+01	9.75E+01	9.82E+01
1758.308	9.72E+01	9.75E+01	9.82E+01

1758.791	9.72E+01	9.75E+01	9.82E+01
1759.273	9.72E+01	9.75E+01	9.82E+01
1759.755	9.72E+01	9.75E+01	9.82E+01
1760.237	9.72E+01	9.76E+01	9.82E+01
1760.719	9.72E+01	9.76E+01	9.82E+01
1761.201	9.72E+01	9.76E+01	9.82E+01
1761.683	9.72E+01	9.76E+01	9.82E+01
1762.165	9.72E+01	9.76E+01	9.83E+01
1762.648	9.73E+01	9.77E+01	9.83E+01
1763.13	9.73E+01	9.77E+01	9.83E+01
1763.612	9.74E+01	9.77E+01	9.83E+01
1764.094	9.75E+01	9.78E+01	9.84E+01
1764.576	9.75E+01	9.78E+01	9.84E+01
1765.058	9.75E+01	9.78E+01	9.83E+01
1765.54	9.75E+01	9.78E+01	9.83E+01
1766.022	9.75E+01	9.77E+01	9.83E+01
1766.505	9.74E+01	9.77E+01	9.82E+01
1766.987	9.74E+01	9.77E+01	9.81E+01
1767.469	9.73E+01	9.77E+01	9.81E+01
1767.951	9.73E+01	9.76E+01	9.81E+01
1768.433	9.72E+01	9.76E+01	9.80E+01
1768.915	9.72E+01	9.76E+01	9.80E+01
1769.397	9.71E+01	9.76E+01	9.79E+01
1769.879	9.70E+01	9.75E+01	9.79E+01
1770.362	9.69E+01	9.75E+01	9.78E+01
1770.844	9.67E+01	9.74E+01	9.78E+01
1771.326	9.66E+01	9.74E+01	9.78E+01
1771.808	9.66E+01	9.74E+01	9.78E+01
1772.29	9.66E+01	9.74E+01	9.79E+01
1772.772	9.66E+01	9.75E+01	9.79E+01
1773.254	9.67E+01	9.75E+01	9.80E+01
1773.736	9.69E+01	9.75E+01	9.81E+01
1774.219	9.70E+01	9.76E+01	9.81E+01
1774.701	9.70E+01	9.76E+01	9.81E+01
1775.183	9.71E+01	9.77E+01	9.82E+01
1775.665	9.72E+01	9.77E+01	9.82E+01
1776.147	9.73E+01	9.77E+01	9.83E+01
1776.629	9.73E+01	9.78E+01	9.83E+01
1777.111	9.74E+01	9.78E+01	9.83E+01
1777.593	9.74E+01	9.78E+01	9.84E+01
1778.076	9.74E+01	9.78E+01	9.84E+01
1778.558	9.74E+01	9.78E+01	9.84E+01
1779.04	9.73E+01	9.78E+01	9.83E+01
1779.522	9.73E+01	9.77E+01	9.83E+01
1780.004	9.73E+01	9.77E+01	9.83E+01
1780.486	9.73E+01	9.77E+01	9.82E+01
1780.968	9.73E+01	9.76E+01	9.82E+01

1781.45	9.74E+01	9.76E+01	9.82E+01
1781.932	9.74E+01	9.76E+01	9.82E+01
1782.415	9.75E+01	9.76E+01	9.82E+01
1782.897	9.75E+01	9.76E+01	9.82E+01
1783.379	9.75E+01	9.76E+01	9.82E+01
1783.861	9.75E+01	9.76E+01	9.82E+01
1784.343	9.75E+01	9.76E+01	9.83E+01
1784.825	9.75E+01	9.76E+01	9.83E+01
1785.307	9.75E+01	9.76E+01	9.83E+01
1785.79	9.75E+01	9.77E+01	9.83E+01
1786.272	9.75E+01	9.77E+01	9.83E+01
1786.754	9.75E+01	9.77E+01	9.83E+01
1787.236	9.75E+01	9.77E+01	9.83E+01
1787.718	9.75E+01	9.77E+01	9.82E+01
1788.2	9.74E+01	9.77E+01	9.82E+01
1788.682	9.74E+01	9.77E+01	9.82E+01
1789.164	9.73E+01	9.77E+01	9.82E+01
1789.647	9.72E+01	9.77E+01	9.82E+01
1790.129	9.71E+01	9.76E+01	9.81E+01
1790.611	9.70E+01	9.76E+01	9.81E+01
1791.093	9.69E+01	9.76E+01	9.81E+01
1791.575	9.69E+01	9.76E+01	9.81E+01
1792.057	9.68E+01	9.76E+01	9.81E+01
1792.539	9.69E+01	9.76E+01	9.81E+01
1793.021	9.69E+01	9.76E+01	9.81E+01
1793.504	9.70E+01	9.76E+01	9.81E+01
1793.986	9.71E+01	9.76E+01	9.81E+01
1794.468	9.72E+01	9.76E+01	9.81E+01
1794.95	9.73E+01	9.76E+01	9.82E+01
1795.432	9.73E+01	9.77E+01	9.82E+01
1795.914	9.74E+01	9.77E+01	9.82E+01
1796.396	9.74E+01	9.77E+01	9.81E+01
1796.878	9.74E+01	9.77E+01	9.81E+01
1797.361	9.74E+01	9.77E+01	9.81E+01
1797.843	9.73E+01	9.77E+01	9.81E+01
1798.325	9.73E+01	9.77E+01	9.81E+01
1798.807	9.73E+01	9.77E+01	9.80E+01
1799.289	9.72E+01	9.77E+01	9.80E+01
1799.771	9.72E+01	9.77E+01	9.80E+01
1800.253	9.72E+01	9.77E+01	9.80E+01
1800.735	9.71E+01	9.77E+01	9.80E+01
1801.218	9.72E+01	9.77E+01	9.81E+01
1801.7	9.72E+01	9.77E+01	9.81E+01
1802.182	9.72E+01	9.77E+01	9.82E+01
1802.664	9.73E+01	9.77E+01	9.82E+01
1803.146	9.73E+01	9.77E+01	9.83E+01
1803.628	9.73E+01	9.77E+01	9.83E+01

1804.11	9.74E+01	9.78E+01	9.83E+01
1804.592	9.74E+01	9.78E+01	9.84E+01
1805.075	9.74E+01	9.78E+01	9.84E+01
1805.557	9.74E+01	9.78E+01	9.84E+01
1806.039	9.74E+01	9.78E+01	9.84E+01
1806.521	9.74E+01	9.77E+01	9.84E+01
1807.003	9.74E+01	9.77E+01	9.84E+01
1807.485	9.74E+01	9.77E+01	9.83E+01
1807.967	9.74E+01	9.77E+01	9.83E+01
1808.449	9.74E+01	9.77E+01	9.83E+01
1808.932	9.74E+01	9.77E+01	9.83E+01
1809.414	9.74E+01	9.77E+01	9.82E+01
1809.896	9.74E+01	9.77E+01	9.82E+01
1810.378	9.74E+01	9.77E+01	9.82E+01
1810.86	9.74E+01	9.76E+01	9.82E+01
1811.342	9.74E+01	9.76E+01	9.82E+01
1811.824	9.74E+01	9.76E+01	9.82E+01
1812.306	9.74E+01	9.75E+01	9.82E+01
1812.789	9.74E+01	9.75E+01	9.82E+01
1813.271	9.74E+01	9.75E+01	9.82E+01
1813.753	9.74E+01	9.75E+01	9.82E+01
1814.235	9.74E+01	9.75E+01	9.82E+01
1814.717	9.74E+01	9.75E+01	9.82E+01
1815.199	9.74E+01	9.75E+01	9.82E+01
1815.681	9.74E+01	9.75E+01	9.82E+01
1816.163	9.73E+01	9.75E+01	9.82E+01
1816.646	9.73E+01	9.75E+01	9.82E+01
1817.128	9.73E+01	9.75E+01	9.82E+01
1817.61	9.73E+01	9.75E+01	9.81E+01
1818.092	9.73E+01	9.75E+01	9.81E+01
1818.574	9.73E+01	9.75E+01	9.81E+01
1819.056	9.73E+01	9.75E+01	9.81E+01
1819.538	9.73E+01	9.75E+01	9.81E+01
1820.02	9.74E+01	9.76E+01	9.81E+01
1820.503	9.74E+01	9.76E+01	9.81E+01
1820.985	9.73E+01	9.76E+01	9.81E+01
1821.467	9.73E+01	9.76E+01	9.81E+01
1821.949	9.73E+01	9.76E+01	9.81E+01
1822.431	9.73E+01	9.76E+01	9.81E+01
1822.913	9.72E+01	9.76E+01	9.81E+01
1823.395	9.72E+01	9.76E+01	9.81E+01
1823.877	9.72E+01	9.76E+01	9.82E+01
1824.359	9.72E+01	9.77E+01	9.82E+01
1824.842	9.72E+01	9.77E+01	9.82E+01
1825.324	9.72E+01	9.76E+01	9.83E+01
1825.806	9.73E+01	9.76E+01	9.83E+01
1826.288	9.73E+01	9.76E+01	9.83E+01

1826.77	9.74E+01	9.76E+01	9.83E+01
1827.252	9.74E+01	9.76E+01	9.83E+01
1827.734	9.74E+01	9.76E+01	9.83E+01
1828.217	9.73E+01	9.75E+01	9.83E+01
1828.699	9.73E+01	9.75E+01	9.82E+01
1829.181	9.72E+01	9.75E+01	9.82E+01
1829.663	9.72E+01	9.75E+01	9.81E+01
1830.145	9.72E+01	9.75E+01	9.80E+01
1830.627	9.72E+01	9.75E+01	9.80E+01
1831.109	9.72E+01	9.76E+01	9.80E+01
1831.591	9.73E+01	9.76E+01	9.80E+01
1832.073	9.74E+01	9.76E+01	9.80E+01
1832.556	9.75E+01	9.77E+01	9.80E+01
1833.038	9.75E+01	9.77E+01	9.81E+01
1833.52	9.76E+01	9.77E+01	9.81E+01
1834.002	9.76E+01	9.77E+01	9.81E+01
1834.484	9.76E+01	9.76E+01	9.82E+01
1834.966	9.76E+01	9.76E+01	9.82E+01
1835.448	9.75E+01	9.76E+01	9.82E+01
1835.931	9.75E+01	9.76E+01	9.82E+01
1836.413	9.75E+01	9.76E+01	9.82E+01
1836.895	9.75E+01	9.76E+01	9.82E+01
1837.377	9.75E+01	9.76E+01	9.83E+01
1837.859	9.75E+01	9.76E+01	9.83E+01
1838.341	9.75E+01	9.76E+01	9.83E+01
1838.823	9.75E+01	9.77E+01	9.83E+01
1839.305	9.75E+01	9.77E+01	9.83E+01
1839.787	9.75E+01	9.78E+01	9.83E+01
1840.27	9.75E+01	9.78E+01	9.83E+01
1840.752	9.75E+01	9.78E+01	9.83E+01
1841.234	9.74E+01	9.78E+01	9.83E+01
1841.716	9.74E+01	9.78E+01	9.82E+01
1842.198	9.73E+01	9.78E+01	9.82E+01
1842.68	9.72E+01	9.78E+01	9.82E+01
1843.162	9.72E+01	9.78E+01	9.81E+01
1843.645	9.71E+01	9.78E+01	9.81E+01
1844.127	9.70E+01	9.78E+01	9.81E+01
1844.609	9.70E+01	9.78E+01	9.80E+01
1845.091	9.69E+01	9.78E+01	9.80E+01
1845.573	9.69E+01	9.77E+01	9.80E+01
1846.055	9.69E+01	9.76E+01	9.80E+01
1846.537	9.69E+01	9.76E+01	9.80E+01
1847.019	9.69E+01	9.75E+01	9.80E+01
1847.501	9.70E+01	9.75E+01	9.81E+01
1847.984	9.70E+01	9.75E+01	9.81E+01
1848.466	9.71E+01	9.75E+01	9.82E+01
1848.948	9.73E+01	9.75E+01	9.83E+01

1849.43	9.74E+01	9.75E+01	9.84E+01
1849.912	9.74E+01	9.76E+01	9.84E+01
1850.394	9.75E+01	9.76E+01	9.84E+01
1850.876	9.75E+01	9.76E+01	9.83E+01
1851.359	9.75E+01	9.76E+01	9.83E+01
1851.841	9.75E+01	9.76E+01	9.82E+01
1852.323	9.75E+01	9.76E+01	9.81E+01
1852.805	9.75E+01	9.76E+01	9.81E+01
1853.287	9.75E+01	9.76E+01	9.80E+01
1853.769	9.75E+01	9.76E+01	9.80E+01
1854.251	9.75E+01	9.76E+01	9.80E+01
1854.733	9.76E+01	9.76E+01	9.81E+01
1855.215	9.76E+01	9.77E+01	9.81E+01
1855.698	9.77E+01	9.77E+01	9.81E+01
1856.18	9.77E+01	9.77E+01	9.82E+01
1856.662	9.77E+01	9.76E+01	9.82E+01
1857.144	9.77E+01	9.76E+01	9.82E+01
1857.626	9.77E+01	9.76E+01	9.82E+01
1858.108	9.76E+01	9.76E+01	9.82E+01
1858.59	9.76E+01	9.76E+01	9.82E+01
1859.073	9.75E+01	9.76E+01	9.82E+01
1859.555	9.75E+01	9.76E+01	9.82E+01
1860.037	9.74E+01	9.75E+01	9.82E+01
1860.519	9.74E+01	9.75E+01	9.82E+01
1861.001	9.74E+01	9.75E+01	9.82E+01
1861.483	9.74E+01	9.75E+01	9.81E+01
1861.965	9.74E+01	9.75E+01	9.81E+01
1862.447	9.74E+01	9.76E+01	9.81E+01
1862.929	9.74E+01	9.76E+01	9.81E+01
1863.412	9.74E+01	9.76E+01	9.81E+01
1863.894	9.74E+01	9.76E+01	9.82E+01
1864.376	9.74E+01	9.76E+01	9.82E+01
1864.858	9.74E+01	9.76E+01	9.82E+01
1865.34	9.73E+01	9.76E+01	9.81E+01
1865.822	9.73E+01	9.76E+01	9.81E+01
1866.304	9.72E+01	9.75E+01	9.80E+01
1866.786	9.71E+01	9.74E+01	9.80E+01
1867.269	9.70E+01	9.74E+01	9.79E+01
1867.751	9.69E+01	9.73E+01	9.79E+01
1868.233	9.68E+01	9.73E+01	9.79E+01
1868.715	9.68E+01	9.73E+01	9.79E+01
1869.197	9.68E+01	9.73E+01	9.79E+01
1869.679	9.69E+01	9.73E+01	9.80E+01
1870.161	9.69E+01	9.74E+01	9.80E+01
1870.643	9.71E+01	9.74E+01	9.81E+01
1871.126	9.72E+01	9.74E+01	9.81E+01
1871.608	9.73E+01	9.75E+01	9.82E+01

1872.09	9.74E+01	9.75E+01	9.82E+01
1872.572	9.75E+01	9.75E+01	9.82E+01
1873.054	9.76E+01	9.76E+01	9.83E+01
1873.536	9.76E+01	9.76E+01	9.83E+01
1874.018	9.77E+01	9.76E+01	9.83E+01
1874.5	9.77E+01	9.76E+01	9.82E+01
1874.983	9.76E+01	9.76E+01	9.82E+01
1875.465	9.75E+01	9.75E+01	9.82E+01
1875.947	9.75E+01	9.75E+01	9.81E+01
1876.429	9.74E+01	9.75E+01	9.81E+01
1876.911	9.73E+01	9.74E+01	9.80E+01
1877.393	9.73E+01	9.74E+01	9.80E+01
1877.875	9.73E+01	9.74E+01	9.79E+01
1878.357	9.73E+01	9.73E+01	9.78E+01
1878.84	9.73E+01	9.73E+01	9.78E+01
1879.322	9.73E+01	9.73E+01	9.78E+01
1879.804	9.73E+01	9.73E+01	9.77E+01
1880.286	9.73E+01	9.73E+01	9.78E+01
1880.768	9.73E+01	9.73E+01	9.78E+01
1881.25	9.74E+01	9.73E+01	9.79E+01
1881.732	9.74E+01	9.73E+01	9.80E+01
1882.214	9.74E+01	9.73E+01	9.80E+01
1882.697	9.73E+01	9.73E+01	9.81E+01
1883.179	9.73E+01	9.73E+01	9.81E+01
1883.661	9.73E+01	9.73E+01	9.81E+01
1884.143	9.73E+01	9.73E+01	9.81E+01
1884.625	9.72E+01	9.73E+01	9.81E+01
1885.107	9.72E+01	9.73E+01	9.81E+01
1885.589	9.72E+01	9.73E+01	9.81E+01
1886.071	9.72E+01	9.72E+01	9.80E+01
1886.554	9.72E+01	9.72E+01	9.80E+01
1887.036	9.71E+01	9.72E+01	9.80E+01
1887.518	9.71E+01	9.72E+01	9.79E+01
1888	9.70E+01	9.72E+01	9.79E+01
1888.482	9.70E+01	9.72E+01	9.79E+01
1888.964	9.70E+01	9.73E+01	9.79E+01
1889.446	9.70E+01	9.74E+01	9.79E+01
1889.928	9.71E+01	9.75E+01	9.80E+01
1890.411	9.72E+01	9.76E+01	9.81E+01
1890.893	9.73E+01	9.77E+01	9.82E+01
1891.375	9.74E+01	9.78E+01	9.83E+01
1891.857	9.75E+01	9.79E+01	9.84E+01
1892.339	9.75E+01	9.79E+01	9.84E+01
1892.821	9.75E+01	9.79E+01	9.85E+01
1893.303	9.74E+01	9.78E+01	9.85E+01
1893.785	9.74E+01	9.77E+01	9.85E+01
1894.268	9.73E+01	9.77E+01	9.85E+01

1894.75	9.73E+01	9.76E+01	9.85E+01
1895.232	9.73E+01	9.76E+01	9.85E+01
1895.714	9.73E+01	9.76E+01	9.85E+01
1896.196	9.74E+01	9.76E+01	9.84E+01
1896.678	9.75E+01	9.76E+01	9.84E+01
1897.16	9.76E+01	9.76E+01	9.83E+01
1897.642	9.76E+01	9.76E+01	9.82E+01
1898.125	9.76E+01	9.76E+01	9.81E+01
1898.607	9.76E+01	9.76E+01	9.81E+01
1899.089	9.76E+01	9.76E+01	9.80E+01
1899.571	9.76E+01	9.76E+01	9.80E+01
1900.053	9.76E+01	9.76E+01	9.81E+01
1900.535	9.76E+01	9.77E+01	9.81E+01
1901.017	9.77E+01	9.77E+01	9.82E+01
1901.5	9.77E+01	9.78E+01	9.83E+01
1901.982	9.76E+01	9.77E+01	9.83E+01
1902.464	9.75E+01	9.77E+01	9.84E+01
1902.946	9.74E+01	9.76E+01	9.84E+01
1903.428	9.73E+01	9.75E+01	9.83E+01
1903.91	9.72E+01	9.74E+01	9.83E+01
1904.392	9.71E+01	9.73E+01	9.83E+01
1904.874	9.71E+01	9.72E+01	9.82E+01
1905.356	9.71E+01	9.72E+01	9.82E+01
1905.839	9.70E+01	9.71E+01	9.82E+01
1906.321	9.70E+01	9.71E+01	9.82E+01
1906.803	9.70E+01	9.70E+01	9.82E+01
1907.285	9.69E+01	9.70E+01	9.82E+01
1907.767	9.69E+01	9.71E+01	9.81E+01
1908.249	9.69E+01	9.71E+01	9.81E+01
1908.731	9.69E+01	9.72E+01	9.81E+01
1909.214	9.69E+01	9.73E+01	9.81E+01
1909.696	9.70E+01	9.73E+01	9.80E+01
1910.178	9.70E+01	9.73E+01	9.80E+01
1910.66	9.71E+01	9.73E+01	9.79E+01
1911.142	9.71E+01	9.73E+01	9.79E+01
1911.624	9.72E+01	9.73E+01	9.79E+01
1912.106	9.72E+01	9.73E+01	9.79E+01
1912.588	9.72E+01	9.73E+01	9.80E+01
1913.07	9.72E+01	9.73E+01	9.81E+01
1913.553	9.71E+01	9.74E+01	9.81E+01
1914.035	9.71E+01	9.75E+01	9.82E+01
1914.517	9.71E+01	9.75E+01	9.82E+01
1914.999	9.71E+01	9.76E+01	9.81E+01
1915.481	9.70E+01	9.76E+01	9.81E+01
1915.963	9.70E+01	9.76E+01	9.80E+01
1916.445	9.70E+01	9.76E+01	9.79E+01
1916.927	9.69E+01	9.75E+01	9.79E+01

1917.41	9.69E+01	9.75E+01	9.79E+01
1917.892	9.70E+01	9.74E+01	9.79E+01
1918.374	9.70E+01	9.74E+01	9.80E+01
1918.856	9.71E+01	9.74E+01	9.82E+01
1919.338	9.72E+01	9.75E+01	9.83E+01
1919.82	9.72E+01	9.75E+01	9.85E+01
1920.302	9.72E+01	9.76E+01	9.86E+01
1920.784	9.72E+01	9.77E+01	9.86E+01
1921.267	9.72E+01	9.77E+01	9.86E+01
1921.749	9.72E+01	9.76E+01	9.86E+01
1922.231	9.72E+01	9.76E+01	9.85E+01
1922.713	9.72E+01	9.75E+01	9.85E+01
1923.195	9.71E+01	9.75E+01	9.85E+01
1923.677	9.72E+01	9.74E+01	9.84E+01
1924.159	9.72E+01	9.74E+01	9.84E+01
1924.641	9.73E+01	9.74E+01	9.84E+01
1925.124	9.73E+01	9.74E+01	9.84E+01
1925.606	9.74E+01	9.74E+01	9.83E+01
1926.088	9.75E+01	9.74E+01	9.83E+01
1926.57	9.76E+01	9.74E+01	9.83E+01
1927.052	9.76E+01	9.74E+01	9.83E+01
1927.534	9.76E+01	9.74E+01	9.83E+01
1928.016	9.76E+01	9.73E+01	9.82E+01
1928.498	9.76E+01	9.72E+01	9.82E+01
1928.981	9.75E+01	9.71E+01	9.81E+01
1929.463	9.74E+01	9.71E+01	9.80E+01
1929.945	9.73E+01	9.71E+01	9.79E+01
1930.427	9.72E+01	9.71E+01	9.79E+01
1930.909	9.72E+01	9.72E+01	9.78E+01
1931.391	9.72E+01	9.73E+01	9.78E+01
1931.873	9.72E+01	9.74E+01	9.78E+01
1932.355	9.73E+01	9.74E+01	9.79E+01
1932.838	9.74E+01	9.75E+01	9.79E+01
1933.32	9.74E+01	9.75E+01	9.79E+01
1933.802	9.75E+01	9.75E+01	9.80E+01
1934.284	9.75E+01	9.75E+01	9.81E+01
1934.766	9.74E+01	9.75E+01	9.81E+01
1935.248	9.74E+01	9.75E+01	9.83E+01
1935.73	9.74E+01	9.76E+01	9.84E+01
1936.212	9.74E+01	9.76E+01	9.85E+01
1936.695	9.75E+01	9.77E+01	9.85E+01
1937.177	9.75E+01	9.77E+01	9.85E+01
1937.659	9.75E+01	9.76E+01	9.85E+01
1938.141	9.75E+01	9.76E+01	9.84E+01
1938.623	9.75E+01	9.75E+01	9.83E+01
1939.105	9.75E+01	9.74E+01	9.82E+01
1939.587	9.74E+01	9.73E+01	9.80E+01

1940.069	9.73E+01	9.72E+01	9.79E+01
1940.552	9.72E+01	9.71E+01	9.78E+01
1941.034	9.72E+01	9.71E+01	9.78E+01
1941.516	9.72E+01	9.71E+01	9.78E+01
1941.998	9.72E+01	9.72E+01	9.79E+01
1942.48	9.73E+01	9.73E+01	9.80E+01
1942.962	9.75E+01	9.75E+01	9.82E+01
1943.444	9.76E+01	9.76E+01	9.83E+01
1943.926	9.77E+01	9.77E+01	9.83E+01
1944.409	9.77E+01	9.77E+01	9.82E+01
1944.891	9.77E+01	9.77E+01	9.81E+01
1945.373	9.77E+01	9.76E+01	9.79E+01
1945.855	9.78E+01	9.75E+01	9.78E+01
1946.337	9.78E+01	9.74E+01	9.77E+01
1946.819	9.78E+01	9.73E+01	9.77E+01
1947.301	9.78E+01	9.72E+01	9.77E+01
1947.783	9.78E+01	9.72E+01	9.78E+01
1948.266	9.78E+01	9.71E+01	9.79E+01
1948.748	9.78E+01	9.71E+01	9.80E+01
1949.23	9.77E+01	9.71E+01	9.81E+01
1949.712	9.77E+01	9.71E+01	9.82E+01
1950.194	9.76E+01	9.70E+01	9.82E+01
1950.676	9.76E+01	9.70E+01	9.83E+01
1951.158	9.76E+01	9.70E+01	9.83E+01
1951.64	9.76E+01	9.70E+01	9.84E+01
1952.123	9.76E+01	9.70E+01	9.85E+01
1952.605	9.76E+01	9.71E+01	9.87E+01
1953.087	9.76E+01	9.71E+01	9.88E+01
1953.569	9.75E+01	9.71E+01	9.88E+01
1954.051	9.73E+01	9.71E+01	9.89E+01
1954.533	9.72E+01	9.72E+01	9.89E+01
1955.015	9.71E+01	9.72E+01	9.89E+01
1955.497	9.70E+01	9.72E+01	9.89E+01
1955.979	9.70E+01	9.73E+01	9.88E+01
1956.462	9.70E+01	9.74E+01	9.88E+01
1956.944	9.71E+01	9.75E+01	9.87E+01
1957.426	9.72E+01	9.76E+01	9.87E+01
1957.908	9.73E+01	9.78E+01	9.87E+01
1958.39	9.75E+01	9.79E+01	9.88E+01
1958.872	9.77E+01	9.80E+01	9.88E+01
1959.354	9.79E+01	9.81E+01	9.88E+01
1959.837	9.80E+01	9.81E+01	9.88E+01
1960.319	9.80E+01	9.80E+01	9.87E+01
1960.801	9.79E+01	9.78E+01	9.85E+01
1961.283	9.77E+01	9.75E+01	9.82E+01
1961.765	9.75E+01	9.70E+01	9.79E+01
1962.247	9.72E+01	9.66E+01	9.77E+01

1962.729	9.70E+01	9.62E+01	9.75E+01
1963.211	9.69E+01	9.59E+01	9.74E+01
1963.693	9.70E+01	9.57E+01	9.74E+01
1964.176	9.72E+01	9.57E+01	9.75E+01
1964.658	9.75E+01	9.58E+01	9.76E+01
1965.14	9.79E+01	9.61E+01	9.78E+01
1965.622	9.83E+01	9.65E+01	9.81E+01
1966.104	9.87E+01	9.70E+01	9.83E+01
1966.586	9.91E+01	9.75E+01	9.86E+01
1967.068	9.93E+01	9.80E+01	9.88E+01
1967.551	9.96E+01	9.85E+01	9.91E+01
1968.033	9.97E+01	9.90E+01	9.93E+01
1968.515	9.98E+01	9.94E+01	9.94E+01
1968.997	9.99E+01	9.97E+01	9.95E+01
1969.479	1.00E+02	9.99E+01	9.95E+01
1969.961	1.00E+02	1.00E+02	9.95E+01
1970.443	9.99E+01	9.99E+01	9.93E+01
1970.925	9.97E+01	9.98E+01	9.91E+01
1971.407	9.93E+01	9.95E+01	9.88E+01
1971.89	9.89E+01	9.93E+01	9.86E+01
1972.372	9.84E+01	9.91E+01	9.83E+01
1972.854	9.79E+01	9.90E+01	9.81E+01
1973.336	9.74E+01	9.88E+01	9.80E+01
1973.818	9.70E+01	9.86E+01	9.80E+01
1974.3	9.66E+01	9.84E+01	9.80E+01
1974.782	9.64E+01	9.80E+01	9.82E+01
1975.265	9.63E+01	9.77E+01	9.84E+01
1975.747	9.64E+01	9.74E+01	9.85E+01
1976.229	9.65E+01	9.72E+01	9.86E+01
1976.711	9.67E+01	9.70E+01	9.86E+01
1977.193	9.68E+01	9.70E+01	9.85E+01
1977.675	9.70E+01	9.70E+01	9.83E+01
1978.157	9.72E+01	9.71E+01	9.82E+01
1978.639	9.73E+01	9.72E+01	9.80E+01
1979.121	9.75E+01	9.73E+01	9.79E+01
1979.604	9.78E+01	9.76E+01	9.79E+01
1980.086	9.80E+01	9.79E+01	9.80E+01
1980.568	9.83E+01	9.82E+01	9.80E+01
1981.05	9.84E+01	9.85E+01	9.81E+01
1981.532	9.85E+01	9.88E+01	9.81E+01
1982.014	9.84E+01	9.90E+01	9.81E+01
1982.496	9.83E+01	9.90E+01	9.81E+01
1982.979	9.80E+01	9.89E+01	9.81E+01
1983.461	9.78E+01	9.87E+01	9.81E+01
1983.943	9.76E+01	9.85E+01	9.82E+01
1984.425	9.74E+01	9.83E+01	9.83E+01
1984.907	9.73E+01	9.81E+01	9.84E+01

1985.389	9.73E+01	9.79E+01	9.84E+01
1985.871	9.73E+01	9.78E+01	9.83E+01
1986.353	9.73E+01	9.76E+01	9.82E+01
1986.835	9.72E+01	9.76E+01	9.80E+01
1987.318	9.72E+01	9.75E+01	9.79E+01
1987.8	9.72E+01	9.75E+01	9.78E+01
1988.282	9.72E+01	9.76E+01	9.77E+01
1988.764	9.72E+01	9.77E+01	9.78E+01
1989.246	9.72E+01	9.78E+01	9.79E+01
1989.728	9.73E+01	9.79E+01	9.79E+01
1990.21	9.73E+01	9.79E+01	9.79E+01
1990.693	9.73E+01	9.78E+01	9.78E+01
1991.175	9.73E+01	9.75E+01	9.76E+01
1991.657	9.73E+01	9.72E+01	9.74E+01
1992.139	9.72E+01	9.68E+01	9.71E+01
1992.621	9.72E+01	9.65E+01	9.68E+01
1993.103	9.72E+01	9.62E+01	9.66E+01
1993.585	9.72E+01	9.60E+01	9.66E+01
1994.067	9.71E+01	9.59E+01	9.66E+01
1994.55	9.70E+01	9.59E+01	9.68E+01
1995.032	9.70E+01	9.60E+01	9.70E+01
1995.514	9.69E+01	9.62E+01	9.73E+01
1995.996	9.69E+01	9.66E+01	9.76E+01
1996.478	9.70E+01	9.69E+01	9.79E+01
1996.96	9.72E+01	9.74E+01	9.81E+01
1997.442	9.74E+01	9.78E+01	9.84E+01
1997.924	9.75E+01	9.81E+01	9.85E+01
1998.406	9.77E+01	9.83E+01	9.86E+01
1998.889	9.77E+01	9.83E+01	9.87E+01
1999.371	9.77E+01	9.83E+01	9.89E+01
1999.853	9.77E+01	9.83E+01	9.90E+01
2000.335	9.77E+01	9.82E+01	9.91E+01
2000.817	9.77E+01	9.81E+01	9.92E+01
2001.299	9.78E+01	9.80E+01	9.92E+01
2001.781	9.79E+01	9.79E+01	9.92E+01
2002.264	9.81E+01	9.78E+01	9.91E+01
2002.746	9.82E+01	9.76E+01	9.89E+01
2003.228	9.82E+01	9.75E+01	9.86E+01
2003.71	9.81E+01	9.73E+01	9.83E+01
2004.192	9.80E+01	9.71E+01	9.81E+01
2004.674	9.79E+01	9.69E+01	9.81E+01
2005.156	9.79E+01	9.68E+01	9.81E+01
2005.638	9.81E+01	9.68E+01	9.84E+01
2006.12	9.83E+01	9.69E+01	9.87E+01
2006.603	9.86E+01	9.71E+01	9.91E+01
2007.085	9.88E+01	9.74E+01	9.95E+01
2007.567	9.88E+01	9.76E+01	9.97E+01

2008.049	9.87E+01	9.77E+01	9.97E+01
2008.531	9.83E+01	9.78E+01	9.96E+01
2009.013	9.78E+01	9.77E+01	9.93E+01
2009.495	9.74E+01	9.76E+01	9.89E+01
2009.978	9.70E+01	9.74E+01	9.85E+01
2010.46	9.69E+01	9.73E+01	9.81E+01
2010.942	9.70E+01	9.73E+01	9.79E+01
2011.424	9.73E+01	9.73E+01	9.78E+01
2011.906	9.77E+01	9.74E+01	9.79E+01
2012.388	9.83E+01	9.76E+01	9.82E+01
2012.87	9.87E+01	9.78E+01	9.86E+01
2013.352	9.91E+01	9.79E+01	9.91E+01
2013.834	9.93E+01	9.80E+01	9.95E+01
2014.317	9.94E+01	9.80E+01	9.98E+01
2014.799	9.93E+01	9.79E+01	9.99E+01
2015.281	9.91E+01	9.78E+01	1.00E+02
2015.763	9.89E+01	9.77E+01	1.00E+02
2016.245	9.86E+01	9.77E+01	9.99E+01
2016.727	9.82E+01	9.78E+01	9.98E+01
2017.209	9.78E+01	9.79E+01	9.97E+01
2017.692	9.75E+01	9.80E+01	9.96E+01
2018.174	9.71E+01	9.81E+01	9.94E+01
2018.656	9.67E+01	9.82E+01	9.92E+01
2019.138	9.63E+01	9.81E+01	9.89E+01
2019.62	9.59E+01	9.80E+01	9.85E+01
2020.102	9.55E+01	9.79E+01	9.79E+01
2020.584	9.52E+01	9.76E+01	9.73E+01
2021.066	9.49E+01	9.72E+01	9.67E+01
2021.548	9.48E+01	9.69E+01	9.61E+01
2022.031	9.48E+01	9.66E+01	9.58E+01
2022.513	9.49E+01	9.63E+01	9.56E+01
2022.995	9.52E+01	9.61E+01	9.58E+01
2023.477	9.54E+01	9.61E+01	9.61E+01
2023.959	9.57E+01	9.61E+01	9.66E+01
2024.441	9.59E+01	9.63E+01	9.72E+01
2024.923	9.61E+01	9.66E+01	9.78E+01
2025.406	9.63E+01	9.69E+01	9.82E+01
2025.888	9.65E+01	9.72E+01	9.85E+01
2026.37	9.68E+01	9.75E+01	9.86E+01
2026.852	9.72E+01	9.79E+01	9.86E+01
2027.334	9.76E+01	9.82E+01	9.85E+01
2027.816	9.80E+01	9.84E+01	9.85E+01
2028.298	9.83E+01	9.87E+01	9.85E+01
2028.78	9.85E+01	9.89E+01	9.85E+01
2029.262	9.86E+01	9.91E+01	9.87E+01
2029.745	9.86E+01	9.92E+01	9.89E+01
2030.227	9.84E+01	9.93E+01	9.90E+01

2030.709	9.82E+01	9.93E+01	9.92E+01
2031.191	9.81E+01	9.92E+01	9.92E+01
2031.673	9.79E+01	9.90E+01	9.92E+01
2032.155	9.78E+01	9.88E+01	9.91E+01
2032.637	9.77E+01	9.85E+01	9.89E+01
2033.12	9.78E+01	9.83E+01	9.87E+01
2033.602	9.78E+01	9.82E+01	9.85E+01
2034.084	9.79E+01	9.81E+01	9.83E+01
2034.566	9.80E+01	9.81E+01	9.82E+01
2035.048	9.81E+01	9.81E+01	9.81E+01
2035.53	9.81E+01	9.81E+01	9.80E+01
2036.012	9.80E+01	9.80E+01	9.79E+01
2036.494	9.79E+01	9.78E+01	9.78E+01
2036.976	9.78E+01	9.75E+01	9.77E+01
2037.459	9.77E+01	9.73E+01	9.75E+01
2037.941	9.77E+01	9.71E+01	9.74E+01
2038.423	9.78E+01	9.69E+01	9.73E+01
2038.905	9.78E+01	9.68E+01	9.72E+01
2039.387	9.79E+01	9.67E+01	9.71E+01
2039.869	9.80E+01	9.67E+01	9.71E+01
2040.351	9.80E+01	9.66E+01	9.70E+01
2040.833	9.80E+01	9.65E+01	9.70E+01
2041.316	9.81E+01	9.64E+01	9.71E+01
2041.798	9.82E+01	9.63E+01	9.72E+01
2042.28	9.83E+01	9.63E+01	9.75E+01
2042.762	9.85E+01	9.63E+01	9.78E+01
2043.244	9.86E+01	9.64E+01	9.81E+01
2043.726	9.87E+01	9.66E+01	9.83E+01
2044.208	9.87E+01	9.68E+01	9.85E+01
2044.69	9.87E+01	9.70E+01	9.85E+01
2045.173	9.85E+01	9.71E+01	9.84E+01
2045.655	9.84E+01	9.72E+01	9.82E+01
2046.137	9.82E+01	9.71E+01	9.78E+01
2046.619	9.80E+01	9.69E+01	9.75E+01
2047.101	9.78E+01	9.67E+01	9.72E+01
2047.583	9.76E+01	9.64E+01	9.69E+01
2048.065	9.75E+01	9.62E+01	9.68E+01
2048.547	9.73E+01	9.60E+01	9.67E+01
2049.03	9.72E+01	9.59E+01	9.67E+01
2049.512	9.72E+01	9.59E+01	9.68E+01
2049.994	9.73E+01	9.60E+01	9.70E+01
2050.476	9.74E+01	9.61E+01	9.73E+01
2050.958	9.76E+01	9.62E+01	9.76E+01
2051.44	9.78E+01	9.64E+01	9.79E+01
2051.922	9.80E+01	9.65E+01	9.83E+01
2052.405	9.82E+01	9.66E+01	9.86E+01
2052.886	9.83E+01	9.68E+01	9.89E+01

2053.369	9.83E+01	9.70E+01	9.91E+01
2053.851	9.83E+01	9.72E+01	9.93E+01
2054.333	9.82E+01	9.75E+01	9.93E+01
2054.815	9.82E+01	9.77E+01	9.93E+01
2055.297	9.80E+01	9.79E+01	9.92E+01
2055.779	9.78E+01	9.79E+01	9.89E+01
2056.261	9.76E+01	9.78E+01	9.87E+01
2056.744	9.73E+01	9.77E+01	9.84E+01
2057.226	9.71E+01	9.74E+01	9.81E+01
2057.708	9.69E+01	9.72E+01	9.78E+01
2058.19	9.69E+01	9.70E+01	9.77E+01
2058.672	9.69E+01	9.68E+01	9.76E+01
2059.154	9.70E+01	9.67E+01	9.76E+01
2059.636	9.71E+01	9.67E+01	9.76E+01
2060.118	9.73E+01	9.68E+01	9.77E+01
2060.601	9.75E+01	9.69E+01	9.77E+01
2061.083	9.77E+01	9.70E+01	9.77E+01
2061.565	9.78E+01	9.72E+01	9.76E+01
2062.047	9.78E+01	9.74E+01	9.76E+01
2062.529	9.78E+01	9.75E+01	9.75E+01
2063.011	9.77E+01	9.77E+01	9.75E+01
2063.493	9.76E+01	9.78E+01	9.75E+01
2063.975	9.75E+01	9.78E+01	9.76E+01
2064.458	9.74E+01	9.78E+01	9.77E+01
2064.94	9.74E+01	9.77E+01	9.78E+01
2065.422	9.74E+01	9.76E+01	9.79E+01
2065.904	9.75E+01	9.75E+01	9.81E+01
2066.386	9.75E+01	9.74E+01	9.82E+01
2066.868	9.76E+01	9.73E+01	9.83E+01
2067.35	9.76E+01	9.73E+01	9.84E+01
2067.833	9.76E+01	9.73E+01	9.84E+01
2068.314	9.75E+01	9.72E+01	9.84E+01
2068.797	9.74E+01	9.72E+01	9.84E+01
2069.279	9.72E+01	9.71E+01	9.82E+01
2069.761	9.70E+01	9.71E+01	9.80E+01
2070.243	9.69E+01	9.70E+01	9.78E+01
2070.725	9.68E+01	9.70E+01	9.77E+01
2071.207	9.68E+01	9.70E+01	9.75E+01
2071.689	9.69E+01	9.70E+01	9.74E+01
2072.172	9.70E+01	9.71E+01	9.74E+01
2072.654	9.72E+01	9.71E+01	9.74E+01
2073.136	9.73E+01	9.72E+01	9.75E+01
2073.618	9.74E+01	9.73E+01	9.76E+01
2074.1	9.74E+01	9.73E+01	9.78E+01
2074.582	9.75E+01	9.73E+01	9.79E+01
2075.064	9.75E+01	9.73E+01	9.80E+01
2075.546	9.75E+01	9.72E+01	9.81E+01

2076.029	9.75E+01	9.72E+01	9.81E+01
2076.511	9.75E+01	9.72E+01	9.82E+01
2076.993	9.75E+01	9.72E+01	9.82E+01
2077.475	9.75E+01	9.71E+01	9.82E+01
2077.957	9.75E+01	9.71E+01	9.81E+01
2078.439	9.75E+01	9.71E+01	9.79E+01
2078.921	9.75E+01	9.71E+01	9.78E+01
2079.403	9.75E+01	9.71E+01	9.76E+01
2079.885	9.75E+01	9.72E+01	9.75E+01
2080.368	9.76E+01	9.72E+01	9.74E+01
2080.85	9.76E+01	9.73E+01	9.74E+01
2081.332	9.77E+01	9.74E+01	9.74E+01
2081.814	9.77E+01	9.74E+01	9.75E+01
2082.296	9.77E+01	9.73E+01	9.76E+01
2082.778	9.77E+01	9.72E+01	9.77E+01
2083.26	9.77E+01	9.71E+01	9.77E+01
2083.742	9.76E+01	9.69E+01	9.77E+01
2084.225	9.75E+01	9.69E+01	9.77E+01
2084.707	9.74E+01	9.68E+01	9.77E+01
2085.189	9.73E+01	9.69E+01	9.77E+01
2085.671	9.72E+01	9.69E+01	9.77E+01
2086.153	9.72E+01	9.70E+01	9.78E+01
2086.635	9.72E+01	9.70E+01	9.79E+01
2087.117	9.72E+01	9.70E+01	9.79E+01
2087.6	9.72E+01	9.70E+01	9.80E+01
2088.082	9.72E+01	9.69E+01	9.80E+01
2088.564	9.73E+01	9.68E+01	9.79E+01
2089.046	9.73E+01	9.68E+01	9.79E+01
2089.528	9.73E+01	9.68E+01	9.78E+01
2090.01	9.74E+01	9.68E+01	9.78E+01
2090.492	9.74E+01	9.68E+01	9.78E+01
2090.974	9.74E+01	9.69E+01	9.78E+01
2091.457	9.74E+01	9.70E+01	9.78E+01
2091.939	9.74E+01	9.71E+01	9.78E+01
2092.421	9.74E+01	9.72E+01	9.78E+01
2092.903	9.73E+01	9.72E+01	9.78E+01
2093.385	9.72E+01	9.73E+01	9.78E+01
2093.867	9.71E+01	9.73E+01	9.77E+01
2094.349	9.70E+01	9.72E+01	9.77E+01
2094.831	9.68E+01	9.71E+01	9.76E+01
2095.313	9.67E+01	9.70E+01	9.76E+01
2095.796	9.66E+01	9.69E+01	9.76E+01
2096.278	9.66E+01	9.69E+01	9.77E+01
2096.76	9.66E+01	9.68E+01	9.77E+01
2097.242	9.67E+01	9.68E+01	9.78E+01
2097.724	9.67E+01	9.68E+01	9.79E+01
2098.206	9.68E+01	9.68E+01	9.80E+01

2098.688	9.68E+01	9.68E+01	9.80E+01
2099.171	9.69E+01	9.68E+01	9.80E+01
2099.653	9.69E+01	9.68E+01	9.80E+01
2100.135	9.70E+01	9.68E+01	9.80E+01
2100.617	9.70E+01	9.69E+01	9.79E+01
2101.099	9.71E+01	9.69E+01	9.78E+01
2101.581	9.73E+01	9.70E+01	9.77E+01
2102.063	9.74E+01	9.71E+01	9.77E+01
2102.545	9.74E+01	9.71E+01	9.77E+01
2103.028	9.75E+01	9.71E+01	9.77E+01
2103.51	9.75E+01	9.71E+01	9.78E+01
2103.992	9.75E+01	9.70E+01	9.79E+01
2104.474	9.74E+01	9.70E+01	9.80E+01
2104.956	9.74E+01	9.69E+01	9.82E+01
2105.438	9.73E+01	9.69E+01	9.83E+01
2105.92	9.73E+01	9.69E+01	9.84E+01
2106.402	9.73E+01	9.69E+01	9.84E+01
2106.885	9.72E+01	9.69E+01	9.84E+01
2107.367	9.72E+01	9.69E+01	9.84E+01
2107.849	9.72E+01	9.70E+01	9.84E+01
2108.331	9.71E+01	9.70E+01	9.84E+01
2108.813	9.71E+01	9.70E+01	9.84E+01
2109.295	9.72E+01	9.70E+01	9.84E+01
2109.777	9.72E+01	9.70E+01	9.83E+01
2110.26	9.73E+01	9.70E+01	9.83E+01
2110.741	9.74E+01	9.71E+01	9.83E+01
2111.224	9.74E+01	9.71E+01	9.82E+01
2111.706	9.75E+01	9.72E+01	9.81E+01
2112.188	9.75E+01	9.72E+01	9.80E+01
2112.67	9.76E+01	9.73E+01	9.79E+01
2113.152	9.76E+01	9.74E+01	9.78E+01
2113.634	9.76E+01	9.74E+01	9.77E+01
2114.116	9.76E+01	9.75E+01	9.76E+01
2114.599	9.76E+01	9.75E+01	9.75E+01
2115.081	9.76E+01	9.75E+01	9.75E+01
2115.563	9.76E+01	9.75E+01	9.74E+01
2116.045	9.76E+01	9.74E+01	9.74E+01
2116.527	9.75E+01	9.73E+01	9.74E+01
2117.009	9.74E+01	9.72E+01	9.74E+01
2117.491	9.73E+01	9.72E+01	9.74E+01
2117.973	9.71E+01	9.71E+01	9.74E+01
2118.456	9.70E+01	9.71E+01	9.74E+01
2118.938	9.68E+01	9.71E+01	9.75E+01
2119.42	9.67E+01	9.71E+01	9.76E+01
2119.902	9.65E+01	9.71E+01	9.76E+01
2120.384	9.65E+01	9.71E+01	9.77E+01
2120.866	9.64E+01	9.71E+01	9.77E+01

2121.348	9.65E+01	9.71E+01	9.77E+01
2121.83	9.65E+01	9.70E+01	9.77E+01
2122.313	9.65E+01	9.70E+01	9.76E+01
2122.795	9.65E+01	9.69E+01	9.75E+01
2123.277	9.65E+01	9.68E+01	9.73E+01
2123.759	9.64E+01	9.67E+01	9.72E+01
2124.241	9.63E+01	9.67E+01	9.71E+01
2124.723	9.63E+01	9.66E+01	9.69E+01
2125.205	9.62E+01	9.66E+01	9.69E+01
2125.688	9.62E+01	9.65E+01	9.68E+01
2126.169	9.63E+01	9.65E+01	9.68E+01
2126.652	9.63E+01	9.65E+01	9.69E+01
2127.134	9.65E+01	9.66E+01	9.69E+01
2127.616	9.66E+01	9.67E+01	9.70E+01
2128.098	9.68E+01	9.68E+01	9.71E+01
2128.58	9.70E+01	9.70E+01	9.72E+01
2129.062	9.71E+01	9.72E+01	9.73E+01
2129.544	9.73E+01	9.74E+01	9.75E+01
2130.027	9.75E+01	9.76E+01	9.76E+01
2130.509	9.76E+01	9.78E+01	9.77E+01
2130.991	9.77E+01	9.78E+01	9.77E+01
2131.473	9.78E+01	9.79E+01	9.78E+01
2131.955	9.79E+01	9.79E+01	9.78E+01
2132.437	9.79E+01	9.79E+01	9.78E+01
2132.919	9.79E+01	9.79E+01	9.77E+01
2133.401	9.78E+01	9.78E+01	9.77E+01
2133.884	9.77E+01	9.77E+01	9.76E+01
2134.366	9.76E+01	9.76E+01	9.74E+01
2134.848	9.74E+01	9.74E+01	9.72E+01
2135.33	9.72E+01	9.73E+01	9.71E+01
2135.812	9.71E+01	9.71E+01	9.70E+01
2136.294	9.69E+01	9.70E+01	9.69E+01
2136.776	9.68E+01	9.70E+01	9.70E+01
2137.258	9.68E+01	9.69E+01	9.71E+01
2137.74	9.68E+01	9.69E+01	9.73E+01
2138.223	9.69E+01	9.70E+01	9.75E+01
2138.705	9.70E+01	9.70E+01	9.77E+01
2139.187	9.72E+01	9.70E+01	9.79E+01
2139.669	9.73E+01	9.70E+01	9.80E+01
2140.151	9.74E+01	9.70E+01	9.80E+01
2140.633	9.75E+01	9.70E+01	9.81E+01
2141.115	9.76E+01	9.70E+01	9.82E+01
2141.597	9.76E+01	9.71E+01	9.82E+01
2142.08	9.76E+01	9.72E+01	9.83E+01
2142.562	9.77E+01	9.73E+01	9.83E+01
2143.044	9.77E+01	9.74E+01	9.84E+01
2143.526	9.77E+01	9.74E+01	9.83E+01

2144.008	9.76E+01	9.74E+01	9.83E+01
2144.49	9.76E+01	9.73E+01	9.82E+01
2144.972	9.75E+01	9.71E+01	9.80E+01
2145.455	9.74E+01	9.70E+01	9.78E+01
2145.937	9.73E+01	9.68E+01	9.77E+01
2146.419	9.72E+01	9.66E+01	9.75E+01
2146.901	9.73E+01	9.65E+01	9.73E+01
2147.383	9.74E+01	9.64E+01	9.72E+01
2147.865	9.77E+01	9.64E+01	9.72E+01
2148.347	9.80E+01	9.65E+01	9.73E+01
2148.829	9.84E+01	9.67E+01	9.76E+01
2149.312	9.88E+01	9.69E+01	9.80E+01
2149.794	9.92E+01	9.73E+01	9.85E+01
2150.276	9.96E+01	9.77E+01	9.90E+01
2150.758	9.98E+01	9.81E+01	9.95E+01
2151.24	1.00E+02	9.84E+01	9.98E+01
2151.722	9.99E+01	9.86E+01	9.99E+01
2152.204	9.97E+01	9.87E+01	9.98E+01
2152.686	9.93E+01	9.86E+01	9.96E+01
2153.168	9.88E+01	9.85E+01	9.92E+01
2153.651	9.83E+01	9.83E+01	9.89E+01
2154.133	9.78E+01	9.82E+01	9.86E+01
2154.615	9.74E+01	9.82E+01	9.85E+01
2155.097	9.71E+01	9.82E+01	9.85E+01
2155.579	9.69E+01	9.84E+01	9.85E+01
2156.061	9.68E+01	9.86E+01	9.86E+01
2156.543	9.68E+01	9.87E+01	9.87E+01
2157.025	9.67E+01	9.87E+01	9.87E+01
2157.508	9.67E+01	9.85E+01	9.86E+01
2157.99	9.66E+01	9.82E+01	9.84E+01
2158.472	9.65E+01	9.78E+01	9.82E+01
2158.954	9.65E+01	9.73E+01	9.78E+01
2159.436	9.64E+01	9.69E+01	9.75E+01
2159.918	9.62E+01	9.65E+01	9.71E+01
2160.4	9.61E+01	9.63E+01	9.68E+01
2160.883	9.60E+01	9.62E+01	9.66E+01
2161.365	9.59E+01	9.62E+01	9.65E+01
2161.847	9.60E+01	9.63E+01	9.65E+01
2162.329	9.61E+01	9.64E+01	9.65E+01
2162.811	9.63E+01	9.65E+01	9.66E+01
2163.293	9.65E+01	9.66E+01	9.68E+01
2163.775	9.68E+01	9.66E+01	9.70E+01
2164.257	9.71E+01	9.66E+01	9.71E+01
2164.74	9.72E+01	9.65E+01	9.73E+01
2165.222	9.74E+01	9.64E+01	9.75E+01
2165.704	9.75E+01	9.65E+01	9.77E+01
2166.186	9.77E+01	9.66E+01	9.80E+01

2166.668	9.79E+01	9.69E+01	9.84E+01
2167.15	9.81E+01	9.73E+01	9.88E+01
2167.632	9.83E+01	9.77E+01	9.91E+01
2168.114	9.85E+01	9.79E+01	9.94E+01
2168.596	9.85E+01	9.81E+01	9.95E+01
2169.079	9.84E+01	9.80E+01	9.94E+01
2169.561	9.82E+01	9.77E+01	9.91E+01
2170.043	9.78E+01	9.73E+01	9.88E+01
2170.525	9.75E+01	9.69E+01	9.84E+01
2171.007	9.72E+01	9.64E+01	9.80E+01
2171.489	9.70E+01	9.61E+01	9.78E+01
2171.971	9.69E+01	9.59E+01	9.76E+01
2172.453	9.69E+01	9.59E+01	9.76E+01
2172.936	9.69E+01	9.60E+01	9.76E+01
2173.418	9.69E+01	9.62E+01	9.77E+01
2173.9	9.70E+01	9.64E+01	9.78E+01
2174.382	9.69E+01	9.66E+01	9.78E+01
2174.864	9.69E+01	9.66E+01	9.78E+01
2175.346	9.68E+01	9.67E+01	9.77E+01
2175.828	9.67E+01	9.66E+01	9.76E+01
2176.311	9.66E+01	9.65E+01	9.74E+01
2176.792	9.66E+01	9.64E+01	9.73E+01
2177.275	9.65E+01	9.63E+01	9.72E+01
2177.757	9.65E+01	9.62E+01	9.72E+01
2178.239	9.66E+01	9.62E+01	9.73E+01
2178.721	9.67E+01	9.63E+01	9.74E+01
2179.203	9.69E+01	9.63E+01	9.75E+01
2179.685	9.71E+01	9.64E+01	9.75E+01
2180.167	9.74E+01	9.64E+01	9.76E+01
2180.65	9.77E+01	9.65E+01	9.75E+01
2181.132	9.80E+01	9.66E+01	9.75E+01
2181.614	9.82E+01	9.67E+01	9.75E+01
2182.096	9.84E+01	9.69E+01	9.75E+01
2182.578	9.84E+01	9.70E+01	9.76E+01
2183.06	9.84E+01	9.70E+01	9.77E+01
2183.542	9.82E+01	9.69E+01	9.77E+01
2184.024	9.79E+01	9.67E+01	9.77E+01
2184.507	9.76E+01	9.64E+01	9.75E+01
2184.989	9.72E+01	9.61E+01	9.72E+01
2185.471	9.68E+01	9.58E+01	9.68E+01
2185.953	9.65E+01	9.55E+01	9.64E+01
2186.435	9.63E+01	9.53E+01	9.60E+01
2186.917	9.61E+01	9.52E+01	9.58E+01
2187.399	9.61E+01	9.53E+01	9.57E+01
2187.881	9.61E+01	9.55E+01	9.58E+01
2188.364	9.61E+01	9.57E+01	9.61E+01
2188.846	9.63E+01	9.59E+01	9.65E+01

2189.328	9.64E+01	9.60E+01	9.70E+01
2189.81	9.66E+01	9.61E+01	9.74E+01
2190.292	9.68E+01	9.61E+01	9.77E+01
2190.774	9.70E+01	9.61E+01	9.79E+01
2191.256	9.71E+01	9.60E+01	9.80E+01
2191.739	9.72E+01	9.59E+01	9.79E+01
2192.221	9.73E+01	9.58E+01	9.79E+01
2192.703	9.74E+01	9.58E+01	9.78E+01
2193.185	9.75E+01	9.59E+01	9.78E+01
2193.667	9.76E+01	9.61E+01	9.79E+01
2194.149	9.78E+01	9.64E+01	9.81E+01
2194.631	9.80E+01	9.67E+01	9.84E+01
2195.113	9.81E+01	9.71E+01	9.86E+01
2195.595	9.82E+01	9.73E+01	9.88E+01
2196.078	9.83E+01	9.74E+01	9.89E+01
2196.56	9.83E+01	9.75E+01	9.89E+01
2197.042	9.83E+01	9.74E+01	9.89E+01
2197.524	9.81E+01	9.73E+01	9.88E+01
2198.006	9.80E+01	9.72E+01	9.86E+01
2198.488	9.79E+01	9.72E+01	9.85E+01
2198.97	9.78E+01	9.72E+01	9.84E+01
2199.452	9.77E+01	9.73E+01	9.82E+01
2199.935	9.77E+01	9.75E+01	9.81E+01
2200.417	9.77E+01	9.76E+01	9.80E+01
2200.899	9.78E+01	9.77E+01	9.79E+01
2201.381	9.79E+01	9.77E+01	9.77E+01
2201.863	9.80E+01	9.77E+01	9.76E+01
2202.345	9.81E+01	9.76E+01	9.75E+01
2202.827	9.81E+01	9.74E+01	9.73E+01
2203.31	9.81E+01	9.73E+01	9.71E+01
2203.792	9.79E+01	9.71E+01	9.70E+01
2204.274	9.76E+01	9.68E+01	9.68E+01
2204.756	9.73E+01	9.66E+01	9.67E+01
2205.238	9.70E+01	9.64E+01	9.67E+01
2205.72	9.67E+01	9.62E+01	9.68E+01
2206.202	9.65E+01	9.62E+01	9.69E+01
2206.684	9.64E+01	9.61E+01	9.71E+01
2207.167	9.64E+01	9.61E+01	9.74E+01
2207.649	9.66E+01	9.62E+01	9.76E+01
2208.131	9.68E+01	9.62E+01	9.77E+01
2208.613	9.70E+01	9.63E+01	9.78E+01
2209.095	9.72E+01	9.63E+01	9.78E+01
2209.577	9.73E+01	9.64E+01	9.78E+01
2210.059	9.75E+01	9.65E+01	9.78E+01
2210.541	9.76E+01	9.65E+01	9.79E+01
2211.023	9.76E+01	9.67E+01	9.79E+01
2211.506	9.76E+01	9.68E+01	9.80E+01

2211.988	9.75E+01	9.69E+01	9.82E+01
2212.47	9.73E+01	9.70E+01	9.82E+01
2212.952	9.72E+01	9.70E+01	9.82E+01
2213.434	9.69E+01	9.69E+01	9.82E+01
2213.916	9.67E+01	9.67E+01	9.80E+01
2214.398	9.65E+01	9.65E+01	9.79E+01
2214.88	9.63E+01	9.63E+01	9.77E+01
2215.363	9.62E+01	9.62E+01	9.75E+01
2215.845	9.61E+01	9.61E+01	9.74E+01
2216.327	9.61E+01	9.61E+01	9.73E+01
2216.809	9.61E+01	9.62E+01	9.74E+01
2217.291	9.62E+01	9.63E+01	9.75E+01
2217.773	9.62E+01	9.64E+01	9.76E+01
2218.255	9.63E+01	9.65E+01	9.78E+01
2218.738	9.63E+01	9.66E+01	9.79E+01
2219.219	9.64E+01	9.66E+01	9.80E+01
2219.702	9.65E+01	9.66E+01	9.81E+01
2220.184	9.66E+01	9.66E+01	9.81E+01
2220.666	9.67E+01	9.67E+01	9.82E+01
2221.148	9.69E+01	9.67E+01	9.83E+01
2221.63	9.71E+01	9.68E+01	9.83E+01
2222.112	9.74E+01	9.68E+01	9.84E+01
2222.594	9.75E+01	9.68E+01	9.84E+01
2223.077	9.77E+01	9.68E+01	9.83E+01
2223.559	9.78E+01	9.66E+01	9.81E+01
2224.041	9.78E+01	9.65E+01	9.79E+01
2224.523	9.77E+01	9.64E+01	9.77E+01
2225.005	9.76E+01	9.63E+01	9.75E+01
2225.487	9.75E+01	9.63E+01	9.73E+01
2225.969	9.74E+01	9.64E+01	9.72E+01
2226.451	9.73E+01	9.66E+01	9.72E+01
2226.934	9.72E+01	9.68E+01	9.73E+01
2227.416	9.71E+01	9.70E+01	9.74E+01
2227.898	9.70E+01	9.72E+01	9.74E+01
2228.38	9.70E+01	9.73E+01	9.75E+01
2228.862	9.69E+01	9.73E+01	9.75E+01
2229.344	9.68E+01	9.74E+01	9.76E+01
2229.826	9.67E+01	9.74E+01	9.76E+01
2230.308	9.66E+01	9.73E+01	9.76E+01
2230.791	9.66E+01	9.73E+01	9.76E+01
2231.273	9.65E+01	9.72E+01	9.77E+01
2231.755	9.64E+01	9.71E+01	9.76E+01
2232.237	9.64E+01	9.69E+01	9.76E+01
2232.719	9.63E+01	9.67E+01	9.75E+01
2233.201	9.62E+01	9.65E+01	9.73E+01
2233.683	9.60E+01	9.63E+01	9.72E+01
2234.166	9.59E+01	9.61E+01	9.70E+01

2234.647	9.57E+01	9.59E+01	9.68E+01
2235.13	9.55E+01	9.58E+01	9.67E+01
2235.612	9.53E+01	9.56E+01	9.65E+01
2236.094	9.51E+01	9.55E+01	9.64E+01
2236.576	9.50E+01	9.53E+01	9.63E+01
2237.058	9.48E+01	9.50E+01	9.62E+01
2237.54	9.47E+01	9.48E+01	9.61E+01
2238.022	9.46E+01	9.45E+01	9.60E+01
2238.505	9.45E+01	9.43E+01	9.60E+01
2238.987	9.44E+01	9.40E+01	9.60E+01
2239.469	9.43E+01	9.38E+01	9.59E+01
2239.951	9.42E+01	9.36E+01	9.59E+01
2240.433	9.41E+01	9.35E+01	9.59E+01
2240.915	9.40E+01	9.34E+01	9.59E+01
2241.397	9.40E+01	9.34E+01	9.59E+01
2241.879	9.40E+01	9.34E+01	9.59E+01
2242.362	9.40E+01	9.34E+01	9.59E+01
2242.844	9.41E+01	9.34E+01	9.59E+01
2243.326	9.41E+01	9.35E+01	9.59E+01
2243.808	9.41E+01	9.35E+01	9.59E+01
2244.29	9.42E+01	9.35E+01	9.59E+01
2244.772	9.42E+01	9.36E+01	9.58E+01
2245.254	9.42E+01	9.36E+01	9.58E+01
2245.736	9.43E+01	9.37E+01	9.59E+01
2246.219	9.44E+01	9.38E+01	9.60E+01
2246.701	9.46E+01	9.41E+01	9.62E+01
2247.183	9.48E+01	9.44E+01	9.64E+01
2247.665	9.50E+01	9.47E+01	9.66E+01
2248.147	9.52E+01	9.50E+01	9.68E+01
2248.629	9.55E+01	9.52E+01	9.69E+01
2249.111	9.56E+01	9.54E+01	9.70E+01
2249.594	9.58E+01	9.55E+01	9.70E+01
2250.075	9.59E+01	9.56E+01	9.70E+01
2250.558	9.60E+01	9.56E+01	9.69E+01
2251.04	9.60E+01	9.56E+01	9.69E+01
2251.522	9.61E+01	9.56E+01	9.69E+01
2252.004	9.61E+01	9.57E+01	9.70E+01
2252.486	9.62E+01	9.58E+01	9.71E+01
2252.968	9.62E+01	9.59E+01	9.72E+01
2253.45	9.63E+01	9.61E+01	9.74E+01
2253.933	9.64E+01	9.62E+01	9.76E+01
2254.415	9.65E+01	9.64E+01	9.77E+01
2254.897	9.66E+01	9.65E+01	9.79E+01
2255.379	9.67E+01	9.66E+01	9.80E+01
2255.861	9.67E+01	9.67E+01	9.80E+01
2256.343	9.67E+01	9.67E+01	9.79E+01
2256.825	9.68E+01	9.67E+01	9.78E+01

2257.307	9.67E+01	9.68E+01	9.77E+01
2257.79	9.68E+01	9.68E+01	9.76E+01
2258.272	9.68E+01	9.69E+01	9.75E+01
2258.754	9.68E+01	9.70E+01	9.75E+01
2259.236	9.68E+01	9.70E+01	9.75E+01
2259.718	9.68E+01	9.71E+01	9.76E+01
2260.2	9.68E+01	9.71E+01	9.77E+01
2260.682	9.68E+01	9.70E+01	9.78E+01
2261.164	9.68E+01	9.70E+01	9.79E+01
2261.646	9.68E+01	9.69E+01	9.80E+01
2262.129	9.69E+01	9.69E+01	9.80E+01
2262.611	9.69E+01	9.69E+01	9.80E+01
2263.093	9.70E+01	9.69E+01	9.80E+01
2263.575	9.70E+01	9.69E+01	9.79E+01
2264.057	9.71E+01	9.69E+01	9.78E+01
2264.539	9.71E+01	9.68E+01	9.77E+01
2265.021	9.70E+01	9.68E+01	9.76E+01
2265.503	9.70E+01	9.67E+01	9.75E+01
2265.986	9.70E+01	9.66E+01	9.75E+01
2266.468	9.69E+01	9.66E+01	9.75E+01
2266.95	9.69E+01	9.67E+01	9.76E+01
2267.432	9.69E+01	9.67E+01	9.76E+01
2267.914	9.69E+01	9.69E+01	9.78E+01
2268.396	9.69E+01	9.70E+01	9.79E+01
2268.878	9.69E+01	9.71E+01	9.80E+01
2269.361	9.70E+01	9.71E+01	9.80E+01
2269.843	9.70E+01	9.72E+01	9.81E+01
2270.325	9.70E+01	9.72E+01	9.81E+01
2270.807	9.70E+01	9.72E+01	9.81E+01
2271.289	9.70E+01	9.72E+01	9.81E+01
2271.771	9.69E+01	9.72E+01	9.81E+01
2272.253	9.68E+01	9.71E+01	9.80E+01
2272.735	9.68E+01	9.71E+01	9.80E+01
2273.218	9.67E+01	9.70E+01	9.79E+01
2273.7	9.67E+01	9.69E+01	9.79E+01
2274.182	9.67E+01	9.68E+01	9.79E+01
2274.664	9.67E+01	9.67E+01	9.78E+01
2275.146	9.67E+01	9.66E+01	9.78E+01
2275.628	9.67E+01	9.66E+01	9.78E+01
2276.11	9.67E+01	9.65E+01	9.78E+01
2276.592	9.67E+01	9.64E+01	9.78E+01
2277.074	9.67E+01	9.64E+01	9.78E+01
2277.557	9.67E+01	9.64E+01	9.77E+01
2278.039	9.67E+01	9.64E+01	9.77E+01
2278.521	9.67E+01	9.64E+01	9.77E+01
2279.003	9.67E+01	9.64E+01	9.77E+01
2279.485	9.67E+01	9.64E+01	9.77E+01

2279.967	9.67E+01	9.64E+01	9.77E+01
2280.449	9.67E+01	9.64E+01	9.78E+01
2280.931	9.68E+01	9.64E+01	9.78E+01
2281.414	9.68E+01	9.65E+01	9.79E+01
2281.896	9.68E+01	9.65E+01	9.79E+01
2282.378	9.69E+01	9.64E+01	9.79E+01
2282.86	9.69E+01	9.64E+01	9.78E+01
2283.342	9.69E+01	9.64E+01	9.78E+01
2283.824	9.68E+01	9.64E+01	9.78E+01
2284.306	9.68E+01	9.64E+01	9.77E+01
2284.789	9.68E+01	9.65E+01	9.77E+01
2285.271	9.68E+01	9.65E+01	9.77E+01
2285.753	9.68E+01	9.66E+01	9.78E+01
2286.235	9.68E+01	9.66E+01	9.78E+01
2286.717	9.68E+01	9.66E+01	9.79E+01
2287.199	9.69E+01	9.66E+01	9.79E+01
2287.681	9.69E+01	9.66E+01	9.79E+01
2288.163	9.69E+01	9.66E+01	9.79E+01
2288.646	9.69E+01	9.65E+01	9.78E+01
2289.128	9.68E+01	9.65E+01	9.77E+01
2289.61	9.68E+01	9.64E+01	9.76E+01
2290.092	9.68E+01	9.64E+01	9.76E+01
2290.574	9.69E+01	9.64E+01	9.75E+01
2291.056	9.69E+01	9.64E+01	9.74E+01
2291.538	9.69E+01	9.64E+01	9.74E+01
2292.02	9.68E+01	9.64E+01	9.74E+01
2292.502	9.68E+01	9.64E+01	9.74E+01
2292.985	9.68E+01	9.64E+01	9.75E+01
2293.467	9.68E+01	9.65E+01	9.75E+01
2293.949	9.68E+01	9.65E+01	9.75E+01
2294.431	9.69E+01	9.65E+01	9.76E+01
2294.913	9.69E+01	9.66E+01	9.76E+01
2295.395	9.69E+01	9.66E+01	9.77E+01
2295.877	9.69E+01	9.66E+01	9.77E+01
2296.36	9.70E+01	9.66E+01	9.78E+01
2296.842	9.70E+01	9.67E+01	9.78E+01
2297.324	9.69E+01	9.67E+01	9.78E+01
2297.806	9.69E+01	9.67E+01	9.78E+01
2298.288	9.69E+01	9.67E+01	9.78E+01
2298.77	9.68E+01	9.67E+01	9.78E+01
2299.252	9.68E+01	9.67E+01	9.78E+01
2299.734	9.68E+01	9.67E+01	9.77E+01
2300.217	9.68E+01	9.67E+01	9.77E+01
2300.699	9.68E+01	9.67E+01	9.77E+01
2301.181	9.68E+01	9.67E+01	9.77E+01
2301.663	9.68E+01	9.67E+01	9.76E+01
2302.145	9.68E+01	9.67E+01	9.76E+01

2302.627	9.67E+01	9.67E+01	9.76E+01
2303.109	9.67E+01	9.67E+01	9.75E+01
2303.591	9.66E+01	9.67E+01	9.75E+01
2304.073	9.65E+01	9.66E+01	9.74E+01
2304.556	9.65E+01	9.66E+01	9.74E+01
2305.038	9.65E+01	9.66E+01	9.74E+01
2305.52	9.65E+01	9.65E+01	9.74E+01
2306.002	9.66E+01	9.66E+01	9.74E+01
2306.484	9.66E+01	9.66E+01	9.75E+01
2306.966	9.67E+01	9.66E+01	9.76E+01
2307.448	9.68E+01	9.66E+01	9.76E+01
2307.93	9.68E+01	9.67E+01	9.77E+01
2308.413	9.69E+01	9.67E+01	9.78E+01
2308.895	9.69E+01	9.67E+01	9.78E+01
2309.377	9.69E+01	9.66E+01	9.78E+01
2309.859	9.69E+01	9.66E+01	9.78E+01
2310.341	9.68E+01	9.65E+01	9.77E+01
2310.823	9.67E+01	9.64E+01	9.76E+01
2311.305	9.67E+01	9.64E+01	9.76E+01
2311.788	9.66E+01	9.63E+01	9.75E+01
2312.27	9.65E+01	9.63E+01	9.74E+01
2312.752	9.65E+01	9.63E+01	9.74E+01
2313.234	9.65E+01	9.64E+01	9.74E+01
2313.716	9.66E+01	9.64E+01	9.74E+01
2314.198	9.66E+01	9.65E+01	9.75E+01
2314.68	9.67E+01	9.65E+01	9.75E+01
2315.162	9.67E+01	9.66E+01	9.75E+01
2315.645	9.67E+01	9.66E+01	9.75E+01
2316.127	9.68E+01	9.66E+01	9.75E+01
2316.609	9.68E+01	9.66E+01	9.75E+01
2317.091	9.68E+01	9.67E+01	9.75E+01
2317.573	9.68E+01	9.67E+01	9.75E+01
2318.055	9.68E+01	9.66E+01	9.75E+01
2318.537	9.67E+01	9.66E+01	9.75E+01
2319.019	9.67E+01	9.66E+01	9.76E+01
2319.501	9.66E+01	9.66E+01	9.76E+01
2319.984	9.65E+01	9.66E+01	9.76E+01
2320.466	9.65E+01	9.66E+01	9.75E+01
2320.948	9.65E+01	9.66E+01	9.76E+01
2321.43	9.65E+01	9.66E+01	9.76E+01
2321.912	9.65E+01	9.65E+01	9.76E+01
2322.394	9.65E+01	9.65E+01	9.76E+01
2322.876	9.66E+01	9.65E+01	9.76E+01
2323.358	9.66E+01	9.65E+01	9.77E+01
2323.841	9.67E+01	9.65E+01	9.77E+01
2324.323	9.67E+01	9.65E+01	9.77E+01
2324.805	9.67E+01	9.65E+01	9.77E+01

2325.287	9.67E+01	9.65E+01	9.77E+01
2325.769	9.67E+01	9.65E+01	9.77E+01
2326.251	9.67E+01	9.64E+01	9.77E+01
2326.733	9.66E+01	9.64E+01	9.76E+01
2327.216	9.66E+01	9.64E+01	9.76E+01
2327.698	9.66E+01	9.63E+01	9.76E+01
2328.18	9.66E+01	9.63E+01	9.76E+01
2328.662	9.66E+01	9.62E+01	9.76E+01
2329.144	9.66E+01	9.62E+01	9.76E+01
2329.626	9.66E+01	9.62E+01	9.76E+01
2330.108	9.67E+01	9.63E+01	9.77E+01
2330.59	9.67E+01	9.63E+01	9.77E+01
2331.073	9.67E+01	9.63E+01	9.78E+01
2331.555	9.68E+01	9.63E+01	9.78E+01
2332.037	9.68E+01	9.63E+01	9.78E+01
2332.519	9.68E+01	9.62E+01	9.78E+01
2333.001	9.68E+01	9.62E+01	9.78E+01
2333.483	9.68E+01	9.62E+01	9.78E+01
2333.965	9.68E+01	9.62E+01	9.77E+01
2334.447	9.68E+01	9.62E+01	9.77E+01
2334.929	9.67E+01	9.62E+01	9.76E+01
2335.412	9.67E+01	9.62E+01	9.76E+01
2335.894	9.66E+01	9.62E+01	9.75E+01
2336.376	9.66E+01	9.63E+01	9.75E+01
2336.858	9.66E+01	9.63E+01	9.75E+01
2337.34	9.66E+01	9.63E+01	9.76E+01
2337.822	9.66E+01	9.64E+01	9.76E+01
2338.304	9.66E+01	9.64E+01	9.76E+01
2338.786	9.67E+01	9.64E+01	9.76E+01
2339.269	9.67E+01	9.65E+01	9.77E+01
2339.751	9.67E+01	9.65E+01	9.77E+01
2340.233	9.67E+01	9.65E+01	9.77E+01
2340.715	9.67E+01	9.66E+01	9.76E+01
2341.197	9.68E+01	9.66E+01	9.76E+01
2341.679	9.68E+01	9.66E+01	9.76E+01
2342.161	9.68E+01	9.66E+01	9.75E+01
2342.644	9.69E+01	9.65E+01	9.75E+01
2343.125	9.69E+01	9.65E+01	9.75E+01
2343.608	9.69E+01	9.64E+01	9.74E+01
2344.09	9.69E+01	9.64E+01	9.74E+01
2344.572	9.69E+01	9.64E+01	9.74E+01
2345.054	9.68E+01	9.63E+01	9.74E+01
2345.536	9.68E+01	9.63E+01	9.74E+01
2346.018	9.67E+01	9.63E+01	9.74E+01
2346.5	9.67E+01	9.64E+01	9.74E+01
2346.983	9.66E+01	9.64E+01	9.74E+01
2347.465	9.66E+01	9.64E+01	9.74E+01

2347.947	9.65E+01	9.64E+01	9.74E+01
2348.429	9.65E+01	9.64E+01	9.74E+01
2348.911	9.65E+01	9.64E+01	9.74E+01
2349.393	9.65E+01	9.64E+01	9.74E+01
2349.875	9.65E+01	9.63E+01	9.74E+01
2350.357	9.66E+01	9.63E+01	9.74E+01
2350.84	9.66E+01	9.63E+01	9.74E+01
2351.322	9.67E+01	9.63E+01	9.74E+01
2351.804	9.67E+01	9.63E+01	9.74E+01
2352.286	9.68E+01	9.64E+01	9.74E+01
2352.768	9.68E+01	9.64E+01	9.75E+01
2353.25	9.69E+01	9.64E+01	9.75E+01
2353.732	9.69E+01	9.65E+01	9.75E+01
2354.214	9.69E+01	9.65E+01	9.75E+01
2354.697	9.70E+01	9.65E+01	9.76E+01
2355.179	9.70E+01	9.64E+01	9.75E+01
2355.661	9.70E+01	9.64E+01	9.75E+01
2356.143	9.70E+01	9.64E+01	9.75E+01
2356.625	9.70E+01	9.63E+01	9.75E+01
2357.107	9.70E+01	9.63E+01	9.75E+01
2357.589	9.69E+01	9.63E+01	9.74E+01
2358.072	9.68E+01	9.63E+01	9.74E+01
2358.553	9.67E+01	9.63E+01	9.74E+01
2359.036	9.66E+01	9.64E+01	9.73E+01
2359.518	9.66E+01	9.64E+01	9.73E+01
2360	9.65E+01	9.65E+01	9.74E+01
2360.482	9.66E+01	9.65E+01	9.74E+01
2360.964	9.66E+01	9.66E+01	9.76E+01
2361.446	9.67E+01	9.66E+01	9.77E+01
2361.928	9.68E+01	9.66E+01	9.78E+01
2362.411	9.69E+01	9.67E+01	9.79E+01
2362.893	9.69E+01	9.66E+01	9.79E+01
2363.375	9.69E+01	9.66E+01	9.79E+01
2363.857	9.69E+01	9.66E+01	9.78E+01
2364.339	9.69E+01	9.65E+01	9.77E+01
2364.821	9.68E+01	9.65E+01	9.77E+01
2365.303	9.67E+01	9.65E+01	9.76E+01
2365.785	9.67E+01	9.65E+01	9.75E+01
2366.268	9.67E+01	9.65E+01	9.75E+01
2366.75	9.67E+01	9.65E+01	9.75E+01
2367.232	9.67E+01	9.65E+01	9.75E+01
2367.714	9.67E+01	9.65E+01	9.75E+01
2368.196	9.67E+01	9.64E+01	9.74E+01
2368.678	9.67E+01	9.64E+01	9.74E+01
2369.16	9.66E+01	9.64E+01	9.74E+01
2369.642	9.66E+01	9.63E+01	9.74E+01
2370.125	9.65E+01	9.63E+01	9.73E+01

2370.607	9.64E+01	9.63E+01	9.73E+01
2371.089	9.64E+01	9.62E+01	9.73E+01
2371.571	9.64E+01	9.63E+01	9.73E+01
2372.053	9.64E+01	9.63E+01	9.73E+01
2372.535	9.64E+01	9.63E+01	9.74E+01
2373.017	9.65E+01	9.64E+01	9.75E+01
2373.5	9.66E+01	9.65E+01	9.76E+01
2373.981	9.67E+01	9.65E+01	9.76E+01
2374.464	9.67E+01	9.65E+01	9.77E+01
2374.946	9.68E+01	9.65E+01	9.77E+01
2375.428	9.68E+01	9.65E+01	9.77E+01
2375.91	9.68E+01	9.65E+01	9.76E+01
2376.392	9.67E+01	9.64E+01	9.76E+01
2376.874	9.67E+01	9.64E+01	9.75E+01
2377.356	9.66E+01	9.63E+01	9.75E+01
2377.839	9.66E+01	9.63E+01	9.74E+01
2378.321	9.65E+01	9.63E+01	9.74E+01
2378.803	9.65E+01	9.63E+01	9.74E+01
2379.285	9.65E+01	9.63E+01	9.74E+01
2379.767	9.65E+01	9.63E+01	9.74E+01
2380.249	9.65E+01	9.63E+01	9.74E+01
2380.731	9.65E+01	9.64E+01	9.74E+01
2381.213	9.66E+01	9.64E+01	9.74E+01
2381.696	9.66E+01	9.64E+01	9.74E+01
2382.178	9.66E+01	9.64E+01	9.74E+01
2382.66	9.66E+01	9.64E+01	9.74E+01
2383.142	9.66E+01	9.64E+01	9.74E+01
2383.624	9.66E+01	9.64E+01	9.74E+01
2384.106	9.65E+01	9.63E+01	9.74E+01
2384.588	9.65E+01	9.62E+01	9.73E+01
2385.07	9.65E+01	9.62E+01	9.73E+01
2385.552	9.64E+01	9.62E+01	9.74E+01
2386.035	9.64E+01	9.61E+01	9.74E+01
2386.517	9.64E+01	9.62E+01	9.75E+01
2386.999	9.65E+01	9.62E+01	9.75E+01
2387.481	9.65E+01	9.63E+01	9.76E+01
2387.963	9.66E+01	9.63E+01	9.77E+01
2388.445	9.66E+01	9.64E+01	9.77E+01
2388.927	9.66E+01	9.64E+01	9.77E+01
2389.41	9.66E+01	9.64E+01	9.77E+01
2389.892	9.66E+01	9.64E+01	9.77E+01
2390.374	9.66E+01	9.64E+01	9.77E+01
2390.856	9.66E+01	9.64E+01	9.76E+01
2391.338	9.65E+01	9.64E+01	9.76E+01
2391.82	9.65E+01	9.63E+01	9.75E+01
2392.302	9.65E+01	9.63E+01	9.75E+01
2392.784	9.65E+01	9.63E+01	9.75E+01

2393.267	9.65E+01	9.62E+01	9.75E+01
2393.749	9.65E+01	9.62E+01	9.74E+01
2394.231	9.66E+01	9.62E+01	9.75E+01
2394.713	9.66E+01	9.62E+01	9.75E+01
2395.195	9.66E+01	9.63E+01	9.75E+01
2395.677	9.66E+01	9.63E+01	9.75E+01
2396.159	9.66E+01	9.63E+01	9.75E+01
2396.641	9.66E+01	9.63E+01	9.75E+01
2397.124	9.65E+01	9.63E+01	9.74E+01
2397.606	9.65E+01	9.63E+01	9.74E+01
2398.088	9.65E+01	9.63E+01	9.74E+01
2398.57	9.64E+01	9.63E+01	9.74E+01
2399.052	9.64E+01	9.63E+01	9.74E+01
2399.534	9.64E+01	9.63E+01	9.74E+01
2400.016	9.64E+01	9.62E+01	9.74E+01
2400.498	9.64E+01	9.62E+01	9.74E+01
2400.98	9.64E+01	9.62E+01	9.74E+01
2401.463	9.64E+01	9.62E+01	9.74E+01
2401.945	9.64E+01	9.62E+01	9.74E+01
2402.427	9.64E+01	9.62E+01	9.75E+01
2402.909	9.64E+01	9.63E+01	9.75E+01
2403.391	9.64E+01	9.63E+01	9.75E+01
2403.873	9.64E+01	9.63E+01	9.75E+01
2404.355	9.64E+01	9.63E+01	9.76E+01
2404.838	9.64E+01	9.63E+01	9.76E+01
2405.32	9.65E+01	9.63E+01	9.76E+01
2405.802	9.65E+01	9.63E+01	9.76E+01
2406.284	9.65E+01	9.63E+01	9.76E+01
2406.766	9.65E+01	9.63E+01	9.76E+01
2407.248	9.65E+01	9.63E+01	9.76E+01
2407.73	9.65E+01	9.63E+01	9.76E+01
2408.212	9.65E+01	9.64E+01	9.76E+01
2408.695	9.65E+01	9.64E+01	9.76E+01
2409.177	9.66E+01	9.64E+01	9.75E+01
2409.659	9.66E+01	9.64E+01	9.75E+01
2410.141	9.66E+01	9.64E+01	9.75E+01
2410.623	9.66E+01	9.64E+01	9.75E+01
2411.105	9.66E+01	9.64E+01	9.74E+01
2411.587	9.66E+01	9.63E+01	9.74E+01
2412.069	9.66E+01	9.63E+01	9.74E+01
2412.552	9.65E+01	9.63E+01	9.74E+01
2413.034	9.65E+01	9.63E+01	9.75E+01
2413.516	9.65E+01	9.63E+01	9.75E+01
2413.998	9.66E+01	9.63E+01	9.75E+01
2414.48	9.66E+01	9.63E+01	9.75E+01
2414.962	9.66E+01	9.63E+01	9.75E+01
2415.444	9.66E+01	9.63E+01	9.75E+01

2415.927	9.66E+01	9.62E+01	9.74E+01
2416.408	9.66E+01	9.62E+01	9.74E+01
2416.891	9.65E+01	9.62E+01	9.74E+01
2417.373	9.65E+01	9.62E+01	9.74E+01
2417.855	9.65E+01	9.62E+01	9.74E+01
2418.337	9.65E+01	9.63E+01	9.74E+01
2418.819	9.65E+01	9.63E+01	9.74E+01
2419.301	9.65E+01	9.63E+01	9.74E+01
2419.783	9.64E+01	9.63E+01	9.74E+01
2420.266	9.64E+01	9.63E+01	9.74E+01
2420.748	9.64E+01	9.63E+01	9.74E+01
2421.23	9.64E+01	9.63E+01	9.74E+01
2421.712	9.64E+01	9.62E+01	9.74E+01
2422.194	9.64E+01	9.62E+01	9.74E+01
2422.676	9.64E+01	9.62E+01	9.74E+01
2423.158	9.65E+01	9.62E+01	9.74E+01
2423.64	9.65E+01	9.62E+01	9.74E+01
2424.123	9.65E+01	9.62E+01	9.74E+01
2424.605	9.65E+01	9.62E+01	9.75E+01
2425.087	9.66E+01	9.62E+01	9.75E+01
2425.569	9.66E+01	9.62E+01	9.75E+01
2426.051	9.66E+01	9.62E+01	9.75E+01
2426.533	9.66E+01	9.62E+01	9.74E+01
2427.015	9.66E+01	9.62E+01	9.74E+01
2427.497	9.66E+01	9.62E+01	9.74E+01
2427.979	9.65E+01	9.62E+01	9.73E+01
2428.462	9.65E+01	9.62E+01	9.73E+01
2428.944	9.64E+01	9.61E+01	9.73E+01
2429.426	9.64E+01	9.61E+01	9.72E+01
2429.908	9.63E+01	9.61E+01	9.72E+01
2430.39	9.63E+01	9.61E+01	9.72E+01
2430.872	9.63E+01	9.61E+01	9.71E+01
2431.354	9.63E+01	9.60E+01	9.71E+01
2431.836	9.63E+01	9.60E+01	9.71E+01
2432.319	9.63E+01	9.60E+01	9.71E+01
2432.801	9.64E+01	9.61E+01	9.72E+01
2433.283	9.64E+01	9.61E+01	9.72E+01
2433.765	9.64E+01	9.61E+01	9.73E+01
2434.247	9.65E+01	9.61E+01	9.73E+01
2434.729	9.65E+01	9.61E+01	9.73E+01
2435.211	9.65E+01	9.61E+01	9.73E+01
2435.694	9.65E+01	9.60E+01	9.73E+01
2436.176	9.65E+01	9.60E+01	9.73E+01
2436.658	9.65E+01	9.60E+01	9.73E+01
2437.14	9.65E+01	9.59E+01	9.72E+01
2437.622	9.65E+01	9.60E+01	9.72E+01
2438.104	9.66E+01	9.60E+01	9.73E+01

2438.586	9.66E+01	9.60E+01	9.73E+01
2439.068	9.66E+01	9.60E+01	9.73E+01
2439.551	9.66E+01	9.60E+01	9.74E+01
2440.033	9.66E+01	9.60E+01	9.74E+01
2440.515	9.67E+01	9.61E+01	9.75E+01
2440.997	9.67E+01	9.61E+01	9.75E+01
2441.479	9.67E+01	9.61E+01	9.75E+01
2441.961	9.67E+01	9.61E+01	9.75E+01
2442.443	9.67E+01	9.61E+01	9.75E+01
2442.925	9.68E+01	9.62E+01	9.75E+01
2443.407	9.68E+01	9.62E+01	9.74E+01
2443.89	9.67E+01	9.63E+01	9.74E+01
2444.372	9.67E+01	9.63E+01	9.73E+01
2444.854	9.67E+01	9.63E+01	9.73E+01
2445.336	9.66E+01	9.64E+01	9.73E+01
2445.818	9.66E+01	9.64E+01	9.73E+01
2446.3	9.65E+01	9.63E+01	9.73E+01
2446.782	9.65E+01	9.63E+01	9.72E+01
2447.264	9.64E+01	9.63E+01	9.72E+01
2447.747	9.64E+01	9.62E+01	9.72E+01
2448.229	9.64E+01	9.62E+01	9.72E+01
2448.711	9.64E+01	9.61E+01	9.72E+01
2449.193	9.64E+01	9.61E+01	9.72E+01
2449.675	9.64E+01	9.61E+01	9.72E+01
2450.157	9.65E+01	9.61E+01	9.73E+01
2450.639	9.65E+01	9.60E+01	9.74E+01
2451.122	9.65E+01	9.60E+01	9.75E+01
2451.604	9.66E+01	9.60E+01	9.75E+01
2452.086	9.66E+01	9.60E+01	9.76E+01
2452.568	9.66E+01	9.60E+01	9.76E+01
2453.05	9.66E+01	9.59E+01	9.75E+01
2453.532	9.66E+01	9.59E+01	9.75E+01
2454.014	9.66E+01	9.59E+01	9.75E+01
2454.496	9.65E+01	9.59E+01	9.74E+01
2454.979	9.65E+01	9.60E+01	9.74E+01
2455.461	9.65E+01	9.60E+01	9.74E+01
2455.943	9.65E+01	9.61E+01	9.73E+01
2456.425	9.65E+01	9.61E+01	9.73E+01
2456.907	9.65E+01	9.62E+01	9.74E+01
2457.389	9.65E+01	9.62E+01	9.74E+01
2457.871	9.65E+01	9.62E+01	9.74E+01
2458.353	9.65E+01	9.62E+01	9.74E+01
2458.835	9.65E+01	9.61E+01	9.74E+01
2459.318	9.64E+01	9.61E+01	9.74E+01
2459.8	9.64E+01	9.61E+01	9.74E+01
2460.282	9.64E+01	9.61E+01	9.73E+01
2460.764	9.64E+01	9.61E+01	9.73E+01

2461.246	9.64E+01	9.61E+01	9.73E+01
2461.728	9.64E+01	9.61E+01	9.73E+01
2462.21	9.65E+01	9.61E+01	9.73E+01
2462.692	9.65E+01	9.62E+01	9.73E+01
2463.175	9.65E+01	9.62E+01	9.73E+01
2463.657	9.65E+01	9.62E+01	9.73E+01
2464.139	9.66E+01	9.62E+01	9.73E+01
2464.621	9.66E+01	9.61E+01	9.74E+01
2465.103	9.66E+01	9.61E+01	9.74E+01
2465.585	9.65E+01	9.60E+01	9.74E+01
2466.067	9.65E+01	9.60E+01	9.74E+01
2466.55	9.65E+01	9.60E+01	9.74E+01
2467.031	9.65E+01	9.59E+01	9.75E+01
2467.514	9.65E+01	9.60E+01	9.75E+01
2467.996	9.65E+01	9.60E+01	9.74E+01
2468.478	9.65E+01	9.60E+01	9.74E+01
2468.96	9.65E+01	9.60E+01	9.74E+01
2469.442	9.65E+01	9.61E+01	9.74E+01
2469.924	9.64E+01	9.61E+01	9.73E+01
2470.406	9.64E+01	9.61E+01	9.73E+01
2470.889	9.64E+01	9.61E+01	9.73E+01
2471.371	9.63E+01	9.60E+01	9.73E+01
2471.853	9.63E+01	9.60E+01	9.73E+01
2472.335	9.63E+01	9.60E+01	9.73E+01
2472.817	9.63E+01	9.60E+01	9.73E+01
2473.299	9.63E+01	9.60E+01	9.74E+01
2473.781	9.64E+01	9.60E+01	9.74E+01
2474.263	9.64E+01	9.61E+01	9.74E+01
2474.746	9.64E+01	9.61E+01	9.74E+01
2475.228	9.65E+01	9.61E+01	9.75E+01
2475.71	9.65E+01	9.62E+01	9.75E+01
2476.192	9.65E+01	9.62E+01	9.75E+01
2476.674	9.65E+01	9.62E+01	9.76E+01
2477.156	9.65E+01	9.62E+01	9.76E+01
2477.638	9.65E+01	9.62E+01	9.76E+01
2478.12	9.65E+01	9.61E+01	9.76E+01
2478.603	9.65E+01	9.61E+01	9.76E+01
2479.085	9.65E+01	9.60E+01	9.75E+01
2479.567	9.65E+01	9.59E+01	9.75E+01
2480.049	9.65E+01	9.59E+01	9.74E+01
2480.531	9.65E+01	9.59E+01	9.74E+01
2481.013	9.64E+01	9.58E+01	9.74E+01
2481.495	9.64E+01	9.58E+01	9.74E+01
2481.978	9.64E+01	9.58E+01	9.74E+01
2482.459	9.64E+01	9.59E+01	9.74E+01
2482.942	9.64E+01	9.59E+01	9.74E+01
2483.424	9.64E+01	9.59E+01	9.73E+01

2483.906	9.64E+01	9.60E+01	9.73E+01
2484.388	9.65E+01	9.60E+01	9.73E+01
2484.87	9.66E+01	9.60E+01	9.73E+01
2485.352	9.66E+01	9.60E+01	9.73E+01
2485.834	9.66E+01	9.60E+01	9.72E+01
2486.317	9.66E+01	9.60E+01	9.72E+01
2486.799	9.65E+01	9.60E+01	9.72E+01
2487.281	9.65E+01	9.60E+01	9.71E+01
2487.763	9.64E+01	9.60E+01	9.71E+01
2488.245	9.63E+01	9.60E+01	9.71E+01
2488.727	9.63E+01	9.60E+01	9.71E+01
2489.209	9.62E+01	9.60E+01	9.71E+01
2489.691	9.62E+01	9.60E+01	9.71E+01
2490.174	9.62E+01	9.59E+01	9.72E+01
2490.656	9.62E+01	9.59E+01	9.72E+01
2491.138	9.62E+01	9.59E+01	9.72E+01
2491.62	9.62E+01	9.58E+01	9.73E+01
2492.102	9.62E+01	9.58E+01	9.73E+01
2492.584	9.63E+01	9.58E+01	9.74E+01
2493.066	9.63E+01	9.58E+01	9.74E+01
2493.548	9.63E+01	9.59E+01	9.74E+01
2494.031	9.63E+01	9.59E+01	9.74E+01
2494.513	9.63E+01	9.59E+01	9.74E+01
2494.995	9.64E+01	9.60E+01	9.74E+01
2495.477	9.64E+01	9.60E+01	9.74E+01
2495.959	9.64E+01	9.60E+01	9.74E+01
2496.441	9.64E+01	9.61E+01	9.74E+01
2496.923	9.63E+01	9.60E+01	9.73E+01
2497.406	9.63E+01	9.60E+01	9.73E+01
2497.888	9.62E+01	9.60E+01	9.72E+01
2498.37	9.62E+01	9.59E+01	9.72E+01
2498.852	9.61E+01	9.58E+01	9.71E+01
2499.334	9.61E+01	9.58E+01	9.70E+01
2499.816	9.61E+01	9.58E+01	9.70E+01
2500.298	9.61E+01	9.58E+01	9.70E+01
2500.78	9.61E+01	9.58E+01	9.70E+01
2501.262	9.62E+01	9.58E+01	9.71E+01
2501.745	9.62E+01	9.58E+01	9.71E+01
2502.227	9.63E+01	9.58E+01	9.72E+01
2502.709	9.63E+01	9.58E+01	9.72E+01
2503.191	9.63E+01	9.58E+01	9.73E+01
2503.673	9.63E+01	9.58E+01	9.73E+01
2504.155	9.63E+01	9.58E+01	9.73E+01
2504.637	9.63E+01	9.59E+01	9.73E+01
2505.119	9.63E+01	9.59E+01	9.73E+01
2505.602	9.63E+01	9.59E+01	9.73E+01
2506.084	9.63E+01	9.59E+01	9.72E+01

2506.566	9.63E+01	9.60E+01	9.72E+01
2507.048	9.63E+01	9.60E+01	9.72E+01
2507.53	9.63E+01	9.60E+01	9.72E+01
2508.012	9.63E+01	9.60E+01	9.72E+01
2508.494	9.63E+01	9.59E+01	9.72E+01
2508.977	9.63E+01	9.59E+01	9.72E+01
2509.458	9.64E+01	9.59E+01	9.72E+01
2509.941	9.64E+01	9.59E+01	9.72E+01
2510.423	9.64E+01	9.59E+01	9.72E+01
2510.905	9.64E+01	9.59E+01	9.72E+01
2511.387	9.64E+01	9.60E+01	9.72E+01
2511.869	9.64E+01	9.60E+01	9.72E+01
2512.351	9.63E+01	9.60E+01	9.72E+01
2512.833	9.63E+01	9.61E+01	9.72E+01
2513.316	9.63E+01	9.61E+01	9.72E+01
2513.798	9.63E+01	9.61E+01	9.72E+01
2514.28	9.63E+01	9.61E+01	9.73E+01
2514.762	9.63E+01	9.62E+01	9.73E+01
2515.244	9.63E+01	9.62E+01	9.73E+01
2515.726	9.64E+01	9.62E+01	9.74E+01
2516.208	9.64E+01	9.62E+01	9.74E+01
2516.69	9.64E+01	9.63E+01	9.74E+01
2517.173	9.65E+01	9.63E+01	9.75E+01
2517.655	9.65E+01	9.63E+01	9.75E+01
2518.137	9.65E+01	9.62E+01	9.75E+01
2518.619	9.65E+01	9.62E+01	9.76E+01
2519.101	9.65E+01	9.61E+01	9.75E+01
2519.583	9.64E+01	9.60E+01	9.75E+01
2520.065	9.64E+01	9.59E+01	9.75E+01
2520.547	9.63E+01	9.57E+01	9.74E+01
2521.03	9.63E+01	9.56E+01	9.74E+01
2521.512	9.62E+01	9.55E+01	9.73E+01
2521.994	9.62E+01	9.55E+01	9.72E+01
2522.476	9.61E+01	9.55E+01	9.72E+01
2522.958	9.61E+01	9.55E+01	9.71E+01
2523.44	9.60E+01	9.56E+01	9.71E+01
2523.922	9.60E+01	9.56E+01	9.71E+01
2524.405	9.60E+01	9.57E+01	9.71E+01
2524.886	9.60E+01	9.58E+01	9.71E+01
2525.369	9.61E+01	9.59E+01	9.71E+01
2525.851	9.61E+01	9.59E+01	9.72E+01
2526.333	9.62E+01	9.59E+01	9.72E+01
2526.815	9.62E+01	9.59E+01	9.72E+01
2527.297	9.62E+01	9.58E+01	9.72E+01
2527.779	9.62E+01	9.58E+01	9.72E+01
2528.261	9.62E+01	9.57E+01	9.72E+01
2528.744	9.62E+01	9.57E+01	9.71E+01

2529.226	9.62E+01	9.57E+01	9.71E+01
2529.708	9.62E+01	9.57E+01	9.70E+01
2530.19	9.62E+01	9.57E+01	9.70E+01
2530.672	9.62E+01	9.57E+01	9.70E+01
2531.154	9.62E+01	9.58E+01	9.70E+01
2531.636	9.62E+01	9.59E+01	9.71E+01
2532.118	9.62E+01	9.59E+01	9.72E+01
2532.601	9.63E+01	9.60E+01	9.72E+01
2533.083	9.63E+01	9.60E+01	9.73E+01
2533.565	9.63E+01	9.60E+01	9.73E+01
2534.047	9.63E+01	9.60E+01	9.73E+01
2534.529	9.63E+01	9.59E+01	9.73E+01
2535.011	9.63E+01	9.59E+01	9.73E+01
2535.493	9.63E+01	9.59E+01	9.73E+01
2535.975	9.63E+01	9.58E+01	9.73E+01
2536.458	9.63E+01	9.58E+01	9.72E+01
2536.94	9.62E+01	9.58E+01	9.72E+01
2537.422	9.62E+01	9.58E+01	9.72E+01
2537.904	9.62E+01	9.59E+01	9.72E+01
2538.386	9.62E+01	9.59E+01	9.72E+01
2538.868	9.62E+01	9.59E+01	9.72E+01
2539.35	9.61E+01	9.59E+01	9.72E+01
2539.833	9.61E+01	9.59E+01	9.72E+01
2540.314	9.61E+01	9.59E+01	9.71E+01
2540.797	9.62E+01	9.59E+01	9.71E+01
2541.279	9.62E+01	9.59E+01	9.71E+01
2541.761	9.62E+01	9.59E+01	9.72E+01
2542.243	9.62E+01	9.59E+01	9.72E+01
2542.725	9.63E+01	9.59E+01	9.72E+01
2543.207	9.63E+01	9.58E+01	9.72E+01
2543.689	9.63E+01	9.58E+01	9.72E+01
2544.172	9.63E+01	9.58E+01	9.72E+01
2544.654	9.63E+01	9.58E+01	9.72E+01
2545.136	9.63E+01	9.58E+01	9.72E+01
2545.618	9.64E+01	9.58E+01	9.72E+01
2546.1	9.64E+01	9.58E+01	9.72E+01
2546.582	9.64E+01	9.58E+01	9.72E+01
2547.064	9.64E+01	9.58E+01	9.72E+01
2547.546	9.64E+01	9.59E+01	9.73E+01
2548.029	9.64E+01	9.59E+01	9.73E+01
2548.511	9.64E+01	9.60E+01	9.74E+01
2548.993	9.65E+01	9.60E+01	9.74E+01
2549.475	9.64E+01	9.60E+01	9.74E+01
2549.957	9.64E+01	9.60E+01	9.74E+01
2550.439	9.64E+01	9.59E+01	9.74E+01
2550.921	9.63E+01	9.59E+01	9.73E+01
2551.403	9.63E+01	9.58E+01	9.73E+01

2551.885	9.62E+01	9.58E+01	9.72E+01
2552.368	9.62E+01	9.57E+01	9.72E+01
2552.85	9.62E+01	9.57E+01	9.72E+01
2553.332	9.62E+01	9.57E+01	9.72E+01
2553.814	9.62E+01	9.57E+01	9.72E+01
2554.296	9.62E+01	9.57E+01	9.72E+01
2554.778	9.63E+01	9.57E+01	9.72E+01
2555.26	9.63E+01	9.57E+01	9.73E+01
2555.742	9.63E+01	9.57E+01	9.73E+01
2556.225	9.63E+01	9.57E+01	9.72E+01
2556.707	9.63E+01	9.57E+01	9.72E+01
2557.189	9.63E+01	9.57E+01	9.72E+01
2557.671	9.63E+01	9.57E+01	9.71E+01
2558.153	9.63E+01	9.56E+01	9.71E+01
2558.635	9.63E+01	9.56E+01	9.71E+01
2559.117	9.64E+01	9.56E+01	9.71E+01
2559.6	9.64E+01	9.57E+01	9.71E+01
2560.082	9.64E+01	9.57E+01	9.71E+01
2560.564	9.64E+01	9.57E+01	9.71E+01
2561.046	9.64E+01	9.57E+01	9.71E+01
2561.528	9.64E+01	9.57E+01	9.71E+01
2562.01	9.64E+01	9.58E+01	9.71E+01
2562.492	9.64E+01	9.58E+01	9.71E+01
2562.974	9.63E+01	9.58E+01	9.71E+01
2563.457	9.63E+01	9.58E+01	9.71E+01
2563.939	9.63E+01	9.59E+01	9.71E+01
2564.421	9.63E+01	9.59E+01	9.71E+01
2564.903	9.63E+01	9.59E+01	9.72E+01
2565.385	9.63E+01	9.59E+01	9.72E+01
2565.867	9.63E+01	9.59E+01	9.72E+01
2566.349	9.63E+01	9.60E+01	9.72E+01
2566.831	9.63E+01	9.60E+01	9.72E+01
2567.313	9.63E+01	9.60E+01	9.72E+01
2567.796	9.63E+01	9.61E+01	9.73E+01
2568.278	9.63E+01	9.61E+01	9.73E+01
2568.76	9.63E+01	9.61E+01	9.73E+01
2569.242	9.64E+01	9.61E+01	9.74E+01
2569.724	9.64E+01	9.61E+01	9.74E+01
2570.206	9.64E+01	9.61E+01	9.74E+01
2570.688	9.63E+01	9.61E+01	9.74E+01
2571.17	9.63E+01	9.60E+01	9.74E+01
2571.653	9.63E+01	9.60E+01	9.74E+01
2572.135	9.63E+01	9.59E+01	9.74E+01
2572.617	9.63E+01	9.59E+01	9.73E+01
2573.099	9.62E+01	9.59E+01	9.73E+01
2573.581	9.62E+01	9.58E+01	9.73E+01
2574.063	9.62E+01	9.58E+01	9.72E+01

2574.545	9.62E+01	9.58E+01	9.72E+01
2575.028	9.62E+01	9.58E+01	9.72E+01
2575.51	9.62E+01	9.58E+01	9.72E+01
2575.992	9.62E+01	9.58E+01	9.72E+01
2576.474	9.62E+01	9.58E+01	9.72E+01
2576.956	9.62E+01	9.58E+01	9.72E+01
2577.438	9.62E+01	9.59E+01	9.72E+01
2577.92	9.62E+01	9.59E+01	9.71E+01
2578.402	9.63E+01	9.59E+01	9.71E+01
2578.885	9.63E+01	9.59E+01	9.71E+01
2579.367	9.63E+01	9.59E+01	9.71E+01
2579.849	9.63E+01	9.58E+01	9.71E+01
2580.331	9.63E+01	9.58E+01	9.72E+01
2580.813	9.63E+01	9.58E+01	9.72E+01
2581.295	9.63E+01	9.58E+01	9.72E+01
2581.777	9.63E+01	9.58E+01	9.72E+01
2582.259	9.63E+01	9.57E+01	9.72E+01
2582.741	9.63E+01	9.57E+01	9.72E+01
2583.224	9.63E+01	9.57E+01	9.72E+01
2583.706	9.63E+01	9.57E+01	9.72E+01
2584.188	9.62E+01	9.57E+01	9.72E+01
2584.67	9.62E+01	9.57E+01	9.72E+01
2585.152	9.62E+01	9.57E+01	9.72E+01
2585.634	9.62E+01	9.57E+01	9.72E+01
2586.116	9.62E+01	9.57E+01	9.72E+01
2586.598	9.62E+01	9.57E+01	9.71E+01
2587.081	9.62E+01	9.57E+01	9.71E+01
2587.563	9.62E+01	9.57E+01	9.71E+01
2588.045	9.62E+01	9.57E+01	9.71E+01
2588.527	9.62E+01	9.57E+01	9.71E+01
2589.009	9.61E+01	9.57E+01	9.71E+01
2589.491	9.61E+01	9.57E+01	9.71E+01
2589.973	9.61E+01	9.57E+01	9.71E+01
2590.456	9.61E+01	9.57E+01	9.71E+01
2590.938	9.61E+01	9.57E+01	9.71E+01
2591.42	9.61E+01	9.57E+01	9.72E+01
2591.902	9.61E+01	9.57E+01	9.72E+01
2592.384	9.61E+01	9.57E+01	9.72E+01
2592.866	9.61E+01	9.57E+01	9.72E+01
2593.348	9.61E+01	9.57E+01	9.72E+01
2593.83	9.62E+01	9.57E+01	9.72E+01
2594.313	9.62E+01	9.56E+01	9.72E+01
2594.795	9.62E+01	9.56E+01	9.72E+01
2595.277	9.61E+01	9.56E+01	9.72E+01
2595.759	9.61E+01	9.56E+01	9.72E+01
2596.241	9.61E+01	9.55E+01	9.71E+01
2596.723	9.61E+01	9.55E+01	9.71E+01

2597.205	9.61E+01	9.55E+01	9.71E+01
2597.687	9.61E+01	9.55E+01	9.71E+01
2598.169	9.61E+01	9.55E+01	9.71E+01
2598.652	9.61E+01	9.56E+01	9.71E+01
2599.134	9.61E+01	9.56E+01	9.72E+01
2599.616	9.61E+01	9.57E+01	9.72E+01
2600.098	9.62E+01	9.57E+01	9.72E+01
2600.58	9.62E+01	9.58E+01	9.72E+01
2601.062	9.62E+01	9.58E+01	9.72E+01
2601.544	9.62E+01	9.59E+01	9.72E+01
2602.027	9.62E+01	9.59E+01	9.72E+01
2602.509	9.62E+01	9.59E+01	9.72E+01
2602.991	9.62E+01	9.59E+01	9.71E+01
2603.473	9.62E+01	9.58E+01	9.71E+01
2603.955	9.62E+01	9.58E+01	9.71E+01
2604.437	9.62E+01	9.58E+01	9.71E+01
2604.919	9.62E+01	9.57E+01	9.71E+01
2605.401	9.62E+01	9.57E+01	9.71E+01
2605.884	9.62E+01	9.57E+01	9.71E+01
2606.366	9.62E+01	9.57E+01	9.71E+01
2606.848	9.62E+01	9.56E+01	9.71E+01
2607.33	9.62E+01	9.56E+01	9.72E+01
2607.812	9.62E+01	9.56E+01	9.72E+01
2608.294	9.61E+01	9.56E+01	9.72E+01
2608.776	9.61E+01	9.56E+01	9.72E+01
2609.258	9.61E+01	9.56E+01	9.72E+01
2609.74	9.61E+01	9.56E+01	9.72E+01
2610.223	9.61E+01	9.57E+01	9.72E+01
2610.705	9.61E+01	9.57E+01	9.72E+01
2611.187	9.62E+01	9.57E+01	9.71E+01
2611.669	9.62E+01	9.57E+01	9.71E+01
2612.151	9.62E+01	9.57E+01	9.71E+01
2612.633	9.62E+01	9.57E+01	9.71E+01
2613.115	9.63E+01	9.57E+01	9.71E+01
2613.597	9.63E+01	9.57E+01	9.71E+01
2614.08	9.63E+01	9.57E+01	9.71E+01
2614.562	9.63E+01	9.57E+01	9.71E+01
2615.044	9.63E+01	9.57E+01	9.71E+01
2615.526	9.62E+01	9.57E+01	9.71E+01
2616.008	9.62E+01	9.56E+01	9.71E+01
2616.49	9.62E+01	9.56E+01	9.71E+01
2616.972	9.62E+01	9.56E+01	9.71E+01
2617.455	9.61E+01	9.56E+01	9.72E+01
2617.937	9.61E+01	9.56E+01	9.71E+01
2618.419	9.61E+01	9.56E+01	9.71E+01
2618.901	9.61E+01	9.56E+01	9.71E+01
2619.383	9.60E+01	9.56E+01	9.71E+01

2619.865	9.60E+01	9.56E+01	9.71E+01
2620.347	9.61E+01	9.55E+01	9.71E+01
2620.829	9.61E+01	9.55E+01	9.71E+01
2621.312	9.61E+01	9.56E+01	9.71E+01
2621.794	9.61E+01	9.56E+01	9.71E+01
2622.276	9.62E+01	9.56E+01	9.71E+01
2622.758	9.62E+01	9.56E+01	9.72E+01
2623.24	9.62E+01	9.56E+01	9.71E+01
2623.722	9.62E+01	9.56E+01	9.71E+01
2624.204	9.62E+01	9.56E+01	9.71E+01
2624.686	9.61E+01	9.56E+01	9.71E+01
2625.168	9.61E+01	9.56E+01	9.71E+01
2625.651	9.61E+01	9.56E+01	9.71E+01
2626.133	9.61E+01	9.56E+01	9.71E+01
2626.615	9.61E+01	9.55E+01	9.71E+01
2627.097	9.60E+01	9.55E+01	9.71E+01
2627.579	9.60E+01	9.54E+01	9.70E+01
2628.061	9.60E+01	9.54E+01	9.70E+01
2628.543	9.59E+01	9.54E+01	9.70E+01
2629.025	9.59E+01	9.54E+01	9.69E+01
2629.508	9.59E+01	9.54E+01	9.69E+01
2629.99	9.59E+01	9.54E+01	9.69E+01
2630.472	9.59E+01	9.54E+01	9.69E+01
2630.954	9.59E+01	9.54E+01	9.69E+01
2631.436	9.59E+01	9.54E+01	9.69E+01
2631.918	9.60E+01	9.54E+01	9.70E+01
2632.4	9.60E+01	9.54E+01	9.70E+01
2632.883	9.60E+01	9.54E+01	9.70E+01
2633.365	9.61E+01	9.54E+01	9.70E+01
2633.847	9.61E+01	9.55E+01	9.71E+01
2634.329	9.61E+01	9.55E+01	9.71E+01
2634.811	9.61E+01	9.55E+01	9.71E+01
2635.293	9.60E+01	9.55E+01	9.71E+01
2635.775	9.60E+01	9.55E+01	9.71E+01
2636.257	9.60E+01	9.55E+01	9.71E+01
2636.74	9.60E+01	9.55E+01	9.71E+01
2637.222	9.61E+01	9.56E+01	9.71E+01
2637.704	9.61E+01	9.56E+01	9.71E+01
2638.186	9.61E+01	9.56E+01	9.71E+01
2638.668	9.61E+01	9.56E+01	9.71E+01
2639.15	9.60E+01	9.55E+01	9.71E+01
2639.632	9.60E+01	9.55E+01	9.71E+01
2640.114	9.60E+01	9.55E+01	9.71E+01
2640.596	9.60E+01	9.55E+01	9.71E+01
2641.079	9.60E+01	9.55E+01	9.71E+01
2641.561	9.60E+01	9.55E+01	9.71E+01
2642.043	9.60E+01	9.55E+01	9.71E+01

2642.525	9.61E+01	9.55E+01	9.71E+01
2643.007	9.61E+01	9.55E+01	9.71E+01
2643.489	9.60E+01	9.55E+01	9.71E+01
2643.971	9.60E+01	9.55E+01	9.71E+01
2644.453	9.60E+01	9.55E+01	9.71E+01
2644.936	9.60E+01	9.55E+01	9.71E+01
2645.418	9.60E+01	9.55E+01	9.71E+01
2645.9	9.60E+01	9.55E+01	9.72E+01
2646.382	9.60E+01	9.55E+01	9.72E+01
2646.864	9.60E+01	9.55E+01	9.72E+01
2647.346	9.60E+01	9.55E+01	9.72E+01
2647.828	9.60E+01	9.55E+01	9.72E+01
2648.311	9.60E+01	9.55E+01	9.71E+01
2648.792	9.60E+01	9.55E+01	9.71E+01
2649.275	9.60E+01	9.55E+01	9.71E+01
2649.757	9.60E+01	9.54E+01	9.71E+01
2650.239	9.60E+01	9.54E+01	9.70E+01
2650.721	9.60E+01	9.54E+01	9.70E+01
2651.203	9.60E+01	9.54E+01	9.70E+01
2651.685	9.60E+01	9.54E+01	9.70E+01
2652.167	9.59E+01	9.54E+01	9.70E+01
2652.65	9.59E+01	9.55E+01	9.70E+01
2653.132	9.59E+01	9.55E+01	9.70E+01
2653.614	9.59E+01	9.55E+01	9.70E+01
2654.096	9.59E+01	9.55E+01	9.69E+01
2654.578	9.59E+01	9.54E+01	9.69E+01
2655.06	9.59E+01	9.54E+01	9.69E+01
2655.542	9.59E+01	9.54E+01	9.69E+01
2656.024	9.59E+01	9.54E+01	9.69E+01
2656.507	9.59E+01	9.54E+01	9.69E+01
2656.989	9.59E+01	9.54E+01	9.70E+01
2657.471	9.59E+01	9.54E+01	9.70E+01
2657.953	9.59E+01	9.54E+01	9.70E+01
2658.435	9.59E+01	9.54E+01	9.70E+01
2658.917	9.59E+01	9.54E+01	9.70E+01
2659.399	9.59E+01	9.54E+01	9.70E+01
2659.881	9.59E+01	9.54E+01	9.70E+01
2660.364	9.59E+01	9.54E+01	9.70E+01
2660.846	9.59E+01	9.53E+01	9.70E+01
2661.328	9.59E+01	9.53E+01	9.70E+01
2661.81	9.59E+01	9.53E+01	9.70E+01
2662.292	9.59E+01	9.53E+01	9.70E+01
2662.774	9.59E+01	9.53E+01	9.70E+01
2663.256	9.59E+01	9.53E+01	9.70E+01
2663.739	9.59E+01	9.53E+01	9.70E+01
2664.22	9.59E+01	9.53E+01	9.70E+01
2664.703	9.59E+01	9.53E+01	9.70E+01

2665.185	9.59E+01	9.53E+01	9.70E+01
2665.667	9.59E+01	9.54E+01	9.70E+01
2666.149	9.59E+01	9.54E+01	9.70E+01
2666.631	9.59E+01	9.54E+01	9.70E+01
2667.113	9.59E+01	9.54E+01	9.70E+01
2667.595	9.59E+01	9.54E+01	9.70E+01
2668.078	9.59E+01	9.54E+01	9.70E+01
2668.56	9.59E+01	9.54E+01	9.70E+01
2669.042	9.59E+01	9.54E+01	9.70E+01
2669.524	9.59E+01	9.54E+01	9.70E+01
2670.006	9.60E+01	9.54E+01	9.70E+01
2670.488	9.60E+01	9.54E+01	9.70E+01
2670.97	9.60E+01	9.55E+01	9.70E+01
2671.452	9.60E+01	9.55E+01	9.70E+01
2671.935	9.60E+01	9.54E+01	9.70E+01
2672.417	9.60E+01	9.54E+01	9.70E+01
2672.899	9.60E+01	9.54E+01	9.70E+01
2673.381	9.60E+01	9.54E+01	9.70E+01
2673.863	9.60E+01	9.54E+01	9.70E+01
2674.345	9.59E+01	9.54E+01	9.70E+01
2674.827	9.59E+01	9.53E+01	9.70E+01
2675.309	9.59E+01	9.53E+01	9.70E+01
2675.792	9.59E+01	9.53E+01	9.70E+01
2676.274	9.59E+01	9.53E+01	9.70E+01
2676.756	9.59E+01	9.53E+01	9.70E+01
2677.238	9.59E+01	9.53E+01	9.69E+01
2677.72	9.59E+01	9.53E+01	9.69E+01
2678.202	9.59E+01	9.53E+01	9.69E+01
2678.684	9.59E+01	9.53E+01	9.69E+01
2679.167	9.59E+01	9.53E+01	9.69E+01
2679.648	9.59E+01	9.53E+01	9.69E+01
2680.131	9.59E+01	9.53E+01	9.69E+01
2680.613	9.59E+01	9.53E+01	9.69E+01
2681.095	9.59E+01	9.53E+01	9.69E+01
2681.577	9.59E+01	9.53E+01	9.70E+01
2682.059	9.59E+01	9.53E+01	9.70E+01
2682.541	9.59E+01	9.53E+01	9.70E+01
2683.023	9.59E+01	9.54E+01	9.70E+01
2683.506	9.59E+01	9.54E+01	9.70E+01
2683.988	9.59E+01	9.54E+01	9.70E+01
2684.47	9.59E+01	9.54E+01	9.70E+01
2684.952	9.59E+01	9.54E+01	9.70E+01
2685.434	9.59E+01	9.54E+01	9.69E+01
2685.916	9.59E+01	9.54E+01	9.69E+01
2686.398	9.59E+01	9.54E+01	9.69E+01
2686.88	9.59E+01	9.54E+01	9.69E+01
2687.363	9.59E+01	9.54E+01	9.69E+01

2687.845	9.59E+01	9.54E+01	9.69E+01
2688.327	9.59E+01	9.54E+01	9.69E+01
2688.809	9.59E+01	9.54E+01	9.69E+01
2689.291	9.59E+01	9.53E+01	9.69E+01
2689.773	9.59E+01	9.53E+01	9.69E+01
2690.255	9.59E+01	9.53E+01	9.69E+01
2690.737	9.59E+01	9.53E+01	9.69E+01
2691.219	9.59E+01	9.53E+01	9.69E+01
2691.702	9.59E+01	9.53E+01	9.69E+01
2692.184	9.59E+01	9.53E+01	9.69E+01
2692.666	9.59E+01	9.53E+01	9.69E+01
2693.148	9.58E+01	9.53E+01	9.69E+01
2693.63	9.58E+01	9.53E+01	9.69E+01
2694.112	9.58E+01	9.53E+01	9.69E+01
2694.594	9.58E+01	9.53E+01	9.69E+01
2695.077	9.58E+01	9.53E+01	9.69E+01
2695.559	9.58E+01	9.53E+01	9.69E+01
2696.041	9.59E+01	9.53E+01	9.69E+01
2696.523	9.59E+01	9.53E+01	9.69E+01
2697.005	9.59E+01	9.53E+01	9.69E+01
2697.487	9.58E+01	9.53E+01	9.69E+01
2697.969	9.58E+01	9.53E+01	9.69E+01
2698.451	9.58E+01	9.53E+01	9.68E+01
2698.934	9.58E+01	9.53E+01	9.68E+01
2699.416	9.58E+01	9.53E+01	9.68E+01
2699.898	9.58E+01	9.53E+01	9.68E+01
2700.38	9.58E+01	9.53E+01	9.68E+01
2700.862	9.59E+01	9.53E+01	9.69E+01
2701.344	9.59E+01	9.53E+01	9.69E+01
2701.826	9.59E+01	9.53E+01	9.69E+01
2702.308	9.59E+01	9.53E+01	9.69E+01
2702.791	9.59E+01	9.53E+01	9.69E+01
2703.273	9.59E+01	9.53E+01	9.69E+01
2703.755	9.59E+01	9.53E+01	9.69E+01
2704.237	9.59E+01	9.52E+01	9.69E+01
2704.719	9.59E+01	9.52E+01	9.69E+01
2705.201	9.59E+01	9.52E+01	9.69E+01
2705.683	9.59E+01	9.52E+01	9.69E+01
2706.166	9.58E+01	9.52E+01	9.69E+01
2706.647	9.58E+01	9.52E+01	9.68E+01
2707.13	9.58E+01	9.53E+01	9.68E+01
2707.612	9.58E+01	9.53E+01	9.68E+01
2708.094	9.58E+01	9.52E+01	9.68E+01
2708.576	9.58E+01	9.52E+01	9.69E+01
2709.058	9.58E+01	9.52E+01	9.69E+01
2709.54	9.58E+01	9.52E+01	9.69E+01
2710.022	9.58E+01	9.52E+01	9.69E+01

2710.505	9.58E+01	9.52E+01	9.69E+01
2710.987	9.58E+01	9.52E+01	9.69E+01
2711.469	9.58E+01	9.52E+01	9.69E+01
2711.951	9.58E+01	9.52E+01	9.69E+01
2712.433	9.58E+01	9.52E+01	9.69E+01
2712.915	9.58E+01	9.52E+01	9.69E+01
2713.397	9.58E+01	9.52E+01	9.69E+01
2713.879	9.58E+01	9.52E+01	9.69E+01
2714.362	9.59E+01	9.53E+01	9.69E+01
2714.844	9.59E+01	9.53E+01	9.69E+01
2715.326	9.59E+01	9.53E+01	9.69E+01
2715.808	9.59E+01	9.53E+01	9.69E+01
2716.29	9.59E+01	9.53E+01	9.69E+01
2716.772	9.59E+01	9.53E+01	9.69E+01
2717.254	9.59E+01	9.53E+01	9.69E+01
2717.736	9.59E+01	9.53E+01	9.69E+01
2718.219	9.59E+01	9.53E+01	9.69E+01
2718.701	9.59E+01	9.52E+01	9.69E+01
2719.183	9.59E+01	9.52E+01	9.69E+01
2719.665	9.59E+01	9.52E+01	9.69E+01
2720.147	9.59E+01	9.52E+01	9.69E+01
2720.629	9.59E+01	9.52E+01	9.69E+01
2721.111	9.59E+01	9.52E+01	9.69E+01
2721.594	9.59E+01	9.52E+01	9.69E+01
2722.075	9.58E+01	9.52E+01	9.69E+01
2722.558	9.58E+01	9.52E+01	9.69E+01
2723.04	9.58E+01	9.52E+01	9.69E+01
2723.522	9.58E+01	9.52E+01	9.69E+01
2724.004	9.58E+01	9.52E+01	9.69E+01
2724.486	9.58E+01	9.52E+01	9.69E+01
2724.968	9.58E+01	9.52E+01	9.69E+01
2725.45	9.58E+01	9.53E+01	9.69E+01
2725.933	9.58E+01	9.53E+01	9.69E+01
2726.415	9.58E+01	9.53E+01	9.69E+01
2726.897	9.58E+01	9.53E+01	9.69E+01
2727.379	9.58E+01	9.53E+01	9.69E+01
2727.861	9.58E+01	9.53E+01	9.69E+01
2728.343	9.58E+01	9.52E+01	9.69E+01
2728.825	9.58E+01	9.52E+01	9.69E+01
2729.307	9.58E+01	9.52E+01	9.69E+01
2729.79	9.58E+01	9.52E+01	9.69E+01
2730.272	9.58E+01	9.52E+01	9.69E+01
2730.754	9.58E+01	9.52E+01	9.69E+01
2731.236	9.58E+01	9.52E+01	9.69E+01
2731.718	9.58E+01	9.52E+01	9.69E+01
2732.2	9.58E+01	9.52E+01	9.69E+01
2732.682	9.58E+01	9.52E+01	9.69E+01

2733.164	9.58E+01	9.52E+01	9.69E+01
2733.646	9.57E+01	9.52E+01	9.68E+01
2734.129	9.57E+01	9.52E+01	9.68E+01
2734.611	9.57E+01	9.52E+01	9.68E+01
2735.093	9.57E+01	9.51E+01	9.68E+01
2735.575	9.57E+01	9.51E+01	9.68E+01
2736.057	9.57E+01	9.51E+01	9.67E+01
2736.539	9.57E+01	9.51E+01	9.67E+01
2737.021	9.58E+01	9.51E+01	9.67E+01
2737.503	9.58E+01	9.51E+01	9.67E+01
2737.986	9.58E+01	9.51E+01	9.68E+01
2738.468	9.58E+01	9.51E+01	9.68E+01
2738.95	9.58E+01	9.51E+01	9.68E+01
2739.432	9.58E+01	9.51E+01	9.68E+01
2739.914	9.58E+01	9.51E+01	9.68E+01
2740.396	9.58E+01	9.51E+01	9.69E+01
2740.878	9.58E+01	9.52E+01	9.69E+01
2741.361	9.58E+01	9.52E+01	9.69E+01
2741.843	9.58E+01	9.52E+01	9.69E+01
2742.325	9.58E+01	9.52E+01	9.69E+01
2742.807	9.58E+01	9.52E+01	9.69E+01
2743.289	9.58E+01	9.52E+01	9.69E+01
2743.771	9.58E+01	9.52E+01	9.69E+01
2744.253	9.58E+01	9.52E+01	9.69E+01
2744.735	9.59E+01	9.52E+01	9.69E+01
2745.218	9.59E+01	9.52E+01	9.69E+01
2745.7	9.59E+01	9.52E+01	9.69E+01
2746.182	9.59E+01	9.52E+01	9.69E+01
2746.664	9.59E+01	9.52E+01	9.69E+01
2747.146	9.59E+01	9.52E+01	9.69E+01
2747.628	9.59E+01	9.52E+01	9.69E+01
2748.11	9.59E+01	9.52E+01	9.69E+01
2748.592	9.59E+01	9.52E+01	9.69E+01
2749.074	9.58E+01	9.52E+01	9.69E+01
2749.557	9.58E+01	9.52E+01	9.69E+01
2750.039	9.58E+01	9.52E+01	9.69E+01
2750.521	9.58E+01	9.52E+01	9.68E+01
2751.003	9.58E+01	9.52E+01	9.68E+01
2751.485	9.58E+01	9.51E+01	9.68E+01
2751.967	9.58E+01	9.51E+01	9.68E+01
2752.449	9.58E+01	9.51E+01	9.68E+01
2752.931	9.59E+01	9.51E+01	9.68E+01
2753.414	9.59E+01	9.51E+01	9.68E+01
2753.896	9.59E+01	9.52E+01	9.68E+01
2754.378	9.59E+01	9.52E+01	9.68E+01
2754.86	9.59E+01	9.52E+01	9.68E+01
2755.342	9.59E+01	9.51E+01	9.68E+01

2755.824	9.59E+01	9.51E+01	9.68E+01
2756.306	9.59E+01	9.51E+01	9.68E+01
2756.789	9.58E+01	9.51E+01	9.68E+01
2757.271	9.58E+01	9.51E+01	9.68E+01
2757.753	9.58E+01	9.51E+01	9.69E+01
2758.235	9.58E+01	9.51E+01	9.69E+01
2758.717	9.58E+01	9.52E+01	9.69E+01
2759.199	9.58E+01	9.52E+01	9.69E+01
2759.681	9.58E+01	9.52E+01	9.69E+01
2760.163	9.59E+01	9.52E+01	9.69E+01
2760.646	9.59E+01	9.52E+01	9.69E+01
2761.128	9.59E+01	9.52E+01	9.69E+01
2761.61	9.59E+01	9.52E+01	9.69E+01
2762.092	9.58E+01	9.51E+01	9.69E+01
2762.574	9.58E+01	9.51E+01	9.69E+01
2763.056	9.58E+01	9.51E+01	9.69E+01
2763.538	9.58E+01	9.51E+01	9.69E+01
2764.02	9.58E+01	9.51E+01	9.69E+01
2764.502	9.58E+01	9.51E+01	9.69E+01
2764.985	9.58E+01	9.51E+01	9.68E+01
2765.467	9.58E+01	9.51E+01	9.68E+01
2765.949	9.58E+01	9.51E+01	9.68E+01
2766.431	9.58E+01	9.51E+01	9.68E+01
2766.913	9.58E+01	9.50E+01	9.68E+01
2767.395	9.58E+01	9.50E+01	9.68E+01
2767.877	9.57E+01	9.50E+01	9.68E+01
2768.359	9.57E+01	9.50E+01	9.68E+01
2768.842	9.58E+01	9.51E+01	9.68E+01
2769.324	9.58E+01	9.51E+01	9.68E+01
2769.806	9.58E+01	9.51E+01	9.68E+01
2770.288	9.58E+01	9.51E+01	9.68E+01
2770.77	9.58E+01	9.51E+01	9.68E+01
2771.252	9.58E+01	9.51E+01	9.68E+01
2771.734	9.58E+01	9.51E+01	9.68E+01
2772.217	9.58E+01	9.51E+01	9.68E+01
2772.698	9.58E+01	9.51E+01	9.68E+01
2773.181	9.59E+01	9.51E+01	9.68E+01
2773.663	9.59E+01	9.51E+01	9.68E+01
2774.145	9.59E+01	9.51E+01	9.68E+01
2774.627	9.59E+01	9.51E+01	9.68E+01
2775.109	9.59E+01	9.51E+01	9.68E+01
2775.591	9.59E+01	9.51E+01	9.68E+01
2776.073	9.58E+01	9.51E+01	9.68E+01
2776.556	9.58E+01	9.51E+01	9.68E+01
2777.038	9.58E+01	9.51E+01	9.68E+01
2777.52	9.58E+01	9.51E+01	9.68E+01
2778.002	9.58E+01	9.51E+01	9.68E+01

2778.484	9.58E+01	9.51E+01	9.68E+01
2778.966	9.58E+01	9.51E+01	9.68E+01
2779.448	9.58E+01	9.50E+01	9.68E+01
2779.93	9.58E+01	9.50E+01	9.68E+01
2780.413	9.58E+01	9.50E+01	9.68E+01
2780.895	9.57E+01	9.50E+01	9.68E+01
2781.377	9.57E+01	9.50E+01	9.67E+01
2781.859	9.57E+01	9.50E+01	9.67E+01
2782.341	9.56E+01	9.49E+01	9.67E+01
2782.823	9.56E+01	9.49E+01	9.67E+01
2783.305	9.56E+01	9.50E+01	9.67E+01
2783.787	9.56E+01	9.50E+01	9.67E+01
2784.27	9.56E+01	9.50E+01	9.68E+01
2784.752	9.56E+01	9.50E+01	9.68E+01
2785.234	9.56E+01	9.50E+01	9.68E+01
2785.716	9.56E+01	9.50E+01	9.68E+01
2786.198	9.56E+01	9.50E+01	9.68E+01
2786.68	9.56E+01	9.49E+01	9.68E+01
2787.162	9.57E+01	9.49E+01	9.68E+01
2787.645	9.57E+01	9.49E+01	9.67E+01
2788.127	9.57E+01	9.49E+01	9.67E+01
2788.609	9.57E+01	9.49E+01	9.67E+01
2789.091	9.57E+01	9.50E+01	9.67E+01
2789.573	9.58E+01	9.50E+01	9.67E+01
2790.055	9.58E+01	9.50E+01	9.67E+01
2790.537	9.58E+01	9.50E+01	9.67E+01
2791.019	9.58E+01	9.50E+01	9.67E+01
2791.501	9.58E+01	9.50E+01	9.67E+01
2791.984	9.57E+01	9.50E+01	9.68E+01
2792.466	9.57E+01	9.50E+01	9.68E+01
2792.948	9.57E+01	9.50E+01	9.68E+01
2793.43	9.57E+01	9.50E+01	9.68E+01
2793.912	9.57E+01	9.50E+01	9.68E+01
2794.394	9.57E+01	9.49E+01	9.68E+01
2794.876	9.57E+01	9.49E+01	9.68E+01
2795.358	9.57E+01	9.49E+01	9.68E+01
2795.841	9.57E+01	9.50E+01	9.68E+01
2796.323	9.57E+01	9.50E+01	9.68E+01
2796.805	9.57E+01	9.50E+01	9.68E+01
2797.287	9.57E+01	9.50E+01	9.68E+01
2797.769	9.57E+01	9.49E+01	9.68E+01
2798.251	9.56E+01	9.49E+01	9.68E+01
2798.733	9.56E+01	9.49E+01	9.67E+01
2799.215	9.56E+01	9.49E+01	9.67E+01
2799.698	9.56E+01	9.49E+01	9.67E+01
2800.18	9.56E+01	9.49E+01	9.67E+01
2800.662	9.57E+01	9.49E+01	9.67E+01

2801.144	9.57E+01	9.50E+01	9.68E+01
2801.626	9.57E+01	9.50E+01	9.68E+01
2802.108	9.57E+01	9.50E+01	9.68E+01
2802.59	9.57E+01	9.50E+01	9.68E+01
2803.073	9.57E+01	9.50E+01	9.68E+01
2803.555	9.57E+01	9.50E+01	9.68E+01
2804.037	9.57E+01	9.50E+01	9.68E+01
2804.519	9.57E+01	9.50E+01	9.68E+01
2805.001	9.57E+01	9.50E+01	9.68E+01
2805.483	9.57E+01	9.50E+01	9.67E+01
2805.965	9.56E+01	9.49E+01	9.67E+01
2806.447	9.56E+01	9.49E+01	9.67E+01
2806.929	9.56E+01	9.49E+01	9.67E+01
2807.412	9.56E+01	9.49E+01	9.67E+01
2807.894	9.56E+01	9.49E+01	9.67E+01
2808.376	9.56E+01	9.49E+01	9.67E+01
2808.858	9.57E+01	9.49E+01	9.67E+01
2809.34	9.57E+01	9.49E+01	9.68E+01
2809.822	9.57E+01	9.50E+01	9.68E+01
2810.304	9.58E+01	9.50E+01	9.68E+01
2810.786	9.58E+01	9.50E+01	9.68E+01
2811.269	9.58E+01	9.49E+01	9.67E+01
2811.751	9.57E+01	9.49E+01	9.67E+01
2812.233	9.57E+01	9.49E+01	9.67E+01
2812.715	9.57E+01	9.49E+01	9.66E+01
2813.197	9.56E+01	9.48E+01	9.66E+01
2813.679	9.56E+01	9.48E+01	9.65E+01
2814.161	9.55E+01	9.48E+01	9.65E+01
2814.644	9.55E+01	9.47E+01	9.65E+01
2815.125	9.55E+01	9.47E+01	9.65E+01
2815.608	9.55E+01	9.47E+01	9.65E+01
2816.09	9.55E+01	9.47E+01	9.66E+01
2816.572	9.55E+01	9.47E+01	9.66E+01
2817.054	9.55E+01	9.48E+01	9.66E+01
2817.536	9.55E+01	9.48E+01	9.66E+01
2818.018	9.55E+01	9.48E+01	9.66E+01
2818.5	9.55E+01	9.48E+01	9.66E+01
2818.983	9.56E+01	9.49E+01	9.66E+01
2819.465	9.56E+01	9.49E+01	9.66E+01
2819.947	9.56E+01	9.49E+01	9.66E+01
2820.429	9.56E+01	9.49E+01	9.66E+01
2820.911	9.56E+01	9.49E+01	9.66E+01
2821.393	9.56E+01	9.49E+01	9.66E+01
2821.875	9.56E+01	9.48E+01	9.66E+01
2822.357	9.55E+01	9.48E+01	9.66E+01
2822.84	9.55E+01	9.48E+01	9.66E+01
2823.322	9.55E+01	9.48E+01	9.66E+01

2823.804	9.55E+01	9.48E+01	9.66E+01
2824.286	9.55E+01	9.48E+01	9.66E+01
2824.768	9.55E+01	9.48E+01	9.66E+01
2825.25	9.55E+01	9.48E+01	9.67E+01
2825.732	9.55E+01	9.48E+01	9.67E+01
2826.214	9.55E+01	9.48E+01	9.67E+01
2826.697	9.54E+01	9.47E+01	9.67E+01
2827.179	9.54E+01	9.47E+01	9.67E+01
2827.661	9.54E+01	9.47E+01	9.67E+01
2828.143	9.54E+01	9.47E+01	9.67E+01
2828.625	9.54E+01	9.47E+01	9.67E+01
2829.107	9.54E+01	9.47E+01	9.67E+01
2829.589	9.54E+01	9.47E+01	9.67E+01
2830.072	9.54E+01	9.47E+01	9.67E+01
2830.553	9.55E+01	9.47E+01	9.67E+01
2831.036	9.55E+01	9.47E+01	9.67E+01
2831.518	9.55E+01	9.47E+01	9.67E+01
2832	9.55E+01	9.47E+01	9.67E+01
2832.482	9.55E+01	9.47E+01	9.68E+01
2832.964	9.55E+01	9.47E+01	9.68E+01
2833.446	9.56E+01	9.47E+01	9.68E+01
2833.928	9.56E+01	9.47E+01	9.68E+01
2834.411	9.56E+01	9.47E+01	9.67E+01
2834.893	9.56E+01	9.47E+01	9.67E+01
2835.375	9.56E+01	9.47E+01	9.67E+01
2835.857	9.56E+01	9.47E+01	9.67E+01
2836.339	9.56E+01	9.47E+01	9.67E+01
2836.821	9.56E+01	9.47E+01	9.67E+01
2837.303	9.56E+01	9.47E+01	9.67E+01
2837.785	9.55E+01	9.47E+01	9.67E+01
2838.268	9.55E+01	9.47E+01	9.66E+01
2838.75	9.55E+01	9.46E+01	9.66E+01
2839.232	9.55E+01	9.46E+01	9.66E+01
2839.714	9.54E+01	9.46E+01	9.66E+01
2840.196	9.54E+01	9.46E+01	9.65E+01
2840.678	9.54E+01	9.46E+01	9.65E+01
2841.16	9.54E+01	9.46E+01	9.65E+01
2841.642	9.54E+01	9.46E+01	9.65E+01
2842.125	9.54E+01	9.46E+01	9.65E+01
2842.607	9.53E+01	9.46E+01	9.65E+01
2843.089	9.53E+01	9.46E+01	9.66E+01
2843.571	9.53E+01	9.45E+01	9.66E+01
2844.053	9.53E+01	9.45E+01	9.66E+01
2844.535	9.53E+01	9.45E+01	9.65E+01
2845.017	9.52E+01	9.45E+01	9.65E+01
2845.5	9.52E+01	9.44E+01	9.65E+01
2845.981	9.52E+01	9.44E+01	9.65E+01

2846.464	9.51E+01	9.44E+01	9.65E+01
2846.946	9.51E+01	9.43E+01	9.65E+01
2847.428	9.51E+01	9.43E+01	9.65E+01
2847.91	9.51E+01	9.43E+01	9.65E+01
2848.392	9.50E+01	9.43E+01	9.65E+01
2848.874	9.50E+01	9.43E+01	9.66E+01
2849.356	9.50E+01	9.43E+01	9.66E+01
2849.839	9.51E+01	9.44E+01	9.66E+01
2850.321	9.51E+01	9.44E+01	9.66E+01
2850.803	9.51E+01	9.45E+01	9.66E+01
2851.285	9.51E+01	9.45E+01	9.66E+01
2851.767	9.51E+01	9.46E+01	9.66E+01
2852.249	9.51E+01	9.46E+01	9.66E+01
2852.731	9.51E+01	9.46E+01	9.66E+01
2853.213	9.51E+01	9.46E+01	9.66E+01
2853.696	9.51E+01	9.45E+01	9.65E+01
2854.178	9.51E+01	9.45E+01	9.65E+01
2854.66	9.51E+01	9.44E+01	9.65E+01
2855.142	9.51E+01	9.44E+01	9.65E+01
2855.624	9.51E+01	9.44E+01	9.64E+01
2856.106	9.51E+01	9.43E+01	9.64E+01
2856.588	9.51E+01	9.43E+01	9.64E+01
2857.07	9.51E+01	9.43E+01	9.65E+01
2857.552	9.51E+01	9.43E+01	9.65E+01
2858.035	9.51E+01	9.43E+01	9.65E+01
2858.517	9.52E+01	9.43E+01	9.65E+01
2858.999	9.52E+01	9.43E+01	9.65E+01
2859.481	9.52E+01	9.43E+01	9.65E+01
2859.963	9.52E+01	9.43E+01	9.65E+01
2860.445	9.52E+01	9.43E+01	9.64E+01
2860.927	9.52E+01	9.43E+01	9.64E+01
2861.409	9.51E+01	9.42E+01	9.64E+01
2861.892	9.51E+01	9.42E+01	9.63E+01
2862.374	9.50E+01	9.42E+01	9.63E+01
2862.856	9.50E+01	9.42E+01	9.63E+01
2863.338	9.50E+01	9.42E+01	9.64E+01
2863.82	9.50E+01	9.42E+01	9.64E+01
2864.302	9.50E+01	9.42E+01	9.64E+01
2864.784	9.50E+01	9.42E+01	9.65E+01
2865.267	9.50E+01	9.42E+01	9.65E+01
2865.749	9.50E+01	9.42E+01	9.65E+01
2866.231	9.50E+01	9.42E+01	9.65E+01
2866.713	9.51E+01	9.42E+01	9.65E+01
2867.195	9.51E+01	9.42E+01	9.65E+01
2867.677	9.51E+01	9.42E+01	9.64E+01
2868.159	9.51E+01	9.42E+01	9.64E+01
2868.641	9.50E+01	9.42E+01	9.64E+01

2869.124	9.50E+01	9.42E+01	9.63E+01
2869.606	9.50E+01	9.42E+01	9.63E+01
2870.088	9.50E+01	9.42E+01	9.63E+01
2870.57	9.50E+01	9.42E+01	9.63E+01
2871.052	9.50E+01	9.42E+01	9.63E+01
2871.534	9.50E+01	9.42E+01	9.63E+01
2872.016	9.50E+01	9.42E+01	9.64E+01
2872.498	9.50E+01	9.42E+01	9.64E+01
2872.98	9.50E+01	9.42E+01	9.64E+01
2873.463	9.50E+01	9.42E+01	9.64E+01
2873.945	9.50E+01	9.43E+01	9.63E+01
2874.427	9.51E+01	9.43E+01	9.63E+01
2874.909	9.51E+01	9.43E+01	9.63E+01
2875.391	9.51E+01	9.43E+01	9.63E+01
2875.873	9.51E+01	9.43E+01	9.63E+01
2876.355	9.52E+01	9.43E+01	9.63E+01
2876.837	9.52E+01	9.43E+01	9.63E+01
2877.32	9.52E+01	9.43E+01	9.63E+01
2877.802	9.52E+01	9.43E+01	9.64E+01
2878.284	9.52E+01	9.43E+01	9.64E+01
2878.766	9.52E+01	9.42E+01	9.64E+01
2879.248	9.52E+01	9.42E+01	9.64E+01
2879.73	9.52E+01	9.42E+01	9.64E+01
2880.212	9.52E+01	9.42E+01	9.64E+01
2880.695	9.52E+01	9.42E+01	9.64E+01
2881.177	9.52E+01	9.42E+01	9.64E+01
2881.659	9.52E+01	9.42E+01	9.64E+01
2882.141	9.51E+01	9.42E+01	9.64E+01
2882.623	9.51E+01	9.42E+01	9.64E+01
2883.105	9.51E+01	9.42E+01	9.64E+01
2883.587	9.51E+01	9.42E+01	9.64E+01
2884.069	9.51E+01	9.42E+01	9.64E+01
2884.552	9.51E+01	9.42E+01	9.64E+01
2885.034	9.51E+01	9.42E+01	9.64E+01
2885.516	9.51E+01	9.42E+01	9.64E+01
2885.998	9.51E+01	9.42E+01	9.64E+01
2886.48	9.51E+01	9.42E+01	9.64E+01
2886.962	9.51E+01	9.42E+01	9.63E+01
2887.444	9.51E+01	9.41E+01	9.63E+01
2887.926	9.51E+01	9.41E+01	9.63E+01
2888.408	9.51E+01	9.41E+01	9.63E+01
2888.891	9.50E+01	9.40E+01	9.63E+01
2889.373	9.50E+01	9.40E+01	9.63E+01
2889.855	9.51E+01	9.40E+01	9.63E+01
2890.337	9.51E+01	9.40E+01	9.63E+01
2890.819	9.51E+01	9.40E+01	9.64E+01
2891.301	9.51E+01	9.41E+01	9.64E+01

2891.783	9.50E+01	9.41E+01	9.64E+01
2892.265	9.50E+01	9.41E+01	9.64E+01
2892.748	9.50E+01	9.41E+01	9.64E+01
2893.23	9.50E+01	9.41E+01	9.63E+01
2893.712	9.50E+01	9.41E+01	9.63E+01
2894.194	9.50E+01	9.41E+01	9.63E+01
2894.676	9.50E+01	9.41E+01	9.63E+01
2895.158	9.49E+01	9.41E+01	9.63E+01
2895.64	9.49E+01	9.40E+01	9.63E+01
2896.123	9.49E+01	9.40E+01	9.63E+01
2896.605	9.49E+01	9.40E+01	9.63E+01
2897.087	9.49E+01	9.40E+01	9.63E+01
2897.569	9.49E+01	9.40E+01	9.63E+01
2898.051	9.49E+01	9.40E+01	9.63E+01
2898.533	9.49E+01	9.41E+01	9.63E+01
2899.015	9.49E+01	9.41E+01	9.63E+01
2899.497	9.49E+01	9.41E+01	9.63E+01
2899.979	9.50E+01	9.41E+01	9.63E+01
2900.462	9.50E+01	9.41E+01	9.63E+01
2900.944	9.50E+01	9.41E+01	9.63E+01
2901.426	9.50E+01	9.41E+01	9.63E+01
2901.908	9.50E+01	9.40E+01	9.63E+01
2902.39	9.49E+01	9.40E+01	9.63E+01
2902.872	9.49E+01	9.40E+01	9.63E+01
2903.354	9.49E+01	9.40E+01	9.63E+01
2903.836	9.49E+01	9.40E+01	9.63E+01
2904.319	9.49E+01	9.39E+01	9.63E+01
2904.801	9.48E+01	9.39E+01	9.63E+01
2905.283	9.48E+01	9.39E+01	9.62E+01
2905.765	9.48E+01	9.38E+01	9.62E+01
2906.247	9.48E+01	9.38E+01	9.62E+01
2906.729	9.47E+01	9.38E+01	9.62E+01
2907.211	9.47E+01	9.38E+01	9.62E+01
2907.694	9.47E+01	9.38E+01	9.61E+01
2908.176	9.46E+01	9.37E+01	9.61E+01
2908.658	9.46E+01	9.37E+01	9.61E+01
2909.14	9.46E+01	9.37E+01	9.61E+01
2909.622	9.45E+01	9.37E+01	9.61E+01
2910.104	9.45E+01	9.37E+01	9.61E+01
2910.586	9.45E+01	9.36E+01	9.61E+01
2911.068	9.45E+01	9.36E+01	9.61E+01
2911.551	9.45E+01	9.35E+01	9.61E+01
2912.033	9.45E+01	9.35E+01	9.61E+01
2912.515	9.44E+01	9.35E+01	9.61E+01
2912.997	9.44E+01	9.34E+01	9.61E+01
2913.479	9.43E+01	9.34E+01	9.60E+01
2913.961	9.43E+01	9.33E+01	9.60E+01

2914.443	9.42E+01	9.33E+01	9.60E+01
2914.925	9.42E+01	9.33E+01	9.59E+01
2915.407	9.41E+01	9.32E+01	9.59E+01
2915.89	9.41E+01	9.32E+01	9.59E+01
2916.372	9.40E+01	9.32E+01	9.59E+01
2916.854	9.40E+01	9.32E+01	9.59E+01
2917.336	9.40E+01	9.32E+01	9.59E+01
2917.818	9.40E+01	9.32E+01	9.59E+01
2918.3	9.40E+01	9.32E+01	9.60E+01
2918.782	9.40E+01	9.32E+01	9.60E+01
2919.264	9.40E+01	9.32E+01	9.60E+01
2919.747	9.40E+01	9.32E+01	9.60E+01
2920.229	9.40E+01	9.32E+01	9.60E+01
2920.711	9.39E+01	9.32E+01	9.59E+01
2921.193	9.39E+01	9.31E+01	9.59E+01
2921.675	9.39E+01	9.31E+01	9.59E+01
2922.157	9.39E+01	9.31E+01	9.59E+01
2922.639	9.39E+01	9.31E+01	9.59E+01
2923.122	9.39E+01	9.31E+01	9.59E+01
2923.604	9.39E+01	9.31E+01	9.59E+01
2924.086	9.39E+01	9.31E+01	9.59E+01
2924.568	9.39E+01	9.31E+01	9.60E+01
2925.05	9.39E+01	9.31E+01	9.60E+01
2925.532	9.39E+01	9.31E+01	9.59E+01
2926.014	9.39E+01	9.31E+01	9.59E+01
2926.496	9.39E+01	9.31E+01	9.59E+01
2926.979	9.39E+01	9.31E+01	9.59E+01
2927.461	9.38E+01	9.31E+01	9.58E+01
2927.943	9.38E+01	9.31E+01	9.58E+01
2928.425	9.38E+01	9.31E+01	9.58E+01
2928.907	9.38E+01	9.31E+01	9.58E+01
2929.389	9.39E+01	9.31E+01	9.58E+01
2929.871	9.39E+01	9.31E+01	9.58E+01
2930.353	9.39E+01	9.31E+01	9.58E+01
2930.835	9.39E+01	9.31E+01	9.58E+01
2931.318	9.39E+01	9.30E+01	9.57E+01
2931.8	9.38E+01	9.31E+01	9.57E+01
2932.282	9.38E+01	9.31E+01	9.57E+01
2932.764	9.38E+01	9.31E+01	9.57E+01
2933.246	9.39E+01	9.31E+01	9.57E+01
2933.728	9.39E+01	9.31E+01	9.57E+01
2934.21	9.39E+01	9.32E+01	9.57E+01
2934.692	9.40E+01	9.32E+01	9.58E+01
2935.175	9.41E+01	9.32E+01	9.58E+01
2935.657	9.41E+01	9.32E+01	9.58E+01
2936.139	9.41E+01	9.32E+01	9.58E+01
2936.621	9.42E+01	9.32E+01	9.58E+01

2937.103	9.42E+01	9.32E+01	9.58E+01
2937.585	9.42E+01	9.32E+01	9.58E+01
2938.067	9.42E+01	9.32E+01	9.58E+01
2938.55	9.42E+01	9.32E+01	9.58E+01
2939.031	9.42E+01	9.32E+01	9.59E+01
2939.514	9.42E+01	9.32E+01	9.59E+01
2939.996	9.42E+01	9.32E+01	9.59E+01
2940.478	9.41E+01	9.32E+01	9.59E+01
2940.96	9.41E+01	9.33E+01	9.59E+01
2941.442	9.41E+01	9.33E+01	9.59E+01
2941.924	9.41E+01	9.33E+01	9.59E+01
2942.406	9.42E+01	9.33E+01	9.59E+01
2942.889	9.42E+01	9.33E+01	9.60E+01
2943.371	9.42E+01	9.34E+01	9.60E+01
2943.853	9.42E+01	9.34E+01	9.60E+01
2944.335	9.43E+01	9.34E+01	9.60E+01
2944.817	9.43E+01	9.34E+01	9.59E+01
2945.299	9.43E+01	9.34E+01	9.59E+01
2945.781	9.44E+01	9.34E+01	9.59E+01
2946.263	9.44E+01	9.34E+01	9.59E+01
2946.746	9.44E+01	9.34E+01	9.60E+01
2947.228	9.44E+01	9.34E+01	9.60E+01
2947.71	9.45E+01	9.34E+01	9.60E+01
2948.192	9.45E+01	9.34E+01	9.60E+01
2948.674	9.45E+01	9.34E+01	9.60E+01
2949.156	9.45E+01	9.34E+01	9.60E+01
2949.638	9.45E+01	9.35E+01	9.60E+01
2950.12	9.45E+01	9.35E+01	9.60E+01
2950.603	9.45E+01	9.35E+01	9.61E+01
2951.085	9.45E+01	9.36E+01	9.61E+01
2951.567	9.45E+01	9.36E+01	9.61E+01
2952.049	9.45E+01	9.36E+01	9.61E+01
2952.531	9.45E+01	9.37E+01	9.61E+01
2953.013	9.45E+01	9.37E+01	9.61E+01
2953.495	9.45E+01	9.37E+01	9.61E+01
2953.978	9.45E+01	9.37E+01	9.61E+01
2954.459	9.45E+01	9.37E+01	9.61E+01
2954.942	9.45E+01	9.37E+01	9.61E+01
2955.424	9.46E+01	9.38E+01	9.62E+01
2955.906	9.46E+01	9.38E+01	9.62E+01
2956.388	9.47E+01	9.38E+01	9.62E+01
2956.87	9.47E+01	9.38E+01	9.62E+01
2957.352	9.47E+01	9.39E+01	9.62E+01
2957.834	9.48E+01	9.39E+01	9.62E+01
2958.317	9.48E+01	9.39E+01	9.62E+01
2958.799	9.48E+01	9.39E+01	9.63E+01
2959.281	9.48E+01	9.40E+01	9.62E+01

2959.763	9.48E+01	9.40E+01	9.62E+01
2960.245	9.48E+01	9.40E+01	9.62E+01
2960.727	9.48E+01	9.40E+01	9.62E+01
2961.209	9.48E+01	9.40E+01	9.63E+01
2961.691	9.48E+01	9.40E+01	9.63E+01
2962.174	9.48E+01	9.40E+01	9.63E+01
2962.656	9.48E+01	9.40E+01	9.63E+01
2963.138	9.48E+01	9.40E+01	9.63E+01
2963.62	9.48E+01	9.39E+01	9.63E+01
2964.102	9.48E+01	9.39E+01	9.63E+01
2964.584	9.48E+01	9.39E+01	9.63E+01
2965.066	9.48E+01	9.39E+01	9.63E+01
2965.548	9.48E+01	9.39E+01	9.63E+01
2966.031	9.48E+01	9.39E+01	9.62E+01
2966.513	9.48E+01	9.39E+01	9.62E+01
2966.995	9.48E+01	9.39E+01	9.62E+01
2967.477	9.48E+01	9.39E+01	9.62E+01
2967.959	9.48E+01	9.40E+01	9.62E+01
2968.441	9.49E+01	9.40E+01	9.62E+01
2968.923	9.49E+01	9.41E+01	9.63E+01
2969.406	9.49E+01	9.41E+01	9.63E+01
2969.887	9.50E+01	9.41E+01	9.63E+01
2970.37	9.50E+01	9.42E+01	9.64E+01
2970.852	9.50E+01	9.42E+01	9.64E+01
2971.334	9.50E+01	9.41E+01	9.64E+01
2971.816	9.50E+01	9.41E+01	9.64E+01
2972.298	9.50E+01	9.41E+01	9.63E+01
2972.78	9.50E+01	9.41E+01	9.63E+01
2973.262	9.50E+01	9.40E+01	9.63E+01
2973.745	9.50E+01	9.40E+01	9.62E+01
2974.227	9.50E+01	9.40E+01	9.62E+01
2974.709	9.50E+01	9.39E+01	9.62E+01
2975.191	9.50E+01	9.39E+01	9.62E+01
2975.673	9.50E+01	9.39E+01	9.62E+01
2976.155	9.50E+01	9.39E+01	9.62E+01
2976.637	9.51E+01	9.39E+01	9.62E+01
2977.119	9.51E+01	9.40E+01	9.62E+01
2977.602	9.51E+01	9.40E+01	9.62E+01
2978.084	9.52E+01	9.41E+01	9.62E+01
2978.566	9.52E+01	9.41E+01	9.63E+01
2979.048	9.52E+01	9.42E+01	9.63E+01
2979.53	9.52E+01	9.42E+01	9.63E+01
2980.012	9.52E+01	9.42E+01	9.63E+01
2980.494	9.52E+01	9.42E+01	9.63E+01
2980.976	9.52E+01	9.42E+01	9.63E+01
2981.458	9.52E+01	9.42E+01	9.63E+01
2981.941	9.52E+01	9.42E+01	9.63E+01

2982.423	9.51E+01	9.42E+01	9.63E+01
2982.905	9.51E+01	9.42E+01	9.63E+01
2983.387	9.51E+01	9.41E+01	9.63E+01
2983.869	9.51E+01	9.41E+01	9.63E+01
2984.351	9.51E+01	9.41E+01	9.63E+01
2984.833	9.51E+01	9.41E+01	9.62E+01
2985.315	9.51E+01	9.41E+01	9.62E+01
2985.798	9.51E+01	9.41E+01	9.62E+01
2986.28	9.51E+01	9.41E+01	9.63E+01
2986.762	9.51E+01	9.41E+01	9.63E+01
2987.244	9.51E+01	9.41E+01	9.64E+01
2987.726	9.51E+01	9.41E+01	9.64E+01
2988.208	9.51E+01	9.41E+01	9.64E+01
2988.69	9.51E+01	9.41E+01	9.65E+01
2989.173	9.51E+01	9.41E+01	9.65E+01
2989.655	9.51E+01	9.41E+01	9.65E+01
2990.137	9.51E+01	9.41E+01	9.64E+01
2990.619	9.51E+01	9.41E+01	9.64E+01
2991.101	9.50E+01	9.41E+01	9.64E+01
2991.583	9.50E+01	9.41E+01	9.63E+01
2992.065	9.50E+01	9.41E+01	9.63E+01
2992.547	9.50E+01	9.41E+01	9.63E+01
2993.03	9.51E+01	9.42E+01	9.63E+01
2993.512	9.51E+01	9.42E+01	9.63E+01
2993.994	9.51E+01	9.42E+01	9.63E+01
2994.476	9.51E+01	9.42E+01	9.63E+01
2994.958	9.52E+01	9.42E+01	9.63E+01
2995.44	9.52E+01	9.42E+01	9.63E+01
2995.922	9.52E+01	9.42E+01	9.63E+01
2996.404	9.52E+01	9.42E+01	9.63E+01
2996.886	9.52E+01	9.42E+01	9.64E+01
2997.369	9.52E+01	9.42E+01	9.64E+01
2997.851	9.53E+01	9.42E+01	9.64E+01
2998.333	9.53E+01	9.42E+01	9.64E+01
2998.815	9.53E+01	9.42E+01	9.64E+01
2999.297	9.53E+01	9.42E+01	9.64E+01
2999.779	9.53E+01	9.42E+01	9.64E+01
3000.261	9.53E+01	9.42E+01	9.63E+01
3000.744	9.53E+01	9.41E+01	9.63E+01
3001.226	9.53E+01	9.41E+01	9.63E+01
3001.708	9.53E+01	9.41E+01	9.63E+01
3002.19	9.52E+01	9.41E+01	9.62E+01
3002.672	9.52E+01	9.41E+01	9.62E+01
3003.154	9.52E+01	9.41E+01	9.63E+01
3003.636	9.51E+01	9.41E+01	9.63E+01
3004.118	9.51E+01	9.41E+01	9.63E+01
3004.601	9.51E+01	9.42E+01	9.63E+01

3005.083	9.51E+01	9.42E+01	9.64E+01
3005.565	9.52E+01	9.42E+01	9.64E+01
3006.047	9.52E+01	9.42E+01	9.64E+01
3006.529	9.52E+01	9.43E+01	9.64E+01
3007.011	9.52E+01	9.43E+01	9.64E+01
3007.493	9.52E+01	9.42E+01	9.64E+01
3007.975	9.52E+01	9.42E+01	9.64E+01
3008.458	9.52E+01	9.42E+01	9.64E+01
3008.94	9.52E+01	9.42E+01	9.64E+01
3009.422	9.52E+01	9.41E+01	9.64E+01
3009.904	9.52E+01	9.41E+01	9.64E+01
3010.386	9.52E+01	9.41E+01	9.64E+01
3010.868	9.52E+01	9.41E+01	9.64E+01
3011.35	9.52E+01	9.41E+01	9.64E+01
3011.833	9.52E+01	9.42E+01	9.64E+01
3012.314	9.53E+01	9.42E+01	9.65E+01
3012.797	9.53E+01	9.42E+01	9.65E+01
3013.279	9.53E+01	9.42E+01	9.65E+01
3013.761	9.53E+01	9.42E+01	9.65E+01
3014.243	9.53E+01	9.42E+01	9.64E+01
3014.725	9.53E+01	9.42E+01	9.64E+01
3015.207	9.53E+01	9.42E+01	9.64E+01
3015.689	9.53E+01	9.42E+01	9.64E+01
3016.172	9.53E+01	9.42E+01	9.64E+01
3016.654	9.53E+01	9.43E+01	9.64E+01
3017.136	9.53E+01	9.43E+01	9.64E+01
3017.618	9.53E+01	9.43E+01	9.64E+01
3018.1	9.53E+01	9.43E+01	9.64E+01
3018.582	9.53E+01	9.43E+01	9.64E+01
3019.064	9.53E+01	9.43E+01	9.65E+01
3019.546	9.53E+01	9.43E+01	9.65E+01
3020.029	9.53E+01	9.43E+01	9.65E+01
3020.511	9.53E+01	9.43E+01	9.65E+01
3020.993	9.53E+01	9.43E+01	9.64E+01
3021.475	9.53E+01	9.43E+01	9.64E+01
3021.957	9.53E+01	9.43E+01	9.64E+01
3022.439	9.53E+01	9.43E+01	9.64E+01
3022.921	9.52E+01	9.42E+01	9.64E+01
3023.403	9.52E+01	9.42E+01	9.64E+01
3023.885	9.52E+01	9.42E+01	9.64E+01
3024.368	9.52E+01	9.42E+01	9.64E+01
3024.85	9.52E+01	9.42E+01	9.64E+01
3025.332	9.52E+01	9.42E+01	9.64E+01
3025.814	9.52E+01	9.43E+01	9.63E+01
3026.296	9.52E+01	9.42E+01	9.63E+01
3026.778	9.53E+01	9.42E+01	9.63E+01
3027.26	9.53E+01	9.42E+01	9.64E+01

3027.742	9.53E+01	9.42E+01	9.64E+01
3028.225	9.53E+01	9.42E+01	9.64E+01
3028.707	9.53E+01	9.42E+01	9.64E+01
3029.189	9.53E+01	9.43E+01	9.64E+01
3029.671	9.52E+01	9.43E+01	9.63E+01
3030.153	9.52E+01	9.43E+01	9.63E+01
3030.635	9.52E+01	9.43E+01	9.63E+01
3031.117	9.52E+01	9.43E+01	9.63E+01
3031.6	9.52E+01	9.43E+01	9.64E+01
3032.082	9.53E+01	9.43E+01	9.64E+01
3032.564	9.53E+01	9.42E+01	9.64E+01
3033.046	9.53E+01	9.42E+01	9.64E+01
3033.528	9.53E+01	9.42E+01	9.65E+01
3034.01	9.53E+01	9.42E+01	9.65E+01
3034.492	9.53E+01	9.42E+01	9.65E+01
3034.974	9.53E+01	9.42E+01	9.65E+01
3035.457	9.53E+01	9.42E+01	9.64E+01
3035.939	9.53E+01	9.42E+01	9.64E+01
3036.421	9.53E+01	9.42E+01	9.64E+01
3036.903	9.53E+01	9.41E+01	9.64E+01
3037.385	9.53E+01	9.41E+01	9.64E+01
3037.867	9.53E+01	9.41E+01	9.64E+01
3038.349	9.53E+01	9.41E+01	9.64E+01
3038.831	9.53E+01	9.41E+01	9.64E+01
3039.313	9.53E+01	9.41E+01	9.64E+01
3039.796	9.53E+01	9.41E+01	9.64E+01
3040.278	9.53E+01	9.41E+01	9.63E+01
3040.76	9.53E+01	9.41E+01	9.63E+01
3041.242	9.53E+01	9.40E+01	9.63E+01
3041.724	9.53E+01	9.40E+01	9.63E+01
3042.206	9.53E+01	9.40E+01	9.63E+01
3042.688	9.53E+01	9.40E+01	9.63E+01
3043.17	9.53E+01	9.40E+01	9.63E+01
3043.653	9.53E+01	9.40E+01	9.63E+01
3044.135	9.53E+01	9.40E+01	9.63E+01
3044.617	9.53E+01	9.41E+01	9.63E+01
3045.099	9.54E+01	9.41E+01	9.63E+01
3045.581	9.53E+01	9.41E+01	9.63E+01
3046.063	9.53E+01	9.42E+01	9.63E+01
3046.545	9.53E+01	9.42E+01	9.63E+01
3047.028	9.53E+01	9.42E+01	9.63E+01
3047.51	9.53E+01	9.42E+01	9.63E+01
3047.992	9.53E+01	9.41E+01	9.63E+01
3048.474	9.53E+01	9.41E+01	9.63E+01
3048.956	9.54E+01	9.41E+01	9.63E+01
3049.438	9.54E+01	9.40E+01	9.63E+01
3049.92	9.54E+01	9.40E+01	9.63E+01

3050.402	9.54E+01	9.40E+01	9.63E+01
3050.885	9.54E+01	9.40E+01	9.63E+01
3051.367	9.54E+01	9.39E+01	9.63E+01
3051.849	9.54E+01	9.39E+01	9.63E+01
3052.331	9.54E+01	9.39E+01	9.63E+01
3052.813	9.54E+01	9.39E+01	9.63E+01
3053.295	9.54E+01	9.40E+01	9.63E+01
3053.777	9.54E+01	9.40E+01	9.63E+01
3054.259	9.54E+01	9.40E+01	9.63E+01
3054.741	9.54E+01	9.40E+01	9.63E+01
3055.224	9.54E+01	9.40E+01	9.63E+01
3055.706	9.54E+01	9.40E+01	9.63E+01
3056.188	9.54E+01	9.40E+01	9.63E+01
3056.67	9.54E+01	9.40E+01	9.63E+01
3057.152	9.54E+01	9.40E+01	9.63E+01
3057.634	9.53E+01	9.40E+01	9.63E+01
3058.116	9.53E+01	9.40E+01	9.63E+01
3058.598	9.53E+01	9.40E+01	9.63E+01
3059.081	9.53E+01	9.41E+01	9.63E+01
3059.563	9.53E+01	9.41E+01	9.63E+01
3060.045	9.53E+01	9.41E+01	9.63E+01
3060.527	9.53E+01	9.40E+01	9.63E+01
3061.009	9.53E+01	9.40E+01	9.63E+01
3061.491	9.52E+01	9.40E+01	9.63E+01
3061.973	9.52E+01	9.40E+01	9.62E+01
3062.456	9.52E+01	9.40E+01	9.62E+01
3062.938	9.51E+01	9.40E+01	9.62E+01
3063.42	9.51E+01	9.41E+01	9.62E+01
3063.902	9.51E+01	9.41E+01	9.62E+01
3064.384	9.51E+01	9.41E+01	9.62E+01
3064.866	9.51E+01	9.42E+01	9.62E+01
3065.348	9.51E+01	9.42E+01	9.63E+01
3065.83	9.51E+01	9.42E+01	9.63E+01
3066.313	9.52E+01	9.42E+01	9.63E+01
3066.795	9.52E+01	9.42E+01	9.63E+01
3067.277	9.52E+01	9.42E+01	9.64E+01
3067.759	9.53E+01	9.42E+01	9.64E+01
3068.241	9.53E+01	9.42E+01	9.64E+01
3068.723	9.54E+01	9.42E+01	9.65E+01
3069.205	9.54E+01	9.42E+01	9.65E+01
3069.687	9.55E+01	9.43E+01	9.65E+01
3070.169	9.55E+01	9.43E+01	9.66E+01
3070.652	9.55E+01	9.43E+01	9.66E+01
3071.134	9.55E+01	9.43E+01	9.66E+01
3071.616	9.55E+01	9.43E+01	9.66E+01
3072.098	9.55E+01	9.43E+01	9.66E+01
3072.58	9.54E+01	9.43E+01	9.66E+01

3073.062	9.54E+01	9.43E+01	9.65E+01
3073.544	9.54E+01	9.42E+01	9.65E+01
3074.026	9.54E+01	9.42E+01	9.65E+01
3074.509	9.54E+01	9.42E+01	9.65E+01
3074.991	9.54E+01	9.42E+01	9.64E+01
3075.473	9.54E+01	9.42E+01	9.64E+01
3075.955	9.54E+01	9.41E+01	9.64E+01
3076.437	9.54E+01	9.41E+01	9.63E+01
3076.919	9.53E+01	9.41E+01	9.63E+01
3077.401	9.53E+01	9.41E+01	9.63E+01
3077.884	9.53E+01	9.41E+01	9.63E+01
3078.365	9.53E+01	9.41E+01	9.63E+01
3078.848	9.53E+01	9.41E+01	9.63E+01
3079.33	9.53E+01	9.41E+01	9.63E+01
3079.812	9.52E+01	9.40E+01	9.63E+01
3080.294	9.52E+01	9.40E+01	9.62E+01
3080.776	9.52E+01	9.40E+01	9.62E+01
3081.258	9.53E+01	9.40E+01	9.62E+01
3081.74	9.53E+01	9.40E+01	9.62E+01
3082.223	9.53E+01	9.40E+01	9.62E+01
3082.705	9.53E+01	9.40E+01	9.62E+01
3083.187	9.53E+01	9.40E+01	9.62E+01
3083.669	9.53E+01	9.40E+01	9.62E+01
3084.151	9.54E+01	9.40E+01	9.62E+01
3084.633	9.54E+01	9.40E+01	9.62E+01
3085.115	9.54E+01	9.40E+01	9.62E+01
3085.597	9.54E+01	9.40E+01	9.62E+01
3086.08	9.55E+01	9.40E+01	9.63E+01
3086.562	9.55E+01	9.41E+01	9.63E+01
3087.044	9.55E+01	9.41E+01	9.64E+01
3087.526	9.55E+01	9.41E+01	9.64E+01
3088.008	9.56E+01	9.41E+01	9.65E+01
3088.49	9.56E+01	9.41E+01	9.65E+01
3088.972	9.56E+01	9.41E+01	9.65E+01
3089.454	9.55E+01	9.41E+01	9.65E+01
3089.937	9.55E+01	9.41E+01	9.64E+01
3090.419	9.55E+01	9.40E+01	9.64E+01
3090.901	9.55E+01	9.40E+01	9.64E+01
3091.383	9.54E+01	9.40E+01	9.64E+01
3091.865	9.54E+01	9.40E+01	9.64E+01
3092.347	9.53E+01	9.40E+01	9.63E+01
3092.829	9.53E+01	9.41E+01	9.63E+01
3093.312	9.53E+01	9.41E+01	9.63E+01
3093.794	9.53E+01	9.41E+01	9.63E+01
3094.276	9.53E+01	9.41E+01	9.63E+01
3094.758	9.53E+01	9.41E+01	9.63E+01
3095.24	9.53E+01	9.41E+01	9.63E+01

3095.722	9.54E+01	9.41E+01	9.63E+01
3096.204	9.54E+01	9.41E+01	9.63E+01
3096.686	9.54E+01	9.41E+01	9.63E+01
3097.168	9.54E+01	9.41E+01	9.63E+01
3097.651	9.54E+01	9.41E+01	9.63E+01
3098.133	9.54E+01	9.41E+01	9.63E+01
3098.615	9.53E+01	9.41E+01	9.63E+01
3099.097	9.53E+01	9.41E+01	9.63E+01
3099.579	9.53E+01	9.41E+01	9.63E+01
3100.061	9.52E+01	9.41E+01	9.63E+01
3100.543	9.52E+01	9.41E+01	9.63E+01
3101.025	9.52E+01	9.41E+01	9.63E+01
3101.508	9.52E+01	9.40E+01	9.63E+01
3101.99	9.52E+01	9.40E+01	9.63E+01
3102.472	9.53E+01	9.40E+01	9.63E+01
3102.954	9.53E+01	9.41E+01	9.63E+01
3103.436	9.53E+01	9.41E+01	9.63E+01
3103.918	9.53E+01	9.41E+01	9.63E+01
3104.4	9.54E+01	9.41E+01	9.63E+01
3104.883	9.54E+01	9.40E+01	9.63E+01
3105.365	9.54E+01	9.40E+01	9.63E+01
3105.847	9.54E+01	9.40E+01	9.63E+01
3106.329	9.54E+01	9.40E+01	9.63E+01
3106.811	9.54E+01	9.40E+01	9.63E+01
3107.293	9.54E+01	9.40E+01	9.63E+01
3107.775	9.54E+01	9.40E+01	9.63E+01
3108.257	9.55E+01	9.40E+01	9.63E+01
3108.74	9.55E+01	9.40E+01	9.63E+01
3109.222	9.55E+01	9.40E+01	9.63E+01
3109.704	9.55E+01	9.40E+01	9.63E+01
3110.186	9.55E+01	9.40E+01	9.63E+01
3110.668	9.55E+01	9.40E+01	9.64E+01
3111.15	9.54E+01	9.40E+01	9.64E+01
3111.632	9.54E+01	9.40E+01	9.64E+01
3112.114	9.54E+01	9.40E+01	9.64E+01
3112.596	9.54E+01	9.40E+01	9.64E+01
3113.079	9.54E+01	9.39E+01	9.64E+01
3113.561	9.53E+01	9.39E+01	9.64E+01
3114.043	9.53E+01	9.39E+01	9.64E+01
3114.525	9.53E+01	9.39E+01	9.64E+01
3115.007	9.53E+01	9.39E+01	9.64E+01
3115.489	9.53E+01	9.39E+01	9.64E+01
3115.971	9.53E+01	9.40E+01	9.64E+01
3116.453	9.54E+01	9.40E+01	9.64E+01
3116.936	9.54E+01	9.40E+01	9.64E+01
3117.418	9.54E+01	9.40E+01	9.64E+01
3117.9	9.54E+01	9.40E+01	9.64E+01

3118.382	9.53E+01	9.40E+01	9.64E+01
3118.864	9.53E+01	9.40E+01	9.63E+01
3119.346	9.53E+01	9.40E+01	9.63E+01
3119.828	9.53E+01	9.39E+01	9.63E+01
3120.311	9.53E+01	9.39E+01	9.63E+01
3120.792	9.53E+01	9.39E+01	9.62E+01
3121.275	9.53E+01	9.40E+01	9.62E+01
3121.757	9.53E+01	9.40E+01	9.62E+01
3122.239	9.53E+01	9.40E+01	9.62E+01
3122.721	9.53E+01	9.40E+01	9.62E+01
3123.203	9.53E+01	9.40E+01	9.62E+01
3123.685	9.53E+01	9.40E+01	9.62E+01
3124.167	9.53E+01	9.40E+01	9.62E+01
3124.65	9.53E+01	9.40E+01	9.63E+01
3125.132	9.53E+01	9.39E+01	9.63E+01
3125.614	9.53E+01	9.39E+01	9.63E+01
3126.096	9.53E+01	9.39E+01	9.63E+01
3126.578	9.53E+01	9.39E+01	9.63E+01
3127.06	9.53E+01	9.39E+01	9.63E+01
3127.542	9.54E+01	9.39E+01	9.63E+01
3128.024	9.54E+01	9.39E+01	9.64E+01
3128.507	9.54E+01	9.39E+01	9.64E+01
3128.989	9.54E+01	9.38E+01	9.64E+01
3129.471	9.54E+01	9.38E+01	9.64E+01
3129.953	9.54E+01	9.38E+01	9.63E+01
3130.435	9.54E+01	9.38E+01	9.63E+01
3130.917	9.53E+01	9.37E+01	9.62E+01
3131.399	9.53E+01	9.37E+01	9.61E+01
3131.881	9.52E+01	9.37E+01	9.61E+01
3132.364	9.52E+01	9.37E+01	9.61E+01
3132.846	9.52E+01	9.37E+01	9.60E+01
3133.328	9.52E+01	9.37E+01	9.61E+01
3133.81	9.52E+01	9.37E+01	9.61E+01
3134.292	9.53E+01	9.38E+01	9.61E+01
3134.774	9.53E+01	9.38E+01	9.62E+01
3135.256	9.53E+01	9.38E+01	9.62E+01
3135.739	9.54E+01	9.38E+01	9.63E+01
3136.22	9.54E+01	9.39E+01	9.63E+01
3136.703	9.54E+01	9.39E+01	9.63E+01
3137.185	9.54E+01	9.39E+01	9.63E+01
3137.667	9.54E+01	9.39E+01	9.63E+01
3138.149	9.54E+01	9.39E+01	9.63E+01
3138.631	9.54E+01	9.39E+01	9.63E+01
3139.113	9.54E+01	9.39E+01	9.63E+01
3139.595	9.54E+01	9.39E+01	9.63E+01
3140.078	9.54E+01	9.39E+01	9.63E+01
3140.56	9.54E+01	9.39E+01	9.63E+01

3141.042	9.54E+01	9.39E+01	9.63E+01
3141.524	9.53E+01	9.39E+01	9.63E+01
3142.006	9.53E+01	9.39E+01	9.63E+01
3142.488	9.53E+01	9.39E+01	9.63E+01
3142.97	9.53E+01	9.39E+01	9.63E+01
3143.452	9.53E+01	9.39E+01	9.62E+01
3143.935	9.54E+01	9.39E+01	9.62E+01
3144.417	9.54E+01	9.38E+01	9.62E+01
3144.899	9.54E+01	9.38E+01	9.61E+01
3145.381	9.54E+01	9.37E+01	9.61E+01
3145.863	9.54E+01	9.37E+01	9.61E+01
3146.345	9.54E+01	9.37E+01	9.61E+01
3146.827	9.54E+01	9.37E+01	9.61E+01
3147.309	9.54E+01	9.37E+01	9.61E+01
3147.792	9.54E+01	9.37E+01	9.61E+01
3148.274	9.54E+01	9.37E+01	9.61E+01
3148.756	9.53E+01	9.37E+01	9.61E+01
3149.238	9.53E+01	9.37E+01	9.61E+01
3149.72	9.52E+01	9.37E+01	9.61E+01
3150.202	9.52E+01	9.37E+01	9.61E+01
3150.684	9.52E+01	9.37E+01	9.61E+01
3151.167	9.52E+01	9.37E+01	9.61E+01
3151.648	9.52E+01	9.37E+01	9.62E+01
3152.131	9.52E+01	9.37E+01	9.62E+01
3152.613	9.52E+01	9.38E+01	9.62E+01
3153.095	9.53E+01	9.38E+01	9.62E+01
3153.577	9.53E+01	9.38E+01	9.62E+01
3154.059	9.53E+01	9.37E+01	9.62E+01
3154.541	9.53E+01	9.37E+01	9.62E+01
3155.023	9.53E+01	9.36E+01	9.62E+01
3155.506	9.53E+01	9.36E+01	9.62E+01
3155.988	9.53E+01	9.36E+01	9.62E+01
3156.47	9.53E+01	9.35E+01	9.62E+01
3156.952	9.53E+01	9.35E+01	9.61E+01
3157.434	9.53E+01	9.35E+01	9.61E+01
3157.916	9.53E+01	9.36E+01	9.61E+01
3158.398	9.53E+01	9.36E+01	9.61E+01
3158.88	9.53E+01	9.36E+01	9.61E+01
3159.363	9.53E+01	9.36E+01	9.61E+01
3159.845	9.52E+01	9.37E+01	9.62E+01
3160.327	9.52E+01	9.37E+01	9.62E+01
3160.809	9.52E+01	9.37E+01	9.62E+01
3161.291	9.52E+01	9.37E+01	9.62E+01
3161.773	9.52E+01	9.37E+01	9.62E+01
3162.255	9.52E+01	9.37E+01	9.62E+01
3162.737	9.52E+01	9.37E+01	9.62E+01
3163.219	9.52E+01	9.37E+01	9.62E+01

3163.702	9.52E+01	9.36E+01	9.63E+01
3164.184	9.53E+01	9.36E+01	9.63E+01
3164.666	9.53E+01	9.36E+01	9.63E+01
3165.148	9.53E+01	9.36E+01	9.63E+01
3165.63	9.54E+01	9.36E+01	9.63E+01
3166.112	9.54E+01	9.36E+01	9.64E+01
3166.594	9.54E+01	9.36E+01	9.64E+01
3167.076	9.54E+01	9.36E+01	9.64E+01
3167.559	9.53E+01	9.35E+01	9.63E+01
3168.041	9.53E+01	9.35E+01	9.63E+01
3168.523	9.53E+01	9.35E+01	9.62E+01
3169.005	9.53E+01	9.34E+01	9.62E+01
3169.487	9.52E+01	9.34E+01	9.61E+01
3169.969	9.52E+01	9.34E+01	9.61E+01
3170.451	9.52E+01	9.34E+01	9.60E+01
3170.934	9.52E+01	9.34E+01	9.60E+01
3171.416	9.52E+01	9.35E+01	9.60E+01
3171.898	9.52E+01	9.35E+01	9.60E+01
3172.38	9.52E+01	9.35E+01	9.60E+01
3172.862	9.52E+01	9.35E+01	9.60E+01
3173.344	9.52E+01	9.35E+01	9.60E+01
3173.826	9.52E+01	9.35E+01	9.60E+01
3174.308	9.52E+01	9.35E+01	9.60E+01
3174.791	9.52E+01	9.36E+01	9.60E+01
3175.273	9.52E+01	9.36E+01	9.60E+01
3175.755	9.52E+01	9.36E+01	9.60E+01
3176.237	9.52E+01	9.36E+01	9.60E+01
3176.719	9.52E+01	9.36E+01	9.60E+01
3177.201	9.53E+01	9.36E+01	9.61E+01
3177.683	9.53E+01	9.37E+01	9.61E+01
3178.165	9.54E+01	9.37E+01	9.61E+01
3178.647	9.54E+01	9.37E+01	9.62E+01
3179.13	9.54E+01	9.37E+01	9.62E+01
3179.612	9.54E+01	9.37E+01	9.62E+01
3180.094	9.54E+01	9.37E+01	9.62E+01
3180.576	9.54E+01	9.37E+01	9.62E+01
3181.058	9.54E+01	9.36E+01	9.62E+01
3181.54	9.54E+01	9.36E+01	9.62E+01
3182.022	9.54E+01	9.36E+01	9.62E+01
3182.504	9.53E+01	9.36E+01	9.62E+01
3182.987	9.53E+01	9.36E+01	9.62E+01
3183.469	9.53E+01	9.35E+01	9.62E+01
3183.951	9.52E+01	9.35E+01	9.62E+01
3184.433	9.52E+01	9.35E+01	9.61E+01
3184.915	9.52E+01	9.35E+01	9.61E+01
3185.397	9.52E+01	9.35E+01	9.62E+01
3185.879	9.52E+01	9.35E+01	9.62E+01

3186.362	9.53E+01	9.36E+01	9.62E+01
3186.844	9.53E+01	9.36E+01	9.62E+01
3187.326	9.53E+01	9.36E+01	9.62E+01
3187.808	9.53E+01	9.36E+01	9.62E+01
3188.29	9.53E+01	9.36E+01	9.61E+01
3188.772	9.53E+01	9.36E+01	9.61E+01
3189.254	9.53E+01	9.36E+01	9.61E+01
3189.736	9.52E+01	9.36E+01	9.60E+01
3190.219	9.52E+01	9.36E+01	9.60E+01
3190.701	9.52E+01	9.36E+01	9.60E+01
3191.183	9.52E+01	9.36E+01	9.59E+01
3191.665	9.52E+01	9.35E+01	9.59E+01
3192.147	9.52E+01	9.35E+01	9.59E+01
3192.629	9.52E+01	9.35E+01	9.59E+01
3193.111	9.52E+01	9.35E+01	9.58E+01
3193.593	9.52E+01	9.35E+01	9.58E+01
3194.075	9.52E+01	9.35E+01	9.58E+01
3194.558	9.52E+01	9.35E+01	9.59E+01
3195.04	9.52E+01	9.35E+01	9.59E+01
3195.522	9.52E+01	9.35E+01	9.59E+01
3196.004	9.52E+01	9.35E+01	9.59E+01
3196.486	9.52E+01	9.35E+01	9.59E+01
3196.968	9.53E+01	9.35E+01	9.59E+01
3197.45	9.53E+01	9.34E+01	9.59E+01
3197.933	9.53E+01	9.34E+01	9.60E+01
3198.415	9.53E+01	9.34E+01	9.60E+01
3198.897	9.53E+01	9.34E+01	9.60E+01
3199.379	9.53E+01	9.34E+01	9.60E+01
3199.861	9.53E+01	9.34E+01	9.60E+01
3200.343	9.53E+01	9.34E+01	9.60E+01
3200.825	9.52E+01	9.34E+01	9.60E+01
3201.307	9.52E+01	9.34E+01	9.60E+01
3201.79	9.52E+01	9.34E+01	9.59E+01
3202.272	9.52E+01	9.34E+01	9.59E+01
3202.754	9.51E+01	9.34E+01	9.59E+01
3203.236	9.51E+01	9.34E+01	9.59E+01
3203.718	9.51E+01	9.33E+01	9.59E+01
3204.2	9.51E+01	9.33E+01	9.58E+01
3204.682	9.51E+01	9.33E+01	9.59E+01
3205.164	9.51E+01	9.33E+01	9.59E+01
3205.646	9.52E+01	9.33E+01	9.59E+01
3206.129	9.52E+01	9.34E+01	9.60E+01
3206.611	9.53E+01	9.34E+01	9.60E+01
3207.093	9.53E+01	9.34E+01	9.61E+01
3207.575	9.53E+01	9.35E+01	9.61E+01
3208.057	9.54E+01	9.35E+01	9.61E+01
3208.539	9.54E+01	9.35E+01	9.61E+01

3209.021	9.53E+01	9.35E+01	9.61E+01
3209.503	9.53E+01	9.35E+01	9.61E+01
3209.986	9.53E+01	9.35E+01	9.61E+01
3210.468	9.52E+01	9.35E+01	9.61E+01
3210.95	9.52E+01	9.35E+01	9.61E+01
3211.432	9.52E+01	9.35E+01	9.61E+01
3211.914	9.51E+01	9.35E+01	9.61E+01
3212.396	9.52E+01	9.35E+01	9.61E+01
3212.878	9.52E+01	9.35E+01	9.61E+01
3213.361	9.52E+01	9.35E+01	9.61E+01
3213.843	9.52E+01	9.35E+01	9.61E+01
3214.325	9.52E+01	9.35E+01	9.61E+01
3214.807	9.51E+01	9.35E+01	9.60E+01
3215.289	9.51E+01	9.34E+01	9.60E+01
3215.771	9.51E+01	9.34E+01	9.60E+01
3216.253	9.51E+01	9.34E+01	9.60E+01
3216.735	9.51E+01	9.34E+01	9.60E+01
3217.218	9.51E+01	9.33E+01	9.59E+01
3217.7	9.51E+01	9.33E+01	9.59E+01
3218.182	9.51E+01	9.33E+01	9.59E+01
3218.664	9.51E+01	9.33E+01	9.59E+01
3219.146	9.51E+01	9.32E+01	9.59E+01
3219.628	9.51E+01	9.32E+01	9.59E+01
3220.11	9.52E+01	9.33E+01	9.59E+01
3220.592	9.52E+01	9.33E+01	9.60E+01
3221.074	9.52E+01	9.33E+01	9.60E+01
3221.557	9.52E+01	9.33E+01	9.60E+01
3222.039	9.53E+01	9.33E+01	9.61E+01
3222.521	9.53E+01	9.33E+01	9.61E+01
3223.003	9.53E+01	9.33E+01	9.61E+01
3223.485	9.53E+01	9.33E+01	9.61E+01
3223.967	9.53E+01	9.33E+01	9.61E+01
3224.449	9.53E+01	9.33E+01	9.61E+01
3224.931	9.52E+01	9.33E+01	9.61E+01
3225.414	9.52E+01	9.33E+01	9.61E+01
3225.896	9.52E+01	9.33E+01	9.61E+01
3226.378	9.52E+01	9.33E+01	9.61E+01
3226.86	9.52E+01	9.33E+01	9.60E+01
3227.342	9.51E+01	9.33E+01	9.60E+01
3227.824	9.51E+01	9.34E+01	9.60E+01
3228.306	9.51E+01	9.34E+01	9.60E+01
3228.789	9.51E+01	9.34E+01	9.60E+01
3229.271	9.51E+01	9.34E+01	9.60E+01
3229.753	9.51E+01	9.34E+01	9.60E+01
3230.235	9.51E+01	9.34E+01	9.60E+01
3230.717	9.50E+01	9.34E+01	9.60E+01
3231.199	9.50E+01	9.34E+01	9.60E+01

3231.681	9.50E+01	9.34E+01	9.60E+01
3232.163	9.50E+01	9.34E+01	9.59E+01
3232.646	9.50E+01	9.33E+01	9.59E+01
3233.128	9.51E+01	9.33E+01	9.59E+01
3233.61	9.51E+01	9.33E+01	9.59E+01
3234.092	9.51E+01	9.33E+01	9.59E+01
3234.574	9.51E+01	9.33E+01	9.59E+01
3235.056	9.51E+01	9.33E+01	9.59E+01
3235.538	9.51E+01	9.32E+01	9.59E+01
3236.02	9.51E+01	9.32E+01	9.59E+01
3236.502	9.51E+01	9.32E+01	9.59E+01
3236.985	9.51E+01	9.32E+01	9.59E+01
3237.467	9.51E+01	9.32E+01	9.59E+01
3237.949	9.51E+01	9.32E+01	9.59E+01
3238.431	9.51E+01	9.32E+01	9.59E+01
3238.913	9.51E+01	9.32E+01	9.59E+01
3239.395	9.51E+01	9.33E+01	9.59E+01
3239.877	9.51E+01	9.33E+01	9.59E+01
3240.359	9.51E+01	9.33E+01	9.59E+01
3240.842	9.51E+01	9.33E+01	9.59E+01
3241.324	9.51E+01	9.33E+01	9.59E+01
3241.806	9.51E+01	9.33E+01	9.59E+01
3242.288	9.51E+01	9.33E+01	9.59E+01
3242.77	9.51E+01	9.32E+01	9.60E+01
3243.252	9.51E+01	9.32E+01	9.60E+01
3243.734	9.51E+01	9.32E+01	9.60E+01
3244.217	9.51E+01	9.31E+01	9.60E+01
3244.698	9.52E+01	9.31E+01	9.59E+01
3245.181	9.52E+01	9.31E+01	9.59E+01
3245.663	9.52E+01	9.31E+01	9.59E+01
3246.145	9.52E+01	9.31E+01	9.59E+01
3246.627	9.52E+01	9.31E+01	9.59E+01
3247.109	9.52E+01	9.32E+01	9.58E+01
3247.591	9.51E+01	9.32E+01	9.58E+01
3248.073	9.51E+01	9.32E+01	9.58E+01
3248.556	9.51E+01	9.32E+01	9.58E+01
3249.038	9.50E+01	9.31E+01	9.58E+01
3249.52	9.50E+01	9.31E+01	9.58E+01
3250.002	9.50E+01	9.31E+01	9.58E+01
3250.484	9.50E+01	9.31E+01	9.58E+01
3250.966	9.51E+01	9.30E+01	9.58E+01
3251.448	9.51E+01	9.30E+01	9.58E+01
3251.93	9.51E+01	9.30E+01	9.58E+01
3252.413	9.51E+01	9.30E+01	9.59E+01
3252.895	9.51E+01	9.30E+01	9.59E+01
3253.377	9.51E+01	9.30E+01	9.59E+01
3253.859	9.51E+01	9.30E+01	9.59E+01

3254.341	9.50E+01	9.30E+01	9.59E+01
3254.823	9.50E+01	9.30E+01	9.59E+01
3255.305	9.50E+01	9.30E+01	9.60E+01
3255.787	9.50E+01	9.30E+01	9.60E+01
3256.27	9.50E+01	9.30E+01	9.60E+01
3256.752	9.50E+01	9.30E+01	9.60E+01
3257.234	9.50E+01	9.30E+01	9.61E+01
3257.716	9.50E+01	9.31E+01	9.61E+01
3258.198	9.51E+01	9.31E+01	9.61E+01
3258.68	9.51E+01	9.32E+01	9.61E+01
3259.162	9.51E+01	9.32E+01	9.61E+01
3259.645	9.51E+01	9.33E+01	9.60E+01
3260.126	9.51E+01	9.33E+01	9.60E+01
3260.609	9.52E+01	9.33E+01	9.59E+01
3261.091	9.52E+01	9.33E+01	9.59E+01
3261.573	9.52E+01	9.32E+01	9.58E+01
3262.055	9.52E+01	9.32E+01	9.58E+01
3262.537	9.51E+01	9.31E+01	9.58E+01
3263.019	9.51E+01	9.31E+01	9.58E+01
3263.501	9.51E+01	9.31E+01	9.58E+01
3263.984	9.51E+01	9.31E+01	9.58E+01
3264.466	9.51E+01	9.31E+01	9.58E+01
3264.948	9.51E+01	9.31E+01	9.59E+01
3265.43	9.51E+01	9.31E+01	9.59E+01
3265.912	9.51E+01	9.31E+01	9.59E+01
3266.394	9.51E+01	9.31E+01	9.59E+01
3266.876	9.50E+01	9.31E+01	9.59E+01
3267.358	9.50E+01	9.31E+01	9.59E+01
3267.841	9.51E+01	9.31E+01	9.59E+01
3268.323	9.51E+01	9.30E+01	9.59E+01
3268.805	9.51E+01	9.30E+01	9.60E+01
3269.287	9.51E+01	9.30E+01	9.60E+01
3269.769	9.52E+01	9.30E+01	9.59E+01
3270.251	9.52E+01	9.30E+01	9.59E+01
3270.733	9.52E+01	9.31E+01	9.59E+01
3271.215	9.52E+01	9.31E+01	9.59E+01
3271.698	9.52E+01	9.31E+01	9.59E+01
3272.18	9.52E+01	9.31E+01	9.58E+01
3272.662	9.52E+01	9.31E+01	9.58E+01
3273.144	9.52E+01	9.31E+01	9.58E+01
3273.626	9.51E+01	9.31E+01	9.59E+01
3274.108	9.51E+01	9.31E+01	9.59E+01
3274.59	9.51E+01	9.31E+01	9.59E+01
3275.073	9.51E+01	9.31E+01	9.59E+01
3275.554	9.50E+01	9.31E+01	9.59E+01
3276.037	9.50E+01	9.31E+01	9.59E+01
3276.519	9.50E+01	9.31E+01	9.59E+01

3277.001	9.50E+01	9.31E+01	9.59E+01
3277.483	9.50E+01	9.30E+01	9.58E+01
3277.965	9.51E+01	9.30E+01	9.58E+01
3278.447	9.51E+01	9.30E+01	9.58E+01
3278.929	9.51E+01	9.30E+01	9.57E+01
3279.412	9.51E+01	9.30E+01	9.57E+01
3279.894	9.51E+01	9.30E+01	9.57E+01
3280.376	9.51E+01	9.31E+01	9.57E+01
3280.858	9.51E+01	9.31E+01	9.57E+01
3281.34	9.51E+01	9.31E+01	9.58E+01
3281.822	9.52E+01	9.32E+01	9.58E+01
3282.304	9.52E+01	9.32E+01	9.58E+01
3282.786	9.52E+01	9.32E+01	9.59E+01
3283.269	9.52E+01	9.32E+01	9.59E+01
3283.751	9.52E+01	9.32E+01	9.59E+01
3284.233	9.52E+01	9.32E+01	9.59E+01
3284.715	9.52E+01	9.32E+01	9.59E+01
3285.197	9.52E+01	9.32E+01	9.59E+01
3285.679	9.51E+01	9.32E+01	9.59E+01
3286.161	9.52E+01	9.32E+01	9.60E+01
3286.643	9.52E+01	9.32E+01	9.60E+01
3287.125	9.52E+01	9.32E+01	9.60E+01
3287.608	9.52E+01	9.32E+01	9.60E+01
3288.09	9.52E+01	9.32E+01	9.60E+01
3288.572	9.53E+01	9.32E+01	9.60E+01
3289.054	9.53E+01	9.31E+01	9.59E+01
3289.536	9.52E+01	9.31E+01	9.59E+01
3290.018	9.52E+01	9.31E+01	9.59E+01
3290.5	9.52E+01	9.30E+01	9.59E+01
3290.982	9.52E+01	9.30E+01	9.59E+01
3291.465	9.52E+01	9.29E+01	9.59E+01
3291.947	9.52E+01	9.29E+01	9.58E+01
3292.429	9.51E+01	9.29E+01	9.58E+01
3292.911	9.51E+01	9.29E+01	9.58E+01
3293.393	9.51E+01	9.29E+01	9.58E+01
3293.875	9.51E+01	9.29E+01	9.58E+01
3294.357	9.50E+01	9.29E+01	9.58E+01
3294.84	9.50E+01	9.29E+01	9.58E+01
3295.322	9.50E+01	9.29E+01	9.58E+01
3295.804	9.50E+01	9.29E+01	9.58E+01
3296.286	9.50E+01	9.29E+01	9.58E+01
3296.768	9.50E+01	9.29E+01	9.58E+01
3297.25	9.51E+01	9.30E+01	9.58E+01
3297.732	9.51E+01	9.30E+01	9.58E+01
3298.214	9.51E+01	9.30E+01	9.58E+01
3298.697	9.51E+01	9.30E+01	9.58E+01
3299.179	9.51E+01	9.30E+01	9.59E+01

3299.661	9.51E+01	9.30E+01	9.59E+01
3300.143	9.51E+01	9.30E+01	9.59E+01
3300.625	9.51E+01	9.30E+01	9.59E+01
3301.107	9.51E+01	9.30E+01	9.59E+01
3301.589	9.51E+01	9.30E+01	9.59E+01
3302.071	9.51E+01	9.30E+01	9.59E+01
3302.553	9.51E+01	9.31E+01	9.59E+01
3303.036	9.52E+01	9.31E+01	9.59E+01
3303.518	9.52E+01	9.31E+01	9.60E+01
3304	9.52E+01	9.31E+01	9.60E+01
3304.482	9.52E+01	9.31E+01	9.60E+01
3304.964	9.53E+01	9.31E+01	9.60E+01
3305.446	9.53E+01	9.30E+01	9.60E+01
3305.928	9.53E+01	9.30E+01	9.60E+01
3306.411	9.53E+01	9.30E+01	9.60E+01
3306.893	9.53E+01	9.30E+01	9.59E+01
3307.375	9.52E+01	9.30E+01	9.59E+01
3307.857	9.52E+01	9.30E+01	9.59E+01
3308.339	9.52E+01	9.30E+01	9.59E+01
3308.821	9.52E+01	9.30E+01	9.58E+01
3309.303	9.52E+01	9.30E+01	9.58E+01
3309.785	9.52E+01	9.30E+01	9.58E+01
3310.268	9.51E+01	9.30E+01	9.58E+01
3310.75	9.51E+01	9.30E+01	9.58E+01
3311.232	9.51E+01	9.31E+01	9.58E+01
3311.714	9.51E+01	9.31E+01	9.58E+01
3312.196	9.51E+01	9.31E+01	9.59E+01
3312.678	9.51E+01	9.31E+01	9.59E+01
3313.16	9.51E+01	9.31E+01	9.59E+01
3313.642	9.51E+01	9.31E+01	9.59E+01
3314.125	9.52E+01	9.32E+01	9.60E+01
3314.607	9.52E+01	9.32E+01	9.60E+01
3315.089	9.52E+01	9.32E+01	9.61E+01
3315.571	9.53E+01	9.33E+01	9.61E+01
3316.053	9.53E+01	9.33E+01	9.61E+01
3316.535	9.53E+01	9.32E+01	9.60E+01
3317.017	9.52E+01	9.32E+01	9.60E+01
3317.5	9.52E+01	9.31E+01	9.59E+01
3317.981	9.51E+01	9.30E+01	9.58E+01
3318.464	9.51E+01	9.30E+01	9.57E+01
3318.946	9.50E+01	9.29E+01	9.56E+01
3319.428	9.50E+01	9.29E+01	9.56E+01
3319.91	9.49E+01	9.28E+01	9.56E+01
3320.392	9.49E+01	9.28E+01	9.56E+01
3320.874	9.49E+01	9.28E+01	9.56E+01
3321.356	9.49E+01	9.28E+01	9.56E+01
3321.839	9.50E+01	9.29E+01	9.57E+01

3322.321	9.50E+01	9.29E+01	9.58E+01
3322.803	9.50E+01	9.29E+01	9.58E+01
3323.285	9.51E+01	9.29E+01	9.59E+01
3323.767	9.51E+01	9.29E+01	9.59E+01
3324.249	9.51E+01	9.29E+01	9.59E+01
3324.731	9.50E+01	9.29E+01	9.59E+01
3325.213	9.50E+01	9.29E+01	9.59E+01
3325.696	9.50E+01	9.29E+01	9.59E+01
3326.178	9.50E+01	9.29E+01	9.59E+01
3326.66	9.50E+01	9.29E+01	9.59E+01
3327.142	9.50E+01	9.29E+01	9.59E+01
3327.624	9.51E+01	9.29E+01	9.59E+01
3328.106	9.51E+01	9.29E+01	9.58E+01
3328.588	9.51E+01	9.29E+01	9.58E+01
3329.07	9.51E+01	9.29E+01	9.58E+01
3329.552	9.51E+01	9.29E+01	9.58E+01
3330.035	9.51E+01	9.29E+01	9.58E+01
3330.517	9.51E+01	9.29E+01	9.57E+01
3330.999	9.50E+01	9.29E+01	9.57E+01
3331.481	9.50E+01	9.29E+01	9.57E+01
3331.963	9.50E+01	9.29E+01	9.57E+01
3332.445	9.50E+01	9.29E+01	9.57E+01
3332.927	9.50E+01	9.30E+01	9.57E+01
3333.409	9.50E+01	9.30E+01	9.57E+01
3333.892	9.51E+01	9.29E+01	9.57E+01
3334.374	9.51E+01	9.29E+01	9.57E+01
3334.856	9.51E+01	9.29E+01	9.57E+01
3335.338	9.51E+01	9.29E+01	9.57E+01
3335.82	9.51E+01	9.29E+01	9.57E+01
3336.302	9.51E+01	9.28E+01	9.57E+01
3336.784	9.51E+01	9.28E+01	9.57E+01
3337.267	9.51E+01	9.28E+01	9.57E+01
3337.749	9.51E+01	9.28E+01	9.57E+01
3338.231	9.51E+01	9.28E+01	9.58E+01
3338.713	9.51E+01	9.28E+01	9.58E+01
3339.195	9.51E+01	9.29E+01	9.58E+01
3339.677	9.51E+01	9.29E+01	9.58E+01
3340.159	9.51E+01	9.29E+01	9.58E+01
3340.641	9.51E+01	9.29E+01	9.58E+01
3341.124	9.51E+01	9.29E+01	9.58E+01
3341.606	9.50E+01	9.29E+01	9.58E+01
3342.088	9.50E+01	9.28E+01	9.58E+01
3342.57	9.50E+01	9.28E+01	9.58E+01
3343.052	9.50E+01	9.28E+01	9.58E+01
3343.534	9.51E+01	9.28E+01	9.58E+01
3344.016	9.51E+01	9.28E+01	9.58E+01
3344.498	9.51E+01	9.28E+01	9.58E+01

3344.98	9.51E+01	9.28E+01	9.58E+01
3345.463	9.51E+01	9.28E+01	9.58E+01
3345.945	9.51E+01	9.28E+01	9.58E+01
3346.427	9.51E+01	9.28E+01	9.58E+01
3346.909	9.51E+01	9.28E+01	9.57E+01
3347.391	9.51E+01	9.28E+01	9.57E+01
3347.873	9.51E+01	9.28E+01	9.57E+01
3348.355	9.51E+01	9.28E+01	9.57E+01
3348.837	9.51E+01	9.28E+01	9.58E+01
3349.32	9.51E+01	9.28E+01	9.58E+01
3349.802	9.51E+01	9.28E+01	9.58E+01
3350.284	9.51E+01	9.28E+01	9.58E+01
3350.766	9.51E+01	9.28E+01	9.58E+01
3351.248	9.51E+01	9.28E+01	9.59E+01
3351.73	9.51E+01	9.28E+01	9.59E+01
3352.212	9.51E+01	9.29E+01	9.59E+01
3352.695	9.50E+01	9.29E+01	9.59E+01
3353.177	9.50E+01	9.29E+01	9.59E+01
3353.659	9.50E+01	9.29E+01	9.59E+01
3354.141	9.50E+01	9.30E+01	9.59E+01
3354.623	9.50E+01	9.30E+01	9.59E+01
3355.105	9.50E+01	9.30E+01	9.58E+01
3355.587	9.50E+01	9.30E+01	9.58E+01
3356.069	9.50E+01	9.30E+01	9.58E+01
3356.552	9.50E+01	9.29E+01	9.58E+01
3357.034	9.50E+01	9.29E+01	9.57E+01
3357.516	9.50E+01	9.29E+01	9.57E+01
3357.998	9.50E+01	9.28E+01	9.57E+01
3358.48	9.50E+01	9.28E+01	9.57E+01
3358.962	9.50E+01	9.28E+01	9.57E+01
3359.444	9.49E+01	9.28E+01	9.57E+01
3359.926	9.49E+01	9.29E+01	9.57E+01
3360.408	9.49E+01	9.29E+01	9.57E+01
3360.891	9.49E+01	9.29E+01	9.58E+01
3361.373	9.49E+01	9.29E+01	9.58E+01
3361.855	9.49E+01	9.29E+01	9.58E+01
3362.337	9.49E+01	9.29E+01	9.59E+01
3362.819	9.49E+01	9.29E+01	9.59E+01
3363.301	9.49E+01	9.29E+01	9.59E+01
3363.783	9.49E+01	9.29E+01	9.59E+01
3364.265	9.49E+01	9.28E+01	9.59E+01
3364.748	9.48E+01	9.28E+01	9.59E+01
3365.23	9.48E+01	9.27E+01	9.58E+01
3365.712	9.48E+01	9.27E+01	9.58E+01
3366.194	9.48E+01	9.27E+01	9.57E+01
3366.676	9.48E+01	9.27E+01	9.57E+01
3367.158	9.48E+01	9.26E+01	9.57E+01

3367.64	9.47E+01	9.27E+01	9.56E+01
3368.123	9.48E+01	9.27E+01	9.56E+01
3368.604	9.48E+01	9.27E+01	9.57E+01
3369.087	9.48E+01	9.27E+01	9.57E+01
3369.569	9.48E+01	9.27E+01	9.57E+01
3370.051	9.48E+01	9.27E+01	9.57E+01
3370.533	9.48E+01	9.28E+01	9.56E+01
3371.015	9.48E+01	9.28E+01	9.56E+01
3371.497	9.48E+01	9.28E+01	9.56E+01
3371.979	9.49E+01	9.28E+01	9.56E+01
3372.462	9.49E+01	9.29E+01	9.56E+01
3372.944	9.49E+01	9.29E+01	9.56E+01
3373.426	9.49E+01	9.29E+01	9.56E+01
3373.908	9.49E+01	9.29E+01	9.56E+01
3374.39	9.49E+01	9.29E+01	9.57E+01
3374.872	9.50E+01	9.29E+01	9.57E+01
3375.354	9.50E+01	9.29E+01	9.57E+01
3375.836	9.50E+01	9.29E+01	9.57E+01
3376.319	9.50E+01	9.29E+01	9.57E+01
3376.801	9.50E+01	9.29E+01	9.58E+01
3377.283	9.51E+01	9.29E+01	9.58E+01
3377.765	9.51E+01	9.30E+01	9.58E+01
3378.247	9.51E+01	9.30E+01	9.59E+01
3378.729	9.51E+01	9.30E+01	9.59E+01
3379.211	9.51E+01	9.30E+01	9.59E+01
3379.693	9.51E+01	9.30E+01	9.59E+01
3380.176	9.51E+01	9.30E+01	9.59E+01
3380.658	9.52E+01	9.30E+01	9.59E+01
3381.14	9.51E+01	9.30E+01	9.59E+01
3381.622	9.51E+01	9.30E+01	9.59E+01
3382.104	9.51E+01	9.30E+01	9.58E+01
3382.586	9.51E+01	9.30E+01	9.58E+01
3383.068	9.50E+01	9.30E+01	9.58E+01
3383.551	9.50E+01	9.30E+01	9.58E+01
3384.032	9.50E+01	9.30E+01	9.58E+01
3384.515	9.50E+01	9.30E+01	9.58E+01
3384.997	9.50E+01	9.30E+01	9.58E+01
3385.479	9.50E+01	9.30E+01	9.58E+01
3385.961	9.51E+01	9.30E+01	9.58E+01
3386.443	9.51E+01	9.31E+01	9.59E+01
3386.925	9.52E+01	9.31E+01	9.59E+01
3387.407	9.52E+01	9.32E+01	9.59E+01
3387.89	9.53E+01	9.32E+01	9.59E+01
3388.372	9.53E+01	9.32E+01	9.59E+01
3388.854	9.53E+01	9.32E+01	9.59E+01
3389.336	9.53E+01	9.32E+01	9.59E+01
3389.818	9.52E+01	9.32E+01	9.59E+01

3390.3	9.52E+01	9.31E+01	9.59E+01
3390.782	9.51E+01	9.30E+01	9.59E+01
3391.264	9.51E+01	9.30E+01	9.59E+01
3391.747	9.50E+01	9.29E+01	9.59E+01
3392.229	9.50E+01	9.29E+01	9.59E+01
3392.711	9.50E+01	9.29E+01	9.59E+01
3393.193	9.49E+01	9.29E+01	9.59E+01
3393.675	9.49E+01	9.29E+01	9.59E+01
3394.157	9.49E+01	9.30E+01	9.59E+01
3394.639	9.49E+01	9.30E+01	9.58E+01
3395.121	9.49E+01	9.30E+01	9.58E+01
3395.604	9.49E+01	9.30E+01	9.58E+01
3396.086	9.49E+01	9.30E+01	9.58E+01
3396.568	9.49E+01	9.30E+01	9.58E+01
3397.05	9.49E+01	9.30E+01	9.58E+01
3397.532	9.48E+01	9.30E+01	9.58E+01
3398.014	9.49E+01	9.29E+01	9.58E+01
3398.496	9.49E+01	9.29E+01	9.58E+01
3398.979	9.49E+01	9.29E+01	9.59E+01
3399.461	9.50E+01	9.29E+01	9.59E+01
3399.943	9.50E+01	9.29E+01	9.60E+01
3400.425	9.51E+01	9.30E+01	9.60E+01
3400.907	9.51E+01	9.30E+01	9.60E+01
3401.389	9.51E+01	9.30E+01	9.60E+01
3401.871	9.51E+01	9.30E+01	9.59E+01
3402.353	9.51E+01	9.30E+01	9.59E+01
3402.835	9.51E+01	9.31E+01	9.58E+01
3403.318	9.51E+01	9.31E+01	9.58E+01
3403.8	9.50E+01	9.31E+01	9.58E+01
3404.282	9.50E+01	9.31E+01	9.58E+01
3404.764	9.50E+01	9.31E+01	9.58E+01
3405.246	9.50E+01	9.31E+01	9.58E+01
3405.728	9.50E+01	9.31E+01	9.58E+01
3406.21	9.50E+01	9.31E+01	9.58E+01
3406.692	9.50E+01	9.31E+01	9.59E+01
3407.175	9.50E+01	9.31E+01	9.58E+01
3407.657	9.49E+01	9.30E+01	9.58E+01
3408.139	9.49E+01	9.30E+01	9.58E+01
3408.621	9.49E+01	9.30E+01	9.58E+01
3409.103	9.49E+01	9.30E+01	9.58E+01
3409.585	9.50E+01	9.30E+01	9.58E+01
3410.067	9.50E+01	9.31E+01	9.58E+01
3410.55	9.51E+01	9.31E+01	9.59E+01
3411.031	9.51E+01	9.31E+01	9.59E+01
3411.514	9.52E+01	9.31E+01	9.59E+01
3411.996	9.52E+01	9.31E+01	9.60E+01
3412.478	9.52E+01	9.32E+01	9.60E+01

3412.96	9.52E+01	9.32E+01	9.60E+01
3413.442	9.52E+01	9.31E+01	9.60E+01
3413.924	9.52E+01	9.31E+01	9.60E+01
3414.406	9.51E+01	9.31E+01	9.59E+01
3414.889	9.51E+01	9.31E+01	9.58E+01
3415.371	9.50E+01	9.30E+01	9.58E+01
3415.853	9.50E+01	9.30E+01	9.57E+01
3416.335	9.49E+01	9.30E+01	9.57E+01
3416.817	9.49E+01	9.30E+01	9.56E+01
3417.299	9.48E+01	9.29E+01	9.56E+01
3417.781	9.48E+01	9.29E+01	9.56E+01
3418.263	9.47E+01	9.29E+01	9.56E+01
3418.746	9.47E+01	9.29E+01	9.57E+01
3419.228	9.47E+01	9.29E+01	9.57E+01
3419.71	9.46E+01	9.30E+01	9.57E+01
3420.192	9.46E+01	9.30E+01	9.57E+01
3420.674	9.47E+01	9.31E+01	9.58E+01
3421.156	9.47E+01	9.31E+01	9.58E+01
3421.638	9.48E+01	9.32E+01	9.58E+01
3422.12	9.48E+01	9.32E+01	9.58E+01
3422.603	9.49E+01	9.32E+01	9.58E+01
3423.085	9.49E+01	9.31E+01	9.58E+01
3423.567	9.49E+01	9.31E+01	9.58E+01
3424.049	9.49E+01	9.30E+01	9.58E+01
3424.531	9.49E+01	9.30E+01	9.58E+01
3425.013	9.49E+01	9.30E+01	9.58E+01
3425.495	9.49E+01	9.30E+01	9.58E+01
3425.978	9.49E+01	9.30E+01	9.58E+01
3426.459	9.49E+01	9.30E+01	9.59E+01
3426.942	9.49E+01	9.30E+01	9.59E+01
3427.424	9.49E+01	9.31E+01	9.59E+01
3427.906	9.49E+01	9.31E+01	9.59E+01
3428.388	9.49E+01	9.31E+01	9.59E+01
3428.87	9.49E+01	9.31E+01	9.59E+01
3429.352	9.49E+01	9.32E+01	9.59E+01
3429.834	9.49E+01	9.32E+01	9.59E+01
3430.317	9.48E+01	9.32E+01	9.59E+01
3430.799	9.48E+01	9.32E+01	9.59E+01
3431.281	9.48E+01	9.32E+01	9.59E+01
3431.763	9.49E+01	9.32E+01	9.58E+01
3432.245	9.49E+01	9.31E+01	9.58E+01
3432.727	9.49E+01	9.31E+01	9.58E+01
3433.209	9.49E+01	9.31E+01	9.58E+01
3433.691	9.50E+01	9.31E+01	9.57E+01
3434.174	9.50E+01	9.30E+01	9.57E+01
3434.656	9.50E+01	9.30E+01	9.57E+01
3435.138	9.50E+01	9.30E+01	9.57E+01

3435.62	9.50E+01	9.31E+01	9.58E+01
3436.102	9.50E+01	9.31E+01	9.58E+01
3436.584	9.50E+01	9.31E+01	9.58E+01
3437.066	9.50E+01	9.32E+01	9.59E+01
3437.548	9.50E+01	9.32E+01	9.59E+01
3438.031	9.50E+01	9.32E+01	9.60E+01
3438.513	9.49E+01	9.32E+01	9.60E+01
3438.995	9.49E+01	9.32E+01	9.60E+01
3439.477	9.49E+01	9.32E+01	9.60E+01
3439.959	9.48E+01	9.32E+01	9.60E+01
3440.441	9.48E+01	9.31E+01	9.59E+01
3440.923	9.47E+01	9.31E+01	9.59E+01
3441.406	9.47E+01	9.30E+01	9.58E+01
3441.887	9.47E+01	9.30E+01	9.58E+01
3442.37	9.46E+01	9.30E+01	9.58E+01
3442.852	9.46E+01	9.30E+01	9.58E+01
3443.334	9.46E+01	9.30E+01	9.57E+01
3443.816	9.46E+01	9.31E+01	9.57E+01
3444.298	9.46E+01	9.31E+01	9.58E+01
3444.78	9.46E+01	9.31E+01	9.57E+01
3445.262	9.45E+01	9.31E+01	9.57E+01
3445.745	9.45E+01	9.30E+01	9.57E+01
3446.227	9.44E+01	9.30E+01	9.57E+01
3446.709	9.44E+01	9.30E+01	9.57E+01
3447.191	9.44E+01	9.29E+01	9.57E+01
3447.673	9.44E+01	9.29E+01	9.57E+01
3448.155	9.45E+01	9.30E+01	9.57E+01
3448.637	9.46E+01	9.30E+01	9.57E+01
3449.119	9.47E+01	9.31E+01	9.57E+01
3449.602	9.48E+01	9.31E+01	9.58E+01
3450.084	9.49E+01	9.31E+01	9.58E+01
3450.566	9.49E+01	9.32E+01	9.58E+01
3451.048	9.50E+01	9.32E+01	9.58E+01
3451.53	9.50E+01	9.32E+01	9.57E+01
3452.012	9.50E+01	9.32E+01	9.57E+01
3452.494	9.50E+01	9.32E+01	9.57E+01
3452.976	9.50E+01	9.32E+01	9.57E+01
3453.458	9.50E+01	9.32E+01	9.57E+01
3453.941	9.49E+01	9.32E+01	9.58E+01
3454.423	9.49E+01	9.32E+01	9.58E+01
3454.905	9.48E+01	9.32E+01	9.58E+01
3455.387	9.48E+01	9.32E+01	9.58E+01
3455.869	9.48E+01	9.32E+01	9.59E+01
3456.351	9.48E+01	9.32E+01	9.59E+01
3456.833	9.48E+01	9.32E+01	9.59E+01
3457.315	9.49E+01	9.32E+01	9.59E+01
3457.798	9.49E+01	9.33E+01	9.59E+01

3458.28	9.48E+01	9.33E+01	9.59E+01
3458.762	9.48E+01	9.32E+01	9.59E+01
3459.244	9.48E+01	9.32E+01	9.59E+01
3459.726	9.47E+01	9.32E+01	9.58E+01
3460.208	9.46E+01	9.32E+01	9.58E+01
3460.69	9.46E+01	9.32E+01	9.59E+01
3461.173	9.46E+01	9.32E+01	9.59E+01
3461.655	9.46E+01	9.32E+01	9.59E+01
3462.137	9.46E+01	9.32E+01	9.60E+01
3462.619	9.47E+01	9.32E+01	9.60E+01
3463.101	9.47E+01	9.32E+01	9.60E+01
3463.583	9.48E+01	9.32E+01	9.60E+01
3464.065	9.48E+01	9.32E+01	9.60E+01
3464.547	9.48E+01	9.31E+01	9.60E+01
3465.03	9.48E+01	9.31E+01	9.59E+01
3465.512	9.48E+01	9.30E+01	9.59E+01
3465.994	9.47E+01	9.30E+01	9.58E+01
3466.476	9.47E+01	9.30E+01	9.58E+01
3466.958	9.47E+01	9.30E+01	9.58E+01
3467.44	9.47E+01	9.31E+01	9.58E+01
3467.922	9.48E+01	9.31E+01	9.58E+01
3468.404	9.48E+01	9.32E+01	9.58E+01
3468.886	9.48E+01	9.32E+01	9.58E+01
3469.369	9.48E+01	9.32E+01	9.58E+01
3469.851	9.49E+01	9.33E+01	9.58E+01
3470.333	9.49E+01	9.33E+01	9.58E+01
3470.815	9.49E+01	9.33E+01	9.58E+01
3471.297	9.49E+01	9.33E+01	9.58E+01
3471.779	9.49E+01	9.33E+01	9.58E+01
3472.261	9.49E+01	9.32E+01	9.58E+01
3472.743	9.49E+01	9.32E+01	9.58E+01
3473.226	9.49E+01	9.31E+01	9.58E+01
3473.708	9.49E+01	9.31E+01	9.58E+01
3474.19	9.49E+01	9.30E+01	9.58E+01
3474.672	9.49E+01	9.30E+01	9.58E+01
3475.154	9.49E+01	9.30E+01	9.58E+01
3475.636	9.49E+01	9.30E+01	9.58E+01
3476.118	9.50E+01	9.30E+01	9.59E+01
3476.601	9.50E+01	9.31E+01	9.59E+01
3477.083	9.50E+01	9.31E+01	9.59E+01
3477.565	9.49E+01	9.32E+01	9.59E+01
3478.047	9.49E+01	9.32E+01	9.59E+01
3478.529	9.48E+01	9.33E+01	9.59E+01
3479.011	9.48E+01	9.33E+01	9.59E+01
3479.493	9.47E+01	9.33E+01	9.59E+01
3479.975	9.46E+01	9.32E+01	9.58E+01
3480.458	9.45E+01	9.32E+01	9.58E+01

3480.94	9.45E+01	9.31E+01	9.58E+01
3481.422	9.44E+01	9.31E+01	9.57E+01
3481.904	9.44E+01	9.31E+01	9.57E+01
3482.386	9.44E+01	9.31E+01	9.57E+01
3482.868	9.45E+01	9.31E+01	9.57E+01
3483.35	9.46E+01	9.31E+01	9.57E+01
3483.832	9.46E+01	9.31E+01	9.58E+01
3484.314	9.47E+01	9.31E+01	9.58E+01
3484.797	9.48E+01	9.32E+01	9.58E+01
3485.279	9.48E+01	9.32E+01	9.58E+01
3485.761	9.48E+01	9.32E+01	9.58E+01
3486.243	9.48E+01	9.32E+01	9.58E+01
3486.725	9.47E+01	9.32E+01	9.58E+01
3487.207	9.47E+01	9.32E+01	9.58E+01
3487.689	9.46E+01	9.32E+01	9.58E+01
3488.171	9.46E+01	9.32E+01	9.58E+01
3488.654	9.45E+01	9.32E+01	9.58E+01
3489.136	9.45E+01	9.31E+01	9.57E+01
3489.618	9.45E+01	9.31E+01	9.57E+01
3490.1	9.45E+01	9.31E+01	9.57E+01
3490.582	9.45E+01	9.31E+01	9.57E+01
3491.064	9.45E+01	9.31E+01	9.57E+01
3491.546	9.45E+01	9.31E+01	9.56E+01
3492.029	9.45E+01	9.31E+01	9.56E+01
3492.511	9.45E+01	9.31E+01	9.56E+01
3492.993	9.45E+01	9.30E+01	9.55E+01
3493.475	9.44E+01	9.30E+01	9.55E+01
3493.957	9.44E+01	9.30E+01	9.55E+01
3494.439	9.44E+01	9.30E+01	9.55E+01
3494.921	9.44E+01	9.30E+01	9.55E+01
3495.403	9.44E+01	9.31E+01	9.55E+01
3495.885	9.45E+01	9.31E+01	9.56E+01
3496.368	9.45E+01	9.31E+01	9.56E+01
3496.85	9.45E+01	9.32E+01	9.57E+01
3497.332	9.46E+01	9.32E+01	9.57E+01
3497.814	9.46E+01	9.32E+01	9.58E+01
3498.296	9.47E+01	9.32E+01	9.58E+01
3498.778	9.46E+01	9.32E+01	9.58E+01
3499.26	9.46E+01	9.32E+01	9.58E+01
3499.742	9.45E+01	9.32E+01	9.57E+01
3500.225	9.44E+01	9.32E+01	9.57E+01
3500.707	9.43E+01	9.31E+01	9.57E+01
3501.189	9.42E+01	9.31E+01	9.56E+01
3501.671	9.41E+01	9.31E+01	9.56E+01
3502.153	9.40E+01	9.30E+01	9.55E+01
3502.635	9.40E+01	9.30E+01	9.55E+01
3503.117	9.40E+01	9.30E+01	9.55E+01

3503.6	9.40E+01	9.30E+01	9.55E+01
3504.082	9.41E+01	9.30E+01	9.55E+01
3504.564	9.42E+01	9.30E+01	9.55E+01
3505.046	9.42E+01	9.30E+01	9.55E+01
3505.528	9.43E+01	9.31E+01	9.56E+01
3506.01	9.44E+01	9.31E+01	9.57E+01
3506.492	9.44E+01	9.32E+01	9.57E+01
3506.974	9.45E+01	9.32E+01	9.58E+01
3507.457	9.45E+01	9.32E+01	9.58E+01
3507.939	9.44E+01	9.32E+01	9.59E+01
3508.421	9.44E+01	9.32E+01	9.59E+01
3508.903	9.44E+01	9.33E+01	9.59E+01
3509.385	9.45E+01	9.33E+01	9.59E+01
3509.867	9.45E+01	9.33E+01	9.59E+01
3510.349	9.45E+01	9.33E+01	9.60E+01
3510.831	9.46E+01	9.34E+01	9.60E+01
3511.313	9.46E+01	9.34E+01	9.60E+01
3511.796	9.47E+01	9.34E+01	9.60E+01
3512.278	9.47E+01	9.34E+01	9.60E+01
3512.76	9.47E+01	9.34E+01	9.59E+01
3513.242	9.48E+01	9.34E+01	9.59E+01
3513.724	9.48E+01	9.34E+01	9.59E+01
3514.206	9.48E+01	9.34E+01	9.59E+01
3514.688	9.48E+01	9.34E+01	9.59E+01
3515.17	9.48E+01	9.35E+01	9.60E+01
3515.653	9.48E+01	9.35E+01	9.60E+01
3516.135	9.48E+01	9.35E+01	9.60E+01
3516.617	9.48E+01	9.35E+01	9.60E+01
3517.099	9.48E+01	9.35E+01	9.60E+01
3517.581	9.48E+01	9.35E+01	9.59E+01
3518.063	9.47E+01	9.35E+01	9.59E+01
3518.545	9.47E+01	9.35E+01	9.59E+01
3519.028	9.46E+01	9.35E+01	9.59E+01
3519.51	9.45E+01	9.34E+01	9.59E+01
3519.992	9.44E+01	9.34E+01	9.58E+01
3520.474	9.43E+01	9.33E+01	9.58E+01
3520.956	9.42E+01	9.33E+01	9.57E+01
3521.438	9.41E+01	9.32E+01	9.57E+01
3521.92	9.40E+01	9.32E+01	9.56E+01
3522.402	9.39E+01	9.31E+01	9.56E+01
3522.885	9.39E+01	9.31E+01	9.56E+01
3523.367	9.39E+01	9.31E+01	9.56E+01
3523.849	9.39E+01	9.31E+01	9.55E+01
3524.331	9.39E+01	9.32E+01	9.55E+01
3524.813	9.39E+01	9.32E+01	9.55E+01
3525.295	9.39E+01	9.32E+01	9.54E+01
3525.777	9.39E+01	9.32E+01	9.54E+01

3526.259	9.38E+01	9.33E+01	9.54E+01
3526.741	9.38E+01	9.33E+01	9.54E+01
3527.224	9.38E+01	9.33E+01	9.55E+01
3527.706	9.39E+01	9.33E+01	9.55E+01
3528.188	9.39E+01	9.33E+01	9.56E+01
3528.67	9.40E+01	9.33E+01	9.57E+01
3529.152	9.41E+01	9.33E+01	9.57E+01
3529.634	9.42E+01	9.33E+01	9.58E+01
3530.116	9.43E+01	9.33E+01	9.58E+01
3530.598	9.44E+01	9.33E+01	9.58E+01
3531.081	9.45E+01	9.33E+01	9.58E+01
3531.563	9.45E+01	9.32E+01	9.58E+01
3532.045	9.45E+01	9.32E+01	9.57E+01
3532.527	9.46E+01	9.33E+01	9.57E+01
3533.009	9.45E+01	9.33E+01	9.57E+01
3533.491	9.45E+01	9.33E+01	9.57E+01
3533.973	9.45E+01	9.34E+01	9.57E+01
3534.456	9.44E+01	9.34E+01	9.57E+01
3534.938	9.43E+01	9.35E+01	9.57E+01
3535.42	9.42E+01	9.35E+01	9.56E+01
3535.902	9.42E+01	9.34E+01	9.56E+01
3536.384	9.41E+01	9.34E+01	9.56E+01
3536.866	9.41E+01	9.34E+01	9.56E+01
3537.348	9.42E+01	9.33E+01	9.56E+01
3537.83	9.43E+01	9.33E+01	9.56E+01
3538.313	9.44E+01	9.33E+01	9.56E+01
3538.795	9.45E+01	9.33E+01	9.56E+01
3539.277	9.45E+01	9.33E+01	9.57E+01
3539.759	9.46E+01	9.33E+01	9.57E+01
3540.241	9.46E+01	9.33E+01	9.57E+01
3540.723	9.45E+01	9.34E+01	9.58E+01
3541.205	9.45E+01	9.34E+01	9.58E+01
3541.687	9.44E+01	9.34E+01	9.57E+01
3542.169	9.43E+01	9.34E+01	9.57E+01
3542.652	9.41E+01	9.34E+01	9.57E+01
3543.134	9.40E+01	9.34E+01	9.56E+01
3543.616	9.38E+01	9.33E+01	9.56E+01
3544.098	9.37E+01	9.33E+01	9.55E+01
3544.58	9.36E+01	9.33E+01	9.55E+01
3545.062	9.35E+01	9.33E+01	9.54E+01
3545.544	9.35E+01	9.33E+01	9.54E+01
3546.026	9.36E+01	9.33E+01	9.55E+01
3546.509	9.37E+01	9.34E+01	9.56E+01
3546.991	9.38E+01	9.35E+01	9.57E+01
3547.473	9.40E+01	9.35E+01	9.58E+01
3547.955	9.42E+01	9.36E+01	9.59E+01
3548.437	9.43E+01	9.37E+01	9.59E+01

3548.919	9.44E+01	9.37E+01	9.59E+01
3549.401	9.44E+01	9.37E+01	9.59E+01
3549.884	9.44E+01	9.36E+01	9.59E+01
3550.365	9.43E+01	9.36E+01	9.59E+01
3550.848	9.42E+01	9.36E+01	9.58E+01
3551.33	9.41E+01	9.35E+01	9.58E+01
3551.812	9.40E+01	9.35E+01	9.58E+01
3552.294	9.40E+01	9.35E+01	9.58E+01
3552.776	9.40E+01	9.34E+01	9.58E+01
3553.258	9.41E+01	9.35E+01	9.58E+01
3553.74	9.42E+01	9.35E+01	9.58E+01
3554.223	9.43E+01	9.35E+01	9.59E+01
3554.705	9.44E+01	9.35E+01	9.59E+01
3555.187	9.45E+01	9.36E+01	9.59E+01
3555.669	9.46E+01	9.36E+01	9.59E+01
3556.151	9.46E+01	9.36E+01	9.59E+01
3556.633	9.46E+01	9.35E+01	9.58E+01
3557.115	9.46E+01	9.35E+01	9.57E+01
3557.597	9.45E+01	9.34E+01	9.56E+01
3558.08	9.44E+01	9.33E+01	9.56E+01
3558.562	9.43E+01	9.33E+01	9.55E+01
3559.044	9.42E+01	9.32E+01	9.54E+01
3559.526	9.41E+01	9.32E+01	9.54E+01
3560.008	9.40E+01	9.32E+01	9.54E+01
3560.49	9.39E+01	9.32E+01	9.54E+01
3560.972	9.39E+01	9.32E+01	9.54E+01
3561.454	9.38E+01	9.32E+01	9.55E+01
3561.937	9.38E+01	9.33E+01	9.55E+01
3562.419	9.38E+01	9.33E+01	9.56E+01
3562.901	9.37E+01	9.33E+01	9.56E+01
3563.383	9.36E+01	9.33E+01	9.56E+01
3563.865	9.34E+01	9.33E+01	9.55E+01
3564.347	9.33E+01	9.33E+01	9.54E+01
3564.829	9.30E+01	9.32E+01	9.53E+01
3565.312	9.28E+01	9.31E+01	9.51E+01
3565.793	9.25E+01	9.30E+01	9.50E+01
3566.276	9.24E+01	9.29E+01	9.49E+01
3566.758	9.24E+01	9.29E+01	9.49E+01
3567.24	9.26E+01	9.30E+01	9.50E+01
3567.722	9.30E+01	9.31E+01	9.51E+01
3568.204	9.34E+01	9.33E+01	9.53E+01
3568.686	9.38E+01	9.35E+01	9.55E+01
3569.168	9.41E+01	9.37E+01	9.57E+01
3569.651	9.43E+01	9.38E+01	9.58E+01
3570.133	9.45E+01	9.38E+01	9.59E+01
3570.615	9.45E+01	9.39E+01	9.60E+01
3571.097	9.46E+01	9.39E+01	9.60E+01

3571.579	9.46E+01	9.39E+01	9.60E+01
3572.061	9.46E+01	9.38E+01	9.60E+01
3572.543	9.46E+01	9.38E+01	9.59E+01
3573.025	9.46E+01	9.38E+01	9.59E+01
3573.508	9.46E+01	9.38E+01	9.59E+01
3573.99	9.45E+01	9.38E+01	9.59E+01
3574.472	9.45E+01	9.38E+01	9.59E+01
3574.954	9.45E+01	9.38E+01	9.59E+01
3575.436	9.45E+01	9.38E+01	9.59E+01
3575.918	9.45E+01	9.37E+01	9.59E+01
3576.4	9.45E+01	9.37E+01	9.59E+01
3576.882	9.45E+01	9.37E+01	9.59E+01
3577.365	9.45E+01	9.37E+01	9.59E+01
3577.847	9.45E+01	9.37E+01	9.59E+01
3578.329	9.45E+01	9.38E+01	9.59E+01
3578.811	9.45E+01	9.38E+01	9.59E+01
3579.293	9.45E+01	9.38E+01	9.59E+01
3579.775	9.45E+01	9.38E+01	9.59E+01
3580.257	9.45E+01	9.38E+01	9.59E+01
3580.74	9.45E+01	9.38E+01	9.59E+01
3581.221	9.44E+01	9.37E+01	9.58E+01
3581.704	9.44E+01	9.37E+01	9.58E+01
3582.186	9.43E+01	9.37E+01	9.58E+01
3582.668	9.42E+01	9.36E+01	9.58E+01
3583.15	9.42E+01	9.36E+01	9.58E+01
3583.632	9.41E+01	9.36E+01	9.58E+01
3584.114	9.41E+01	9.36E+01	9.58E+01
3584.596	9.40E+01	9.36E+01	9.58E+01
3585.079	9.38E+01	9.36E+01	9.58E+01
3585.561	9.37E+01	9.36E+01	9.58E+01
3586.043	9.35E+01	9.35E+01	9.57E+01
3586.525	9.33E+01	9.35E+01	9.56E+01
3587.007	9.31E+01	9.34E+01	9.56E+01
3587.489	9.30E+01	9.34E+01	9.55E+01
3587.971	9.31E+01	9.33E+01	9.55E+01
3588.453	9.33E+01	9.33E+01	9.55E+01
3588.936	9.35E+01	9.34E+01	9.55E+01
3589.418	9.37E+01	9.35E+01	9.56E+01
3589.9	9.39E+01	9.35E+01	9.56E+01
3590.382	9.40E+01	9.36E+01	9.57E+01
3590.864	9.41E+01	9.37E+01	9.57E+01
3591.346	9.41E+01	9.37E+01	9.57E+01
3591.828	9.41E+01	9.38E+01	9.57E+01
3592.31	9.40E+01	9.38E+01	9.56E+01
3592.792	9.40E+01	9.38E+01	9.56E+01
3593.275	9.39E+01	9.39E+01	9.56E+01
3593.757	9.38E+01	9.39E+01	9.56E+01

3594.239	9.38E+01	9.39E+01	9.56E+01
3594.721	9.38E+01	9.39E+01	9.56E+01
3595.203	9.38E+01	9.39E+01	9.57E+01
3595.685	9.38E+01	9.39E+01	9.57E+01
3596.167	9.39E+01	9.39E+01	9.57E+01
3596.65	9.39E+01	9.39E+01	9.58E+01
3597.132	9.38E+01	9.38E+01	9.58E+01
3597.614	9.38E+01	9.38E+01	9.57E+01
3598.096	9.37E+01	9.37E+01	9.57E+01
3598.578	9.37E+01	9.37E+01	9.57E+01
3599.06	9.36E+01	9.37E+01	9.56E+01
3599.542	9.36E+01	9.36E+01	9.55E+01
3600.024	9.36E+01	9.36E+01	9.55E+01
3600.507	9.36E+01	9.37E+01	9.55E+01
3600.989	9.37E+01	9.37E+01	9.55E+01
3601.471	9.39E+01	9.38E+01	9.56E+01
3601.953	9.40E+01	9.39E+01	9.56E+01
3602.435	9.42E+01	9.40E+01	9.57E+01
3602.917	9.43E+01	9.41E+01	9.58E+01
3603.399	9.44E+01	9.41E+01	9.58E+01
3603.881	9.44E+01	9.42E+01	9.59E+01
3604.364	9.43E+01	9.41E+01	9.58E+01
3604.846	9.42E+01	9.41E+01	9.58E+01
3605.328	9.41E+01	9.40E+01	9.57E+01
3605.81	9.38E+01	9.39E+01	9.56E+01
3606.292	9.36E+01	9.38E+01	9.54E+01
3606.774	9.34E+01	9.37E+01	9.53E+01
3607.256	9.33E+01	9.36E+01	9.52E+01
3607.738	9.32E+01	9.36E+01	9.52E+01
3608.22	9.32E+01	9.36E+01	9.52E+01
3608.703	9.32E+01	9.37E+01	9.52E+01
3609.185	9.33E+01	9.37E+01	9.53E+01
3609.667	9.34E+01	9.38E+01	9.53E+01
3610.149	9.34E+01	9.38E+01	9.53E+01
3610.631	9.34E+01	9.38E+01	9.53E+01
3611.113	9.33E+01	9.37E+01	9.52E+01
3611.595	9.32E+01	9.37E+01	9.51E+01
3612.078	9.31E+01	9.36E+01	9.51E+01
3612.56	9.30E+01	9.35E+01	9.50E+01
3613.042	9.29E+01	9.34E+01	9.50E+01
3613.524	9.29E+01	9.34E+01	9.50E+01
3614.006	9.29E+01	9.34E+01	9.51E+01
3614.488	9.30E+01	9.34E+01	9.51E+01
3614.97	9.30E+01	9.35E+01	9.52E+01
3615.452	9.31E+01	9.35E+01	9.53E+01
3615.935	9.31E+01	9.35E+01	9.53E+01
3616.417	9.30E+01	9.35E+01	9.53E+01

3616.899	9.30E+01	9.35E+01	9.52E+01
3617.381	9.28E+01	9.34E+01	9.52E+01
3617.863	9.27E+01	9.34E+01	9.52E+01
3618.345	9.27E+01	9.34E+01	9.51E+01
3618.827	9.26E+01	9.34E+01	9.51E+01
3619.309	9.27E+01	9.34E+01	9.51E+01
3619.792	9.28E+01	9.34E+01	9.51E+01
3620.274	9.31E+01	9.35E+01	9.52E+01
3620.756	9.33E+01	9.35E+01	9.52E+01
3621.238	9.35E+01	9.36E+01	9.53E+01
3621.72	9.37E+01	9.37E+01	9.54E+01
3622.202	9.39E+01	9.37E+01	9.55E+01
3622.684	9.40E+01	9.38E+01	9.55E+01
3623.167	9.41E+01	9.38E+01	9.56E+01
3623.648	9.41E+01	9.38E+01	9.56E+01
3624.131	9.40E+01	9.38E+01	9.56E+01
3624.613	9.39E+01	9.38E+01	9.56E+01
3625.095	9.38E+01	9.38E+01	9.56E+01
3625.577	9.36E+01	9.38E+01	9.55E+01
3626.059	9.34E+01	9.38E+01	9.55E+01
3626.541	9.31E+01	9.38E+01	9.55E+01
3627.023	9.28E+01	9.37E+01	9.54E+01
3627.506	9.25E+01	9.37E+01	9.54E+01
3627.988	9.23E+01	9.36E+01	9.54E+01
3628.47	9.23E+01	9.36E+01	9.55E+01
3628.952	9.24E+01	9.37E+01	9.56E+01
3629.434	9.27E+01	9.37E+01	9.57E+01
3629.916	9.31E+01	9.38E+01	9.58E+01
3630.398	9.34E+01	9.39E+01	9.59E+01
3630.88	9.36E+01	9.40E+01	9.59E+01
3631.363	9.38E+01	9.40E+01	9.59E+01
3631.845	9.38E+01	9.41E+01	9.59E+01
3632.327	9.38E+01	9.41E+01	9.58E+01
3632.809	9.38E+01	9.41E+01	9.58E+01
3633.291	9.39E+01	9.40E+01	9.57E+01
3633.773	9.39E+01	9.40E+01	9.57E+01
3634.255	9.39E+01	9.40E+01	9.56E+01
3634.737	9.40E+01	9.40E+01	9.57E+01
3635.219	9.41E+01	9.39E+01	9.57E+01
3635.702	9.42E+01	9.39E+01	9.57E+01
3636.184	9.42E+01	9.39E+01	9.58E+01
3636.666	9.43E+01	9.39E+01	9.58E+01
3637.148	9.43E+01	9.39E+01	9.59E+01
3637.63	9.43E+01	9.39E+01	9.59E+01
3638.112	9.43E+01	9.39E+01	9.59E+01
3638.594	9.43E+01	9.39E+01	9.59E+01
3639.076	9.43E+01	9.39E+01	9.59E+01

3639.559	9.42E+01	9.39E+01	9.59E+01
3640.041	9.42E+01	9.39E+01	9.59E+01
3640.523	9.42E+01	9.39E+01	9.58E+01
3641.005	9.41E+01	9.40E+01	9.58E+01
3641.487	9.41E+01	9.40E+01	9.57E+01
3641.969	9.41E+01	9.40E+01	9.57E+01
3642.451	9.41E+01	9.40E+01	9.57E+01
3642.934	9.41E+01	9.40E+01	9.57E+01
3643.416	9.40E+01	9.40E+01	9.56E+01
3643.898	9.39E+01	9.40E+01	9.56E+01
3644.38	9.37E+01	9.39E+01	9.56E+01
3644.862	9.35E+01	9.38E+01	9.56E+01
3645.344	9.32E+01	9.37E+01	9.55E+01
3645.826	9.28E+01	9.36E+01	9.55E+01
3646.308	9.24E+01	9.35E+01	9.54E+01
3646.791	9.20E+01	9.34E+01	9.53E+01
3647.273	9.16E+01	9.34E+01	9.52E+01
3647.755	9.13E+01	9.33E+01	9.51E+01
3648.237	9.10E+01	9.33E+01	9.50E+01
3648.719	9.09E+01	9.33E+01	9.49E+01
3649.201	9.10E+01	9.34E+01	9.49E+01
3649.683	9.12E+01	9.35E+01	9.49E+01
3650.165	9.16E+01	9.36E+01	9.51E+01
3650.647	9.21E+01	9.37E+01	9.53E+01
3651.13	9.26E+01	9.38E+01	9.55E+01
3651.612	9.31E+01	9.38E+01	9.56E+01
3652.094	9.35E+01	9.39E+01	9.57E+01
3652.576	9.37E+01	9.39E+01	9.58E+01
3653.058	9.39E+01	9.39E+01	9.58E+01
3653.54	9.40E+01	9.40E+01	9.58E+01
3654.022	9.40E+01	9.40E+01	9.57E+01
3654.504	9.39E+01	9.40E+01	9.56E+01
3654.987	9.38E+01	9.41E+01	9.56E+01
3655.469	9.37E+01	9.42E+01	9.55E+01
3655.951	9.37E+01	9.42E+01	9.56E+01
3656.433	9.38E+01	9.43E+01	9.57E+01
3656.915	9.39E+01	9.44E+01	9.58E+01
3657.397	9.41E+01	9.44E+01	9.59E+01
3657.879	9.43E+01	9.45E+01	9.60E+01
3658.362	9.44E+01	9.45E+01	9.61E+01
3658.844	9.45E+01	9.44E+01	9.62E+01
3659.326	9.46E+01	9.44E+01	9.62E+01
3659.808	9.46E+01	9.44E+01	9.62E+01
3660.29	9.46E+01	9.43E+01	9.62E+01
3660.772	9.46E+01	9.43E+01	9.62E+01
3661.254	9.46E+01	9.43E+01	9.62E+01
3661.736	9.46E+01	9.43E+01	9.62E+01

3662.219	9.47E+01	9.43E+01	9.62E+01
3662.701	9.47E+01	9.43E+01	9.62E+01
3663.183	9.47E+01	9.44E+01	9.62E+01
3663.665	9.47E+01	9.44E+01	9.62E+01
3664.147	9.47E+01	9.44E+01	9.62E+01
3664.629	9.47E+01	9.44E+01	9.62E+01
3665.111	9.47E+01	9.45E+01	9.62E+01
3665.593	9.46E+01	9.45E+01	9.62E+01
3666.075	9.45E+01	9.45E+01	9.62E+01
3666.558	9.44E+01	9.45E+01	9.61E+01
3667.04	9.42E+01	9.45E+01	9.61E+01
3667.522	9.39E+01	9.45E+01	9.59E+01
3668.004	9.36E+01	9.44E+01	9.58E+01
3668.486	9.33E+01	9.43E+01	9.56E+01
3668.968	9.29E+01	9.42E+01	9.54E+01
3669.45	9.27E+01	9.42E+01	9.53E+01
3669.932	9.27E+01	9.42E+01	9.53E+01
3670.415	9.28E+01	9.42E+01	9.53E+01
3670.897	9.30E+01	9.43E+01	9.54E+01
3671.379	9.32E+01	9.44E+01	9.55E+01
3671.861	9.34E+01	9.45E+01	9.56E+01
3672.343	9.35E+01	9.46E+01	9.56E+01
3672.825	9.35E+01	9.46E+01	9.56E+01
3673.307	9.34E+01	9.46E+01	9.56E+01
3673.79	9.32E+01	9.46E+01	9.55E+01
3674.271	9.29E+01	9.45E+01	9.54E+01
3674.754	9.26E+01	9.44E+01	9.53E+01
3675.236	9.24E+01	9.42E+01	9.52E+01
3675.718	9.24E+01	9.41E+01	9.52E+01
3676.2	9.26E+01	9.40E+01	9.53E+01
3676.682	9.29E+01	9.39E+01	9.53E+01
3677.164	9.31E+01	9.39E+01	9.53E+01
3677.646	9.33E+01	9.39E+01	9.54E+01
3678.129	9.35E+01	9.40E+01	9.54E+01
3678.611	9.37E+01	9.41E+01	9.54E+01
3679.093	9.39E+01	9.42E+01	9.55E+01
3679.575	9.40E+01	9.43E+01	9.55E+01
3680.057	9.42E+01	9.44E+01	9.56E+01
3680.539	9.44E+01	9.45E+01	9.57E+01
3681.021	9.46E+01	9.46E+01	9.58E+01
3681.503	9.47E+01	9.46E+01	9.59E+01
3681.986	9.47E+01	9.46E+01	9.60E+01
3682.468	9.48E+01	9.47E+01	9.60E+01
3682.95	9.47E+01	9.46E+01	9.61E+01
3683.432	9.47E+01	9.46E+01	9.61E+01
3683.914	9.46E+01	9.45E+01	9.60E+01
3684.396	9.44E+01	9.45E+01	9.60E+01

3684.878	9.43E+01	9.44E+01	9.59E+01
3685.36	9.42E+01	9.44E+01	9.59E+01
3685.843	9.40E+01	9.43E+01	9.58E+01
3686.325	9.38E+01	9.42E+01	9.57E+01
3686.807	9.37E+01	9.42E+01	9.56E+01
3687.289	9.34E+01	9.41E+01	9.55E+01
3687.771	9.32E+01	9.40E+01	9.54E+01
3688.253	9.29E+01	9.40E+01	9.53E+01
3688.735	9.27E+01	9.39E+01	9.53E+01
3689.218	9.26E+01	9.39E+01	9.53E+01
3689.699	9.26E+01	9.40E+01	9.53E+01
3690.182	9.27E+01	9.40E+01	9.54E+01
3690.664	9.29E+01	9.41E+01	9.55E+01
3691.146	9.33E+01	9.43E+01	9.57E+01
3691.628	9.37E+01	9.44E+01	9.59E+01
3692.11	9.41E+01	9.45E+01	9.60E+01
3692.592	9.44E+01	9.46E+01	9.62E+01
3693.074	9.47E+01	9.47E+01	9.63E+01
3693.557	9.49E+01	9.47E+01	9.64E+01
3694.039	9.50E+01	9.48E+01	9.64E+01
3694.521	9.51E+01	9.48E+01	9.65E+01
3695.003	9.51E+01	9.48E+01	9.65E+01
3695.485	9.51E+01	9.48E+01	9.64E+01
3695.967	9.50E+01	9.47E+01	9.63E+01
3696.449	9.49E+01	9.47E+01	9.62E+01
3696.931	9.48E+01	9.46E+01	9.61E+01
3697.414	9.48E+01	9.46E+01	9.61E+01
3697.896	9.47E+01	9.45E+01	9.60E+01
3698.378	9.47E+01	9.45E+01	9.59E+01
3698.86	9.46E+01	9.45E+01	9.59E+01
3699.342	9.46E+01	9.45E+01	9.58E+01
3699.824	9.45E+01	9.45E+01	9.58E+01
3700.306	9.44E+01	9.46E+01	9.59E+01
3700.788	9.44E+01	9.46E+01	9.59E+01
3701.271	9.44E+01	9.47E+01	9.59E+01
3701.753	9.44E+01	9.47E+01	9.60E+01
3702.235	9.45E+01	9.48E+01	9.60E+01
3702.717	9.45E+01	9.48E+01	9.61E+01
3703.199	9.46E+01	9.48E+01	9.61E+01
3703.681	9.47E+01	9.48E+01	9.61E+01
3704.163	9.47E+01	9.47E+01	9.61E+01
3704.646	9.47E+01	9.47E+01	9.61E+01
3705.128	9.47E+01	9.47E+01	9.61E+01
3705.61	9.47E+01	9.47E+01	9.61E+01
3706.092	9.47E+01	9.46E+01	9.61E+01
3706.574	9.46E+01	9.46E+01	9.61E+01
3707.056	9.45E+01	9.45E+01	9.61E+01

3707.538	9.43E+01	9.45E+01	9.60E+01
3708.02	9.41E+01	9.44E+01	9.60E+01
3708.502	9.38E+01	9.44E+01	9.58E+01
3708.985	9.34E+01	9.43E+01	9.57E+01
3709.467	9.30E+01	9.42E+01	9.55E+01
3709.949	9.26E+01	9.42E+01	9.53E+01
3710.431	9.23E+01	9.41E+01	9.52E+01
3710.913	9.22E+01	9.42E+01	9.52E+01
3711.395	9.23E+01	9.42E+01	9.52E+01
3711.877	9.26E+01	9.43E+01	9.53E+01
3712.359	9.29E+01	9.43E+01	9.54E+01
3712.842	9.33E+01	9.44E+01	9.55E+01
3713.324	9.36E+01	9.45E+01	9.56E+01
3713.806	9.39E+01	9.46E+01	9.57E+01
3714.288	9.42E+01	9.46E+01	9.58E+01
3714.77	9.44E+01	9.46E+01	9.59E+01
3715.252	9.46E+01	9.46E+01	9.59E+01
3715.734	9.46E+01	9.46E+01	9.59E+01
3716.217	9.47E+01	9.46E+01	9.59E+01
3716.698	9.47E+01	9.46E+01	9.59E+01
3717.181	9.46E+01	9.45E+01	9.59E+01
3717.663	9.45E+01	9.45E+01	9.59E+01
3718.145	9.45E+01	9.44E+01	9.59E+01
3718.627	9.44E+01	9.44E+01	9.59E+01
3719.109	9.44E+01	9.44E+01	9.59E+01
3719.591	9.43E+01	9.44E+01	9.59E+01
3720.073	9.43E+01	9.44E+01	9.60E+01
3720.556	9.42E+01	9.45E+01	9.60E+01
3721.038	9.41E+01	9.45E+01	9.59E+01
3721.52	9.40E+01	9.45E+01	9.59E+01
3722.002	9.39E+01	9.45E+01	9.59E+01
3722.484	9.38E+01	9.45E+01	9.59E+01
3722.966	9.37E+01	9.45E+01	9.58E+01
3723.448	9.37E+01	9.45E+01	9.58E+01
3723.93	9.37E+01	9.45E+01	9.57E+01
3724.413	9.37E+01	9.45E+01	9.57E+01
3724.895	9.37E+01	9.45E+01	9.56E+01
3725.377	9.38E+01	9.45E+01	9.55E+01
3725.859	9.38E+01	9.46E+01	9.55E+01
3726.341	9.40E+01	9.46E+01	9.56E+01
3726.823	9.41E+01	9.46E+01	9.56E+01
3727.305	9.43E+01	9.46E+01	9.57E+01
3727.787	9.44E+01	9.46E+01	9.58E+01
3728.27	9.45E+01	9.46E+01	9.59E+01
3728.752	9.46E+01	9.46E+01	9.60E+01
3729.234	9.46E+01	9.45E+01	9.60E+01
3729.716	9.45E+01	9.45E+01	9.60E+01

3730.198	9.44E+01	9.44E+01	9.59E+01
3730.68	9.42E+01	9.43E+01	9.58E+01
3731.162	9.39E+01	9.42E+01	9.57E+01
3731.645	9.36E+01	9.42E+01	9.56E+01
3732.126	9.33E+01	9.41E+01	9.54E+01
3732.609	9.31E+01	9.40E+01	9.53E+01
3733.091	9.28E+01	9.40E+01	9.52E+01
3733.573	9.26E+01	9.40E+01	9.51E+01
3734.055	9.24E+01	9.40E+01	9.50E+01
3734.537	9.23E+01	9.41E+01	9.49E+01
3735.019	9.22E+01	9.41E+01	9.49E+01
3735.501	9.22E+01	9.41E+01	9.49E+01
3735.984	9.23E+01	9.42E+01	9.50E+01
3736.466	9.24E+01	9.42E+01	9.51E+01
3736.948	9.27E+01	9.42E+01	9.53E+01
3737.43	9.29E+01	9.43E+01	9.54E+01
3737.912	9.31E+01	9.43E+01	9.55E+01
3738.394	9.33E+01	9.43E+01	9.56E+01
3738.876	9.34E+01	9.43E+01	9.57E+01
3739.358	9.34E+01	9.43E+01	9.57E+01
3739.841	9.35E+01	9.43E+01	9.57E+01
3740.323	9.34E+01	9.43E+01	9.57E+01
3740.805	9.34E+01	9.43E+01	9.57E+01
3741.287	9.33E+01	9.42E+01	9.56E+01
3741.769	9.33E+01	9.42E+01	9.56E+01
3742.251	9.32E+01	9.41E+01	9.55E+01
3742.733	9.30E+01	9.41E+01	9.54E+01
3743.215	9.28E+01	9.40E+01	9.52E+01
3743.698	9.26E+01	9.39E+01	9.51E+01
3744.18	9.24E+01	9.39E+01	9.50E+01
3744.662	9.22E+01	9.40E+01	9.50E+01
3745.144	9.22E+01	9.41E+01	9.50E+01
3745.626	9.23E+01	9.42E+01	9.52E+01
3746.108	9.25E+01	9.42E+01	9.53E+01
3746.59	9.26E+01	9.43E+01	9.54E+01
3747.073	9.27E+01	9.43E+01	9.54E+01
3747.554	9.27E+01	9.42E+01	9.54E+01
3748.037	9.27E+01	9.41E+01	9.54E+01
3748.519	9.25E+01	9.40E+01	9.52E+01
3749.001	9.23E+01	9.38E+01	9.51E+01
3749.483	9.20E+01	9.37E+01	9.49E+01
3749.965	9.17E+01	9.36E+01	9.48E+01
3750.447	9.16E+01	9.36E+01	9.47E+01
3750.929	9.16E+01	9.36E+01	9.48E+01
3751.412	9.19E+01	9.38E+01	9.49E+01
3751.894	9.22E+01	9.40E+01	9.51E+01
3752.376	9.26E+01	9.41E+01	9.52E+01

3752.858	9.30E+01	9.43E+01	9.54E+01
3753.34	9.33E+01	9.43E+01	9.55E+01
3753.822	9.35E+01	9.44E+01	9.55E+01
3754.304	9.37E+01	9.44E+01	9.56E+01
3754.786	9.38E+01	9.44E+01	9.56E+01
3755.269	9.38E+01	9.43E+01	9.55E+01
3755.751	9.38E+01	9.43E+01	9.55E+01
3756.233	9.37E+01	9.42E+01	9.55E+01
3756.715	9.36E+01	9.42E+01	9.55E+01
3757.197	9.35E+01	9.42E+01	9.55E+01
3757.679	9.34E+01	9.42E+01	9.56E+01
3758.161	9.34E+01	9.42E+01	9.56E+01
3758.643	9.34E+01	9.42E+01	9.57E+01
3759.125	9.34E+01	9.43E+01	9.58E+01
3759.608	9.36E+01	9.43E+01	9.59E+01
3760.09	9.38E+01	9.44E+01	9.59E+01
3760.572	9.40E+01	9.44E+01	9.60E+01
3761.054	9.42E+01	9.45E+01	9.61E+01
3761.536	9.43E+01	9.45E+01	9.62E+01
3762.018	9.44E+01	9.46E+01	9.62E+01
3762.5	9.45E+01	9.46E+01	9.62E+01
3762.982	9.44E+01	9.46E+01	9.62E+01
3763.465	9.44E+01	9.46E+01	9.62E+01
3763.947	9.42E+01	9.45E+01	9.62E+01
3764.429	9.41E+01	9.45E+01	9.61E+01
3764.911	9.41E+01	9.45E+01	9.61E+01
3765.393	9.40E+01	9.44E+01	9.60E+01
3765.875	9.41E+01	9.44E+01	9.60E+01
3766.357	9.41E+01	9.44E+01	9.60E+01
3766.84	9.42E+01	9.44E+01	9.59E+01
3767.322	9.42E+01	9.44E+01	9.59E+01
3767.804	9.42E+01	9.44E+01	9.58E+01
3768.286	9.41E+01	9.43E+01	9.57E+01
3768.768	9.41E+01	9.43E+01	9.56E+01
3769.25	9.40E+01	9.42E+01	9.56E+01
3769.732	9.41E+01	9.42E+01	9.56E+01
3770.214	9.41E+01	9.42E+01	9.57E+01
3770.697	9.42E+01	9.43E+01	9.58E+01
3771.179	9.44E+01	9.44E+01	9.59E+01
3771.661	9.45E+01	9.45E+01	9.60E+01
3772.143	9.47E+01	9.46E+01	9.61E+01
3772.625	9.48E+01	9.46E+01	9.62E+01
3773.107	9.49E+01	9.47E+01	9.62E+01
3773.589	9.49E+01	9.48E+01	9.62E+01
3774.071	9.50E+01	9.48E+01	9.62E+01
3774.553	9.50E+01	9.48E+01	9.62E+01
3775.036	9.49E+01	9.48E+01	9.62E+01

3775.518	9.49E+01	9.48E+01	9.62E+01
3776	9.48E+01	9.47E+01	9.61E+01
3776.482	9.47E+01	9.47E+01	9.61E+01
3776.964	9.47E+01	9.46E+01	9.61E+01
3777.446	9.46E+01	9.46E+01	9.60E+01
3777.928	9.45E+01	9.46E+01	9.60E+01
3778.41	9.44E+01	9.46E+01	9.60E+01
3778.893	9.44E+01	9.46E+01	9.59E+01
3779.375	9.44E+01	9.46E+01	9.60E+01
3779.857	9.44E+01	9.46E+01	9.60E+01
3780.339	9.45E+01	9.46E+01	9.61E+01
3780.821	9.46E+01	9.46E+01	9.61E+01
3781.303	9.46E+01	9.46E+01	9.61E+01
3781.785	9.47E+01	9.46E+01	9.61E+01
3782.268	9.47E+01	9.46E+01	9.61E+01
3782.75	9.47E+01	9.46E+01	9.61E+01
3783.232	9.46E+01	9.46E+01	9.61E+01
3783.714	9.45E+01	9.47E+01	9.60E+01
3784.196	9.45E+01	9.47E+01	9.60E+01
3784.678	9.45E+01	9.47E+01	9.60E+01
3785.16	9.45E+01	9.47E+01	9.60E+01
3785.642	9.46E+01	9.47E+01	9.60E+01
3786.125	9.47E+01	9.47E+01	9.61E+01
3786.607	9.48E+01	9.48E+01	9.61E+01
3787.089	9.48E+01	9.48E+01	9.62E+01
3787.571	9.49E+01	9.48E+01	9.62E+01
3788.053	9.50E+01	9.47E+01	9.62E+01
3788.535	9.50E+01	9.47E+01	9.63E+01
3789.017	9.51E+01	9.47E+01	9.63E+01
3789.499	9.51E+01	9.46E+01	9.63E+01
3789.981	9.51E+01	9.46E+01	9.64E+01
3790.464	9.52E+01	9.46E+01	9.64E+01
3790.946	9.52E+01	9.46E+01	9.64E+01
3791.428	9.52E+01	9.47E+01	9.65E+01
3791.91	9.52E+01	9.47E+01	9.65E+01
3792.392	9.51E+01	9.47E+01	9.65E+01
3792.874	9.50E+01	9.46E+01	9.64E+01
3793.356	9.49E+01	9.46E+01	9.63E+01
3793.838	9.47E+01	9.45E+01	9.62E+01
3794.321	9.45E+01	9.45E+01	9.61E+01
3794.803	9.43E+01	9.44E+01	9.59E+01
3795.285	9.41E+01	9.44E+01	9.58E+01
3795.767	9.39E+01	9.44E+01	9.56E+01
3796.249	9.38E+01	9.44E+01	9.55E+01
3796.731	9.37E+01	9.44E+01	9.55E+01
3797.213	9.37E+01	9.44E+01	9.55E+01
3797.696	9.37E+01	9.44E+01	9.55E+01

3798.178	9.36E+01	9.44E+01	9.56E+01
3798.66	9.36E+01	9.45E+01	9.56E+01
3799.142	9.35E+01	9.45E+01	9.56E+01
3799.624	9.34E+01	9.45E+01	9.56E+01
3800.106	9.32E+01	9.45E+01	9.56E+01
3800.588	9.31E+01	9.45E+01	9.56E+01
3801.07	9.30E+01	9.45E+01	9.56E+01
3801.552	9.30E+01	9.44E+01	9.56E+01
3802.035	9.32E+01	9.44E+01	9.57E+01
3802.517	9.34E+01	9.44E+01	9.58E+01
3802.999	9.37E+01	9.44E+01	9.58E+01
3803.481	9.38E+01	9.44E+01	9.59E+01
3803.963	9.39E+01	9.44E+01	9.59E+01
3804.445	9.39E+01	9.44E+01	9.59E+01
3804.927	9.38E+01	9.43E+01	9.58E+01
3805.409	9.38E+01	9.43E+01	9.58E+01
3805.892	9.37E+01	9.43E+01	9.57E+01
3806.374	9.36E+01	9.43E+01	9.57E+01
3806.856	9.36E+01	9.43E+01	9.57E+01
3807.338	9.38E+01	9.43E+01	9.57E+01
3807.82	9.40E+01	9.44E+01	9.58E+01
3808.302	9.42E+01	9.44E+01	9.59E+01
3808.784	9.44E+01	9.45E+01	9.60E+01
3809.267	9.46E+01	9.45E+01	9.61E+01
3809.749	9.47E+01	9.46E+01	9.62E+01
3810.231	9.48E+01	9.46E+01	9.62E+01
3810.713	9.49E+01	9.46E+01	9.63E+01
3811.195	9.50E+01	9.47E+01	9.63E+01
3811.677	9.50E+01	9.47E+01	9.63E+01
3812.159	9.49E+01	9.47E+01	9.63E+01
3812.641	9.49E+01	9.48E+01	9.63E+01
3813.124	9.48E+01	9.48E+01	9.62E+01
3813.606	9.47E+01	9.48E+01	9.61E+01
3814.088	9.45E+01	9.48E+01	9.60E+01
3814.57	9.43E+01	9.48E+01	9.59E+01
3815.052	9.41E+01	9.47E+01	9.58E+01
3815.534	9.38E+01	9.46E+01	9.56E+01
3816.016	9.37E+01	9.45E+01	9.55E+01
3816.498	9.36E+01	9.44E+01	9.54E+01
3816.98	9.35E+01	9.43E+01	9.54E+01
3817.463	9.35E+01	9.43E+01	9.54E+01
3817.945	9.34E+01	9.43E+01	9.53E+01
3818.427	9.33E+01	9.43E+01	9.53E+01
3818.909	9.31E+01	9.43E+01	9.52E+01
3819.391	9.28E+01	9.42E+01	9.51E+01
3819.873	9.25E+01	9.42E+01	9.50E+01
3820.355	9.23E+01	9.41E+01	9.49E+01

3820.837	9.22E+01	9.41E+01	9.49E+01
3821.32	9.22E+01	9.41E+01	9.50E+01
3821.802	9.25E+01	9.41E+01	9.51E+01
3822.284	9.27E+01	9.42E+01	9.52E+01
3822.766	9.30E+01	9.43E+01	9.53E+01
3823.248	9.33E+01	9.43E+01	9.54E+01
3823.73	9.34E+01	9.44E+01	9.55E+01
3824.212	9.35E+01	9.45E+01	9.55E+01
3824.695	9.36E+01	9.45E+01	9.56E+01
3825.177	9.36E+01	9.45E+01	9.56E+01
3825.659	9.36E+01	9.45E+01	9.56E+01
3826.141	9.37E+01	9.45E+01	9.57E+01
3826.623	9.37E+01	9.46E+01	9.57E+01
3827.105	9.39E+01	9.46E+01	9.58E+01
3827.587	9.40E+01	9.46E+01	9.58E+01
3828.069	9.41E+01	9.46E+01	9.59E+01
3828.552	9.43E+01	9.46E+01	9.59E+01
3829.034	9.43E+01	9.46E+01	9.60E+01
3829.516	9.43E+01	9.46E+01	9.60E+01
3829.998	9.43E+01	9.47E+01	9.59E+01
3830.48	9.42E+01	9.47E+01	9.59E+01
3830.962	9.41E+01	9.46E+01	9.58E+01
3831.444	9.40E+01	9.46E+01	9.58E+01
3831.926	9.39E+01	9.46E+01	9.57E+01
3832.408	9.37E+01	9.45E+01	9.57E+01
3832.891	9.36E+01	9.44E+01	9.56E+01
3833.373	9.35E+01	9.44E+01	9.56E+01
3833.855	9.33E+01	9.43E+01	9.55E+01
3834.337	9.32E+01	9.43E+01	9.55E+01
3834.819	9.30E+01	9.42E+01	9.54E+01
3835.301	9.29E+01	9.43E+01	9.54E+01
3835.783	9.28E+01	9.43E+01	9.53E+01
3836.265	9.27E+01	9.44E+01	9.53E+01
3836.748	9.26E+01	9.44E+01	9.53E+01
3837.23	9.25E+01	9.45E+01	9.52E+01
3837.712	9.24E+01	9.45E+01	9.52E+01
3838.194	9.23E+01	9.44E+01	9.52E+01
3838.676	9.22E+01	9.44E+01	9.51E+01
3839.158	9.23E+01	9.44E+01	9.52E+01
3839.64	9.24E+01	9.44E+01	9.52E+01
3840.123	9.26E+01	9.44E+01	9.53E+01
3840.604	9.29E+01	9.45E+01	9.54E+01
3841.087	9.31E+01	9.45E+01	9.55E+01
3841.569	9.33E+01	9.46E+01	9.56E+01
3842.051	9.35E+01	9.46E+01	9.57E+01
3842.533	9.37E+01	9.46E+01	9.57E+01
3843.015	9.38E+01	9.46E+01	9.57E+01

3843.497	9.39E+01	9.46E+01	9.58E+01
3843.979	9.41E+01	9.46E+01	9.58E+01
3844.462	9.42E+01	9.46E+01	9.58E+01
3844.944	9.43E+01	9.46E+01	9.59E+01
3845.426	9.45E+01	9.47E+01	9.59E+01
3845.908	9.46E+01	9.47E+01	9.60E+01
3846.39	9.46E+01	9.47E+01	9.60E+01
3846.872	9.46E+01	9.47E+01	9.60E+01
3847.354	9.46E+01	9.47E+01	9.60E+01
3847.836	9.45E+01	9.47E+01	9.60E+01
3848.319	9.44E+01	9.46E+01	9.60E+01
3848.801	9.42E+01	9.45E+01	9.59E+01
3849.283	9.40E+01	9.45E+01	9.58E+01
3849.765	9.37E+01	9.43E+01	9.56E+01
3850.247	9.34E+01	9.42E+01	9.54E+01
3850.729	9.30E+01	9.41E+01	9.52E+01
3851.211	9.25E+01	9.39E+01	9.49E+01
3851.693	9.20E+01	9.38E+01	9.47E+01
3852.176	9.16E+01	9.36E+01	9.44E+01
3852.658	9.13E+01	9.35E+01	9.44E+01
3853.14	9.13E+01	9.36E+01	9.44E+01
3853.622	9.15E+01	9.37E+01	9.47E+01
3854.104	9.20E+01	9.39E+01	9.50E+01
3854.586	9.24E+01	9.40E+01	9.52E+01
3855.068	9.28E+01	9.41E+01	9.54E+01
3855.551	9.31E+01	9.42E+01	9.55E+01
3856.032	9.34E+01	9.42E+01	9.56E+01
3856.515	9.36E+01	9.42E+01	9.57E+01
3856.997	9.39E+01	9.43E+01	9.57E+01
3857.479	9.41E+01	9.43E+01	9.58E+01
3857.961	9.42E+01	9.43E+01	9.58E+01
3858.443	9.44E+01	9.44E+01	9.59E+01
3858.925	9.44E+01	9.44E+01	9.59E+01
3859.407	9.44E+01	9.44E+01	9.59E+01
3859.89	9.44E+01	9.44E+01	9.59E+01
3860.372	9.43E+01	9.44E+01	9.59E+01
3860.854	9.41E+01	9.44E+01	9.58E+01
3861.336	9.40E+01	9.43E+01	9.58E+01
3861.818	9.38E+01	9.43E+01	9.58E+01
3862.3	9.37E+01	9.43E+01	9.57E+01
3862.782	9.36E+01	9.43E+01	9.57E+01
3863.264	9.35E+01	9.44E+01	9.57E+01
3863.747	9.35E+01	9.44E+01	9.56E+01
3864.229	9.35E+01	9.45E+01	9.57E+01
3864.711	9.35E+01	9.45E+01	9.57E+01
3865.193	9.36E+01	9.46E+01	9.58E+01
3865.675	9.38E+01	9.46E+01	9.59E+01

3866.157	9.39E+01	9.46E+01	9.60E+01
3866.639	9.39E+01	9.47E+01	9.60E+01
3867.121	9.39E+01	9.46E+01	9.60E+01
3867.604	9.38E+01	9.46E+01	9.60E+01
3868.086	9.36E+01	9.46E+01	9.59E+01
3868.568	9.34E+01	9.45E+01	9.57E+01
3869.05	9.32E+01	9.45E+01	9.56E+01
3869.532	9.30E+01	9.44E+01	9.54E+01
3870.014	9.30E+01	9.44E+01	9.54E+01
3870.496	9.30E+01	9.44E+01	9.54E+01
3870.979	9.32E+01	9.44E+01	9.54E+01
3871.46	9.34E+01	9.45E+01	9.55E+01
3871.943	9.35E+01	9.45E+01	9.56E+01
3872.425	9.36E+01	9.46E+01	9.56E+01
3872.907	9.36E+01	9.46E+01	9.57E+01
3873.389	9.37E+01	9.46E+01	9.57E+01
3873.871	9.38E+01	9.46E+01	9.58E+01
3874.353	9.40E+01	9.46E+01	9.58E+01
3874.835	9.42E+01	9.46E+01	9.59E+01
3875.318	9.44E+01	9.46E+01	9.60E+01
3875.8	9.45E+01	9.46E+01	9.61E+01
3876.282	9.46E+01	9.46E+01	9.61E+01
3876.764	9.47E+01	9.46E+01	9.61E+01
3877.246	9.47E+01	9.46E+01	9.61E+01
3877.728	9.46E+01	9.46E+01	9.60E+01
3878.21	9.45E+01	9.45E+01	9.59E+01
3878.692	9.43E+01	9.45E+01	9.58E+01
3879.175	9.40E+01	9.44E+01	9.57E+01
3879.657	9.38E+01	9.44E+01	9.56E+01
3880.139	9.36E+01	9.44E+01	9.55E+01
3880.621	9.35E+01	9.44E+01	9.54E+01
3881.103	9.35E+01	9.44E+01	9.54E+01
3881.585	9.35E+01	9.44E+01	9.54E+01
3882.067	9.36E+01	9.44E+01	9.55E+01
3882.549	9.36E+01	9.44E+01	9.55E+01
3883.031	9.36E+01	9.45E+01	9.56E+01
3883.514	9.36E+01	9.45E+01	9.56E+01
3883.996	9.36E+01	9.45E+01	9.56E+01
3884.478	9.35E+01	9.45E+01	9.56E+01
3884.96	9.36E+01	9.44E+01	9.57E+01
3885.442	9.36E+01	9.44E+01	9.57E+01
3885.924	9.38E+01	9.44E+01	9.58E+01
3886.406	9.39E+01	9.44E+01	9.59E+01
3886.888	9.41E+01	9.45E+01	9.59E+01
3887.371	9.43E+01	9.45E+01	9.60E+01
3887.853	9.44E+01	9.45E+01	9.60E+01
3888.335	9.44E+01	9.45E+01	9.60E+01

3888.817	9.44E+01	9.45E+01	9.60E+01
3889.299	9.43E+01	9.45E+01	9.60E+01
3889.781	9.42E+01	9.44E+01	9.59E+01
3890.263	9.40E+01	9.44E+01	9.58E+01
3890.746	9.39E+01	9.43E+01	9.57E+01
3891.228	9.38E+01	9.43E+01	9.56E+01
3891.71	9.37E+01	9.43E+01	9.56E+01
3892.192	9.37E+01	9.43E+01	9.55E+01
3892.674	9.38E+01	9.43E+01	9.56E+01
3893.156	9.40E+01	9.43E+01	9.56E+01
3893.638	9.41E+01	9.44E+01	9.57E+01
3894.12	9.43E+01	9.44E+01	9.57E+01
3894.603	9.44E+01	9.45E+01	9.58E+01
3895.085	9.46E+01	9.45E+01	9.59E+01
3895.567	9.46E+01	9.46E+01	9.60E+01
3896.049	9.47E+01	9.46E+01	9.60E+01
3896.531	9.46E+01	9.46E+01	9.60E+01
3897.013	9.46E+01	9.45E+01	9.60E+01
3897.495	9.44E+01	9.45E+01	9.60E+01
3897.977	9.42E+01	9.45E+01	9.60E+01
3898.459	9.40E+01	9.45E+01	9.59E+01
3898.942	9.38E+01	9.44E+01	9.58E+01
3899.424	9.36E+01	9.44E+01	9.58E+01
3899.906	9.34E+01	9.44E+01	9.57E+01
3900.388	9.33E+01	9.45E+01	9.57E+01
3900.87	9.31E+01	9.44E+01	9.57E+01
3901.352	9.30E+01	9.44E+01	9.56E+01
3901.834	9.29E+01	9.44E+01	9.55E+01
3902.317	9.28E+01	9.43E+01	9.54E+01
3902.799	9.27E+01	9.43E+01	9.54E+01
3903.281	9.27E+01	9.43E+01	9.53E+01
3903.763	9.28E+01	9.43E+01	9.53E+01
3904.245	9.30E+01	9.44E+01	9.54E+01
3904.727	9.33E+01	9.45E+01	9.55E+01
3905.209	9.36E+01	9.46E+01	9.57E+01
3905.691	9.40E+01	9.47E+01	9.59E+01
3906.174	9.43E+01	9.48E+01	9.61E+01
3906.656	9.45E+01	9.48E+01	9.62E+01
3907.138	9.47E+01	9.48E+01	9.63E+01
3907.62	9.49E+01	9.48E+01	9.64E+01
3908.102	9.50E+01	9.48E+01	9.64E+01
3908.584	9.51E+01	9.48E+01	9.64E+01
3909.066	9.51E+01	9.48E+01	9.64E+01
3909.548	9.51E+01	9.48E+01	9.64E+01
3910.031	9.51E+01	9.48E+01	9.63E+01
3910.513	9.51E+01	9.48E+01	9.63E+01
3910.995	9.51E+01	9.48E+01	9.63E+01

3911.477	9.51E+01	9.48E+01	9.63E+01
3911.959	9.51E+01	9.48E+01	9.63E+01
3912.441	9.51E+01	9.49E+01	9.63E+01
3912.923	9.50E+01	9.49E+01	9.63E+01
3913.406	9.50E+01	9.49E+01	9.63E+01
3913.887	9.49E+01	9.49E+01	9.62E+01
3914.37	9.48E+01	9.49E+01	9.62E+01
3914.852	9.46E+01	9.48E+01	9.61E+01
3915.334	9.45E+01	9.48E+01	9.60E+01
3915.816	9.44E+01	9.47E+01	9.59E+01
3916.298	9.42E+01	9.47E+01	9.59E+01
3916.78	9.41E+01	9.47E+01	9.59E+01
3917.262	9.41E+01	9.46E+01	9.59E+01
3917.745	9.41E+01	9.46E+01	9.59E+01
3918.227	9.41E+01	9.47E+01	9.60E+01
3918.709	9.42E+01	9.47E+01	9.61E+01
3919.191	9.43E+01	9.47E+01	9.62E+01
3919.673	9.44E+01	9.47E+01	9.62E+01
3920.155	9.45E+01	9.47E+01	9.63E+01
3920.637	9.46E+01	9.47E+01	9.63E+01
3921.119	9.46E+01	9.47E+01	9.63E+01
3921.602	9.46E+01	9.46E+01	9.63E+01
3922.084	9.45E+01	9.46E+01	9.62E+01
3922.566	9.45E+01	9.46E+01	9.62E+01
3923.048	9.44E+01	9.45E+01	9.61E+01
3923.53	9.43E+01	9.45E+01	9.61E+01
3924.012	9.42E+01	9.45E+01	9.60E+01
3924.494	9.43E+01	9.45E+01	9.60E+01
3924.976	9.44E+01	9.46E+01	9.61E+01
3925.458	9.45E+01	9.47E+01	9.61E+01
3925.941	9.46E+01	9.47E+01	9.61E+01
3926.423	9.47E+01	9.48E+01	9.61E+01
3926.905	9.48E+01	9.48E+01	9.61E+01
3927.387	9.48E+01	9.48E+01	9.61E+01
3927.869	9.47E+01	9.48E+01	9.61E+01
3928.351	9.46E+01	9.47E+01	9.61E+01
3928.833	9.45E+01	9.47E+01	9.61E+01
3929.315	9.43E+01	9.47E+01	9.61E+01
3929.798	9.42E+01	9.46E+01	9.60E+01
3930.28	9.41E+01	9.46E+01	9.60E+01
3930.762	9.41E+01	9.46E+01	9.60E+01
3931.244	9.41E+01	9.46E+01	9.60E+01
3931.726	9.42E+01	9.46E+01	9.60E+01
3932.208	9.42E+01	9.46E+01	9.60E+01
3932.69	9.44E+01	9.47E+01	9.61E+01
3933.173	9.45E+01	9.47E+01	9.61E+01
3933.655	9.47E+01	9.47E+01	9.61E+01

3934.137	9.48E+01	9.47E+01	9.61E+01
3934.619	9.49E+01	9.48E+01	9.61E+01
3935.101	9.50E+01	9.48E+01	9.62E+01
3935.583	9.51E+01	9.48E+01	9.62E+01
3936.065	9.51E+01	9.48E+01	9.62E+01
3936.547	9.51E+01	9.48E+01	9.62E+01
3937.03	9.51E+01	9.48E+01	9.62E+01
3937.512	9.51E+01	9.48E+01	9.63E+01
3937.994	9.50E+01	9.48E+01	9.63E+01
3938.476	9.50E+01	9.48E+01	9.63E+01
3938.958	9.50E+01	9.47E+01	9.63E+01
3939.44	9.50E+01	9.47E+01	9.63E+01
3939.922	9.49E+01	9.47E+01	9.63E+01
3940.404	9.48E+01	9.46E+01	9.62E+01
3940.886	9.47E+01	9.46E+01	9.61E+01
3941.369	9.46E+01	9.46E+01	9.61E+01
3941.851	9.44E+01	9.45E+01	9.60E+01
3942.333	9.44E+01	9.45E+01	9.59E+01
3942.815	9.43E+01	9.45E+01	9.59E+01
3943.297	9.44E+01	9.45E+01	9.60E+01
3943.779	9.45E+01	9.45E+01	9.60E+01
3944.261	9.45E+01	9.45E+01	9.61E+01
3944.743	9.46E+01	9.45E+01	9.61E+01
3945.226	9.47E+01	9.46E+01	9.62E+01
3945.708	9.47E+01	9.46E+01	9.62E+01
3946.19	9.46E+01	9.46E+01	9.62E+01
3946.672	9.45E+01	9.46E+01	9.61E+01
3947.154	9.44E+01	9.46E+01	9.61E+01
3947.636	9.44E+01	9.46E+01	9.60E+01
3948.118	9.43E+01	9.46E+01	9.60E+01
3948.601	9.43E+01	9.46E+01	9.60E+01
3949.083	9.44E+01	9.47E+01	9.60E+01
3949.565	9.44E+01	9.47E+01	9.60E+01
3950.047	9.45E+01	9.47E+01	9.61E+01
3950.529	9.46E+01	9.48E+01	9.61E+01
3951.011	9.46E+01	9.48E+01	9.61E+01
3951.493	9.47E+01	9.48E+01	9.62E+01
3951.975	9.48E+01	9.48E+01	9.62E+01
3952.458	9.48E+01	9.48E+01	9.62E+01
3952.94	9.48E+01	9.48E+01	9.62E+01
3953.422	9.49E+01	9.48E+01	9.63E+01
3953.904	9.49E+01	9.48E+01	9.63E+01
3954.386	9.50E+01	9.48E+01	9.63E+01
3954.868	9.50E+01	9.48E+01	9.63E+01
3955.35	9.50E+01	9.48E+01	9.62E+01
3955.832	9.50E+01	9.48E+01	9.62E+01
3956.314	9.49E+01	9.48E+01	9.62E+01

3956.797	9.49E+01	9.47E+01	9.61E+01
3957.279	9.49E+01	9.47E+01	9.61E+01
3957.761	9.49E+01	9.47E+01	9.61E+01
3958.243	9.48E+01	9.47E+01	9.61E+01
3958.725	9.48E+01	9.47E+01	9.60E+01
3959.207	9.48E+01	9.47E+01	9.60E+01
3959.689	9.48E+01	9.47E+01	9.60E+01
3960.171	9.48E+01	9.47E+01	9.61E+01
3960.654	9.48E+01	9.47E+01	9.61E+01
3961.136	9.48E+01	9.47E+01	9.62E+01
3961.618	9.48E+01	9.47E+01	9.62E+01
3962.1	9.49E+01	9.48E+01	9.62E+01
3962.582	9.49E+01	9.48E+01	9.63E+01
3963.064	9.50E+01	9.48E+01	9.63E+01
3963.546	9.50E+01	9.48E+01	9.63E+01
3964.029	9.51E+01	9.48E+01	9.63E+01
3964.51	9.51E+01	9.48E+01	9.63E+01
3964.993	9.50E+01	9.48E+01	9.63E+01
3965.475	9.50E+01	9.47E+01	9.62E+01
3965.957	9.50E+01	9.47E+01	9.62E+01
3966.439	9.49E+01	9.47E+01	9.62E+01
3966.921	9.49E+01	9.46E+01	9.62E+01
3967.403	9.49E+01	9.46E+01	9.62E+01
3967.885	9.49E+01	9.46E+01	9.62E+01
3968.368	9.49E+01	9.46E+01	9.63E+01
3968.85	9.49E+01	9.47E+01	9.63E+01
3969.332	9.49E+01	9.47E+01	9.64E+01
3969.814	9.49E+01	9.48E+01	9.64E+01
3970.296	9.49E+01	9.48E+01	9.64E+01
3970.778	9.49E+01	9.48E+01	9.64E+01
3971.26	9.49E+01	9.48E+01	9.63E+01
3971.742	9.49E+01	9.48E+01	9.63E+01
3972.225	9.48E+01	9.48E+01	9.62E+01
3972.707	9.48E+01	9.48E+01	9.62E+01
3973.189	9.48E+01	9.48E+01	9.62E+01
3973.671	9.48E+01	9.47E+01	9.61E+01
3974.153	9.48E+01	9.47E+01	9.61E+01
3974.635	9.48E+01	9.47E+01	9.61E+01
3975.117	9.48E+01	9.47E+01	9.61E+01
3975.599	9.49E+01	9.47E+01	9.61E+01
3976.082	9.49E+01	9.48E+01	9.62E+01
3976.564	9.50E+01	9.48E+01	9.62E+01
3977.046	9.51E+01	9.48E+01	9.63E+01
3977.528	9.51E+01	9.49E+01	9.63E+01
3978.01	9.52E+01	9.49E+01	9.63E+01
3978.492	9.52E+01	9.49E+01	9.63E+01
3978.974	9.52E+01	9.48E+01	9.63E+01

3979.457	9.51E+01	9.48E+01	9.62E+01
3979.938	9.51E+01	9.48E+01	9.62E+01
3980.421	9.50E+01	9.47E+01	9.62E+01
3980.903	9.49E+01	9.47E+01	9.62E+01
3981.385	9.49E+01	9.47E+01	9.62E+01
3981.867	9.49E+01	9.47E+01	9.62E+01
3982.349	9.49E+01	9.47E+01	9.62E+01
3982.831	9.49E+01	9.47E+01	9.63E+01
3983.313	9.50E+01	9.48E+01	9.63E+01
3983.796	9.50E+01	9.48E+01	9.63E+01
3984.278	9.50E+01	9.49E+01	9.64E+01
3984.76	9.51E+01	9.49E+01	9.64E+01
3985.242	9.51E+01	9.49E+01	9.64E+01
3985.724	9.52E+01	9.50E+01	9.64E+01
3986.206	9.52E+01	9.50E+01	9.64E+01
3986.688	9.52E+01	9.50E+01	9.63E+01
3987.17	9.52E+01	9.50E+01	9.63E+01
3987.653	9.52E+01	9.50E+01	9.63E+01
3988.135	9.52E+01	9.49E+01	9.63E+01
3988.617	9.52E+01	9.49E+01	9.63E+01
3989.099	9.52E+01	9.49E+01	9.63E+01
3989.581	9.51E+01	9.49E+01	9.63E+01
3990.063	9.51E+01	9.49E+01	9.63E+01
3990.545	9.51E+01	9.49E+01	9.63E+01
3991.027	9.51E+01	9.49E+01	9.63E+01
3991.51	9.51E+01	9.49E+01	9.63E+01
3991.992	9.51E+01	9.49E+01	9.63E+01
3992.474	9.51E+01	9.49E+01	9.63E+01
3992.956	9.50E+01	9.49E+01	9.63E+01
3993.438	9.50E+01	9.49E+01	9.63E+01
3993.92	9.50E+01	9.49E+01	9.63E+01
3994.402	9.50E+01	9.49E+01	9.63E+01
3994.885	9.50E+01	9.49E+01	9.63E+01
3995.367	9.51E+01	9.49E+01	9.63E+01
3995.849	9.52E+01	9.50E+01	9.64E+01
3996.331	9.52E+01	9.50E+01	9.64E+01
3996.813	9.52E+01	9.50E+01	9.64E+01
3997.295	9.53E+01	9.50E+01	9.64E+01
3997.777	9.52E+01	9.50E+01	9.64E+01
3998.259	9.52E+01	9.50E+01	9.64E+01
3998.741	9.52E+01	9.50E+01	9.63E+01
3999.224	9.52E+01	9.49E+01	9.63E+01
3999.706	9.52E+01	9.49E+01	9.63E+01
4000.188	9.52E+01	9.49E+01	9.63E+01

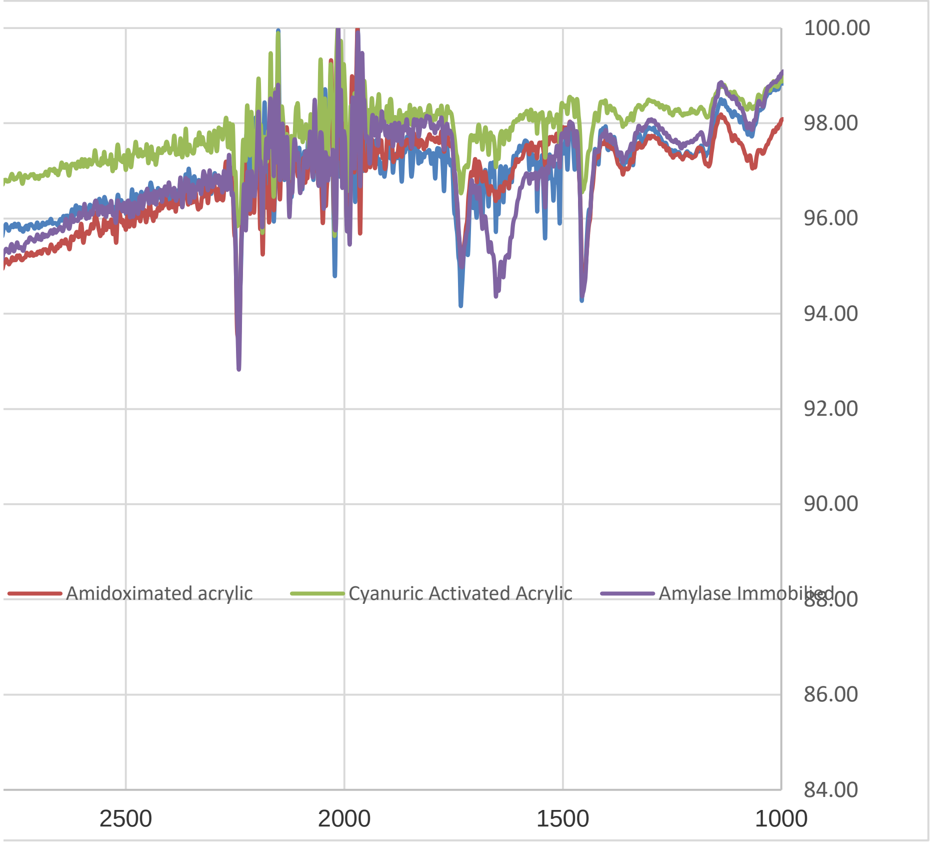

0.00E+00
9.89E+01
9.86E+01
9.85E+01
9.85E+01
9.86E+01
9.87E+01
9.88E+01
9.89E+01
9.89E+01
9.88E+01
9.87E+01
9.86E+01
9.85E+01
9.85E+01
9.85E+01
9.85E+01
9.86E+01
9.88E+01
9.89E+01
9.90E+01
9.91E+01
9.93E+01
9.94E+01
9.95E+01
9.96E+01
9.96E+01
9.95E+01
9.94E+01
9.93E+01
9.91E+01
9.90E+01
9.88E+01
9.87E+01
9.87E+01
9.86E+01
9.86E+01
9.86E+01
9.85E+01
9.85E+01
9.86E+01
9.86E+01
9.87E+01
9.89E+01
9.90E+01
9.91E+01

9.92E+01
9.93E+01
9.93E+01
9.93E+01
9.93E+01
9.92E+01
9.91E+01
9.91E+01
9.90E+01
9.90E+01
9.90E+01
9.89E+01
9.89E+01
9.88E+01
9.86E+01
9.85E+01
9.84E+01
9.83E+01
9.83E+01
9.83E+01
9.83E+01
9.83E+01
9.83E+01
9.84E+01
9.84E+01
9.84E+01
9.84E+01
9.84E+01
9.84E+01
9.84E+01
9.84E+01
9.84E+01
9.83E+01
9.83E+01
9.83E+01
9.84E+01
9.84E+01
9.84E+01
9.85E+01
9.85E+01
9.85E+01
9.86E+01
9.86E+01
9.86E+01
9.86E+01
9.85E+01
9.84E+01

9.83E+01
9.82E+01
9.81E+01
9.81E+01
9.81E+01
9.81E+01
9.81E+01
9.82E+01
9.83E+01
9.84E+01
9.84E+01
9.84E+01
9.85E+01
9.85E+01
9.86E+01
9.86E+01
9.87E+01
9.87E+01
9.86E+01
9.85E+01
9.84E+01
9.83E+01
9.82E+01
9.81E+01
9.80E+01
9.80E+01
9.81E+01
9.82E+01
9.83E+01
9.84E+01
9.85E+01
9.85E+01
9.86E+01
9.86E+01
9.85E+01
9.85E+01
9.85E+01
9.85E+01
9.85E+01
9.85E+01
9.85E+01
9.85E+01
9.85E+01
9.85E+01
9.84E+01
9.84E+01
9.83E+01

9.82E+01
9.82E+01
9.82E+01
9.82E+01
9.82E+01
9.82E+01
9.82E+01
9.82E+01
9.81E+01
9.81E+01
9.80E+01
9.80E+01
9.80E+01
9.80E+01
9.80E+01
9.80E+01
9.80E+01
9.81E+01
9.81E+01
9.82E+01
9.83E+01
9.83E+01
9.84E+01
9.84E+01
9.85E+01
9.85E+01
9.85E+01
9.86E+01
9.86E+01
9.86E+01
9.86E+01
9.86E+01
9.85E+01
9.85E+01
9.84E+01
9.83E+01
9.83E+01
9.83E+01
9.83E+01
9.83E+01
9.83E+01
9.82E+01
9.82E+01
9.82E+01
9.82E+01
9.82E+01
9.81E+01

9.81E+01
9.80E+01
9.80E+01
9.79E+01
9.79E+01
9.79E+01
9.79E+01
9.79E+01
9.80E+01
9.80E+01
9.81E+01
9.82E+01
9.83E+01
9.83E+01
9.84E+01
9.84E+01
9.84E+01
9.84E+01
9.83E+01
9.83E+01
9.83E+01
9.82E+01
9.82E+01
9.82E+01
9.82E+01
9.82E+01
9.82E+01
9.82E+01
9.82E+01
9.82E+01
9.82E+01
9.81E+01
9.81E+01
9.81E+01
9.81E+01
9.81E+01
9.82E+01
9.82E+01
9.82E+01
9.83E+01
9.83E+01
9.83E+01
9.83E+01
9.83E+01
9.82E+01
9.81E+01
9.80E+01

9.79E+01
9.79E+01
9.79E+01
9.79E+01
9.80E+01
9.80E+01
9.81E+01
9.81E+01
9.81E+01
9.81E+01
9.80E+01
9.80E+01
9.80E+01
9.80E+01
9.80E+01
9.80E+01
9.80E+01
9.80E+01
9.80E+01
9.79E+01
9.79E+01
9.79E+01
9.78E+01
9.78E+01
9.78E+01
9.78E+01
9.78E+01
9.78E+01
9.78E+01
9.78E+01
9.78E+01
9.78E+01
9.78E+01
9.77E+01
9.77E+01
9.77E+01
9.77E+01
9.77E+01
9.77E+01
9.77E+01
9.78E+01
9.78E+01
9.78E+01
9.79E+01
9.79E+01
9.79E+01
9.80E+01

9.80E+01
9.80E+01
9.79E+01
9.79E+01
9.79E+01
9.78E+01
9.78E+01
9.77E+01
9.77E+01
9.77E+01
9.77E+01
9.77E+01
9.77E+01
9.77E+01
9.77E+01
9.77E+01
9.77E+01
9.77E+01
9.78E+01
9.78E+01
9.78E+01
9.78E+01
9.78E+01
9.78E+01
9.79E+01
9.79E+01
9.79E+01
9.80E+01
9.80E+01
9.80E+01
9.80E+01
9.80E+01
9.79E+01
9.79E+01
9.79E+01
9.78E+01
9.78E+01
9.78E+01
9.79E+01
9.79E+01
9.79E+01
9.79E+01
9.79E+01
9.79E+01
9.79E+01
9.79E+01
9.79E+01

9.79E+01
9.80E+01
9.80E+01
9.80E+01
9.80E+01
9.80E+01
9.80E+01
9.80E+01
9.79E+01
9.79E+01
9.79E+01
9.78E+01
9.78E+01
9.78E+01
9.77E+01
9.77E+01
9.77E+01
9.78E+01
9.78E+01
9.78E+01
9.78E+01
9.79E+01
9.79E+01
9.79E+01
9.79E+01
9.79E+01
9.80E+01
9.80E+01
9.80E+01
9.81E+01
9.81E+01
9.81E+01
9.81E+01
9.81E+01
9.81E+01
9.81E+01
9.81E+01
9.81E+01
9.81E+01
9.81E+01
9.80E+01
9.80E+01
9.80E+01
9.80E+01
9.80E+01
9.81E+01
9.81E+01

9.81E+01
9.81E+01
9.82E+01
9.82E+01
9.82E+01
9.82E+01
9.83E+01
9.83E+01
9.83E+01
9.82E+01
9.82E+01
9.82E+01
9.82E+01
9.82E+01
9.82E+01
9.81E+01
9.81E+01
9.81E+01
9.81E+01
9.81E+01
9.81E+01
9.81E+01
9.81E+01
9.81E+01
9.81E+01
9.80E+01
9.80E+01
9.80E+01
9.80E+01
9.80E+01
9.80E+01
9.80E+01
9.80E+01
9.80E+01
9.81E+01
9.81E+01
9.81E+01
9.80E+01
9.80E+01
9.80E+01
9.80E+01
9.80E+01
9.80E+01
9.80E+01
9.80E+01
9.81E+01
9.81E+01

9.81E+01
9.81E+01
9.81E+01
9.81E+01
9.81E+01
9.81E+01
9.81E+01
9.81E+01
9.81E+01
9.81E+01
9.81E+01
9.81E+01
9.81E+01
9.81E+01
9.82E+01
9.82E+01
9.81E+01
9.81E+01
9.81E+01
9.81E+01
9.80E+01
9.80E+01
9.80E+01
9.80E+01
9.80E+01
9.80E+01
9.80E+01
9.80E+01
9.80E+01
9.80E+01
9.80E+01
9.81E+01
9.81E+01
9.81E+01
9.81E+01
9.80E+01
9.80E+01
9.80E+01
9.80E+01
9.80E+01
9.79E+01
9.79E+01
9.79E+01
9.79E+01
9.79E+01
9.79E+01
9.80E+01

9.80E+01
9.80E+01
9.81E+01
9.81E+01
9.82E+01
9.82E+01
9.82E+01
9.82E+01
9.82E+01
9.81E+01
9.81E+01
9.80E+01
9.80E+01
9.80E+01
9.80E+01
9.80E+01
9.80E+01
9.80E+01
9.80E+01
9.81E+01
9.81E+01
9.81E+01
9.80E+01
9.80E+01
9.80E+01
9.79E+01
9.79E+01
9.79E+01
9.79E+01
9.79E+01
9.78E+01
9.78E+01
9.78E+01
9.78E+01
9.78E+01
9.78E+01
9.78E+01
9.78E+01
9.78E+01
9.79E+01
9.79E+01
9.79E+01
9.80E+01
9.80E+01
9.80E+01
9.80E+01
9.80E+01

9.80E+01
9.80E+01
9.80E+01
9.80E+01
9.80E+01
9.80E+01
9.80E+01
9.80E+01
9.80E+01
9.80E+01
9.80E+01
9.80E+01
9.80E+01
9.80E+01
9.80E+01
9.80E+01
9.80E+01
9.79E+01
9.79E+01
9.79E+01
9.79E+01
9.79E+01
9.79E+01
9.80E+01
9.80E+01
9.80E+01
9.80E+01
9.80E+01
9.80E+01
9.80E+01
9.80E+01
9.80E+01
9.80E+01
9.80E+01
9.80E+01
9.80E+01
9.80E+01
9.80E+01
9.80E+01
9.80E+01
9.80E+01
9.80E+01
9.80E+01
9.80E+01
9.80E+01
9.80E+01
9.80E+01

9.80E+01
9.80E+01
9.80E+01
9.79E+01
9.79E+01
9.79E+01
9.79E+01
9.79E+01
9.79E+01
9.79E+01
9.79E+01
9.79E+01
9.79E+01
9.79E+01
9.79E+01
9.80E+01
9.80E+01
9.80E+01
9.80E+01
9.80E+01
9.79E+01
9.79E+01
9.79E+01
9.79E+01
9.79E+01
9.79E+01
9.79E+01
9.78E+01
9.78E+01
9.78E+01
9.78E+01
9.78E+01
9.79E+01
9.79E+01
9.79E+01
9.80E+01
9.80E+01
9.80E+01
9.80E+01
9.79E+01
9.79E+01
9.79E+01
9.79E+01
9.79E+01
9.79E+01
9.79E+01
9.80E+01

9.80E+01
9.80E+01
9.79E+01
9.79E+01
9.79E+01
9.79E+01
9.79E+01
9.79E+01
9.79E+01
9.79E+01
9.79E+01
9.79E+01
9.79E+01
9.79E+01
9.79E+01
9.79E+01
9.79E+01
9.79E+01
9.79E+01
9.79E+01
9.79E+01
9.79E+01
9.79E+01
9.80E+01
9.80E+01
9.80E+01
9.80E+01
9.80E+01
9.81E+01
9.81E+01
9.81E+01
9.81E+01
9.80E+01
9.80E+01
9.80E+01
9.80E+01
9.80E+01
9.80E+01
9.80E+01
9.80E+01
9.80E+01
9.80E+01
9.80E+01
9.80E+01
9.79E+01
9.79E+01
9.79E+01

9.79E+01
9.80E+01
9.80E+01
9.80E+01
9.80E+01
9.80E+01
9.80E+01
9.80E+01
9.79E+01
9.79E+01
9.79E+01
9.79E+01
9.79E+01
9.79E+01
9.79E+01
9.79E+01
9.79E+01
9.79E+01
9.80E+01
9.80E+01
9.80E+01
9.80E+01
9.80E+01
9.80E+01
9.80E+01
9.80E+01
9.80E+01
9.80E+01
9.80E+01
9.80E+01
9.80E+01
9.81E+01
9.81E+01
9.81E+01
9.81E+01
9.80E+01
9.80E+01
9.80E+01
9.80E+01
9.80E+01
9.80E+01
9.79E+01
9.79E+01
9.79E+01
9.79E+01
9.79E+01
9.79E+01

9.79E+01
9.79E+01
9.80E+01
9.79E+01
9.79E+01
9.79E+01
9.79E+01
9.79E+01
9.79E+01
9.79E+01
9.79E+01
9.79E+01
9.79E+01
9.79E+01
9.79E+01
9.79E+01
9.79E+01
9.80E+01
9.80E+01
9.80E+01
9.79E+01
9.79E+01
9.79E+01
9.79E+01
9.79E+01
9.79E+01
9.80E+01
9.80E+01
9.80E+01
9.80E+01
9.80E+01
9.79E+01
9.79E+01
9.79E+01
9.79E+01
9.79E+01
9.79E+01
9.79E+01
9.79E+01
9.79E+01
9.79E+01
9.80E+01
9.80E+01
9.80E+01
9.80E+01
9.80E+01
9.80E+01

9.81E+01
9.80E+01
9.80E+01
9.80E+01
9.80E+01
9.80E+01
9.80E+01
9.80E+01
9.80E+01
9.79E+01
9.79E+01
9.79E+01
9.79E+01
9.79E+01
9.80E+01
9.80E+01
9.80E+01
9.80E+01
9.81E+01
9.81E+01
9.81E+01
9.81E+01
9.80E+01
9.80E+01
9.80E+01
9.80E+01
9.80E+01
9.80E+01
9.80E+01
9.81E+01
9.81E+01
9.81E+01
9.81E+01
9.81E+01
9.81E+01
9.81E+01
9.81E+01
9.81E+01
9.81E+01
9.81E+01
9.81E+01
9.81E+01
9.81E+01
9.80E+01
9.80E+01
9.80E+01
9.80E+01

9.80E+01
9.80E+01
9.80E+01
9.80E+01
9.80E+01
9.80E+01
9.80E+01
9.80E+01
9.80E+01
9.80E+01
9.81E+01
9.81E+01
9.81E+01
9.82E+01
9.82E+01
9.82E+01
9.82E+01
9.82E+01
9.82E+01
9.82E+01
9.82E+01
9.82E+01
9.82E+01
9.82E+01
9.82E+01
9.82E+01
9.82E+01
9.83E+01
9.83E+01
9.83E+01
9.83E+01
9.84E+01
9.84E+01
9.84E+01
9.84E+01
9.84E+01
9.84E+01
9.84E+01
9.84E+01
9.84E+01
9.84E+01
9.84E+01
9.84E+01
9.84E+01
9.84E+01
9.83E+01
9.83E+01

9.83E+01
9.83E+01
9.83E+01
9.83E+01
9.83E+01
9.83E+01
9.83E+01
9.83E+01
9.84E+01
9.84E+01
9.84E+01
9.84E+01
9.83E+01
9.83E+01
9.83E+01
9.83E+01
9.83E+01
9.83E+01
9.84E+01
9.84E+01
9.84E+01
9.84E+01
9.85E+01
9.85E+01
9.85E+01
9.85E+01
9.85E+01
9.85E+01
9.85E+01
9.85E+01
9.85E+01
9.84E+01
9.84E+01
9.84E+01
9.84E+01
9.84E+01
9.84E+01
9.84E+01
9.84E+01
9.84E+01
9.84E+01
9.84E+01
9.84E+01
9.85E+01
9.85E+01
9.85E+01
9.85E+01

9.85E+01
9.85E+01
9.85E+01
9.85E+01
9.85E+01
9.85E+01
9.85E+01
9.85E+01
9.85E+01
9.84E+01
9.85E+01
9.85E+01
9.85E+01
9.85E+01
9.85E+01
9.85E+01
9.85E+01
9.85E+01
9.85E+01
9.85E+01
9.85E+01
9.86E+01
9.86E+01
9.86E+01
9.86E+01
9.86E+01
9.86E+01
9.86E+01
9.86E+01
9.86E+01
9.86E+01
9.86E+01
9.86E+01
9.86E+01
9.86E+01
9.86E+01
9.86E+01
9.86E+01
9.87E+01
9.87E+01
9.87E+01
9.87E+01
9.86E+01
9.86E+01
9.86E+01
9.86E+01
9.86E+01

9.86E+01
9.86E+01
9.86E+01
9.87E+01
9.87E+01
9.87E+01
9.87E+01
9.87E+01
9.87E+01
9.88E+01
9.88E+01
9.88E+01
9.88E+01
9.88E+01
9.88E+01
9.87E+01
9.87E+01
9.87E+01
9.87E+01
9.86E+01
9.86E+01
9.86E+01
9.86E+01
9.86E+01
9.86E+01
9.87E+01
9.87E+01
9.87E+01
9.87E+01
9.87E+01
9.87E+01
9.87E+01
9.87E+01
9.87E+01
9.87E+01
9.87E+01
9.88E+01
9.88E+01
9.88E+01
9.89E+01
9.89E+01
9.89E+01
9.89E+01
9.89E+01
9.89E+01
9.89E+01
9.88E+01

9.88E+01
9.88E+01
9.88E+01
9.88E+01
9.88E+01
9.88E+01
9.88E+01
9.88E+01
9.89E+01
9.89E+01
9.89E+01
9.89E+01
9.89E+01
9.89E+01
9.89E+01
9.89E+01
9.89E+01
9.89E+01
9.89E+01
9.89E+01
9.89E+01
9.89E+01
9.89E+01
9.89E+01
9.89E+01
9.89E+01
9.89E+01
9.89E+01
9.89E+01
9.89E+01
9.89E+01
9.89E+01
9.89E+01
9.90E+01
9.90E+01
9.90E+01
9.90E+01
9.90E+01
9.90E+01
9.90E+01
9.89E+01
9.89E+01
9.89E+01
9.89E+01
9.89E+01
9.89E+01
9.89E+01

9.89E+01
9.89E+01
9.89E+01
9.89E+01
9.90E+01
9.90E+01
9.90E+01
9.89E+01
9.89E+01
9.89E+01
9.89E+01
9.89E+01
9.89E+01
9.89E+01
9.89E+01
9.89E+01
9.89E+01
9.89E+01
9.90E+01
9.90E+01
9.90E+01
9.90E+01
9.90E+01
9.90E+01
9.90E+01
9.89E+01
9.89E+01
9.89E+01
9.89E+01
9.89E+01
9.89E+01
9.89E+01
9.89E+01
9.89E+01
9.89E+01
9.89E+01
9.89E+01
9.89E+01
9.89E+01
9.89E+01
9.89E+01
9.89E+01
9.89E+01
9.89E+01
9.90E+01
9.90E+01
9.90E+01
9.90E+01

9.90E+01
9.90E+01
9.90E+01
9.90E+01
9.90E+01
9.90E+01
9.89E+01
9.89E+01
9.89E+01
9.89E+01
9.89E+01
9.89E+01
9.89E+01
9.89E+01
9.89E+01
9.89E+01
9.89E+01
9.89E+01
9.89E+01
9.89E+01
9.89E+01
9.89E+01
9.89E+01
9.89E+01
9.89E+01
9.89E+01
9.89E+01
9.89E+01
9.90E+01
9.90E+01
9.90E+01
9.90E+01
9.90E+01
9.89E+01
9.89E+01
9.89E+01
9.89E+01
9.89E+01
9.89E+01
9.89E+01
9.89E+01
9.89E+01
9.89E+01
9.89E+01
9.89E+01
9.89E+01
9.90E+01
9.90E+01

9.90E+01
9.90E+01
9.90E+01
9.90E+01
9.90E+01
9.90E+01
9.90E+01
9.90E+01
9.90E+01
9.90E+01
9.90E+01
9.90E+01
9.90E+01
9.89E+01
9.89E+01
9.89E+01
9.89E+01
9.89E+01
9.89E+01
9.90E+01
9.90E+01
9.89E+01
9.89E+01
9.89E+01
9.89E+01
9.89E+01
9.89E+01
9.89E+01
9.89E+01
9.89E+01
9.90E+01
9.90E+01
9.90E+01
9.90E+01
9.90E+01
9.90E+01
9.90E+01
9.90E+01
9.90E+01
9.90E+01
9.90E+01
9.90E+01
9.90E+01
9.90E+01
9.90E+01
9.91E+01
9.91E+01
9.91E+01

9.90E+01
9.90E+01
9.90E+01
9.90E+01
9.90E+01
9.90E+01
9.90E+01
9.90E+01
9.90E+01
9.90E+01
9.90E+01
9.90E+01
9.90E+01
9.90E+01
9.90E+01
9.91E+01
9.91E+01
9.91E+01
9.91E+01
9.92E+01
9.92E+01
9.92E+01
9.92E+01
9.92E+01
9.92E+01
9.92E+01
9.92E+01
9.92E+01
9.92E+01
9.92E+01
9.91E+01
9.91E+01
9.91E+01
9.91E+01
9.91E+01
9.91E+01
9.92E+01
9.92E+01
9.92E+01
9.92E+01
9.92E+01
9.92E+01
9.92E+01
9.92E+01
9.91E+01
9.91E+01
9.91E+01

9.91E+01
9.91E+01
9.91E+01
9.91E+01
9.91E+01
9.91E+01
9.90E+01
9.90E+01
9.90E+01
9.90E+01
9.91E+01
9.91E+01
9.91E+01
9.91E+01
9.91E+01
9.91E+01
9.91E+01
9.91E+01
9.91E+01
9.91E+01
9.91E+01
9.90E+01
9.90E+01
9.90E+01
9.90E+01
9.90E+01
9.90E+01
9.90E+01
9.90E+01
9.90E+01
9.90E+01
9.90E+01
9.90E+01
9.90E+01
9.90E+01
9.90E+01
9.90E+01
9.90E+01
9.90E+01
9.89E+01
9.89E+01
9.89E+01
9.89E+01
9.89E+01
9.89E+01
9.89E+01
9.89E+01

9.89E+01
9.89E+01
9.89E+01
9.89E+01
9.89E+01
9.89E+01
9.89E+01
9.89E+01
9.89E+01
9.89E+01
9.89E+01
9.89E+01
9.89E+01
9.89E+01
9.89E+01
9.89E+01
9.89E+01
9.88E+01
9.88E+01
9.88E+01
9.88E+01
9.88E+01
9.88E+01
9.88E+01
9.88E+01
9.88E+01
9.88E+01
9.88E+01
9.88E+01
9.88E+01
9.88E+01
9.88E+01
9.88E+01
9.88E+01
9.88E+01
9.88E+01
9.88E+01
9.88E+01
9.88E+01
9.88E+01
9.88E+01
9.88E+01
9.87E+01
9.87E+01
9.87E+01
9.87E+01
9.87E+01

9.87E+01
9.87E+01
9.86E+01
9.86E+01
9.86E+01
9.86E+01
9.86E+01
9.86E+01
9.85E+01
9.85E+01
9.85E+01
9.85E+01
9.84E+01
9.84E+01
9.84E+01
9.84E+01
9.84E+01
9.83E+01
9.83E+01
9.84E+01
9.84E+01
9.84E+01
9.84E+01
9.84E+01
9.84E+01
9.84E+01
9.84E+01
9.84E+01
9.84E+01
9.84E+01
9.84E+01
9.84E+01
9.84E+01
9.84E+01
9.85E+01
9.85E+01
9.85E+01
9.85E+01
9.85E+01
9.84E+01
9.84E+01
9.84E+01
9.84E+01
9.84E+01
9.83E+01
9.83E+01
9.83E+01

9.83E+01
9.82E+01
9.82E+01
9.82E+01
9.81E+01
9.81E+01
9.81E+01
9.81E+01
9.81E+01
9.81E+01
9.81E+01
9.81E+01
9.81E+01
9.81E+01
9.80E+01
9.80E+01
9.80E+01
9.79E+01
9.79E+01
9.79E+01
9.79E+01
9.79E+01
9.79E+01
9.79E+01
9.79E+01
9.79E+01
9.79E+01
9.79E+01
9.79E+01
9.79E+01
9.80E+01
9.80E+01
9.80E+01
9.80E+01
9.80E+01
9.80E+01
9.80E+01
9.80E+01
9.80E+01
9.80E+01
9.79E+01
9.79E+01
9.79E+01
9.79E+01
9.79E+01
9.79E+01
9.79E+01

9.79E+01
9.80E+01
9.80E+01
9.80E+01
9.80E+01
9.80E+01
9.81E+01
9.81E+01
9.81E+01
9.81E+01
9.81E+01
9.81E+01
9.82E+01
9.82E+01
9.82E+01
9.82E+01
9.82E+01
9.82E+01
9.82E+01
9.83E+01
9.83E+01
9.83E+01
9.83E+01
9.83E+01
9.83E+01
9.83E+01
9.83E+01
9.83E+01
9.83E+01
9.83E+01
9.83E+01
9.83E+01
9.83E+01
9.83E+01
9.83E+01
9.84E+01
9.84E+01
9.84E+01
9.84E+01
9.84E+01
9.84E+01
9.84E+01
9.84E+01
9.84E+01
9.84E+01
9.84E+01
9.84E+01

9.84E+01
9.85E+01
9.85E+01
9.85E+01
9.85E+01
9.85E+01
9.85E+01
9.85E+01
9.85E+01
9.85E+01
9.85E+01
9.85E+01
9.85E+01
9.85E+01
9.86E+01
9.86E+01
9.86E+01
9.86E+01
9.85E+01
9.85E+01
9.85E+01
9.85E+01
9.85E+01
9.85E+01
9.85E+01
9.85E+01
9.85E+01
9.85E+01
9.86E+01
9.86E+01
9.86E+01
9.86E+01
9.86E+01
9.86E+01
9.86E+01
9.86E+01
9.86E+01
9.86E+01
9.86E+01
9.86E+01
9.86E+01
9.86E+01
9.86E+01
9.86E+01
9.86E+01
9.86E+01
9.86E+01

9.86E+01
9.86E+01
9.87E+01
9.87E+01
9.87E+01
9.87E+01
9.87E+01
9.87E+01
9.87E+01
9.87E+01
9.87E+01
9.88E+01
9.88E+01
9.88E+01
9.88E+01
9.88E+01
9.88E+01
9.88E+01
9.88E+01
9.88E+01
9.88E+01
9.88E+01
9.88E+01
9.88E+01
9.88E+01
9.88E+01
9.89E+01
9.89E+01
9.89E+01
9.89E+01
9.88E+01
9.88E+01
9.88E+01
9.88E+01
9.88E+01
9.88E+01
9.88E+01
9.88E+01
9.88E+01
9.88E+01
9.87E+01
9.87E+01
9.86E+01
9.86E+01
9.86E+01
9.86E+01
9.86E+01

9.86E+01
9.86E+01
9.86E+01
9.86E+01
9.86E+01
9.85E+01
9.85E+01
9.85E+01
9.85E+01
9.85E+01
9.84E+01
9.84E+01
9.84E+01
9.84E+01
9.84E+01
9.83E+01
9.83E+01
9.83E+01
9.83E+01
9.83E+01
9.82E+01
9.82E+01
9.82E+01
9.81E+01
9.81E+01
9.81E+01
9.80E+01
9.80E+01
9.79E+01
9.79E+01
9.79E+01
9.78E+01
9.78E+01
9.78E+01
9.77E+01
9.77E+01
9.77E+01
9.77E+01
9.76E+01
9.76E+01
9.76E+01
9.76E+01
9.76E+01
9.76E+01
9.76E+01
9.76E+01
9.76E+01

9.76E+01
9.75E+01
9.75E+01
9.75E+01
9.75E+01
9.76E+01
9.76E+01
9.76E+01
9.76E+01
9.76E+01
9.76E+01
9.76E+01
9.76E+01
9.76E+01
9.76E+01
9.76E+01
9.76E+01
9.77E+01
9.77E+01
9.77E+01
9.77E+01
9.77E+01
9.78E+01
9.78E+01
9.78E+01
9.78E+01
9.78E+01
9.78E+01
9.78E+01
9.78E+01
9.78E+01
9.78E+01
9.78E+01
9.78E+01
9.78E+01
9.78E+01
9.78E+01
9.78E+01
9.78E+01
9.78E+01
9.78E+01
9.78E+01
9.78E+01
9.77E+01
9.77E+01
9.77E+01
9.77E+01

9.77E+01
9.77E+01
9.77E+01
9.77E+01
9.77E+01
9.77E+01
9.77E+01
9.77E+01
9.77E+01
9.77E+01
9.77E+01
9.77E+01
9.77E+01
9.77E+01
9.77E+01
9.77E+01
9.76E+01
9.76E+01
9.76E+01
9.76E+01
9.76E+01
9.76E+01
9.76E+01
9.76E+01
9.76E+01
9.76E+01
9.76E+01
9.76E+01
9.76E+01
9.76E+01
9.76E+01
9.76E+01
9.76E+01
9.76E+01
9.76E+01
9.76E+01
9.76E+01
9.76E+01
9.76E+01
9.76E+01
9.76E+01
9.76E+01
9.76E+01
9.76E+01
9.76E+01
9.76E+01
9.76E+01

9.76E+01
9.76E+01
9.76E+01
9.76E+01
9.76E+01
9.76E+01
9.76E+01
9.76E+01
9.76E+01
9.76E+01
9.75E+01
9.75E+01
9.75E+01
9.75E+01
9.75E+01
9.75E+01
9.76E+01
9.76E+01
9.76E+01
9.76E+01
9.76E+01
9.76E+01
9.75E+01
9.75E+01
9.75E+01
9.75E+01
9.75E+01
9.75E+01
9.75E+01
9.75E+01
9.75E+01
9.75E+01
9.75E+01
9.75E+01
9.75E+01
9.75E+01
9.75E+01
9.76E+01
9.76E+01
9.76E+01
9.76E+01
9.76E+01
9.76E+01
9.76E+01
9.76E+01
9.76E+01
9.76E+01

9.76E+01
9.76E+01
9.76E+01
9.76E+01
9.76E+01
9.76E+01
9.76E+01
9.76E+01
9.77E+01
9.77E+01
9.77E+01
9.76E+01
9.76E+01
9.76E+01
9.76E+01
9.76E+01
9.76E+01
9.76E+01
9.76E+01
9.76E+01
9.76E+01
9.76E+01
9.76E+01
9.76E+01
9.76E+01
9.76E+01
9.76E+01
9.76E+01
9.76E+01
9.76E+01
9.76E+01
9.76E+01
9.77E+01
9.77E+01
9.77E+01
9.77E+01
9.77E+01
9.77E+01
9.77E+01
9.77E+01
9.77E+01
9.77E+01
9.77E+01
9.77E+01
9.77E+01
9.77E+01
9.77E+01

9.77E+01
9.77E+01
9.77E+01
9.77E+01
9.78E+01
9.78E+01
9.78E+01
9.78E+01
9.78E+01
9.78E+01
9.78E+01
9.78E+01
9.78E+01
9.78E+01
9.78E+01
9.78E+01
9.78E+01
9.78E+01
9.78E+01
9.78E+01
9.78E+01
9.78E+01
9.79E+01
9.79E+01
9.79E+01
9.79E+01
9.79E+01
9.79E+01
9.79E+01
9.79E+01
9.79E+01
9.79E+01
9.79E+01
9.79E+01
9.79E+01
9.79E+01
9.79E+01
9.79E+01
9.79E+01
9.79E+01
9.79E+01
9.79E+01
9.79E+01
9.80E+01
9.80E+01
9.80E+01
9.80E+01

9.80E+01
9.80E+01
9.80E+01
9.80E+01
9.80E+01
9.80E+01
9.80E+01
9.80E+01
9.80E+01
9.80E+01
9.80E+01
9.80E+01
9.80E+01
9.80E+01
9.80E+01
9.81E+01
9.81E+01
9.81E+01
9.81E+01
9.81E+01
9.80E+01
9.80E+01
9.80E+01
9.80E+01
9.80E+01
9.81E+01
9.81E+01
9.81E+01
9.81E+01
9.81E+01
9.81E+01
9.81E+01
9.81E+01
9.81E+01
9.81E+01
9.80E+01
9.80E+01
9.80E+01
9.81E+01
9.81E+01
9.81E+01
9.81E+01
9.81E+01
9.81E+01
9.81E+01
9.80E+01
9.80E+01

9.80E+01
9.80E+01
9.80E+01
9.80E+01
9.80E+01
9.80E+01
9.80E+01
9.80E+01
9.80E+01
9.80E+01
9.79E+01
9.79E+01
9.79E+01
9.79E+01
9.79E+01
9.79E+01
9.79E+01
9.79E+01
9.79E+01
9.79E+01
9.79E+01
9.79E+01
9.79E+01
9.79E+01
9.79E+01
9.78E+01
9.78E+01
9.78E+01
9.78E+01
9.78E+01
9.79E+01
9.79E+01
9.79E+01
9.79E+01
9.79E+01
9.79E+01
9.79E+01
9.79E+01
9.79E+01
9.79E+01
9.79E+01
9.79E+01
9.79E+01
9.79E+01
9.79E+01
9.79E+01
9.79E+01

9.79E+01
9.79E+01
9.78E+01
9.78E+01
9.78E+01
9.78E+01
9.78E+01
9.78E+01
9.78E+01
9.78E+01
9.78E+01
9.78E+01
9.78E+01
9.78E+01
9.78E+01
9.77E+01
9.77E+01
9.77E+01
9.76E+01
9.76E+01
9.75E+01
9.75E+01
9.75E+01
9.74E+01
9.74E+01
9.75E+01
9.75E+01
9.75E+01
9.75E+01
9.76E+01
9.76E+01
9.76E+01
9.76E+01
9.76E+01
9.75E+01
9.75E+01
9.75E+01
9.75E+01
9.75E+01
9.75E+01
9.74E+01
9.74E+01
9.74E+01
9.74E+01
9.74E+01
9.74E+01
9.74E+01

9.74E+01
9.74E+01
9.74E+01
9.74E+01
9.74E+01
9.74E+01
9.74E+01
9.73E+01
9.73E+01
9.73E+01
9.73E+01
9.73E+01
9.73E+01
9.73E+01
9.73E+01
9.73E+01
9.73E+01
9.73E+01
9.73E+01
9.73E+01
9.73E+01
9.72E+01
9.72E+01
9.72E+01
9.72E+01
9.72E+01
9.72E+01
9.72E+01
9.73E+01
9.73E+01
9.74E+01
9.74E+01
9.74E+01
9.74E+01
9.74E+01
9.74E+01
9.74E+01
9.74E+01
9.74E+01
9.74E+01
9.74E+01
9.74E+01
9.74E+01
9.73E+01
9.73E+01
9.73E+01
9.73E+01

9.73E+01
9.73E+01
9.73E+01
9.74E+01
9.74E+01
9.74E+01
9.74E+01
9.75E+01
9.75E+01
9.75E+01
9.75E+01
9.75E+01
9.75E+01
9.76E+01
9.76E+01
9.76E+01
9.76E+01
9.76E+01
9.77E+01
9.77E+01
9.77E+01
9.77E+01
9.77E+01
9.77E+01
9.76E+01
9.76E+01
9.76E+01
9.76E+01
9.76E+01
9.76E+01
9.76E+01
9.76E+01
9.77E+01
9.77E+01
9.77E+01
9.77E+01
9.78E+01
9.78E+01
9.78E+01
9.77E+01
9.77E+01
9.77E+01
9.77E+01
9.76E+01
9.76E+01
9.76E+01
9.77E+01

9.77E+01
9.77E+01
9.77E+01
9.76E+01
9.76E+01
9.76E+01
9.77E+01
9.77E+01
9.78E+01
9.78E+01
9.78E+01
9.79E+01
9.79E+01
9.79E+01
9.78E+01
9.78E+01
9.78E+01
9.77E+01
9.77E+01
9.77E+01
9.77E+01
9.77E+01
9.78E+01
9.78E+01
9.78E+01
9.78E+01
9.78E+01
9.78E+01
9.77E+01
9.77E+01
9.77E+01
9.77E+01
9.77E+01
9.77E+01
9.77E+01
9.77E+01
9.77E+01
9.77E+01
9.76E+01
9.76E+01
9.76E+01
9.75E+01
9.75E+01
9.75E+01
9.74E+01
9.74E+01
9.74E+01

9.74E+01
9.74E+01
9.74E+01
9.74E+01
9.74E+01
9.74E+01
9.74E+01
9.74E+01
9.74E+01
9.74E+01
9.74E+01
9.74E+01
9.74E+01
9.74E+01
9.74E+01
9.74E+01
9.74E+01
9.74E+01
9.73E+01
9.73E+01
9.72E+01
9.71E+01
9.71E+01
9.70E+01
9.70E+01
9.69E+01
9.69E+01
9.69E+01
9.68E+01
9.68E+01
9.67E+01
9.67E+01
9.66E+01
9.65E+01
9.65E+01
9.64E+01
9.63E+01
9.62E+01
9.62E+01
9.61E+01
9.61E+01
9.61E+01
9.60E+01
9.60E+01
9.60E+01
9.59E+01
9.59E+01

9.58E+01
9.58E+01
9.57E+01
9.57E+01
9.56E+01
9.55E+01
9.54E+01
9.54E+01
9.53E+01
9.52E+01
9.51E+01
9.50E+01
9.49E+01
9.48E+01
9.48E+01
9.47E+01
9.47E+01
9.46E+01
9.46E+01
9.46E+01
9.46E+01
9.45E+01
9.45E+01
9.45E+01
9.45E+01
9.44E+01
9.44E+01
9.44E+01
9.44E+01
9.44E+01
9.44E+01
9.45E+01
9.46E+01
9.49E+01
9.52E+01
9.55E+01
9.58E+01
9.60E+01
9.62E+01
9.64E+01
9.65E+01
9.67E+01
9.68E+01
9.69E+01
9.70E+01
9.71E+01
9.72E+01

9.73E+01
9.74E+01
9.75E+01
9.76E+01
9.76E+01
9.77E+01
9.78E+01
9.78E+01
9.78E+01
9.78E+01
9.78E+01
9.78E+01
9.78E+01
9.78E+01
9.77E+01
9.76E+01
9.76E+01
9.75E+01
9.75E+01
9.75E+01
9.76E+01
9.77E+01
9.77E+01
9.78E+01
9.78E+01
9.79E+01
9.79E+01
9.80E+01
9.80E+01
9.80E+01
9.80E+01
9.80E+01
9.80E+01
9.80E+01
9.80E+01
9.80E+01
9.80E+01
9.80E+01
9.80E+01
9.80E+01
9.80E+01
9.80E+01
9.80E+01
9.80E+01
9.80E+01
9.79E+01
9.79E+01

9.78E+01
9.78E+01
9.77E+01
9.76E+01
9.76E+01
9.75E+01
9.76E+01
9.76E+01
9.77E+01
9.78E+01
9.78E+01
9.79E+01
9.79E+01
9.79E+01
9.79E+01
9.79E+01
9.78E+01
9.78E+01
9.78E+01
9.77E+01
9.77E+01
9.77E+01
9.77E+01
9.77E+01
9.77E+01
9.78E+01
9.78E+01
9.78E+01
9.78E+01
9.78E+01
9.78E+01
9.78E+01
9.78E+01
9.78E+01
9.77E+01
9.77E+01
9.77E+01
9.76E+01
9.75E+01
9.74E+01
9.72E+01
9.71E+01
9.71E+01
9.71E+01
9.71E+01
9.72E+01
9.73E+01

9.73E+01
9.73E+01
9.74E+01
9.74E+01
9.75E+01
9.75E+01
9.75E+01
9.75E+01
9.75E+01
9.75E+01
9.75E+01
9.75E+01
9.74E+01
9.74E+01
9.73E+01
9.73E+01
9.73E+01
9.73E+01
9.73E+01
9.73E+01
9.73E+01
9.72E+01
9.72E+01
9.71E+01
9.71E+01
9.71E+01
9.71E+01
9.72E+01
9.72E+01
9.73E+01
9.73E+01
9.73E+01
9.73E+01
9.73E+01
9.72E+01
9.72E+01
9.72E+01
9.72E+01
9.72E+01
9.72E+01
9.72E+01
9.73E+01
9.73E+01
9.73E+01
9.73E+01
9.72E+01
9.72E+01

9.71E+01
9.70E+01
9.70E+01
9.70E+01
9.70E+01
9.70E+01
9.71E+01
9.71E+01
9.71E+01
9.71E+01
9.70E+01
9.70E+01
9.69E+01
9.68E+01
9.66E+01
9.64E+01
9.63E+01
9.62E+01
9.63E+01
9.63E+01
9.64E+01
9.65E+01
9.66E+01
9.66E+01
9.66E+01
9.67E+01
9.67E+01
9.68E+01
9.69E+01
9.70E+01
9.70E+01
9.70E+01
9.71E+01
9.70E+01
9.70E+01
9.70E+01
9.70E+01
9.70E+01
9.70E+01
9.70E+01
9.70E+01
9.70E+01
9.70E+01
9.70E+01
9.69E+01
9.69E+01
9.69E+01

9.69E+01
9.68E+01
9.68E+01
9.68E+01
9.68E+01
9.68E+01
9.67E+01
9.67E+01
9.66E+01
9.66E+01
9.67E+01
9.68E+01
9.68E+01
9.69E+01
9.69E+01
9.70E+01
9.70E+01
9.70E+01
9.70E+01
9.69E+01
9.69E+01
9.69E+01
9.69E+01
9.69E+01
9.69E+01
9.69E+01
9.69E+01
9.69E+01
9.69E+01
9.68E+01
9.68E+01
9.68E+01
9.69E+01
9.69E+01
9.69E+01
9.69E+01
9.69E+01
9.69E+01
9.69E+01
9.69E+01
9.69E+01
9.68E+01
9.68E+01
9.68E+01
9.67E+01
9.67E+01
9.68E+01

9.68E+01
9.68E+01
9.69E+01
9.69E+01
9.69E+01
9.69E+01
9.69E+01
9.69E+01
9.69E+01
9.69E+01
9.69E+01
9.69E+01
9.69E+01
9.69E+01
9.69E+01
9.69E+01
9.69E+01
9.69E+01
9.69E+01
9.69E+01
9.69E+01
9.69E+01
9.69E+01
9.69E+01
9.68E+01
9.68E+01
9.68E+01
9.68E+01
9.68E+01
9.68E+01
9.67E+01
9.67E+01
9.67E+01
9.67E+01
9.67E+01
9.66E+01
9.66E+01
9.66E+01
9.66E+01
9.66E+01
9.66E+01
9.66E+01
9.66E+01
9.66E+01
9.66E+01
9.66E+01
9.65E+01

9.65E+01
9.65E+01
9.64E+01
9.64E+01
9.64E+01
9.64E+01
9.64E+01
9.64E+01
9.63E+01
9.63E+01
9.63E+01
9.63E+01
9.62E+01
9.62E+01
9.61E+01
9.61E+01
9.61E+01
9.61E+01
9.60E+01
9.60E+01
9.60E+01
9.60E+01
9.60E+01
9.60E+01
9.60E+01
9.60E+01
9.59E+01
9.59E+01
9.59E+01
9.59E+01
9.58E+01
9.58E+01
9.57E+01
9.57E+01
9.56E+01
9.56E+01
9.56E+01
9.56E+01
9.56E+01
9.56E+01
9.56E+01
9.56E+01
9.55E+01
9.55E+01
9.54E+01
9.54E+01
9.53E+01

9.52E+01
9.52E+01
9.52E+01
9.52E+01
9.52E+01
9.52E+01
9.52E+01
9.52E+01
9.52E+01
9.52E+01
9.52E+01
9.52E+01
9.52E+01
9.52E+01
9.52E+01
9.52E+01
9.52E+01
9.52E+01
9.52E+01
9.52E+01
9.51E+01
9.51E+01
9.51E+01
9.50E+01
9.49E+01
9.49E+01
9.48E+01
9.48E+01
9.48E+01
9.48E+01
9.48E+01
9.49E+01
9.49E+01
9.49E+01
9.50E+01
9.50E+01
9.50E+01
9.51E+01
9.51E+01
9.51E+01
9.51E+01
9.51E+01
9.50E+01
9.50E+01
9.49E+01
9.49E+01
9.48E+01

9.47E+01
9.46E+01
9.45E+01
9.45E+01
9.45E+01
9.46E+01
9.46E+01
9.47E+01
9.47E+01
9.47E+01
9.47E+01
9.47E+01
9.46E+01
9.45E+01
9.45E+01
9.44E+01
9.44E+01
9.44E+01
9.45E+01
9.47E+01
9.48E+01
9.48E+01
9.49E+01
9.50E+01
9.51E+01
9.51E+01
9.52E+01
9.52E+01
9.52E+01
9.52E+01
9.52E+01
9.53E+01
9.53E+01
9.53E+01
9.53E+01
9.53E+01
9.53E+01
9.54E+01
9.54E+01
9.55E+01
9.55E+01
9.55E+01
9.55E+01
9.55E+01
9.54E+01
9.54E+01
9.54E+01

9.53E+01
9.53E+01
9.53E+01
9.53E+01
9.54E+01
9.54E+01
9.55E+01
9.55E+01
9.56E+01
9.56E+01
9.57E+01
9.57E+01
9.57E+01
9.58E+01
9.58E+01
9.59E+01
9.59E+01
9.60E+01
9.60E+01
9.60E+01
9.60E+01
9.60E+01
9.60E+01
9.60E+01
9.60E+01
9.60E+01
9.60E+01
9.60E+01
9.60E+01
9.59E+01
9.59E+01
9.58E+01
9.58E+01
9.58E+01
9.58E+01
9.59E+01
9.59E+01
9.60E+01
9.61E+01
9.61E+01
9.62E+01
9.62E+01
9.63E+01
9.64E+01
9.64E+01
9.65E+01
9.66E+01

9.66E+01
9.66E+01
9.67E+01
9.66E+01
9.66E+01
9.66E+01
9.66E+01
9.65E+01
9.65E+01
9.65E+01
9.64E+01
9.64E+01
9.64E+01
9.65E+01
9.65E+01
9.65E+01
9.66E+01
9.66E+01
9.67E+01
9.67E+01
9.67E+01
9.67E+01
9.67E+01
9.66E+01
9.66E+01
9.66E+01
9.66E+01
9.66E+01
9.65E+01
9.65E+01
9.66E+01
9.66E+01
9.66E+01
9.67E+01
9.67E+01
9.68E+01
9.68E+01
9.68E+01
9.68E+01
9.68E+01
9.67E+01
9.67E+01
9.66E+01
9.66E+01
9.65E+01
9.65E+01
9.64E+01

9.63E+01
9.62E+01
9.62E+01
9.61E+01
9.60E+01
9.60E+01
9.59E+01
9.59E+01
9.58E+01
9.58E+01
9.57E+01
9.57E+01
9.57E+01
9.57E+01
9.57E+01
9.57E+01
9.56E+01
9.56E+01
9.56E+01
9.55E+01
9.55E+01
9.55E+01
9.55E+01
9.54E+01
9.54E+01
9.54E+01
9.54E+01
9.53E+01
9.53E+01
9.52E+01
9.52E+01
9.51E+01
9.51E+01
9.50E+01
9.50E+01
9.50E+01
9.50E+01
9.50E+01
9.50E+01
9.50E+01
9.50E+01
9.50E+01
9.51E+01
9.51E+01
9.52E+01
9.53E+01
9.54E+01

9.55E+01
9.55E+01
9.56E+01
9.57E+01
9.57E+01
9.58E+01
9.59E+01
9.60E+01
9.61E+01
9.62E+01
9.62E+01
9.63E+01
9.64E+01
9.64E+01
9.64E+01
9.65E+01
9.65E+01
9.66E+01
9.67E+01
9.68E+01
9.68E+01
9.69E+01
9.69E+01
9.70E+01
9.70E+01
9.70E+01
9.70E+01
9.71E+01
9.71E+01
9.72E+01
9.73E+01
9.74E+01
9.74E+01
9.75E+01
9.76E+01
9.76E+01
9.77E+01
9.77E+01
9.78E+01
9.78E+01
9.78E+01
9.78E+01
9.78E+01
9.78E+01
9.78E+01
9.78E+01
9.78E+01

9.78E+01
9.78E+01
9.78E+01
9.78E+01
9.78E+01
9.78E+01
9.78E+01
9.78E+01
9.78E+01
9.79E+01
9.79E+01
9.80E+01
9.80E+01
9.80E+01
9.80E+01
9.80E+01
9.79E+01
9.79E+01
9.79E+01
9.79E+01
9.78E+01
9.78E+01
9.77E+01
9.77E+01
9.76E+01
9.75E+01
9.75E+01
9.74E+01
9.75E+01
9.75E+01
9.76E+01
9.77E+01
9.77E+01
9.78E+01
9.79E+01
9.79E+01
9.80E+01
9.80E+01
9.81E+01
9.81E+01
9.81E+01
9.81E+01
9.81E+01
9.80E+01
9.80E+01
9.80E+01
9.80E+01

9.80E+01
9.80E+01
9.80E+01
9.80E+01
9.80E+01
9.80E+01
9.80E+01
9.80E+01
9.79E+01
9.79E+01
9.79E+01
9.79E+01
9.79E+01
9.79E+01
9.79E+01
9.79E+01
9.79E+01
9.79E+01
9.79E+01
9.79E+01
9.79E+01
9.78E+01
9.78E+01
9.78E+01
9.78E+01
9.78E+01
9.78E+01
9.79E+01
9.79E+01
9.79E+01
9.80E+01
9.80E+01
9.80E+01
9.80E+01
9.80E+01
9.79E+01
9.79E+01
9.79E+01
9.79E+01
9.79E+01
9.79E+01
9.79E+01
9.79E+01
9.79E+01
9.80E+01
9.80E+01
9.80E+01

9.81E+01
9.81E+01
9.81E+01
9.81E+01
9.81E+01
9.81E+01
9.81E+01
9.81E+01
9.81E+01
9.81E+01
9.81E+01
9.81E+01
9.81E+01
9.82E+01
9.82E+01
9.82E+01
9.82E+01
9.81E+01
9.81E+01
9.81E+01
9.81E+01
9.80E+01
9.80E+01
9.80E+01
9.80E+01
9.80E+01
9.79E+01
9.79E+01
9.79E+01
9.79E+01
9.79E+01
9.80E+01
9.80E+01
9.80E+01
9.80E+01
9.80E+01
9.80E+01
9.80E+01
9.80E+01
9.81E+01
9.81E+01
9.81E+01
9.81E+01
9.81E+01
9.80E+01
9.80E+01
9.79E+01

9.79E+01
9.79E+01
9.78E+01
9.78E+01
9.78E+01
9.77E+01
9.77E+01
9.77E+01
9.78E+01
9.78E+01
9.79E+01
9.79E+01
9.80E+01
9.80E+01
9.81E+01
9.81E+01
9.81E+01
9.81E+01
9.80E+01
9.80E+01
9.80E+01
9.80E+01
9.80E+01
9.80E+01
9.80E+01
9.80E+01
9.81E+01
9.81E+01
9.81E+01
9.81E+01
9.80E+01
9.80E+01
9.79E+01
9.78E+01
9.78E+01
9.77E+01
9.76E+01
9.76E+01
9.75E+01
9.75E+01
9.75E+01
9.75E+01
9.75E+01
9.76E+01
9.76E+01
9.77E+01
9.78E+01

9.78E+01
9.79E+01
9.79E+01
9.79E+01
9.79E+01
9.78E+01
9.78E+01
9.78E+01
9.78E+01
9.78E+01
9.78E+01
9.79E+01
9.79E+01
9.80E+01
9.80E+01
9.81E+01
9.81E+01
9.81E+01
9.81E+01
9.80E+01
9.80E+01
9.80E+01
9.79E+01
9.79E+01
9.79E+01
9.79E+01
9.78E+01
9.79E+01
9.79E+01
9.79E+01
9.79E+01
9.79E+01
9.79E+01
9.79E+01
9.79E+01
9.78E+01
9.78E+01
9.77E+01
9.76E+01
9.76E+01
9.76E+01
9.75E+01
9.76E+01
9.76E+01
9.76E+01
9.76E+01
9.76E+01

9.77E+01
9.77E+01
9.78E+01
9.79E+01
9.79E+01
9.80E+01
9.80E+01
9.80E+01
9.79E+01
9.79E+01
9.78E+01
9.77E+01
9.77E+01
9.76E+01
9.76E+01
9.76E+01
9.77E+01
9.77E+01
9.77E+01
9.78E+01
9.78E+01
9.79E+01
9.79E+01
9.80E+01
9.80E+01
9.80E+01
9.80E+01
9.80E+01
9.80E+01
9.79E+01
9.78E+01
9.77E+01
9.76E+01
9.76E+01
9.75E+01
9.75E+01
9.75E+01
9.76E+01
9.77E+01
9.78E+01
9.79E+01
9.79E+01
9.80E+01
9.80E+01
9.79E+01
9.79E+01
9.79E+01

9.78E+01
9.78E+01
9.78E+01
9.78E+01
9.78E+01
9.78E+01
9.78E+01
9.77E+01
9.77E+01
9.77E+01
9.77E+01
9.78E+01
9.78E+01
9.79E+01
9.79E+01
9.79E+01
9.79E+01
9.79E+01
9.78E+01
9.78E+01
9.78E+01
9.77E+01
9.77E+01
9.78E+01
9.78E+01
9.78E+01
9.79E+01
9.79E+01
9.80E+01
9.80E+01
9.80E+01
9.80E+01
9.80E+01
9.80E+01
9.80E+01
9.80E+01
9.80E+01
9.81E+01
9.81E+01
9.82E+01
9.82E+01
9.83E+01
9.82E+01
9.81E+01
9.80E+01
9.79E+01
9.79E+01

9.78E+01
9.78E+01
9.79E+01
9.79E+01
9.80E+01
9.80E+01
9.79E+01
9.78E+01
9.77E+01
9.76E+01
9.76E+01
9.75E+01
9.75E+01
9.76E+01
9.77E+01
9.78E+01
9.79E+01
9.80E+01
9.81E+01
9.81E+01
9.81E+01
9.81E+01
9.81E+01
9.80E+01
9.79E+01
9.78E+01
9.78E+01
9.77E+01
9.77E+01
9.78E+01
9.78E+01
9.79E+01
9.80E+01
9.80E+01
9.81E+01
9.81E+01
9.82E+01
9.82E+01
9.82E+01
9.81E+01
9.81E+01
9.80E+01
9.79E+01
9.78E+01
9.77E+01
9.76E+01
9.75E+01

9.74E+01
9.73E+01
9.72E+01
9.72E+01
9.72E+01
9.72E+01
9.73E+01
9.74E+01
9.75E+01
9.76E+01
9.76E+01
9.76E+01
9.76E+01
9.76E+01
9.76E+01
9.77E+01
9.77E+01
9.77E+01
9.77E+01
9.77E+01
9.77E+01
9.77E+01
9.77E+01
9.77E+01
9.78E+01
9.79E+01
9.80E+01
9.82E+01
9.83E+01
9.84E+01
9.85E+01
9.85E+01
9.85E+01
9.85E+01
9.86E+01
9.87E+01
9.88E+01
9.90E+01
9.93E+01
9.94E+01
9.95E+01
9.94E+01
9.92E+01
9.88E+01
9.84E+01
9.79E+01
9.74E+01

9.70E+01
9.68E+01
9.67E+01
9.67E+01
9.69E+01
9.72E+01
9.76E+01
9.81E+01
9.86E+01
9.90E+01
9.94E+01
9.97E+01
9.98E+01
9.99E+01
9.99E+01
9.98E+01
9.96E+01
9.93E+01
9.91E+01
9.88E+01
9.86E+01
9.84E+01
9.81E+01
9.79E+01
9.77E+01
9.75E+01
9.73E+01
9.71E+01
9.71E+01
9.71E+01
9.71E+01
9.72E+01
9.74E+01
9.76E+01
9.79E+01
9.81E+01
9.83E+01
9.85E+01
9.85E+01
9.85E+01
9.84E+01
9.82E+01
9.79E+01
9.77E+01
9.74E+01
9.71E+01
9.68E+01

9.65E+01
9.62E+01
9.60E+01
9.57E+01
9.55E+01
9.55E+01
9.55E+01
9.56E+01
9.58E+01
9.60E+01
9.61E+01
9.62E+01
9.62E+01
9.61E+01
9.60E+01
9.59E+01
9.58E+01
9.57E+01
9.57E+01
9.58E+01
9.59E+01
9.61E+01
9.63E+01
9.66E+01
9.69E+01
9.72E+01
9.75E+01
9.76E+01
9.77E+01
9.77E+01
9.78E+01
9.78E+01
9.78E+01
9.78E+01
9.78E+01
9.76E+01
9.74E+01
9.72E+01
9.69E+01
9.67E+01
9.66E+01
9.67E+01
9.70E+01
9.74E+01
9.79E+01
9.83E+01
9.86E+01

9.87E+01
9.86E+01
9.84E+01
9.81E+01
9.79E+01
9.77E+01
9.78E+01
9.80E+01
9.84E+01
9.89E+01
9.94E+01
9.98E+01
1.00E+02
1.00E+02
9.98E+01
9.94E+01
9.89E+01
9.83E+01
9.77E+01
9.71E+01
9.66E+01
9.62E+01
9.60E+01
9.58E+01
9.58E+01
9.58E+01
9.58E+01
9.58E+01
9.58E+01
9.58E+01
9.59E+01
9.61E+01
9.63E+01
9.66E+01
9.69E+01
9.72E+01
9.74E+01
9.74E+01
9.74E+01
9.74E+01
9.73E+01
9.73E+01
9.72E+01
9.72E+01
9.71E+01
9.71E+01
9.70E+01

9.70E+01
9.69E+01
9.69E+01
9.69E+01
9.70E+01
9.71E+01
9.72E+01
9.72E+01
9.73E+01
9.72E+01
9.72E+01
9.70E+01
9.69E+01
9.68E+01
9.68E+01
9.68E+01
9.69E+01
9.70E+01
9.72E+01
9.72E+01
9.73E+01
9.73E+01
9.73E+01
9.73E+01
9.73E+01
9.74E+01
9.74E+01
9.74E+01
9.73E+01
9.71E+01
9.69E+01
9.66E+01
9.64E+01
9.63E+01
9.62E+01
9.62E+01
9.63E+01
9.65E+01
9.67E+01
9.69E+01
9.71E+01
9.73E+01
9.75E+01
9.77E+01
9.77E+01
9.78E+01
9.78E+01

9.78E+01
9.78E+01
9.78E+01
9.78E+01
9.78E+01
9.77E+01
9.75E+01
9.73E+01
9.71E+01
9.69E+01
9.67E+01
9.67E+01
9.67E+01
9.68E+01
9.69E+01
9.69E+01
9.70E+01
9.70E+01
9.70E+01
9.71E+01
9.71E+01
9.71E+01
9.72E+01
9.73E+01
9.74E+01
9.76E+01
9.79E+01
9.81E+01
9.83E+01
9.84E+01
9.85E+01
9.84E+01
9.83E+01
9.80E+01
9.77E+01
9.75E+01
9.72E+01
9.70E+01
9.69E+01
9.69E+01
9.69E+01
9.69E+01
9.70E+01
9.70E+01
9.71E+01
9.72E+01
9.73E+01

9.74E+01
9.75E+01
9.76E+01
9.77E+01
9.78E+01
9.78E+01
9.78E+01
9.77E+01
9.76E+01
9.75E+01
9.75E+01
9.74E+01
9.74E+01
9.74E+01
9.73E+01
9.73E+01
9.72E+01
9.71E+01
9.71E+01
9.71E+01
9.71E+01
9.72E+01
9.72E+01
9.73E+01
9.74E+01
9.74E+01
9.74E+01
9.74E+01
9.74E+01
9.73E+01
9.73E+01
9.73E+01
9.73E+01
9.73E+01
9.72E+01
9.72E+01
9.71E+01
9.70E+01
9.69E+01
9.68E+01
9.67E+01
9.67E+01
9.67E+01
9.67E+01
9.67E+01
9.67E+01
9.67E+01

9.67E+01
9.66E+01
9.66E+01
9.66E+01
9.65E+01
9.65E+01
9.66E+01
9.66E+01
9.67E+01
9.68E+01
9.69E+01
9.70E+01
9.71E+01
9.72E+01
9.73E+01
9.74E+01
9.74E+01
9.74E+01
9.73E+01
9.73E+01
9.72E+01
9.72E+01
9.72E+01
9.73E+01
9.73E+01
9.74E+01
9.75E+01
9.75E+01
9.76E+01
9.76E+01
9.77E+01
9.77E+01
9.77E+01
9.76E+01
9.76E+01
9.75E+01
9.74E+01
9.72E+01
9.71E+01
9.69E+01
9.68E+01
9.66E+01
9.66E+01
9.65E+01
9.65E+01
9.66E+01
9.66E+01

9.67E+01
9.67E+01
9.67E+01
9.66E+01
9.65E+01
9.64E+01
9.63E+01
9.62E+01
9.61E+01
9.60E+01
9.61E+01
9.62E+01
9.63E+01
9.65E+01
9.68E+01
9.70E+01
9.72E+01
9.74E+01
9.76E+01
9.76E+01
9.77E+01
9.77E+01
9.77E+01
9.77E+01
9.76E+01
9.75E+01
9.74E+01
9.72E+01
9.71E+01
9.69E+01
9.69E+01
9.68E+01
9.69E+01
9.69E+01
9.70E+01
9.71E+01
9.73E+01
9.74E+01
9.76E+01
9.77E+01
9.79E+01
9.80E+01
9.80E+01
9.80E+01
9.79E+01
9.77E+01
9.76E+01

9.74E+01
9.72E+01
9.71E+01
9.70E+01
9.69E+01
9.68E+01
9.68E+01
9.68E+01
9.68E+01
9.70E+01
9.71E+01
9.74E+01
9.78E+01
9.81E+01
9.85E+01
9.87E+01
9.88E+01
9.87E+01
9.85E+01
9.82E+01
9.78E+01
9.75E+01
9.73E+01
9.72E+01
9.73E+01
9.75E+01
9.78E+01
9.81E+01
9.84E+01
9.86E+01
9.86E+01
9.85E+01
9.82E+01
9.78E+01
9.74E+01
9.70E+01
9.69E+01
9.69E+01
9.71E+01
9.74E+01
9.76E+01
9.78E+01
9.78E+01
9.77E+01
9.76E+01
9.75E+01
9.75E+01

9.76E+01
9.78E+01
9.81E+01
9.83E+01
9.85E+01
9.85E+01
9.85E+01
9.84E+01
9.83E+01
9.82E+01
9.82E+01
9.81E+01
9.79E+01
9.78E+01
9.76E+01
9.75E+01
9.73E+01
9.73E+01
9.73E+01
9.73E+01
9.74E+01
9.75E+01
9.76E+01
9.77E+01
9.77E+01
9.77E+01
9.76E+01
9.75E+01
9.74E+01
9.72E+01
9.71E+01
9.70E+01
9.70E+01
9.70E+01
9.70E+01
9.71E+01
9.71E+01
9.70E+01
9.68E+01
9.66E+01
9.64E+01
9.61E+01
9.60E+01
9.58E+01
9.58E+01
9.59E+01
9.60E+01

9.62E+01
9.65E+01
9.68E+01
9.70E+01
9.71E+01
9.72E+01
9.72E+01
9.72E+01
9.72E+01
9.72E+01
9.74E+01
9.76E+01
9.78E+01
9.80E+01
9.82E+01
9.82E+01
9.82E+01
9.82E+01
9.80E+01
9.79E+01
9.77E+01
9.75E+01
9.74E+01
9.72E+01
9.71E+01
9.69E+01
9.68E+01
9.67E+01
9.67E+01
9.66E+01
9.66E+01
9.66E+01
9.65E+01
9.65E+01
9.65E+01
9.65E+01
9.65E+01
9.66E+01
9.66E+01
9.67E+01
9.67E+01
9.67E+01
9.68E+01
9.68E+01
9.68E+01
9.69E+01
9.70E+01

9.70E+01
9.70E+01
9.69E+01
9.68E+01
9.67E+01
9.65E+01
9.64E+01
9.63E+01
9.62E+01
9.62E+01
9.62E+01
9.62E+01
9.63E+01
9.64E+01
9.65E+01
9.66E+01
9.67E+01
9.68E+01
9.69E+01
9.70E+01
9.70E+01
9.70E+01
9.69E+01
9.67E+01
9.65E+01
9.62E+01
9.60E+01
9.58E+01
9.57E+01
9.58E+01
9.59E+01
9.61E+01
9.63E+01
9.64E+01
9.66E+01
9.67E+01
9.67E+01
9.67E+01
9.66E+01
9.65E+01
9.64E+01
9.62E+01
9.61E+01
9.60E+01
9.59E+01
9.58E+01
9.57E+01

9.57E+01
9.56E+01
9.55E+01
9.54E+01
9.52E+01
9.50E+01
9.47E+01
9.44E+01
9.41E+01
9.38E+01
9.35E+01
9.32E+01
9.30E+01
9.29E+01
9.28E+01
9.29E+01
9.30E+01
9.31E+01
9.33E+01
9.34E+01
9.36E+01
9.37E+01
9.39E+01
9.40E+01
9.42E+01
9.43E+01
9.45E+01
9.47E+01
9.49E+01
9.51E+01
9.53E+01
9.55E+01
9.56E+01
9.58E+01
9.59E+01
9.60E+01
9.60E+01
9.61E+01
9.61E+01
9.62E+01
9.63E+01
9.64E+01
9.65E+01
9.65E+01
9.65E+01
9.65E+01
9.65E+01

9.65E+01
9.65E+01
9.65E+01
9.66E+01
9.67E+01
9.67E+01
9.68E+01
9.69E+01
9.69E+01
9.70E+01
9.71E+01
9.72E+01
9.73E+01
9.73E+01
9.73E+01
9.73E+01
9.72E+01
9.71E+01
9.69E+01
9.68E+01
9.67E+01
9.66E+01
9.66E+01
9.66E+01
9.66E+01
9.66E+01
9.67E+01
9.67E+01
9.67E+01
9.67E+01
9.67E+01
9.67E+01
9.67E+01
9.68E+01
9.68E+01
9.69E+01
9.70E+01
9.70E+01
9.70E+01
9.70E+01
9.69E+01
9.68E+01
9.67E+01
9.66E+01
9.66E+01
9.65E+01
9.66E+01

9.66E+01
9.67E+01
9.67E+01
9.68E+01
9.68E+01
9.69E+01
9.69E+01
9.68E+01
9.68E+01
9.67E+01
9.67E+01
9.67E+01
9.67E+01
9.68E+01
9.68E+01
9.69E+01
9.69E+01
9.69E+01
9.69E+01
9.68E+01
9.68E+01
9.68E+01
9.68E+01
9.67E+01
9.68E+01
9.68E+01
9.68E+01
9.68E+01
9.69E+01
9.69E+01
9.69E+01
9.69E+01
9.69E+01
9.69E+01
9.69E+01
9.68E+01
9.68E+01
9.68E+01
9.68E+01
9.67E+01
9.67E+01
9.67E+01
9.67E+01
9.67E+01
9.67E+01
9.67E+01
9.67E+01

9.67E+01
9.67E+01
9.67E+01
9.67E+01
9.67E+01
9.67E+01
9.68E+01
9.68E+01
9.69E+01
9.69E+01
9.70E+01
9.70E+01
9.70E+01
9.70E+01
9.69E+01
9.69E+01
9.68E+01
9.67E+01
9.66E+01
9.66E+01
9.66E+01
9.66E+01
9.66E+01
9.67E+01
9.67E+01
9.67E+01
9.67E+01
9.67E+01
9.67E+01
9.68E+01
9.68E+01
9.68E+01
9.69E+01
9.69E+01
9.69E+01
9.69E+01
9.69E+01
9.70E+01
9.70E+01
9.70E+01
9.70E+01
9.70E+01
9.70E+01
9.69E+01
9.69E+01
9.68E+01
9.68E+01

9.67E+01
9.67E+01
9.66E+01
9.66E+01
9.65E+01
9.65E+01
9.65E+01
9.65E+01
9.66E+01
9.66E+01
9.67E+01
9.67E+01
9.67E+01
9.68E+01
9.68E+01
9.68E+01
9.68E+01
9.68E+01
9.68E+01
9.67E+01
9.67E+01
9.67E+01
9.66E+01
9.66E+01
9.66E+01
9.66E+01
9.66E+01
9.66E+01
9.67E+01
9.67E+01
9.67E+01
9.66E+01
9.66E+01
9.66E+01
9.65E+01
9.65E+01
9.65E+01
9.64E+01
9.64E+01
9.65E+01
9.65E+01
9.66E+01
9.66E+01
9.67E+01
9.67E+01
9.67E+01
9.67E+01

9.67E+01
9.67E+01
9.66E+01
9.66E+01
9.66E+01
9.65E+01
9.65E+01
9.65E+01
9.64E+01
9.64E+01
9.64E+01
9.63E+01
9.63E+01
9.63E+01
9.64E+01
9.64E+01
9.65E+01
9.66E+01
9.67E+01
9.68E+01
9.68E+01
9.68E+01
9.68E+01
9.67E+01
9.66E+01
9.66E+01
9.66E+01
9.66E+01
9.67E+01
9.67E+01
9.68E+01
9.69E+01
9.68E+01
9.68E+01
9.67E+01
9.67E+01
9.66E+01
9.66E+01
9.66E+01
9.66E+01
9.65E+01
9.65E+01
9.65E+01
9.64E+01
9.64E+01
9.63E+01
9.63E+01

9.62E+01
9.62E+01
9.63E+01
9.63E+01
9.64E+01
9.65E+01
9.67E+01
9.68E+01
9.68E+01
9.69E+01
9.69E+01
9.68E+01
9.68E+01
9.67E+01
9.66E+01
9.65E+01
9.65E+01
9.65E+01
9.65E+01
9.65E+01
9.65E+01
9.65E+01
9.65E+01
9.65E+01
9.65E+01
9.65E+01
9.64E+01
9.64E+01
9.64E+01
9.64E+01
9.64E+01
9.64E+01
9.64E+01
9.64E+01
9.65E+01
9.65E+01
9.66E+01
9.66E+01
9.66E+01
9.66E+01
9.66E+01
9.66E+01
9.66E+01
9.66E+01
9.65E+01
9.65E+01
9.65E+01

9.65E+01
9.65E+01
9.65E+01
9.65E+01
9.66E+01
9.66E+01
9.66E+01
9.66E+01
9.66E+01
9.66E+01
9.66E+01
9.66E+01
9.66E+01
9.66E+01
9.67E+01
9.67E+01
9.67E+01
9.68E+01
9.68E+01
9.67E+01
9.67E+01
9.67E+01
9.66E+01
9.66E+01
9.65E+01
9.65E+01
9.65E+01
9.65E+01
9.65E+01
9.65E+01
9.65E+01
9.66E+01
9.66E+01
9.66E+01
9.67E+01
9.67E+01
9.67E+01
9.67E+01
9.66E+01
9.66E+01
9.66E+01
9.66E+01
9.66E+01
9.66E+01
9.66E+01
9.65E+01
9.65E+01

9.64E+01
9.64E+01
9.64E+01
9.63E+01
9.64E+01
9.64E+01
9.64E+01
9.65E+01
9.65E+01
9.66E+01
9.66E+01
9.66E+01
9.66E+01
9.66E+01
9.66E+01
9.65E+01
9.65E+01
9.65E+01
9.65E+01
9.65E+01
9.65E+01
9.66E+01
9.66E+01
9.66E+01
9.65E+01
9.65E+01
9.65E+01
9.64E+01
9.64E+01
9.64E+01
9.63E+01
9.63E+01
9.63E+01
9.63E+01
9.64E+01
9.64E+01
9.65E+01
9.66E+01
9.66E+01
9.66E+01
9.65E+01
9.65E+01
9.64E+01
9.64E+01
9.63E+01
9.63E+01
9.63E+01

9.64E+01
9.64E+01
9.65E+01
9.65E+01
9.65E+01
9.65E+01
9.65E+01
9.65E+01
9.65E+01
9.65E+01
9.65E+01
9.64E+01
9.64E+01
9.64E+01
9.64E+01
9.63E+01
9.63E+01
9.63E+01
9.63E+01
9.63E+01
9.64E+01
9.64E+01
9.65E+01
9.65E+01
9.66E+01
9.66E+01
9.66E+01
9.66E+01
9.66E+01
9.65E+01
9.65E+01
9.65E+01
9.65E+01
9.65E+01
9.65E+01
9.65E+01
9.65E+01
9.65E+01
9.65E+01
9.65E+01
9.64E+01
9.64E+01
9.63E+01
9.63E+01
9.63E+01
9.63E+01
9.62E+01

9.62E+01
9.62E+01
9.62E+01
9.62E+01
9.63E+01
9.64E+01
9.64E+01
9.65E+01
9.65E+01
9.65E+01
9.65E+01
9.65E+01
9.64E+01
9.64E+01
9.64E+01
9.65E+01
9.64E+01
9.64E+01
9.64E+01
9.63E+01
9.62E+01
9.61E+01
9.61E+01
9.61E+01
9.61E+01
9.62E+01
9.62E+01
9.62E+01
9.63E+01
9.63E+01
9.63E+01
9.63E+01
9.62E+01
9.62E+01
9.62E+01
9.63E+01
9.63E+01
9.63E+01
9.63E+01
9.63E+01
9.63E+01
9.63E+01
9.62E+01
9.62E+01
9.61E+01
9.61E+01
9.60E+01

9.60E+01
9.61E+01
9.61E+01
9.62E+01
9.62E+01
9.63E+01
9.63E+01
9.63E+01
9.63E+01
9.63E+01
9.62E+01
9.61E+01
9.61E+01
9.60E+01
9.60E+01
9.61E+01
9.61E+01
9.62E+01
9.63E+01
9.63E+01
9.64E+01
9.64E+01
9.64E+01
9.64E+01
9.64E+01
9.63E+01
9.63E+01
9.63E+01
9.63E+01
9.62E+01
9.62E+01
9.62E+01
9.61E+01
9.61E+01
9.61E+01
9.61E+01
9.62E+01
9.62E+01
9.62E+01
9.63E+01
9.63E+01
9.63E+01
9.63E+01
9.63E+01
9.63E+01
9.63E+01
9.62E+01

9.62E+01
9.62E+01
9.62E+01
9.62E+01
9.62E+01
9.62E+01
9.62E+01
9.63E+01
9.63E+01
9.63E+01
9.63E+01
9.63E+01
9.63E+01
9.63E+01
9.62E+01
9.62E+01
9.62E+01
9.62E+01
9.62E+01
9.62E+01
9.63E+01
9.63E+01
9.63E+01
9.64E+01
9.64E+01
9.64E+01
9.64E+01
9.63E+01
9.63E+01
9.62E+01
9.61E+01
9.60E+01
9.60E+01
9.59E+01
9.59E+01
9.59E+01
9.59E+01
9.60E+01
9.60E+01
9.61E+01
9.61E+01
9.61E+01
9.61E+01
9.61E+01
9.61E+01
9.61E+01
9.61E+01

9.61E+01
9.61E+01
9.61E+01
9.61E+01
9.62E+01
9.62E+01
9.63E+01
9.63E+01
9.63E+01
9.64E+01
9.64E+01
9.63E+01
9.63E+01
9.63E+01
9.62E+01
9.62E+01
9.61E+01
9.61E+01
9.61E+01
9.62E+01
9.62E+01
9.62E+01
9.62E+01
9.63E+01
9.63E+01
9.62E+01
9.62E+01
9.62E+01
9.62E+01
9.62E+01
9.61E+01
9.61E+01
9.61E+01
9.60E+01
9.60E+01
9.61E+01
9.61E+01
9.61E+01
9.62E+01
9.62E+01
9.62E+01
9.62E+01
9.62E+01
9.62E+01
9.61E+01
9.61E+01
9.61E+01

9.61E+01
9.61E+01
9.61E+01
9.62E+01
9.62E+01
9.62E+01
9.63E+01
9.63E+01
9.63E+01
9.62E+01
9.62E+01
9.62E+01
9.62E+01
9.62E+01
9.63E+01
9.63E+01
9.62E+01
9.62E+01
9.62E+01
9.62E+01
9.61E+01
9.61E+01
9.61E+01
9.61E+01
9.61E+01
9.61E+01
9.61E+01
9.62E+01
9.62E+01
9.63E+01
9.63E+01
9.63E+01
9.64E+01
9.64E+01
9.64E+01
9.64E+01
9.64E+01
9.64E+01
9.64E+01
9.63E+01
9.63E+01
9.63E+01
9.63E+01
9.62E+01
9.62E+01
9.62E+01
9.62E+01

9.62E+01
9.62E+01
9.61E+01
9.61E+01
9.61E+01
9.61E+01
9.61E+01
9.61E+01
9.61E+01
9.61E+01
9.62E+01
9.62E+01
9.62E+01
9.61E+01
9.61E+01
9.61E+01
9.61E+01
9.61E+01
9.60E+01
9.60E+01
9.60E+01
9.60E+01
9.60E+01
9.60E+01
9.61E+01
9.61E+01
9.61E+01
9.61E+01
9.61E+01
9.61E+01
9.60E+01
9.60E+01
9.60E+01
9.60E+01
9.60E+01
9.60E+01
9.61E+01
9.61E+01
9.62E+01
9.62E+01
9.63E+01
9.63E+01
9.63E+01
9.63E+01
9.63E+01
9.62E+01
9.62E+01

9.61E+01
9.61E+01
9.61E+01
9.61E+01
9.61E+01
9.61E+01
9.61E+01
9.61E+01
9.61E+01
9.61E+01
9.60E+01
9.60E+01
9.60E+01
9.60E+01
9.60E+01
9.61E+01
9.61E+01
9.61E+01
9.61E+01
9.61E+01
9.61E+01
9.61E+01
9.60E+01
9.60E+01
9.60E+01
9.60E+01
9.60E+01
9.59E+01
9.59E+01
9.59E+01
9.60E+01
9.60E+01
9.60E+01
9.60E+01
9.60E+01
9.60E+01
9.60E+01
9.60E+01
9.60E+01
9.60E+01
9.60E+01
9.60E+01
9.60E+01
9.60E+01
9.60E+01
9.59E+01
9.59E+01

9.59E+01
9.59E+01
9.59E+01
9.60E+01
9.60E+01
9.60E+01
9.60E+01
9.61E+01
9.61E+01
9.61E+01
9.61E+01
9.60E+01
9.60E+01
9.60E+01
9.59E+01
9.59E+01
9.59E+01
9.58E+01
9.58E+01
9.58E+01
9.58E+01
9.58E+01
9.58E+01
9.58E+01
9.58E+01
9.58E+01
9.58E+01
9.59E+01
9.59E+01
9.59E+01
9.59E+01
9.59E+01
9.59E+01
9.59E+01
9.60E+01
9.60E+01
9.60E+01
9.60E+01
9.60E+01
9.60E+01
9.59E+01
9.59E+01
9.59E+01
9.58E+01
9.58E+01
9.58E+01
9.58E+01

9.58E+01
9.58E+01
9.58E+01
9.57E+01
9.57E+01
9.57E+01
9.57E+01
9.57E+01
9.58E+01
9.58E+01
9.58E+01
9.58E+01
9.58E+01
9.58E+01
9.58E+01
9.58E+01
9.58E+01
9.58E+01
9.58E+01
9.58E+01
9.58E+01
9.58E+01
9.58E+01
9.58E+01
9.58E+01
9.58E+01
9.58E+01
9.58E+01
9.57E+01
9.57E+01
9.57E+01
9.57E+01
9.57E+01
9.57E+01
9.57E+01
9.57E+01
9.57E+01
9.57E+01
9.57E+01
9.57E+01
9.57E+01
9.57E+01
9.58E+01
9.58E+01
9.58E+01
9.58E+01
9.58E+01

9.58E+01
9.57E+01
9.57E+01
9.57E+01
9.57E+01
9.57E+01
9.57E+01
9.57E+01
9.57E+01
9.57E+01
9.57E+01
9.57E+01
9.58E+01
9.58E+01
9.57E+01
9.57E+01
9.57E+01
9.57E+01
9.57E+01
9.57E+01
9.57E+01
9.57E+01
9.57E+01
9.57E+01
9.57E+01
9.56E+01
9.56E+01
9.56E+01
9.56E+01
9.56E+01
9.56E+01
9.56E+01
9.56E+01
9.56E+01
9.56E+01
9.56E+01
9.56E+01
9.57E+01
9.57E+01
9.57E+01
9.57E+01
9.56E+01
9.56E+01
9.56E+01
9.56E+01
9.56E+01
9.56E+01

9.56E+01
9.56E+01
9.56E+01
9.57E+01
9.57E+01
9.57E+01
9.57E+01
9.57E+01
9.56E+01
9.56E+01
9.56E+01
9.56E+01
9.56E+01
9.56E+01
9.56E+01
9.56E+01
9.56E+01
9.56E+01
9.56E+01
9.56E+01
9.56E+01
9.56E+01
9.56E+01
9.56E+01
9.56E+01
9.56E+01
9.56E+01
9.56E+01
9.56E+01
9.57E+01
9.57E+01
9.57E+01
9.57E+01
9.56E+01
9.56E+01
9.56E+01
9.56E+01
9.56E+01
9.56E+01
9.56E+01
9.56E+01
9.55E+01
9.55E+01
9.55E+01
9.55E+01
9.55E+01
9.55E+01

9.55E+01
9.55E+01
9.55E+01
9.56E+01
9.56E+01
9.56E+01
9.56E+01
9.56E+01
9.56E+01
9.56E+01
9.56E+01
9.56E+01
9.56E+01
9.56E+01
9.56E+01
9.56E+01
9.56E+01
9.56E+01
9.56E+01
9.56E+01
9.56E+01
9.56E+01
9.56E+01
9.56E+01
9.56E+01
9.55E+01
9.55E+01
9.55E+01
9.55E+01
9.55E+01
9.55E+01
9.55E+01
9.55E+01
9.55E+01
9.55E+01
9.55E+01
9.55E+01
9.55E+01
9.55E+01
9.55E+01
9.55E+01
9.55E+01
9.55E+01
9.55E+01
9.54E+01
9.54E+01
9.54E+01

9.54E+01
9.53E+01
9.53E+01
9.53E+01
9.53E+01
9.53E+01
9.53E+01
9.53E+01
9.53E+01
9.54E+01
9.54E+01
9.54E+01
9.54E+01
9.54E+01
9.54E+01
9.54E+01
9.54E+01
9.54E+01
9.54E+01
9.54E+01
9.54E+01
9.54E+01
9.54E+01
9.54E+01
9.54E+01
9.54E+01
9.54E+01
9.54E+01
9.54E+01
9.54E+01
9.54E+01
9.55E+01
9.55E+01
9.55E+01
9.55E+01
9.55E+01
9.55E+01
9.55E+01
9.54E+01
9.54E+01
9.54E+01
9.54E+01
9.54E+01
9.54E+01
9.54E+01
9.54E+01
9.53E+01
9.53E+01

9.53E+01
9.53E+01
9.53E+01
9.53E+01
9.53E+01
9.54E+01
9.54E+01
9.54E+01
9.54E+01
9.54E+01
9.55E+01
9.55E+01
9.55E+01
9.55E+01
9.55E+01
9.54E+01
9.54E+01
9.54E+01
9.54E+01
9.54E+01
9.53E+01
9.53E+01
9.53E+01
9.53E+01
9.53E+01
9.53E+01
9.53E+01
9.53E+01
9.53E+01
9.53E+01
9.53E+01
9.53E+01
9.53E+01
9.53E+01
9.53E+01
9.53E+01
9.54E+01
9.54E+01
9.54E+01
9.54E+01
9.53E+01
9.53E+01
9.53E+01
9.53E+01
9.53E+01
9.53E+01
9.53E+01

9.53E+01
9.53E+01
9.53E+01
9.53E+01
9.53E+01
9.52E+01
9.52E+01
9.52E+01
9.52E+01
9.51E+01
9.51E+01
9.51E+01
9.51E+01
9.52E+01
9.52E+01
9.52E+01
9.52E+01
9.52E+01
9.51E+01
9.51E+01
9.51E+01
9.51E+01
9.52E+01
9.52E+01
9.52E+01
9.52E+01
9.52E+01
9.52E+01
9.51E+01
9.51E+01
9.51E+01
9.51E+01
9.51E+01
9.51E+01
9.51E+01
9.51E+01
9.51E+01
9.52E+01
9.52E+01
9.51E+01
9.51E+01
9.51E+01
9.51E+01
9.51E+01
9.51E+01
9.51E+01
9.51E+01

9.51E+01
9.52E+01
9.52E+01
9.52E+01
9.52E+01
9.52E+01
9.52E+01
9.52E+01
9.52E+01
9.52E+01
9.51E+01
9.51E+01
9.51E+01
9.51E+01
9.51E+01
9.51E+01
9.51E+01
9.52E+01
9.52E+01
9.52E+01
9.52E+01
9.52E+01
9.52E+01
9.51E+01
9.51E+01
9.51E+01
9.50E+01
9.50E+01
9.50E+01
9.49E+01
9.49E+01
9.50E+01
9.50E+01
9.50E+01
9.50E+01
9.50E+01
9.50E+01
9.50E+01
9.50E+01
9.50E+01
9.50E+01
9.50E+01
9.50E+01
9.50E+01
9.50E+01
9.50E+01
9.50E+01

9.50E+01
9.49E+01
9.49E+01
9.49E+01
9.49E+01
9.49E+01
9.49E+01
9.49E+01
9.49E+01
9.49E+01
9.49E+01
9.49E+01
9.49E+01
9.49E+01
9.49E+01
9.49E+01
9.49E+01
9.49E+01
9.49E+01
9.49E+01
9.49E+01
9.49E+01
9.49E+01
9.49E+01
9.49E+01
9.49E+01
9.49E+01
9.49E+01
9.49E+01
9.49E+01
9.49E+01
9.49E+01
9.48E+01
9.48E+01
9.48E+01
9.48E+01
9.48E+01
9.47E+01
9.47E+01
9.47E+01
9.47E+01
9.46E+01
9.46E+01
9.46E+01
9.46E+01
9.45E+01
9.45E+01

9.45E+01
9.44E+01
9.44E+01
9.44E+01
9.44E+01
9.44E+01
9.44E+01
9.44E+01
9.44E+01
9.44E+01
9.44E+01
9.45E+01
9.45E+01
9.45E+01
9.45E+01
9.45E+01
9.44E+01
9.44E+01
9.44E+01
9.44E+01
9.44E+01
9.44E+01
9.44E+01
9.44E+01
9.44E+01
9.44E+01
9.44E+01
9.44E+01
9.44E+01
9.44E+01
9.44E+01
9.43E+01
9.43E+01
9.43E+01
9.43E+01
9.43E+01
9.43E+01
9.43E+01
9.44E+01
9.44E+01
9.44E+01
9.44E+01
9.44E+01
9.43E+01
9.43E+01
9.43E+01
9.43E+01

9.42E+01
9.42E+01
9.42E+01
9.42E+01
9.42E+01
9.42E+01
9.42E+01
9.42E+01
9.42E+01
9.42E+01
9.43E+01
9.43E+01
9.43E+01
9.43E+01
9.43E+01
9.43E+01
9.43E+01
9.42E+01
9.42E+01
9.42E+01
9.42E+01
9.42E+01
9.42E+01
9.42E+01
9.42E+01
9.42E+01
9.42E+01
9.42E+01
9.42E+01
9.42E+01
9.42E+01
9.42E+01
9.42E+01
9.42E+01
9.42E+01
9.42E+01
9.42E+01
9.42E+01
9.42E+01
9.42E+01
9.42E+01
9.42E+01
9.41E+01
9.41E+01
9.41E+01
9.41E+01
9.41E+01

9.41E+01
9.41E+01
9.41E+01
9.40E+01
9.40E+01
9.40E+01
9.40E+01
9.40E+01
9.40E+01
9.40E+01
9.40E+01
9.40E+01
9.40E+01
9.40E+01
9.40E+01
9.39E+01
9.39E+01
9.39E+01
9.38E+01
9.38E+01
9.38E+01
9.38E+01
9.37E+01
9.37E+01
9.37E+01
9.37E+01
9.37E+01
9.36E+01
9.36E+01
9.36E+01
9.36E+01
9.35E+01
9.35E+01
9.35E+01
9.35E+01
9.34E+01
9.34E+01
9.34E+01
9.34E+01
9.33E+01
9.33E+01
9.33E+01
9.32E+01
9.31E+01
9.31E+01
9.30E+01
9.29E+01

9.29E+01
9.28E+01
9.28E+01
9.28E+01
9.28E+01
9.28E+01
9.28E+01
9.28E+01
9.28E+01
9.28E+01
9.28E+01
9.28E+01
9.27E+01
9.27E+01
9.27E+01
9.27E+01
9.26E+01
9.26E+01
9.26E+01
9.26E+01
9.27E+01
9.27E+01
9.27E+01
9.27E+01
9.27E+01
9.27E+01
9.27E+01
9.27E+01
9.27E+01
9.27E+01
9.27E+01
9.27E+01
9.27E+01
9.26E+01
9.26E+01
9.26E+01
9.26E+01
9.25E+01
9.25E+01
9.25E+01
9.26E+01
9.26E+01
9.26E+01
9.26E+01
9.26E+01
9.27E+01
9.27E+01

9.27E+01
9.27E+01
9.27E+01
9.27E+01
9.27E+01
9.27E+01
9.27E+01
9.27E+01
9.27E+01
9.27E+01
9.27E+01
9.27E+01
9.27E+01
9.27E+01
9.28E+01
9.28E+01
9.28E+01
9.28E+01
9.28E+01
9.28E+01
9.29E+01
9.29E+01
9.29E+01
9.29E+01
9.29E+01
9.30E+01
9.30E+01
9.30E+01
9.30E+01
9.30E+01
9.30E+01
9.29E+01
9.29E+01
9.30E+01
9.30E+01
9.30E+01
9.30E+01
9.31E+01
9.31E+01
9.31E+01
9.31E+01
9.31E+01
9.32E+01
9.32E+01
9.32E+01
9.32E+01
9.32E+01

9.32E+01
9.32E+01
9.32E+01
9.33E+01
9.33E+01
9.33E+01
9.33E+01
9.33E+01
9.33E+01
9.33E+01
9.33E+01
9.32E+01
9.32E+01
9.32E+01
9.32E+01
9.32E+01
9.32E+01
9.32E+01
9.32E+01
9.33E+01
9.33E+01
9.33E+01
9.33E+01
9.33E+01
9.33E+01
9.33E+01
9.33E+01
9.33E+01
9.32E+01
9.32E+01
9.32E+01
9.32E+01
9.32E+01
9.32E+01
9.32E+01
9.33E+01
9.33E+01
9.33E+01
9.33E+01
9.34E+01
9.34E+01
9.34E+01
9.33E+01
9.33E+01
9.33E+01
9.33E+01
9.33E+01

9.33E+01
9.33E+01
9.33E+01
9.33E+01
9.33E+01
9.33E+01
9.33E+01
9.33E+01
9.32E+01
9.32E+01
9.32E+01
9.32E+01
9.33E+01
9.33E+01
9.33E+01
9.33E+01
9.32E+01
9.32E+01
9.32E+01
9.32E+01
9.32E+01
9.32E+01
9.32E+01
9.32E+01
9.32E+01
9.32E+01
9.32E+01
9.32E+01
9.32E+01
9.32E+01
9.32E+01
9.32E+01
9.32E+01
9.32E+01
9.31E+01
9.31E+01
9.31E+01
9.30E+01
9.30E+01
9.30E+01
9.30E+01
9.30E+01
9.30E+01
9.30E+01
9.30E+01
9.30E+01
9.30E+01

9.30E+01
9.30E+01
9.30E+01
9.30E+01
9.30E+01
9.30E+01
9.30E+01
9.29E+01
9.29E+01
9.29E+01
9.29E+01
9.29E+01
9.29E+01
9.29E+01
9.29E+01
9.29E+01
9.30E+01
9.30E+01
9.30E+01
9.30E+01
9.30E+01
9.30E+01
9.30E+01
9.30E+01
9.30E+01
9.30E+01
9.30E+01
9.30E+01
9.30E+01
9.30E+01
9.30E+01
9.30E+01
9.30E+01
9.30E+01
9.30E+01
9.29E+01
9.29E+01
9.29E+01
9.29E+01
9.29E+01
9.29E+01
9.29E+01
9.29E+01
9.29E+01
9.28E+01
9.28E+01
9.28E+01

9.28E+01
9.28E+01
9.27E+01
9.27E+01
9.27E+01
9.27E+01
9.27E+01
9.27E+01
9.27E+01
9.27E+01
9.27E+01
9.27E+01
9.27E+01
9.27E+01
9.28E+01
9.28E+01
9.28E+01
9.27E+01
9.27E+01
9.27E+01
9.27E+01
9.26E+01
9.26E+01
9.26E+01
9.26E+01
9.26E+01
9.25E+01
9.25E+01
9.25E+01
9.25E+01
9.25E+01
9.25E+01
9.25E+01
9.25E+01
9.25E+01
9.25E+01
9.25E+01
9.25E+01
9.25E+01
9.25E+01
9.25E+01
9.25E+01
9.24E+01
9.24E+01
9.24E+01
9.24E+01
9.24E+01

9.24E+01
9.24E+01
9.24E+01
9.23E+01
9.23E+01
9.23E+01
9.23E+01
9.23E+01
9.23E+01
9.23E+01
9.23E+01
9.22E+01
9.22E+01
9.22E+01
9.22E+01
9.22E+01
9.22E+01
9.22E+01
9.21E+01
9.21E+01
9.21E+01
9.21E+01
9.21E+01
9.21E+01
9.21E+01
9.20E+01
9.20E+01
9.20E+01
9.20E+01
9.20E+01
9.20E+01
9.20E+01
9.20E+01
9.20E+01
9.20E+01
9.20E+01
9.20E+01
9.20E+01
9.20E+01
9.20E+01
9.20E+01
9.20E+01
9.20E+01
9.20E+01
9.20E+01
9.20E+01
9.20E+01

9.20E+01
9.20E+01
9.20E+01
9.20E+01
9.20E+01
9.19E+01
9.19E+01
9.19E+01
9.18E+01
9.18E+01
9.18E+01
9.18E+01
9.17E+01
9.17E+01
9.16E+01
9.16E+01
9.15E+01
9.15E+01
9.15E+01
9.14E+01
9.14E+01
9.14E+01
9.14E+01
9.14E+01
9.15E+01
9.15E+01
9.15E+01
9.15E+01
9.15E+01
9.15E+01
9.15E+01
9.15E+01
9.15E+01
9.15E+01
9.15E+01
9.15E+01
9.15E+01
9.15E+01
9.14E+01
9.14E+01
9.14E+01
9.14E+01
9.13E+01
9.13E+01
9.13E+01
9.13E+01
9.13E+01

9.13E+01
9.13E+01
9.13E+01
9.13E+01
9.13E+01
9.13E+01
9.12E+01
9.12E+01
9.12E+01
9.11E+01
9.11E+01
9.11E+01
9.11E+01
9.11E+01
9.12E+01
9.12E+01
9.12E+01
9.12E+01
9.12E+01
9.12E+01
9.11E+01
9.11E+01
9.11E+01
9.11E+01
9.10E+01
9.10E+01
9.10E+01
9.10E+01
9.09E+01
9.09E+01
9.09E+01
9.09E+01
9.09E+01
9.08E+01
9.08E+01
9.08E+01
9.08E+01
9.07E+01
9.07E+01
9.06E+01
9.06E+01
9.06E+01
9.06E+01
9.06E+01
9.06E+01
9.07E+01
9.07E+01

9.07E+01
9.06E+01
9.06E+01
9.06E+01
9.05E+01
9.05E+01
9.05E+01
9.05E+01
9.05E+01
9.05E+01
9.05E+01
9.05E+01
9.05E+01
9.05E+01
9.04E+01
9.04E+01
9.04E+01
9.03E+01
9.03E+01
9.03E+01
9.03E+01
9.03E+01
9.03E+01
9.03E+01
9.03E+01
9.03E+01
9.02E+01
9.01E+01
9.01E+01
9.00E+01
9.00E+01
9.00E+01
9.00E+01
9.00E+01
9.00E+01
9.00E+01
9.00E+01
9.00E+01
9.00E+01
9.00E+01
9.01E+01
9.01E+01
9.00E+01
9.00E+01
9.00E+01
9.00E+01
8.99E+01

8.99E+01
8.98E+01
8.98E+01
8.98E+01
8.97E+01
8.97E+01
8.97E+01
8.97E+01
8.97E+01
8.97E+01
8.96E+01
8.96E+01
8.96E+01
8.96E+01
8.95E+01
8.95E+01
8.95E+01
8.94E+01
8.94E+01
8.94E+01
8.94E+01
8.94E+01
8.94E+01
8.95E+01
8.95E+01
8.95E+01
8.94E+01
8.94E+01
8.94E+01
8.93E+01
8.93E+01
8.92E+01
8.92E+01
8.92E+01
8.91E+01
8.91E+01
8.91E+01
8.91E+01
8.91E+01
8.91E+01
8.91E+01
8.90E+01
8.90E+01
8.90E+01
8.90E+01
8.89E+01
8.89E+01

8.89E+01
8.89E+01
8.89E+01
8.89E+01
8.89E+01
8.89E+01
8.89E+01
8.89E+01
8.88E+01
8.88E+01
8.87E+01
8.87E+01
8.87E+01
8.86E+01
8.86E+01
8.86E+01
8.86E+01
8.86E+01
8.86E+01
8.86E+01
8.86E+01
8.86E+01
8.86E+01
8.86E+01
8.86E+01
8.86E+01
8.86E+01
8.85E+01
8.85E+01
8.85E+01
8.84E+01
8.84E+01
8.84E+01
8.83E+01
8.83E+01
8.83E+01
8.83E+01
8.83E+01
8.84E+01
8.84E+01
8.83E+01
8.83E+01
8.82E+01
8.82E+01
8.81E+01
8.81E+01
8.81E+01

8.80E+01
8.80E+01
8.81E+01
8.81E+01
8.81E+01
8.81E+01
8.81E+01
8.80E+01
8.80E+01
8.80E+01
8.79E+01
8.79E+01
8.78E+01
8.78E+01
8.78E+01
8.78E+01
8.78E+01
8.78E+01
8.78E+01
8.78E+01
8.78E+01
8.78E+01
8.78E+01
8.78E+01
8.78E+01
8.78E+01
8.77E+01
8.77E+01
8.77E+01
8.77E+01
8.76E+01
8.76E+01
8.76E+01
8.76E+01
8.75E+01
8.75E+01
8.74E+01
8.74E+01
8.74E+01
8.74E+01
8.74E+01
8.74E+01
8.75E+01
8.75E+01
8.75E+01
8.75E+01
8.75E+01

8.74E+01
8.74E+01
8.74E+01
8.74E+01
8.73E+01
8.73E+01
8.73E+01
8.73E+01
8.73E+01
8.73E+01
8.73E+01
8.72E+01
8.72E+01
8.72E+01
8.72E+01
8.71E+01
8.71E+01
8.71E+01
8.70E+01
8.70E+01
8.69E+01
8.69E+01
8.69E+01
8.69E+01
8.69E+01
8.69E+01
8.69E+01
8.70E+01
8.69E+01
8.69E+01
8.69E+01
8.69E+01
8.68E+01
8.68E+01
8.68E+01
8.68E+01
8.68E+01
8.68E+01
8.67E+01
8.67E+01
8.67E+01
8.67E+01
8.67E+01
8.67E+01
8.67E+01
8.66E+01
8.66E+01

8.66E+01
8.66E+01
8.66E+01
8.66E+01
8.66E+01
8.66E+01
8.65E+01
8.65E+01
8.65E+01
8.65E+01
8.65E+01
8.65E+01
8.65E+01
8.65E+01
8.65E+01
8.65E+01
8.65E+01
8.65E+01
8.65E+01
8.65E+01
8.65E+01
8.65E+01
8.65E+01
8.65E+01
8.65E+01
8.65E+01
8.65E+01
8.64E+01
8.64E+01
8.64E+01
8.64E+01
8.64E+01
8.64E+01
8.64E+01
8.63E+01
8.63E+01
8.63E+01
8.63E+01
8.63E+01
8.63E+01
8.63E+01
8.63E+01
8.63E+01
8.62E+01
8.62E+01
8.62E+01
8.61E+01

8.61E+01
8.61E+01
8.61E+01
8.61E+01
8.61E+01
8.61E+01
8.61E+01
8.62E+01
8.62E+01
8.62E+01
8.62E+01
8.62E+01
8.62E+01
8.62E+01
8.62E+01
8.61E+01
8.61E+01
8.61E+01
8.60E+01
8.60E+01
8.60E+01
8.60E+01
8.60E+01
8.60E+01
8.60E+01
8.60E+01
8.59E+01
8.59E+01
8.59E+01
8.59E+01
8.59E+01
8.60E+01
8.60E+01
8.60E+01
8.60E+01
8.59E+01
8.59E+01
8.59E+01
8.59E+01
8.58E+01
8.58E+01
8.58E+01
8.58E+01
8.58E+01
8.58E+01
8.58E+01
8.58E+01

8.58E+01
8.58E+01
8.58E+01
8.59E+01
8.59E+01
8.59E+01
8.59E+01
8.59E+01
8.59E+01
8.59E+01
8.59E+01
8.59E+01
8.59E+01
8.59E+01
8.59E+01
8.59E+01
8.58E+01
8.58E+01
8.58E+01
8.58E+01
8.58E+01
8.58E+01
8.58E+01
8.58E+01
8.58E+01
8.58E+01
8.58E+01
8.58E+01
8.58E+01
8.58E+01
8.58E+01
8.57E+01
8.57E+01
8.57E+01
8.56E+01
8.56E+01
8.55E+01
8.55E+01
8.55E+01
8.55E+01
8.54E+01
8.54E+01
8.54E+01
8.54E+01
8.54E+01
8.54E+01
8.54E+01
8.55E+01

8.55E+01
8.55E+01
8.55E+01
8.55E+01
8.56E+01
8.56E+01
8.56E+01
8.56E+01
8.56E+01
8.56E+01
8.56E+01
8.56E+01
8.55E+01
8.55E+01
8.54E+01
8.53E+01
8.53E+01
8.53E+01
8.53E+01
8.53E+01
8.53E+01
8.53E+01
8.53E+01
8.53E+01
8.54E+01
8.54E+01
8.54E+01
8.54E+01
8.54E+01
8.54E+01
8.54E+01
8.54E+01
8.54E+01
8.55E+01
8.54E+01
8.54E+01
8.54E+01
8.54E+01
8.53E+01
8.52E+01
8.52E+01
8.52E+01
8.51E+01
8.51E+01
8.51E+01
8.51E+01
8.51E+01

8.51E+01
8.51E+01
8.52E+01
8.52E+01
8.52E+01
8.51E+01
8.51E+01
8.51E+01
8.51E+01
8.51E+01
8.51E+01
8.51E+01
8.51E+01
8.51E+01
8.50E+01
8.50E+01
8.50E+01
8.50E+01
8.50E+01
8.50E+01
8.50E+01
8.50E+01
8.50E+01
8.50E+01
8.51E+01
8.51E+01
8.51E+01
8.50E+01
8.50E+01
8.50E+01
8.49E+01
8.49E+01
8.49E+01
8.49E+01
8.49E+01
8.49E+01
8.49E+01
8.49E+01
8.49E+01
8.49E+01
8.49E+01
8.49E+01
8.49E+01
8.48E+01
8.48E+01
8.48E+01
8.47E+01

8.47E+01
8.47E+01
8.47E+01
8.47E+01
8.47E+01
8.48E+01
8.48E+01
8.48E+01
8.48E+01
8.48E+01
8.48E+01
8.48E+01
8.48E+01
8.48E+01
8.47E+01
8.47E+01
8.47E+01
8.47E+01
8.46E+01
8.46E+01
8.46E+01
8.46E+01
8.46E+01
8.46E+01
8.46E+01
8.46E+01
8.46E+01
8.46E+01
8.46E+01
8.46E+01
8.46E+01
8.46E+01
8.46E+01
8.46E+01
8.46E+01
8.46E+01
8.46E+01
8.46E+01
8.46E+01
8.46E+01
8.46E+01
8.46E+01
8.46E+01
8.45E+01
8.44E+01
8.44E+01
8.43E+01

8.43E+01
8.43E+01
8.43E+01
8.43E+01
8.43E+01
8.43E+01
8.43E+01
8.43E+01
8.43E+01
8.44E+01
8.44E+01
8.44E+01
8.44E+01
8.44E+01
8.44E+01
8.44E+01
8.44E+01
8.44E+01
8.44E+01
8.44E+01
8.44E+01
8.44E+01
8.44E+01
8.45E+01
8.45E+01
8.45E+01
8.45E+01
8.45E+01
8.45E+01
8.45E+01
8.46E+01
8.46E+01
8.46E+01
8.46E+01
8.46E+01
8.45E+01
8.45E+01
8.45E+01
8.45E+01
8.46E+01
8.46E+01
8.46E+01
8.46E+01
8.46E+01
8.46E+01
8.46E+01
8.46E+01

8.46E+01
8.45E+01
8.45E+01
8.45E+01
8.45E+01
8.45E+01
8.45E+01
8.45E+01
8.46E+01
8.46E+01
8.46E+01
8.46E+01
8.46E+01
8.46E+01
8.45E+01
8.45E+01
8.45E+01
8.45E+01
8.45E+01
8.45E+01
8.45E+01
8.45E+01
8.46E+01
8.46E+01
8.46E+01
8.46E+01
8.46E+01
8.46E+01
8.46E+01
8.46E+01
8.46E+01
8.47E+01
8.47E+01
8.47E+01
8.46E+01
8.46E+01
8.46E+01
8.46E+01
8.46E+01
8.46E+01
8.46E+01
8.46E+01
8.47E+01
8.47E+01
8.47E+01
8.47E+01
8.47E+01

8.47E+01
8.47E+01
8.47E+01
8.47E+01
8.47E+01
8.47E+01
8.47E+01
8.47E+01
8.46E+01
8.46E+01
8.46E+01
8.46E+01
8.46E+01
8.46E+01
8.46E+01
8.46E+01
8.46E+01
8.46E+01
8.47E+01
8.47E+01
8.47E+01
8.48E+01
8.48E+01
8.48E+01
8.48E+01
8.48E+01
8.48E+01
8.48E+01
8.48E+01
8.49E+01
8.49E+01
8.49E+01
8.49E+01
8.49E+01
8.49E+01
8.49E+01
8.49E+01
8.49E+01
8.49E+01
8.49E+01
8.49E+01
8.49E+01
8.49E+01
8.49E+01
8.49E+01
8.49E+01
8.49E+01

8.49E+01
8.49E+01
8.49E+01
8.50E+01
8.50E+01
8.50E+01
8.50E+01
8.50E+01
8.50E+01
8.50E+01
8.50E+01
8.49E+01
8.49E+01
8.49E+01
8.50E+01
8.50E+01
8.50E+01
8.51E+01
8.51E+01
8.51E+01
8.51E+01
8.50E+01
8.50E+01
8.50E+01
8.50E+01
8.50E+01
8.51E+01
8.51E+01
8.52E+01
8.52E+01
8.52E+01
8.52E+01
8.52E+01
8.52E+01
8.52E+01
8.52E+01
8.52E+01
8.52E+01
8.52E+01
8.52E+01
8.53E+01
8.53E+01
8.53E+01
8.54E+01
8.54E+01
8.54E+01
8.54E+01

8.55E+01
8.55E+01
8.55E+01
8.55E+01
8.55E+01
8.55E+01
8.55E+01
8.55E+01
8.56E+01
8.56E+01
8.56E+01
8.56E+01
8.56E+01
8.56E+01
8.55E+01
8.55E+01
8.55E+01
8.56E+01
8.56E+01
8.56E+01
8.57E+01
8.57E+01
8.58E+01
8.58E+01
8.58E+01
8.58E+01
8.59E+01
8.59E+01
8.59E+01
8.59E+01
8.58E+01
8.58E+01
8.58E+01
8.58E+01
8.58E+01
8.57E+01
8.57E+01
8.57E+01
8.58E+01
8.58E+01
8.58E+01
8.59E+01
8.59E+01
8.59E+01
8.59E+01
8.59E+01
8.59E+01

8.59E+01
8.59E+01
8.58E+01
8.58E+01
8.59E+01
8.59E+01
8.59E+01
8.59E+01
8.59E+01
8.59E+01
8.59E+01
8.59E+01
8.59E+01
8.60E+01
8.60E+01
8.60E+01
8.61E+01
8.61E+01
8.61E+01
8.61E+01
8.61E+01
8.61E+01
8.61E+01
8.61E+01
8.61E+01
8.61E+01
8.61E+01
8.61E+01
8.61E+01
8.61E+01
8.62E+01
8.62E+01
8.63E+01
8.63E+01
8.63E+01
8.64E+01
8.64E+01
8.64E+01
8.64E+01
8.64E+01
8.64E+01
8.64E+01
8.64E+01
8.64E+01
8.64E+01
8.64E+01
8.64E+01

8.64E+01
8.64E+01
8.64E+01
8.65E+01
8.65E+01
8.65E+01
8.66E+01
8.66E+01
8.66E+01
8.66E+01
8.66E+01
8.66E+01
8.67E+01
8.67E+01
8.67E+01
8.67E+01
8.68E+01
8.68E+01
8.68E+01
8.68E+01
8.69E+01
8.69E+01
8.69E+01
8.70E+01
8.70E+01
8.71E+01
8.72E+01
8.72E+01
8.73E+01
8.73E+01
8.73E+01
8.73E+01
8.73E+01
8.72E+01
8.72E+01
8.71E+01
8.71E+01
8.70E+01
8.70E+01
8.69E+01
8.69E+01
8.69E+01
8.69E+01
8.69E+01
8.69E+01
8.69E+01
8.69E+01

8.69E+01
8.70E+01
8.70E+01
8.71E+01
8.72E+01
8.72E+01
8.73E+01
8.74E+01
8.74E+01
8.74E+01
8.74E+01
8.74E+01
8.74E+01
8.75E+01
8.75E+01
8.75E+01
8.75E+01
8.75E+01
8.75E+01
8.75E+01
8.75E+01
8.75E+01
8.76E+01
8.76E+01
8.76E+01
8.77E+01
8.77E+01
8.77E+01
8.78E+01
8.78E+01
8.78E+01
8.78E+01
8.78E+01
8.77E+01
8.77E+01
8.76E+01
8.76E+01
8.76E+01
8.75E+01
8.75E+01
8.75E+01
8.76E+01
8.77E+01
8.78E+01
8.79E+01
8.80E+01
8.81E+01

8.82E+01
8.82E+01
8.82E+01
8.82E+01
8.81E+01
8.81E+01
8.80E+01
8.80E+01
8.80E+01
8.80E+01
8.81E+01
8.81E+01
8.82E+01
8.83E+01
8.83E+01
8.84E+01
8.84E+01
8.84E+01
8.84E+01
8.84E+01
8.83E+01
8.83E+01
8.82E+01
8.82E+01
8.82E+01
8.82E+01
8.83E+01
8.83E+01
8.83E+01
8.83E+01
8.83E+01
8.83E+01
8.82E+01
8.81E+01
8.80E+01
8.79E+01
8.78E+01
8.79E+01
8.80E+01
8.83E+01
8.85E+01
8.87E+01
8.89E+01
8.90E+01
8.91E+01
8.91E+01
8.92E+01

8.92E+01
8.92E+01
8.92E+01
8.92E+01
8.91E+01
8.91E+01
8.91E+01
8.91E+01
8.91E+01
8.91E+01
8.91E+01
8.92E+01
8.92E+01
8.92E+01
8.93E+01
8.94E+01
8.94E+01
8.94E+01
8.94E+01
8.94E+01
8.94E+01
8.94E+01
8.94E+01
8.93E+01
8.93E+01
8.93E+01
8.93E+01
8.92E+01
8.92E+01
8.91E+01
8.90E+01
8.89E+01
8.89E+01
8.89E+01
8.90E+01
8.91E+01
8.92E+01
8.93E+01
8.93E+01
8.94E+01
8.94E+01
8.95E+01
8.95E+01
8.95E+01
8.95E+01
8.95E+01
8.95E+01

8.95E+01
8.95E+01
8.96E+01
8.96E+01
8.96E+01
8.96E+01
8.96E+01
8.95E+01
8.95E+01
8.95E+01
8.94E+01
8.94E+01
8.95E+01
8.95E+01
8.96E+01
8.98E+01
8.99E+01
9.00E+01
9.01E+01
9.02E+01
9.02E+01
9.02E+01
9.01E+01
9.00E+01
8.99E+01
8.97E+01
8.95E+01
8.94E+01
8.93E+01
8.93E+01
8.94E+01
8.95E+01
8.96E+01
8.97E+01
8.97E+01
8.97E+01
8.97E+01
8.96E+01
8.95E+01
8.95E+01
8.95E+01
8.95E+01
8.96E+01
8.96E+01
8.97E+01
8.97E+01
8.97E+01

8.96E+01
8.96E+01
8.96E+01
8.96E+01
8.97E+01
8.98E+01
8.99E+01
9.01E+01
9.02E+01
9.03E+01
9.04E+01
9.05E+01
9.06E+01
9.06E+01
9.06E+01
9.06E+01
9.06E+01
9.06E+01
9.05E+01
9.05E+01
9.04E+01
9.03E+01
9.01E+01
9.01E+01
9.01E+01
9.02E+01
9.03E+01
9.05E+01
9.07E+01
9.08E+01
9.09E+01
9.09E+01
9.09E+01
9.09E+01
9.09E+01
9.10E+01
9.10E+01
9.11E+01
9.11E+01
9.12E+01
9.13E+01
9.13E+01
9.13E+01
9.14E+01
9.14E+01
9.14E+01
9.14E+01

9.15E+01
9.15E+01
9.15E+01
9.15E+01
9.15E+01
9.14E+01
9.14E+01
9.14E+01
9.13E+01
9.13E+01
9.12E+01
9.12E+01
9.11E+01
9.10E+01
9.09E+01
9.08E+01
9.06E+01
9.05E+01
9.04E+01
9.03E+01
9.03E+01
9.05E+01
9.07E+01
9.10E+01
9.12E+01
9.15E+01
9.16E+01
9.18E+01
9.18E+01
9.18E+01
9.18E+01
9.19E+01
9.19E+01
9.20E+01
9.21E+01
9.22E+01
9.24E+01
9.25E+01
9.27E+01
9.27E+01
9.28E+01
9.29E+01
9.29E+01
9.29E+01
9.30E+01
9.30E+01
9.30E+01

9.31E+01
9.31E+01
9.31E+01
9.31E+01
9.31E+01
9.31E+01
9.31E+01
9.31E+01
9.30E+01
9.30E+01
9.29E+01
9.29E+01
9.28E+01
9.26E+01
9.25E+01
9.25E+01
9.24E+01
9.25E+01
9.26E+01
9.27E+01
9.28E+01
9.28E+01
9.28E+01
9.27E+01
9.26E+01
9.25E+01
9.24E+01
9.24E+01
9.25E+01
9.26E+01
9.27E+01
9.29E+01
9.30E+01
9.30E+01
9.31E+01
9.32E+01
9.33E+01
9.34E+01
9.35E+01
9.36E+01
9.37E+01
9.37E+01
9.36E+01
9.36E+01
9.35E+01
9.34E+01
9.33E+01

9.33E+01
9.32E+01
9.31E+01
9.30E+01
9.30E+01
9.29E+01
9.28E+01
9.28E+01
9.27E+01
9.28E+01
9.29E+01
9.30E+01
9.32E+01
9.34E+01
9.36E+01
9.38E+01
9.40E+01
9.41E+01
9.42E+01
9.43E+01
9.44E+01
9.43E+01
9.43E+01
9.42E+01
9.41E+01
9.40E+01
9.40E+01
9.39E+01
9.39E+01
9.40E+01
9.40E+01
9.41E+01
9.42E+01
9.42E+01
9.43E+01
9.43E+01
9.43E+01
9.43E+01
9.43E+01
9.43E+01
9.43E+01
9.43E+01
9.43E+01
9.43E+01
9.42E+01
9.42E+01
9.41E+01

9.40E+01
9.39E+01
9.37E+01
9.36E+01
9.34E+01
9.33E+01
9.32E+01
9.32E+01
9.33E+01
9.34E+01
9.35E+01
9.36E+01
9.37E+01
9.38E+01
9.39E+01
9.40E+01
9.41E+01
9.42E+01
9.42E+01
9.43E+01
9.43E+01
9.43E+01
9.43E+01
9.42E+01
9.41E+01
9.41E+01
9.40E+01
9.39E+01
9.39E+01
9.38E+01
9.38E+01
9.38E+01
9.38E+01
9.38E+01
9.38E+01
9.38E+01
9.38E+01
9.39E+01
9.39E+01
9.40E+01
9.41E+01
9.41E+01
9.42E+01
9.43E+01
9.43E+01
9.43E+01
9.43E+01

9.43E+01
9.42E+01
9.41E+01
9.40E+01
9.38E+01
9.37E+01
9.36E+01
9.35E+01
9.33E+01
9.32E+01
9.31E+01
9.31E+01
9.31E+01
9.32E+01
9.34E+01
9.36E+01
9.38E+01
9.39E+01
9.40E+01
9.41E+01
9.41E+01
9.41E+01
9.40E+01
9.40E+01
9.39E+01
9.37E+01
9.36E+01
9.35E+01
9.33E+01
9.32E+01
9.32E+01
9.33E+01
9.34E+01
9.35E+01
9.36E+01
9.35E+01
9.35E+01
9.33E+01
9.31E+01
9.29E+01
9.26E+01
9.24E+01
9.24E+01
9.25E+01
9.27E+01
9.30E+01
9.33E+01

9.35E+01
9.37E+01
9.38E+01
9.39E+01
9.39E+01
9.39E+01
9.39E+01
9.38E+01
9.38E+01
9.37E+01
9.37E+01
9.37E+01
9.37E+01
9.38E+01
9.39E+01
9.40E+01
9.41E+01
9.42E+01
9.42E+01
9.43E+01
9.43E+01
9.43E+01
9.43E+01
9.42E+01
9.42E+01
9.42E+01
9.42E+01
9.42E+01
9.43E+01
9.43E+01
9.43E+01
9.43E+01
9.42E+01
9.42E+01
9.41E+01
9.41E+01
9.41E+01
9.42E+01
9.43E+01
9.44E+01
9.45E+01
9.46E+01
9.46E+01
9.47E+01
9.47E+01
9.47E+01
9.47E+01

9.47E+01
9.46E+01
9.46E+01
9.45E+01
9.45E+01
9.44E+01
9.44E+01
9.44E+01
9.44E+01
9.45E+01
9.45E+01
9.46E+01
9.47E+01
9.47E+01
9.47E+01
9.47E+01
9.47E+01
9.46E+01
9.45E+01
9.45E+01
9.45E+01
9.45E+01
9.46E+01
9.46E+01
9.47E+01
9.48E+01
9.48E+01
9.48E+01
9.48E+01
9.48E+01
9.48E+01
9.48E+01
9.48E+01
9.48E+01
9.47E+01
9.47E+01
9.46E+01
9.46E+01
9.45E+01
9.43E+01
9.42E+01
9.41E+01
9.40E+01
9.40E+01
9.40E+01
9.40E+01
9.41E+01

9.41E+01
9.41E+01
9.41E+01
9.41E+01
9.40E+01
9.39E+01
9.39E+01
9.39E+01
9.40E+01
9.41E+01
9.42E+01
9.43E+01
9.43E+01
9.43E+01
9.42E+01
9.42E+01
9.41E+01
9.41E+01
9.41E+01
9.41E+01
9.42E+01
9.44E+01
9.45E+01
9.46E+01
9.46E+01
9.47E+01
9.47E+01
9.47E+01
9.47E+01
9.47E+01
9.47E+01
9.47E+01
9.47E+01
9.47E+01
9.47E+01
9.46E+01
9.45E+01
9.44E+01
9.43E+01
9.42E+01
9.41E+01
9.40E+01
9.39E+01
9.38E+01
9.36E+01
9.34E+01
9.32E+01

9.31E+01
9.31E+01
9.32E+01
9.33E+01
9.35E+01
9.36E+01
9.38E+01
9.39E+01
9.40E+01
9.41E+01
9.42E+01
9.43E+01
9.44E+01
9.44E+01
9.45E+01
9.45E+01
9.45E+01
9.45E+01
9.44E+01
9.44E+01
9.43E+01
9.42E+01
9.42E+01
9.41E+01
9.41E+01
9.40E+01
9.40E+01
9.39E+01
9.39E+01
9.38E+01
9.38E+01
9.38E+01
9.38E+01
9.39E+01
9.39E+01
9.39E+01
9.38E+01
9.38E+01
9.38E+01
9.38E+01
9.39E+01
9.40E+01
9.41E+01
9.42E+01
9.43E+01
9.44E+01
9.45E+01

9.46E+01
9.47E+01
9.47E+01
9.47E+01
9.47E+01
9.47E+01
9.47E+01
9.47E+01
9.47E+01
9.47E+01
9.46E+01
9.45E+01
9.44E+01
9.43E+01
9.41E+01
9.38E+01
9.35E+01
9.32E+01
9.29E+01
9.27E+01
9.27E+01
9.30E+01
9.33E+01
9.36E+01
9.38E+01
9.40E+01
9.41E+01
9.42E+01
9.42E+01
9.43E+01
9.44E+01
9.44E+01
9.44E+01
9.44E+01
9.44E+01
9.43E+01
9.43E+01
9.42E+01
9.42E+01
9.41E+01
9.40E+01
9.39E+01
9.39E+01
9.38E+01
9.39E+01
9.39E+01
9.40E+01

9.41E+01
9.42E+01
9.43E+01
9.43E+01
9.42E+01
9.41E+01
9.40E+01
9.39E+01
9.39E+01
9.39E+01
9.40E+01
9.41E+01
9.41E+01
9.42E+01
9.42E+01
9.43E+01
9.43E+01
9.44E+01
9.45E+01
9.46E+01
9.46E+01
9.47E+01
9.47E+01
9.47E+01
9.46E+01
9.45E+01
9.44E+01
9.43E+01
9.41E+01
9.40E+01
9.39E+01
9.39E+01
9.39E+01
9.39E+01
9.39E+01
9.39E+01
9.39E+01
9.39E+01
9.39E+01
9.40E+01
9.41E+01
9.42E+01
9.43E+01
9.44E+01
9.45E+01
9.46E+01
9.46E+01

9.46E+01
9.46E+01
9.45E+01
9.44E+01
9.43E+01
9.43E+01
9.42E+01
9.42E+01
9.42E+01
9.42E+01
9.43E+01
9.44E+01
9.45E+01
9.45E+01
9.45E+01
9.45E+01
9.45E+01
9.44E+01
9.44E+01
9.43E+01
9.42E+01
9.42E+01
9.42E+01
9.42E+01
9.42E+01
9.42E+01
9.41E+01
9.40E+01
9.39E+01
9.39E+01
9.38E+01
9.38E+01
9.39E+01
9.41E+01
9.43E+01
9.44E+01
9.46E+01
9.48E+01
9.49E+01
9.49E+01
9.50E+01
9.50E+01
9.50E+01
9.50E+01
9.49E+01
9.49E+01
9.49E+01

9.49E+01
9.49E+01
9.49E+01
9.49E+01
9.48E+01
9.48E+01
9.47E+01
9.46E+01
9.45E+01
9.44E+01
9.44E+01
9.43E+01
9.43E+01
9.43E+01
9.44E+01
9.45E+01
9.45E+01
9.46E+01
9.47E+01
9.47E+01
9.47E+01
9.47E+01
9.46E+01
9.45E+01
9.44E+01
9.44E+01
9.44E+01
9.44E+01
9.45E+01
9.46E+01
9.47E+01
9.47E+01
9.48E+01
9.48E+01
9.48E+01
9.47E+01
9.47E+01
9.46E+01
9.46E+01
9.45E+01
9.45E+01
9.45E+01
9.45E+01
9.45E+01
9.46E+01
9.47E+01
9.48E+01

9.48E+01
9.49E+01
9.49E+01
9.50E+01
9.50E+01
9.50E+01
9.50E+01
9.50E+01
9.50E+01
9.50E+01
9.50E+01
9.49E+01
9.49E+01
9.48E+01
9.47E+01
9.46E+01
9.45E+01
9.45E+01
9.45E+01
9.45E+01
9.45E+01
9.46E+01
9.46E+01
9.47E+01
9.47E+01
9.47E+01
9.47E+01
9.47E+01
9.47E+01
9.47E+01
9.48E+01
9.48E+01
9.49E+01
9.49E+01
9.50E+01
9.50E+01
9.50E+01
9.50E+01
9.50E+01
9.50E+01
9.50E+01
9.50E+01
9.50E+01
9.49E+01
9.49E+01
9.49E+01
9.48E+01

9.48E+01
9.48E+01
9.48E+01
9.48E+01
9.48E+01
9.48E+01
9.48E+01
9.48E+01
9.48E+01
9.49E+01
9.49E+01
9.50E+01
9.50E+01
9.50E+01
9.50E+01
9.50E+01
9.50E+01
9.50E+01
9.49E+01
9.49E+01
9.49E+01
9.49E+01
9.48E+01
9.48E+01
9.48E+01
9.48E+01
9.48E+01
9.48E+01
9.48E+01
9.49E+01
9.49E+01
9.49E+01
9.49E+01
9.48E+01
9.48E+01
9.48E+01
9.48E+01
9.48E+01
9.48E+01
9.48E+01
9.48E+01
9.48E+01
9.48E+01
9.48E+01
9.49E+01
9.48E+01
9.48E+01

9.48E+01
9.48E+01
9.48E+01
9.48E+01
9.48E+01
9.48E+01
9.48E+01
9.49E+01
9.50E+01
9.50E+01
9.51E+01
9.51E+01
9.51E+01
9.51E+01
9.51E+01
9.51E+01
9.50E+01
9.50E+01
9.50E+01
9.49E+01
9.49E+01
9.49E+01
9.50E+01
9.50E+01
9.51E+01
9.51E+01
9.51E+01
9.51E+01
9.51E+01
9.51E+01
9.51E+01
9.51E+01
9.51E+01
9.51E+01
9.51E+01
9.51E+01
9.51E+01
9.51E+01
9.51E+01
9.51E+01
9.51E+01
9.51E+01
9.50E+01
9.50E+01
