# Supplementary material for: α-Amylase immobilization on amidoximated acrylic microfibres activated by cyanuric chloride
Source: R Soc Open Sci. 2018 Nov 28;5(11):172164. doi: 10.1098/rsos.172164 (PMC6281920; doi:10.1098/rsos.172164)
Supplement: Supplementary Figure 4 [file rsos172164supp3.pdf]

A-Amylase immobilization on amidoximated acrylic microfibers activated by cyanuric chloride

Yaaser Q. Almulaiky[1,2], Faisal M. Aqlan[3], Musab Aldhahri[4,5], Mohammed Baeshen[6] Tariq Jamal Khan[7], Khalid A. Khan[8], Mohamed Afifi[6,9], Ammar AL-Farga[1], Mohiuddin Khan Warsi[1], Mohammed Alkaled[6], Aisha A.M. Alayafi[6]

[1]Department of Biochemistry, Faculty of Science, University of Jeddah, Jeddah, Saudi Arabia
[2]Chemistry Department, Faculty of Applied Science, Taiz University, Taiz, Yemen
[3]Chemistry Department, Faculty of Science, University of Jeddah, Jeddah, Saudi Arabia
[4]Department of Biochemistry, Faculty of Science, King Abdulaziz University, Jeddah, Saudi Arabia
[5]Center of Nanotechnology, King Abdulaziz University, Jeddah, Saudi Arabia
[6]Department of biology, Faculty of Science, University of Jeddah, Jeddah, Saudi Arabia
[7]Stem Cell P2 Laboratory, The Center for Reproductive Medicine, Shantou University Medical College, Shantou, 515041, People‘s Republic of China
[8]Chemistry Department, Faculty of Science, King Abdulaziz University, Jeddah, Saudi Arabia
[9]Biochemistry Department, Faculty of Veterinary Medicine, Zagazig University, Egypt

Supplementary Figure 4

Number of repeated reaction	Residual activity %	Residual units	OD at 560 nm<br>n/3
1	100	12.6	1.380
2	95	11.97	1.311
3	92	11.59	1.2696
4	84	10.58	1.1592
5	80	10.08	1.104
6	78	9.83	1.0764
7	77	9.69	1.0626
8	77	9.67	1.060
9	72	9.07	0.993
19	72	9.07	0.991

Supplementary Figure 4 Reuse of immobilized α-amylase**.** Reusability tests of the immobilized a-amylase were carried out by addition of reaction mixture contained 1ml of 50 mM sodium acetate buffer, pH 5.5, 1ml of starch (1%) and 1cm2 immobilized enzyme. The reaction stopped

by the addition of1ml DNS reagent, followed by incubation in a boiling water bath for 10 min followed by cooling. The absorbance was recorded at 560 nm.
