# Supplementary material for: α-Amylase immobilization on amidoximated acrylic microfibres activated by cyanuric chloride
Source: R Soc Open Sci. 2018 Nov 28;5(11):172164. doi: 10.1098/rsos.172164 (PMC6281920; doi:10.1098/rsos.172164)
Supplement: Supplementary Figure 5 [file rsos172164supp4.pdf]

A-Amylase immobilization on amidoximated acrylic microfibers activated by cyanuric chloride

Yaaser Q. Almulaiky[1,2], Faisal M. Aqlan[3], Musab Aldhahri[4,5], Mohammed Baeshen[6] Tariq Jamal Khan[7], Khalid A. Khan[8], Mohamed Afifi[6,9], Ammar AL-Farga[1], Mohiuddin Khan Warsi[1], Mohammed Alkaled[6], Aisha A.M. Alayafi[6]

[1]Department of Biochemistry, Faculty of Science, University of Jeddah, Jeddah, Saudi Arabia
[2]Chemistry Department, Faculty of Applied Science, Taiz University, Taiz, Yemen
[3]Chemistry Department, Faculty of Science, University of Jeddah, Jeddah, Saudi Arabia
[4]Department of Biochemistry, Faculty of Science, King Abdulaziz University, Jeddah, Saudi Arabia
[5]Center of Nanotechnology, King Abdulaziz University, Jeddah, Saudi Arabia
[6]Department of biology, Faculty of Science, University of Jeddah, Jeddah, Saudi Arabia
[7]Stem Cell P2 Laboratory, The Center for Reproductive Medicine, Shantou University Medical College, Shantou, 515041, People‘s Republic of China
[8]Chemistry Department, Faculty of Science, King Abdulaziz University, Jeddah, Saudi Arabia
[9]Biochemistry Department, Faculty of Veterinary Medicine, Zagazig University, Egypt

Supplementary Figure 5a

pH	Soluble enzyme Residual activity %	OD at 560 nm (n/3)	Immobilized enzyme Residual activity %	OD at 560 nm (n/3)
4.5	27	0.276	41	0.269
5	45	0.455	53	0.348
5.5	81	0.823	75	0.493
6	100	1.015	86	0.565
6.5	90	0.913	100	0.658
7	77	0.781	85	0.559
7.5	38	0.385	68	0.447
8	29	0.294	47	0.309
8.5	21	0.213	39	0.256
9	17	0.176	23	0.151

Supplementary Figure 5a Optimum pH (a), Free and immobilized enzyme activity was determined at various pH using different buffers, sodium acetate (pH 4.0-6.0) and Tris-HCl (6.5-9) at 50 mM concentration. the assay was performed as described in α –amylase assay. The maximum activity was taken as 100% and % relative activity was plotted against different pH values.

Supplementary Figure 5b

Temperature C^0	Soluble enzyme Residual activity %		Immobilized enzyme Residual activity %	
	%	OD at 560 (n/3)	%	OD at 560 (n/3)
30	33	0.428	39	0.289
40	51	0.661	64	0.475
50	100	1.297	88	0.654
60	81	1.051	100	0.743
70	74	0.959	84	0.624
80	42	0.544	65	0.483

Supplementary Figure 5b Effect of temperature. Free and immobilized enzyme activity was determined at a temperature range of 20-80°C. The assay was performed as described in α – amylase assay, except at different temperature as mentioned above. The maximum activity was taken as 100% and % relative activity was plotted against different temperatures.

Supplementary Figure 5c

Temperature C^0	Soluble enzyme Residual activity %		Immobilized enzyme Residual activity %	
	%	OD at 560 (n/3)	%	OD at 560 (n/3)
30	100	1.293	100	0.747
40	100	1.291	100	0.756
50	100	1.295	100	0.750
60	81	1.045	100	0.753
70	74	0.955	84	0.635
80	42	0.542	65	0.489

Supplementary Figure 5c, thermal stability (c). Free and immobilized enzyme activity was determined at a temperature range of 20-80°C for 30 min prior to substrate addition. The assay was performed as described in α –amylase assay. The maximum activity was taken as 100% and % relative activity was plotted against different temperatures.

Supplementary Figure 5d

Starch 1%		Soluble enzyme Residual activity %	Immobilized enzyme Residual activity %
Volume	1/S (mg)	1/V (µmol/min)	1/V (µmol/min)
66 µl	0.66	5.5	6
50 µl	0.5	5.1	5.4
40 µl	0.4	4.8	4.9
33 µl	0.33	4.5	4.6
28 µl	0.28	3.9	4.4

Supplementary Figure 5d, **km (d).** The k_m values were determined from Lineweaver-Burk plots for free and immobilized enzyme by using starch concentrations from 3-7 mg/0.5 ml. The assay was performed as described in α –amylase assay.
