# Supplementary material for: α-Amylase immobilization on amidoximated acrylic microfibres activated by cyanuric chloride
Source: R Soc Open Sci. 2018 Nov 28;5(11):172164. doi: 10.1098/rsos.172164 (PMC6281920; doi:10.1098/rsos.172164)
Supplement: Supplementary Table 1 [file rsos172164supp5.pdf]

A-Amylase immobilization on amidoximated acrylic microfibers activated by cyanuric chloride

Yaaser Q. Almulaiky[1,2], Faisal M. Aqlan[3], Musab Aldhahri[4,5], Mohammed Baeshen[6] Tariq Jamal Khan[7], Khalid A. Khan[8], Mohamed Afifi[6,9], Ammar AL-Farga[1], Mohiuddin Khan Warsi[1], Mohammed Alkaled[6], Aisha A.M. Alayafi[6]

[1]Department of Biochemistry, Faculty of Science, University of Jeddah, Jeddah, Saudi Arabia
[2]Chemistry Department, Faculty of Applied Science, Taiz University, Taiz, Yemen
[3]Chemistry Department, Faculty of Science, University of Jeddah, Jeddah, Saudi Arabia
[4]Department of Biochemistry, Faculty of Science, King Abdulaziz University, Jeddah, Saudi Arabia
[5]Center of Nanotechnology, King Abdulaziz University, Jeddah, Saudi Arabia
[6]Department of biology, Faculty of Science, University of Jeddah, Jeddah, Saudi Arabia
[7]Stem Cell P2 Laboratory, The Center for Reproductive Medicine, Shantou University Medical College, Shantou, 515041, People‘s Republic of China
[8]Chemistry Department, Faculty of Science, King Abdulaziz University, Jeddah, Saudi Arabia
[9]Biochemistry Department, Faculty of Veterinary Medicine, Zagazig University, Egypt

Supplementary Table 1

Activation of acrylic fiber with cyanuric chloride

An ice-cooled solution of cyanuric chloride (2-6% w/w) in 100 ml of acetone-water mixture (50% v/v, 1:1) was prepared. Acrylic fiber (2 g) was added into this solution and left with shaking for 30 min at 0°C. Then, sodium bicarbonate solution (10% w/v, 100 ml) was drop wisely added to the above reaction mixture while shaking within 30 min at 0°C. The reaction mixture was further kept under shaking and at 0°C overnight. The acrylic fiber sample was removed from the shaker bath and washed several times with acetone, water and acetone, dried in ventilated hood and kept in a plastic bag in refrigerator ready for enzyme immobilization.

Immobilization procedure

Enzyme immobilization was carried out by end over end at 90 rpm onto the treated acrylic fiber using a solution of a-amylase made in 50 mM sodium acetate buffer (pH 4) or Tris-HCl (pH 7.0 or 8.5) at room temperature during overnight. Aliquots of the supernatant were drawn up and the acrylic fiber was dried at room temperature to verify the advancement of the immobilization.

Table 1S Effect of cyanuric chloride percentage and pH on the immobilization efficiency of α-amylase.

	pH											
	8.5				7				4			
Cyanuric chloride (%)	n O.D at 560nm	n/3	Units/min	Immobilization efficiency (%)	n O.D at 560nm	n/3	Units/min	Immobilization efficiency (%)	n O.D at 560nm	n/3	Units/min	Immobilization efficiency (%)
2	0.387	0.384	8	16	0.918	0.912	19	38	0.508	0.504	10.5	23
	0.382				0.903				0.496			
	0.383				0.915				0.508			
4	0.557	0.552	11.5	23	1.887	1.896	39.5	**79**	0.428	0.432	9	18
	0.549				1.903				0.435			
	0.550				1.898				0.433			
6	0.641	0.648	13.5	27	1.012	1.008	21	42	0.357	0.360	7.5	15
	0.653				1.003				0.364			
	0.650				1.009				0.359			

n:repeated time of reading, activity of soluble enzyme = 50 unit

n/3: mean value
