# Supplementary material for: α-Amylase immobilization on amidoximated acrylic microfibres activated by cyanuric chloride
Source: R Soc Open Sci. 2018 Nov 28;5(11):172164. doi: 10.1098/rsos.172164 (PMC6281920; doi:10.1098/rsos.172164)
Supplement: Supplementary Table 2 [file rsos172164supp6.pdf]

**A-Amylase immobilization on amidoximated acrylic microfibers activated by cyanuric chloride**

Yaaser Q. Almulaiky[1,2], Faisal M. Aqlan[3], Musab Aldhahri[4,5], Mohammed Baeshen[6] Tariq Jamal Khan[7], Khalid A. Khan[8], Mohamed Afifi[6,9], Ammar AL-Farga[1], Mohiuddin Khan Warsi[1], Mohammed Alkaled[6], Aisha A.M. Alayafi[6]

[1]Department of Biochemistry, Faculty of Science, University of Jeddah, Jeddah, Saudi Arabia
[2]Chemistry Department, Faculty of Applied Science, Taiz University, Taiz, Yemen
[3]Chemistry Department, Faculty of Science, University of Jeddah, Jeddah, Saudi Arabia
[4]Department of Biochemistry, Faculty of Science, King Abdulaziz University, Jeddah, Saudi Arabia
[5]Center of Nanotechnology, King Abdulaziz University, Jeddah, Saudi Arabia
[6]Department of biology, Faculty of Science, University of Jeddah, Jeddah, Saudi Arabia
[7]Stem Cell P2 Laboratory, The Center for Reproductive Medicine, Shantou University Medical College, Shantou, 515041, People's Republic of China
[8]Chemistry Department, Faculty of Science, King Abdulaziz University, Jeddah, Saudi Arabia
[9]Biochemistry Department, Faculty of Veterinary Medicine, Zagazig University, Egypt

## Supplementary Table 2

substrate specificity of soluble and immobilized α-amylase.

The activity with starch as the substrate is regarded as 100% activity.

| Substrate | soluble α-amylase | OD at 560 nm | Immobilized α-amylase | OD at 560 nm |
|---|---|---|---|---|
| | Residual activity % | n/3 | Residual activity % | n/3 |
| Starch | 100 | 1.231 | 100 | 0.769 |
| Glycogen | 81 | 0.997 | 90 | 0.692 |
| Amylopectin | 61 | 0.751 | 86 | 0.661 |
| Alpha-cyclodextrine | 40 | 0.493 | 48 | 0.369 |
| Beta-cyclodextrine | 26 | 0.320 | 65 | 0.499 |
| amylose | 17 | 0.209 | 28 | 0.215 |
