# Supplementary material for: α-Amylase immobilization on amidoximated acrylic microfibres activated by cyanuric chloride
Source: R Soc Open Sci. 2018 Nov 28;5(11):172164. doi: 10.1098/rsos.172164 (PMC6281920; doi:10.1098/rsos.172164)
Supplement: Supplementary Table 2 [file rsos172164supp7.pdf]

A-Amylase immobilization on amidoximated acrylic microfibers activated by cyanuric chloride

Yaaser Q. Almulaiky[1,2], Faisal M. Aqlan[3], Musab Aldhahri[4,5], Mohammed Baeshen[6] Tariq Jamal Khan[7], Khalid A. Khan[8], Mohamed Afifi[6,9], Ammar AL-Farga[1], Mohiuddin Khan Warsi[1], Mohammed Alkaled[6], Aisha A.M. Alayafi[6]

[1]Department of Biochemistry, Faculty of Science, University of Jeddah, Jeddah, Saudi Arabia
[2]Chemistry Department, Faculty of Applied Science, Taiz University, Taiz, Yemen
[3]Chemistry Department, Faculty of Science, University of Jeddah, Jeddah, Saudi Arabia
[4]Department of Biochemistry, Faculty of Science, King Abdulaziz University, Jeddah, Saudi Arabia
[5]Center of Nanotechnology, King Abdulaziz University, Jeddah, Saudi Arabia
[6]Department of biology, Faculty of Science, University of Jeddah, Jeddah, Saudi Arabia
[7]Stem Cell P2 Laboratory, The Center for Reproductive Medicine, Shantou University Medical College, Shantou, 515041, People's Republic of China
[8]Chemistry Department, Faculty of Science, King Abdulaziz University, Jeddah, Saudi Arabia
[9]Biochemistry Department, Faculty of Veterinary Medicine, Zagazig University, Egypt

Supplementary Table 2

metal ion effects on soluble and immobilized α-amylase.

Metals 2 mM	soluble α-amylase	OD at 560 nm n/3	Immobilized α-amylase	OD at 560 nm n/3
	Residual activity %		Residual activity %	
control	100	1.219	100	0.730
Ni^{2+}	86	1.048	120	0.876
Ca^{2+}	102	1.243	123	0.897
Cu^{2+}	54	0.658	81	0.590
Co^{2+}	98	1.194	108	0.788
Zn^{2+}	63	0.767	76	0.554
Hg^{2+}	21	0.255	49	0.357
Pb^{2+}	57	0.694	70	0.511
