# Supplementary material for: α-Amylase immobilization on amidoximated acrylic microfibres activated by cyanuric chloride
Source: R Soc Open Sci. 2018 Nov 28;5(11):172164. doi: 10.1098/rsos.172164 (PMC6281920; doi:10.1098/rsos.172164)
Supplement: Supplementary Table 4 [file rsos172164supp8.pdf]

A-Amylase immobilization on amidoximated acrylic microfibers activated by cyanuric chloride

Yaaser Q. Almulaiky[1,2], Faisal M. Aqlan[3], Musab Aldhahri[4,5], Mohammed Baeshen[6] Tariq Jamal Khan[7], Khalid A. Khan[8], Mohamed Afifi[6,9], Ammar AL-Farga[1], Mohiuddin Khan Warsi[1], Mohammed Alkaled[6], Aisha A.M. Alayafi[6]

[1]Department of Biochemistry, Faculty of Science, University of Jeddah, Jeddah, Saudi Arabia
[2]Chemistry Department, Faculty of Applied Science, Taiz University, Taiz, Yemen
[3]Chemistry Department, Faculty of Science, University of Jeddah, Jeddah, Saudi Arabia
[4]Department of Biochemistry, Faculty of Science, King Abdulaziz University, Jeddah, Saudi Arabia
[5]Center of Nanotechnology, King Abdulaziz University, Jeddah, Saudi Arabia
[6]Department of biology, Faculty of Science, University of Jeddah, Jeddah, Saudi Arabia
[7]Stem Cell P2 Laboratory, The Center for Reproductive Medicine, Shantou University Medical College, Shantou, 515041, People‘s Republic of China
[8]Chemistry Department, Faculty of Science, King Abdulaziz University, Jeddah, Saudi Arabia
[9]Biochemistry Department, Faculty of Veterinary Medicine, Zagazig University, Egypt

Supplementary Table 4

Inhibitor effects on soluble and immobilized α-amylase.

Inhibitor 2mM	Soluble α-amylase	OD at 560 nm n/3	Immobilized α-amylase	OD at 560 nm n/3
	Residual activity %		Residual activity %	
EDTA	18	0.219	60	0.438
Sodium Citrate	45	0.548	70	0.511
Sodium Oxalate	59	0.719	76	0.554
1,10 phenatroline	60	0.731	88	0.642
DTNB	61	0.745	84	0.613
